# Molecular mechanism of topoisomerase poisoning by the peptide antibiotic albicidin

**DOI:** 10.1038/s41929-022-00904-1

**Published:** 2023-01-23

**Authors:** Elizabeth Michalczyk, Kay Hommernick, Iraj Behroz, Marcel Kulike, Zuzanna Pakosz-Stępień, Lukasz Mazurek, Maria Seidel, Maria Kunert, Karine Santos, Holger von Moeller, Bernhard Loll, John B. Weston, Andi Mainz, Jonathan G. Heddle, Roderich D. Süssmuth, Dmitry Ghilarov

**Affiliations:** 1 Malopolska Centre of Biotechnology, Jagiellonian University, Krakow, Poland; 2 Institut für Chemie, Technische Universität Berlin, Berlin, Germany; 3 Postgraduate School of Molecular Medicine, Warsaw, Poland; 4 moloX GmbH, Berlin, Germany; 5 Institut für Chemie und Biochemie, Freie Universität Berlin, Berlin, Germany; 6 John Innes Centre, Norwich Research Park, Norwich, UK

**Keywords:** Mechanism of action, Target validation, Structure-based drug design, Enzyme mechanisms, Antibiotics

## Abstract

The peptide antibiotic albicidin is a DNA topoisomerase inhibitor with low-nanomolar bactericidal activity towards fluoroquinolone-resistant Gram-negative pathogens. However, its mode of action is poorly understood. We determined a 2.6 Å resolution cryoelectron microscopy structure of a ternary complex between *Escherichia coli* topoisomerase DNA gyrase, a 217 bp double-stranded DNA fragment and albicidin. Albicidin employs a dual binding mechanism where one end of the molecule obstructs the crucial gyrase dimer interface, while the other intercalates between the fragments of cleaved DNA substrate. Thus, albicidin efficiently locks DNA gyrase, preventing it from religating DNA and completing its catalytic cycle. Two additional structures of this trapped state were determined using synthetic albicidin analogues that demonstrate improved solubility, and activity against a range of gyrase variants and *E. coli* topoisomerase IV. The extraordinary promiscuity of the DNA-intercalating region of albicidins and their excellent performance against fluoroquinolone-resistant bacteria holds great promise for the development of last-resort antibiotics.

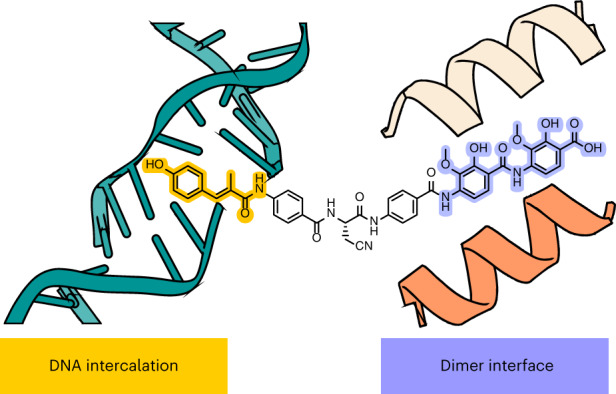

## Abstract (web summary)

Albicidin is a peptide antibiotic that has shown great promise for inhibiting DNA topoisomerase of fluoroquinolone-resistant Gram-negative pathogens, but its mode of action is not fully clear. Now, cryoelectron microscopy structures of albicidin–gyrase complexes provide detailed insights into the mechanism of this natural product.

## Main

The multidrug-resistant Gram-negative nosocomial pathogens (*Klebsiella pneumoniae*, *Acinetobacter baumannii*, *Pseudomonas aeruginosa*, *Enterobacter* spp., *Escherichia coli*, *Salmonella typhimurium*) present a pressing health burden, exacerbated by the COVID-19 pandemic^1,2^. *A. baumannii* and other Gram-negative infections are leading causes of high mortality in intensive care units, with some strains becoming pan-resistant^3^. This is a particular concern as the drug-discovery programmes of the major pharmaceutical companies based on high-throughput screening have not yielded any new compound class targeting Gram-negative bacteria in the last 50 years^4^. Combined with the high attrition rates of drug candidates in preclinical trials, this has resulted in a situation where new compound classes effective against Gram-negative bacteria that can enter the developmental pipeline are urgently required.

**Fig. 1:**
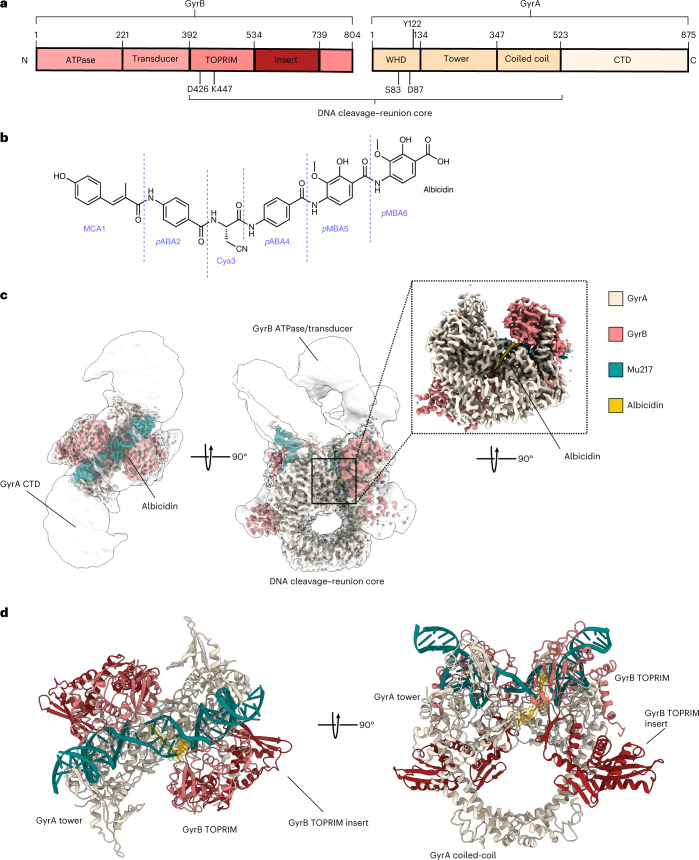
Structure of Gyr–Mu217–albicidin. **a**, Scheme of GyrA and GyrB domains. The DNA cleavage–reunion complex is shown, along with the catalytic residue (Tyr122) and residues involved in quinolone resistance (GyrB Lys447 and Asp426 and GyrA Ser83 and Asp87). A consistent colour code is used throughout the manuscript: beige, GyrA; coral, GyrB. **b**, Chemical structure of albicidin. **c**, An overview of the **Gyr–Mu217–albicidin** cryo-EM map depicted as an overlay of two different contour level maps. Low-resolution contour (white) illustrates the position of GyrA CTDs and GyrB ATPase domains. High-resolution core part, including albicidin (in the zoomed-in image), is coloured according to the scheme in **a**: coral, GyrB; beige, GyrA; teal, DNA; yellow, albicidin. **d**, Cartoon representation of the overall model.

Type II topoisomerase DNA gyrase is essential in bacteria and absent in humans; along with homologous topoisomerase IV (Topo IV) it is a major antibiotic focus, being the target of the widely used fluoroquinolones (FQs), the last class of broad-spectrum antibiotics to have been introduced into general clinical practice^5^. DNA gyrase is a heart-shaped heterotetrameric (A_2_B_2_) enzyme containing two GyrA and two GyrB monomers (Fig. 1a). It negatively supercoils (underwinds) bacterial DNA by creating a temporary double-strand break in the bound DNA segment (G or gate segment) through which it guides the adjacent (T or transported) segment of the same DNA, consuming ATP in the process. During the catalytic cycle, a GyrB-coordinated magnesium ion is required to cleave a phosphodiester bond in each DNA strand, with DNA 5**′** ends temporarily attached to the catalytic tyrosine residues of GyrA (Tyr122 in *E. coli*). As the release of double-strand breaks would be fatal for the cell, the cleavage of substrate DNA is tightly coupled with the opening/closing of protein–protein interfaces (gates) controlled by ATP binding and hydrolysis^6^. The effectiveness of FQs stems from their so-called gyrase poisoning mechanism which exploits an intrinsic DNA cleavage activity of gyrase by converting it into a toxin rather than simply inhibiting its catalytic action. FQs prevent DNA religation and stall the enzyme–DNA complex (cleavage complex) by intercalating into cleaved DNA, while forming a so-called water–metal ion bridge to Ser83 and Asp87 of GyrA (in *E. coli*)^5,7,8^. Consequently, the resulting FQ–protein–DNA adducts trigger the SOS response and cell death by a mechanism that probably involves processing of the cleavage complex to reveal the DNA breaks^9^. FQs remain remarkably effective, but side effects^10^ have restricted FQ use in the United States by a black box warning^11^ and there is widespread resistance^1^, prompting the search for new chemical leads. Of these, the most advanced clinically are ‘novel bacterial topoisomerase inhibitors’ (NBTIs) exemplified by gepotidacin, which is currently under investigation in two phase III trials for infections caused by Gram-negative bacteria^12,13^, and spiropyrimidinediones such as zoliflodacin^14^ which is currently in phase III clinical trials for treatment of patients with uncomplicated gonorrhoea. While many other compounds of the NBTI class are cardiotoxic due to the hERG (human ether-à-go-go-related gene) channel inhibition^10^, gepotidacin has only mild effects on heart rate^15^.

The hybrid polyketide–peptide antibiotic albicidin is produced by the Gram-negative plant pathogen *Xanthomonas albilineans* which causes devastating leaf scald disease in sugarcane^16^. Tissue necrosis and rapid plant death is the result of inhibition of DNA replication in chloroplasts—organelles of cyanobacterial origin with functional DNA gyrase^17–19^. Albicidin inhibits DNA gyrase at nanomolar concentration by stabilizing the cleavage complex^20^. Mode-of-action studies of albicidin have suffered from long-standing difficulties in accessing the compound and identifying its chemical structure. Recently we determined that albicidin is an elongated molecule consisting of six residues (Fig. 1b): a methyl *p*-coumaric acid (MCA1), *p*-aminobenzoic acid (*p*ABA2 and *p*ABA4), β-cyano-l-alanine (Cya3) and 4-amino-2-hydroxy-3-methoxybenzoic acid (*p*MBA5 and *p*MBA6)^21^. We have established synthetic routes for the parent compound^22^ and multiple derivatives^23–25^, including ones with improved characteristics and nanomolar-range activity against Gram-negative bacteria such as *E. coli*, *S. typhimurium* and *A. baumannii*. Importantly when considering eventual clinical use, these derivatives also demonstrated a good safety profile and efficacy in animal infection models using *E. coli*^26^. Albicidin resistance factors AlbA^27–29^, AlbD^30–32^ and AlbG^20,33,34^ were previously characterized by us and others. Structurally related gyrase inhibitors, cystobactamides, were also described in myxobacteria^35^. Despite these advances, the molecular mechanism and thus the targeted synthesis of rationally improved albicidins have so far remained elusive.

Here we applied cryoelectron microscopy (cryo-EM) to successfully determine several albicidin–gyrase structures, each requiring the full protein complex acting on an extended substrate DNA. Together with biochemical studies, our data illustrate how albicidins and other *para*-aminobenzoic acid-type natural products prevent DNA religation to achieve their antibiotic effect. The structures allow us to pinpoint the pharmacophores responsible for molecular recognition, and thus to rationally design variants of the toxin to overcome resistance mutations or to improve pharmacological properties of compounds such as their solubility, without affecting target binding. These findings are indispensable for the structure-guided development of albicidins as next-generation antimicrobial therapeutics.

## Results

### Requirements for cleavage complex stabilization by albicidin

All available crystal structures of DNA gyrase determined to date use the so-called gyrase cleavage core fusions, that is, truncated and fused constructs consisting of the C-terminal topoisomerase-primase (TOPRIM) domain of GyrB and the N-terminal part of GyrA (Fig. 1a). ATPase domains of GyrB and DNA-wrapping C-terminal domains (CTD) of GyrA, which are crucial for DNA supercoiling activity^36^, are absent in these protein constructs. Several attempts to co-crystallize core fusions with albicidin were not successful. Therefore, we switched to an alternative cryo-EM approach involving full-length *E. coli* gyrase and short DNA oligonucleotides reported by the Bax group^37^. However, we repeatedly failed to observe bound albicidin in the model.

**Extended Data Fig. 1:**
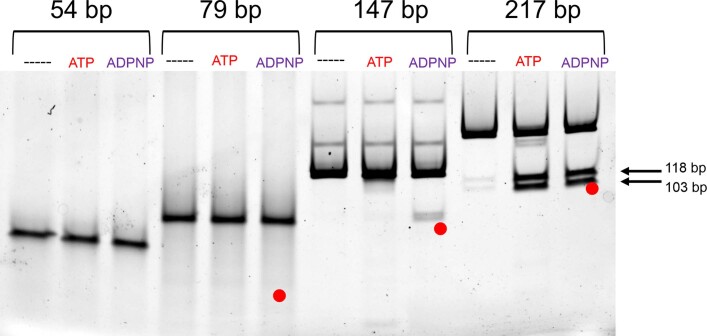
Length dependency of albicidin-mediated DNA cleavage. Position of cleaved DNA indicated by arrows and red dots. ATP and ADPNP were added as indicated, (----) stands for no nucleotide added. 20 nM gyrase, 1 µM albicidin and 25 nM DNA was used in each reaction.

We hypothesized that albicidin binding might require a strand-passage event to reveal the hidden inhibitor binding pocket. As it is known that stabilization of the gyrase cleavage complex by microcin B17 requires a long DNA segment (>150 bp) to be present^38,39^, we adopted a similar approach for albicidin. We thus tested a range of different DNA fragments, representing strong gyrase-binding sites (SGSs) of plasmid pBR322 or phage Mu, and found strong cleavage of the 217 bp Mu SGS fragment (Mu217) in the presence of albicidin, ATP or its non-hydrolysable analogue ADPNP. Importantly, almost no cleavage was detected with fragments shorter than 150 bp or in the absence of a nucleotide (Extended Data Fig. 1). A long DNA fragment able to engage the GyrA CTDs was thus essential to populate an albicidin-susceptible conformation of DNA gyrase. In summary, cleavage complex stabilization by albicidin critically depends on the presence of nucleotide and the length of the DNA substrate. We scaled-up the production of Mu217 (Methods) and used it to determine all the structures described below.

### Albicidin traps gyrase through asymmetric bicentric binding

**Extended Data Fig. 2:**
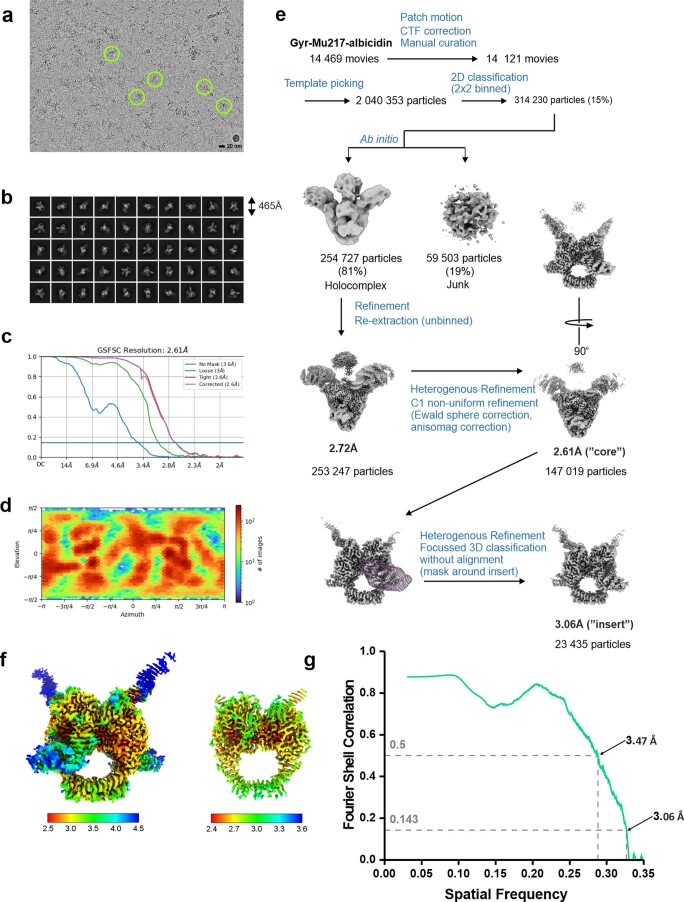
Cryo-EM data processing for Gyr-Mu217-albicidin. **a**. A representative micrograph with example gyrase particles indicated. **b**. A selection of 2D classes, box size in angstroms indicated. **c**. FSC curve for the highest-resolution reconstruction as outputted by cryoSPARC. **d**. Euler angle distribution as output by cryoSPARC. **e**. Processing scheme (see *Methods* for description). **f**. Local resolution maps contoured at two different levels (5σ; 15σ) to illustrate resolution distribution from >2.5 Å next to the DNA and compound to <4 Å at the ends of the TOPRIM insert. **g**. Map-to-model curve.

**Extended Data Fig. 3:**
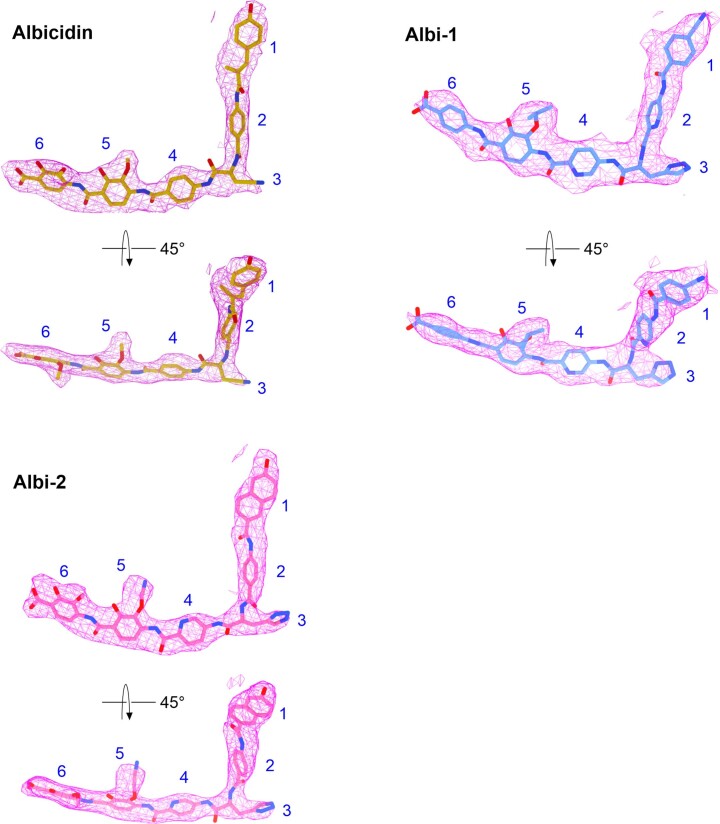
Coulomb potential density maps for albicidin and derivatives Albi-1 and Albi-2. Residue numbers are indicated. All maps are contoured at 11 σ level.

Cryo-EM micrographs of the gyrase–DNA–albicidin complex in the presence of ADPNP (**Gyr–Mu217–albicidin**) revealed high structural homogeneity and virtually no DNA static disorder. This allowed reconstruction of the enzyme cleavage core (residues 8–524 of GyrA and 405–804 of GyrB) at local resolution of 2.6 Å (Fig. 1c,d and Extended Data Figs. 2 and 3) and unambiguous assignment of the Mu217 DNA sequence, a dramatic improvement compared to the previously available data^40^. CTDs of GyrA with wrapped DNA and ATPase domains of GyrB were also observed but at much lower resolution (>5 Å) due to intrinsic flexibility (Fig. 1c) and were not modelled. The overall appearance of the holocomplex was similar to the only other available cryo-EM structure of *E. coli* gyrase in complex with gepotidacin (PDB: 6RKW and 6RKV)^40^. Apart from the DNA, the enzyme had almost perfect C2 symmetry, with ATPase domains centred roughly on the central axis of the complex above the GyrA/GyrA′ protein–protein interface (DNA-gate) with a small tilt towards one of the CTDs. The Mu217 DNA was cleaved, and the cleavage sites corresponded exactly to the positions known from biochemical^41^ or next-generation sequencing^42^ experiments (5′-**T/G**ATTT-3′, and 5′-**A/A**ATCA-3′ on the opposite strand; gyrase cleaves DNA leaving 4 bp overhangs).

**Fig. 2:**
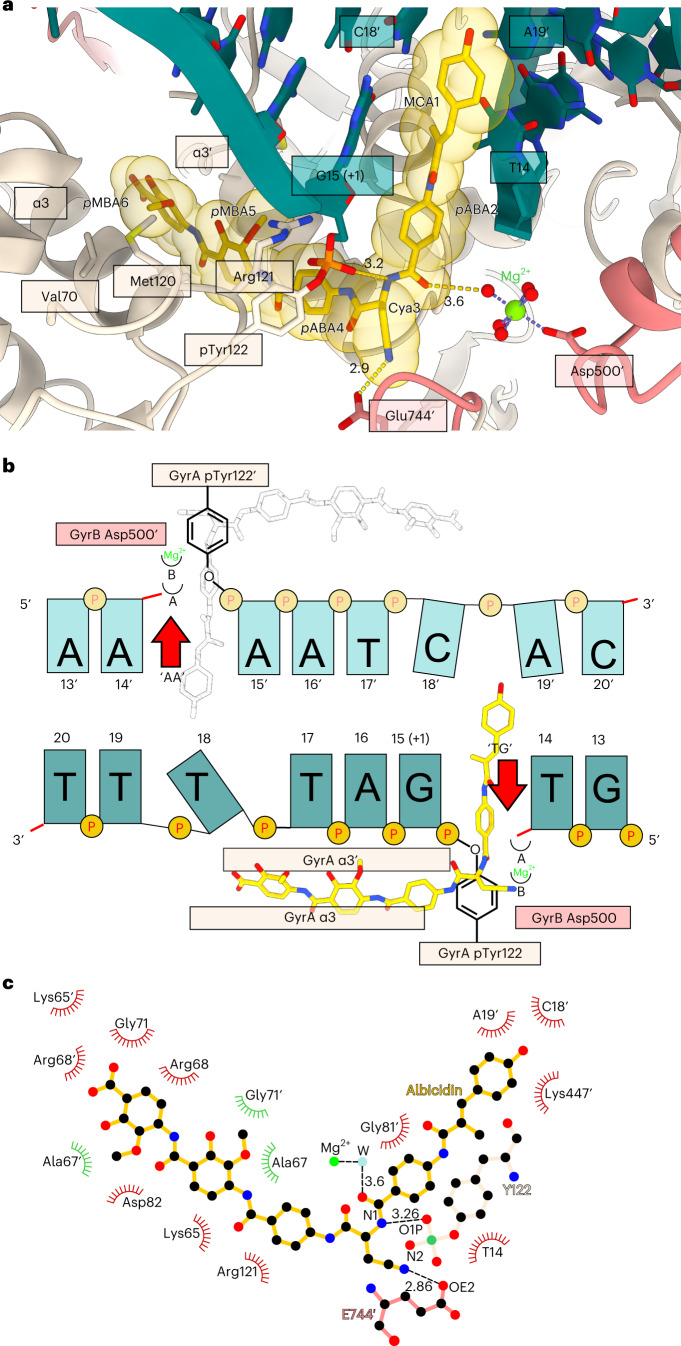
Albicidin-binding pocket. **a**, Enlarged view of the albicidin-binding site in the **Gyr–Mu217–albicidin** structure. Gyrase is represented as a cartoon, and albicidin as a stick representation. Van der Waals radii for albicidin atoms are shown as transparent yellow spheres. Two opposing GyrA helices α3 and α3′ at the dimer interface (DNA gate) form one part of the binding pocket, while DNA bases form another part. Distances (Å) between the modelled metal ion water shell and GyrB Glu744′ to Cya3 of albicidin are indicated. **b**, Schematic of albicidin binding in the context of the Mu cleavage site. Two potential binding pockets next to the scission sites ‘TG’ and ‘AA’ are labelled by the red arrows. Albicidin position is depicted by the sticks model (yellow), with the grey image indicating the potential alternative orientation not observed in the **Gyr–Mu217–albicidin** data. Two metal-binding sites, A and B, are indicated as half-circles. **c**, A LigPlot^76^ two-dimensional diagram of the albicidin-binding site. Hydrogen bonds and lengths (<4 Å) are indicated with dashed lines and the non-bonding and hydrophobic interactions (<4 Å) are labelled by the red and green spiked arcs, respectively. W, water coordinated to the metal (presumed Mg^2+^) ion.

**Extended Data Fig. 4:**
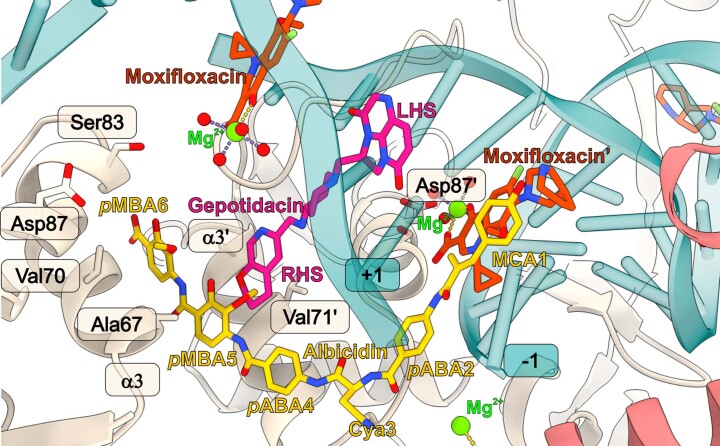
Comparison of albicidin, FQ and gepotidacin binding sites. **Gyr-Mu217-albicidin** structure is shown as cartoon representation with albicidin shown as yellow sticks. GyrA is shown in beige, DNA in teal and GyrB in coral. Catalytic Tyr122 and parts of GyrB are not shown for clarity. Albicidin residues, GyrA/GyrA’ interface (α3/α3’) and DNA bases next to the break (+1/-1) are indicated. To compare binding sites of different compounds, *E. coli* gyrase-gepotidacin cryo-EM structure (PDB:6RKS) and moxifloxacin (MFX) - *Staphylococcus aureus* crystal structure (PDB:5CDQ) were superimposed onto **Gyr-Mu217-albicidin** and aligned to the GyrA’ protomer using *match maker* in ChimeraX^71^. Two MFX molecules (MFX, MFX’) are shown as orange stick representations. A single bound gepotidacin molecule is shown as magenta sticks, with DNA-binding left-hand-side (LHS) and hydrophobic pocket binding right-hand-side (RHS) labelled. Note the overlap between the gepotidacin RHS and the methoxy group of *p*MBA5. MFX is bound via a so called ‘water–metal ion bridge’ to Ser83 and Asp87. The distance between the Asp87 and MFX in one protomer versus another illustrates large-scale movement of the albicidin-bound gyrase in comparison to the moxifloxacin-bound structure.

A single bound albicidin molecule was clearly observed in the Coulomb potential density map (Fig. 1c,d). The N-terminal end of the L-shaped molecule (residues MCA1 and *p*ABA2) intercalated between the cleaved DNA fragments right next to the phosphotyrosyl bond, between nucleobases T14 and G15 of the 5′-**T/G**ATTT-3′ non-palindromic cleavage site (Fig. 2a,b). The same ‘+1’ pocket is occupied by FQs and related compounds, although the binding mode is completely different (see Extended Data Fig. 4 for the comparison of symmetric FQ- and asymmetric gepotidacin- and albicidin-binding sites)^43^. The terminal hydroxyl of MCA reached C18 and A19 on the opposite DNA strand, located within 5 Å of GyrB Lys447. The requirement for intercalation of an oblong fragment explains the decreased activity of the kinked and thus shorter (*Z*)-MCA1 isomer of albicidin^44,45^. At the same time, the C-terminal segment of albicidin (*p*MBA5 and *p*MBA6) filled the space between two opposing helices α3 and α3′ (residues 66–76), forming the GyrA/GyrA′ dimer interface (DNA gate) (Fig. 2c). This required a substantial opening of the enzyme, evident by the sliding-door-like motion of the GyrA/GyrA′ monomers: the GyrA tower domain is shifted 10 Å outwards compared to the gepotidacin cryo-EM structure, while the aforementioned helix α3 slides by 5 Å and moves 3 Å away from its opposing monomer. Similar shifts were observed for GyrB including the TOPRIM domain and insert (Supplementary Fig. 1). Formation of the phosphotyrosine pTyr122 led to an 8 Å displacement of DNA ends (Supplementary Fig. 1). Our structure thus represents the previously unseen catalytic intermediate that can be placed between the partially open precleavage gepotidacin structure (PDB: 6RKV) and the fully open state (topo IIα structure, PDB: 5ZEN^46^ or open *Streptococcus pneumoniae* GyrA dimer^47^, PDB: 6N1P) (Supplementary Fig. 1 and Supplementary Movie 1). Interestingly, the position of GyrA CTDs also differed from earlier observations^40^. In our map, CTDs are moved considerably upwards in a symmetric fashion, nevertheless, the projected DNA path points away from the enzyme cavity (see Supplementary Fig. 2 for the rigid body fit). The observed position of CTDs might indicate the stage of the catalytic cycle at which the enzyme is trapped (Discussion).

In stark contrast to FQ-like molecules, which bind to the enzyme symmetrically, the N-terminal end of albicidin occupied only one-half of the DNA cleavage site: between T14 and G15-pTyr122, which we designate the TG pocket (3′T and 5′G in strand 5′-**T/G**ATTT-3′). This asymmetric binding caused pronounced DNA distortion in the symmetry-related AA pocket (between 3′A and 5′A in strand 5′-**A/A**ATCA-3′), which is smaller in size (Fig. 2). It is noteworthy that while theoretically possible, we did not observe even low-occupancy density for albicidin in this smaller AA pocket. Therefore, the size of the intercalating group in conjunction with the properties of DNA must impose selectivity towards the TG pocket. We are not aware of previous examples of such strong selectivity shown by any topoisomerase poison.

**Fig. 3:**
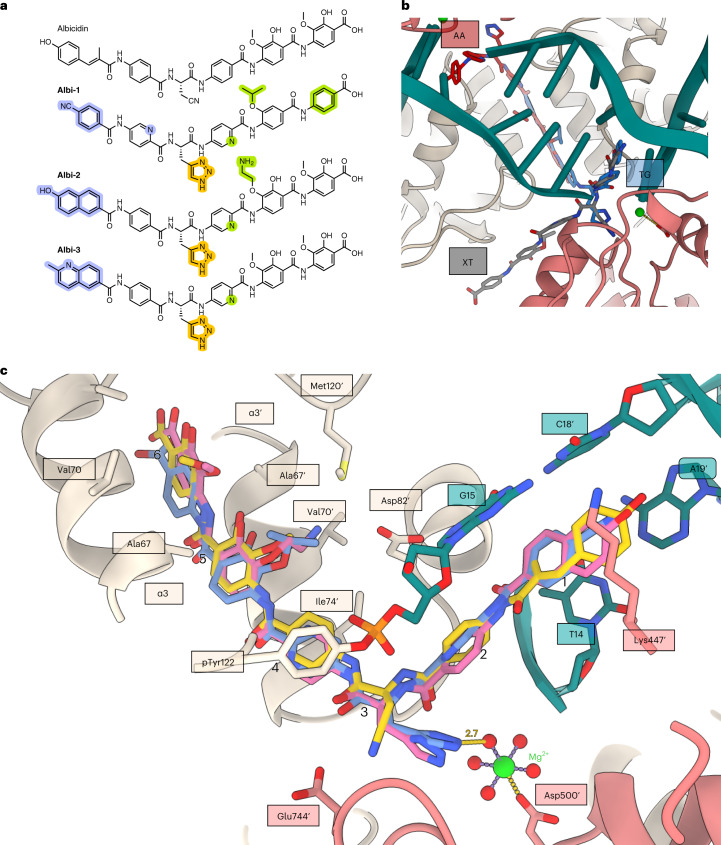
Binding of albicidin derivatives. **a**, Chemical structure of parent albicidin, **Albi-1**, **Albi-2** and **Albi-3**. Modifications in the N-terminal, central or C-terminal region of the molecule are highlighted in violet, orange or lime, respectively. **b**, An overlay of **Albi-1** (brick, blue or grey sticks) bound in three positions (TG, AA and XT) found in the cryo-EM density. **c**, Comparison of albicidin (gold)**, Albi-1** (blue) and **Albi-2** (pink) binding in the main (TG) binding pocket. Arabic numerals indicate the peptide residues numbers (Extended Data Fig. 7). To create the figure, GyrA subunits were aligned to the main **Gyr–Mu217–albicidin** model in ChimeraX^71^ and bound ligands shown in stick representation. Interacting residues of GyrA and GyrB are labelled, as is the distance (Å) between the triazole and the water shell of the Mg^2+^ ion.

Albicidin binding nevertheless exploited the symmetry of the enzyme in an elegant way, with the virtually identical residues *p*MBA5 and *p*MBA6 bound in a pseudosymmetric manner. The methoxy groups of *p*MBA5 and *p*MBA6 occupied hydrophobic pockets formed by side chains of GyrA Ala67, Val70 and Met120 and their counterparts from the opposing GyrA′ monomer (Fig. 2 and Fig. 3c). Interestingly, the same hydrophobic residues are also targeted by gepotidacin, but in an entirely different way (Extended Data Fig. 4). Residue Cya3 in albicidin, serving as a hinge point for the otherwise rather rigid molecule, was in close contact with catalytic Tyr122 of GyrA with a hydrogen bond forming between the N1 amide nitrogen and the scissile phosphate (3.3 Å). Another interaction was observed between the side-chain carboxylate of Glu744 (GyrB′) and the nitrile (N2) nitrogen of Cya3 (Fig. 2c). Apart from these hydrogen bonds, the majority of interactions are hydrophobic as albicidin precisely filled the available space between α3/α3′ helices or DNA bases. Therefore, binding of albicidin requires opening of the DNA gate and the precise positioning of the main gyrase catalytic residues, most probably occurring during a single round of DNA strand passage.

**Extended Data Fig. 5:**
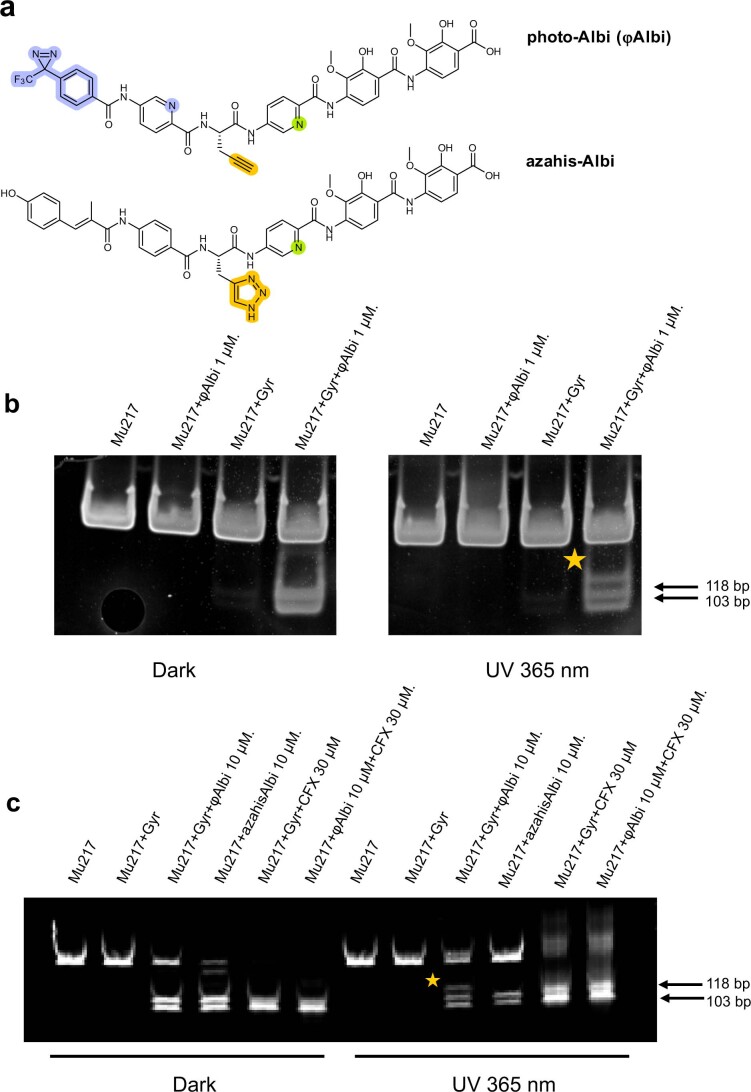
Photocrosslinking of diazirine-labelled albicidin (photo-Albi) to the DNA. **a**. Structures of N-terminal **photo-Albi** and control compound. **b**. DNA cleavage assay with Mu217 fragment and indicated concentrations of compounds. The labelled DNA band appearing upon irradiation of a photo-compound when bound to the complex is marked by a yellow star. **c**. Ciprofloxacin competition experiment. Pre-incubation with excess CFX prevents albicidin binding and crosslinking. Details of the experiment are available in *Methods* section.

We probed the binding mode of albicidin using an orthogonal method: we designed a photocrosslinkable analogue with an N-terminal diazirine (**photo-Albi**) that was found to be active against gyrase. Ultraviolet irradiation of the Gyr-Mu217–**photo-Albi** complex led to a shift in the position of the DNA band on a polyacrylamide gel (Extended Data Fig. 5). This shifted band did not appear upon irradiation of a non-photocrosslinkable analogue. Irradiation of a **photo-Albi**–DNA mixture also did not lead to crosslinking, therefore indicating that the N-terminus of **photo-Albi** is positioned to interact with cleaved DNA only when gyrase is present and catalytic conditions are satisfied. Addition of a molar excess of the FQ ciprofloxacin (CFX) outcompeted **photo-Albi** and precluded the appearance of the shifted DNA band, in agreement with the partially overlapping binding loci of FQs and albicidin (the TG pocket) (Extended Data Fig. 4).

**Extended Data Fig. 6:**
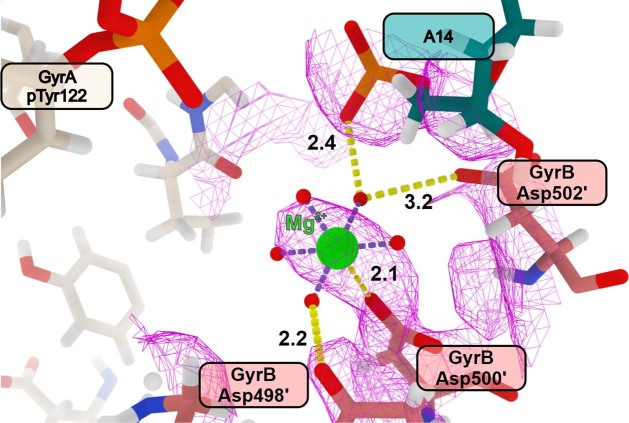
Metal binding site in Gyr-Mu217-albicidin. The water shell around the postulated Mg^2+^ ion was modelled and refined with ideal geometrical restraints (see *Methods*). Map density contoured at 9σ level. Distances in Å to the closest atoms are indicated.

There has been an ongoing debate regarding the exact mechanism of metal-ion-dependent DNA cleavage employed by type II topoisomerases; various crystal structures have been analysed and a range of occupancies at two metal-binding sites, termed A and B, have been reported^43,48^. Analysis of these structures has been complicated by the symmetry-related disorder present in many of them. In addition, different metals (Mg^2+^, Mn^2+^, Zn^2+^) have been used to obtain crystals which can considerably affect the interpretation. In our structure, a long substrate DNA is wrapped around GyrA CTDs, bound in a single orientation, and cleaved as the result of enzymatic activity, allowing us to observe a clear homogeneous state before DNA religation takes place. As the complex was prepared in 2.5 mM Mg(OAc)_2_ (Methods) we assigned Mg^2+^ as the most probable identity for the observed single, well-defined density stretching from Asp500 of both GyrB protomers in the complex. The shape of the density was consistent with the expected octahedral coordination sphere (Fig. 2a,b and Extended Data Fig. 6). Partial opening of the DNA gate in our structure led to movement of GyrB subunits further apart, with the metal ion still being attached to Asp500. Thus, although the actual position of the metal was not directly equivalent to the one observed in the previously reported crystal structures, it nevertheless corresponded to the B configuration used to temporarily store the metal during strand passage^43^. As our structure most probably represents the conformation before the religation step (Discussion), the likely interpretation is that the metal ion remains attached to residue Asp500 throughout the catalytic cycle of DNA gyrase. This might help to anchor the nucleophilic 3′OH group of the cleaved DNA to the GyrB TOPRIM domain and ultimately, to GyrA via the latter’s N-terminal helix. Although the observation cannot be considered as a definite proof, our results are thus compatible with the single-ion mechanism, where the metal ion is stored in the so-called B configuration for a large part of the catalytic cycle while the DNA is cleaved, but moves towards the pTyr122 to catalyse religation. We cannot, however, exclude a temporary recruitment of a second metal ion immediately before the religation or cleavage takes place.

### Potentiated albicidin derivatives show binding heterogeneity

**Extended Data Fig. 7:**
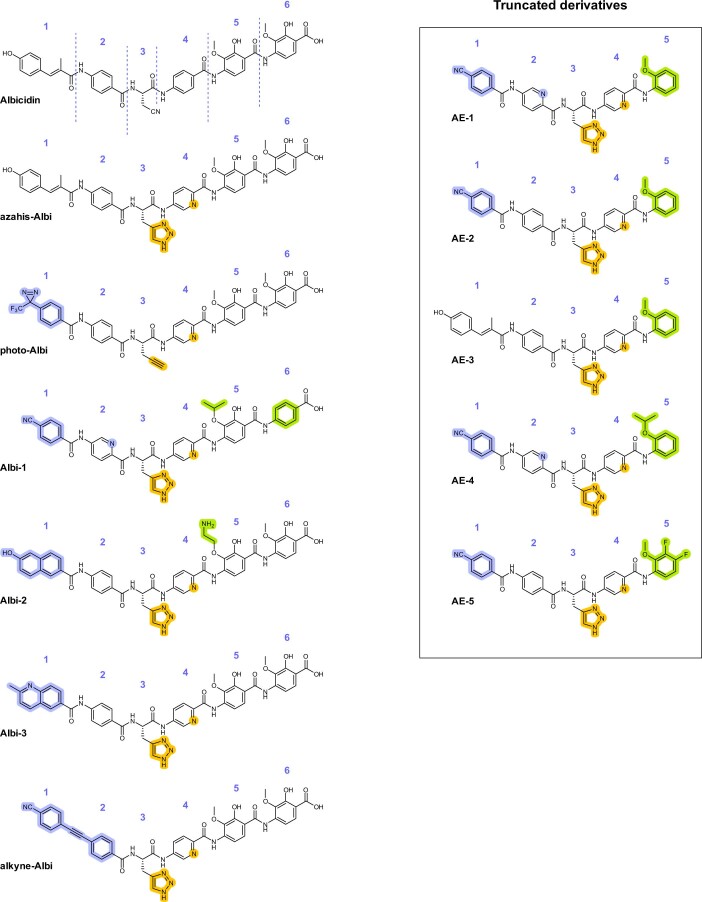
Structures of all albicidin derivatives used in this study. Individual residues are marked as in Fig. 1.

**Extended Data Fig. 8:**
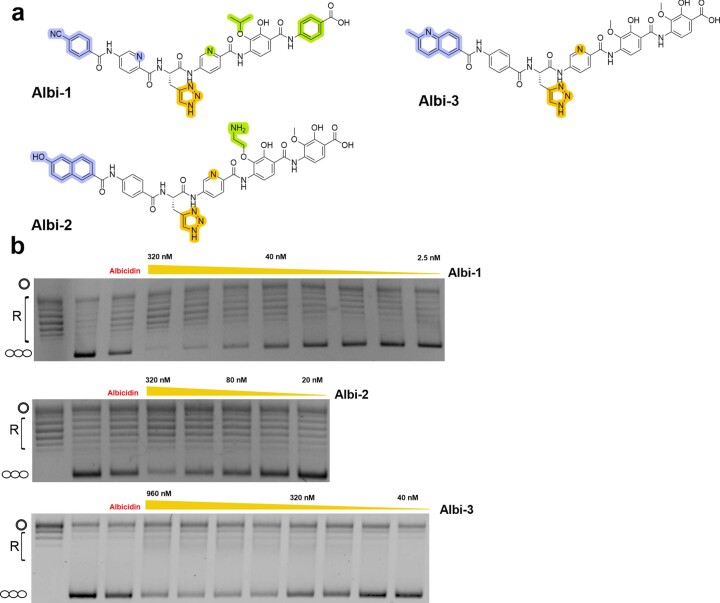
Inhibition of supercoiling by Albi-1/2/3. **a**. Chemical structures of **Albi-1**, **Albi-2** and **Albi-3**. **b**. Example supercoiling inhibition assay gels: **Albi-1**, decreasing concentrations (320, 160, 80, 40, 20, 10, 5, 2.5 nM); **Albi-2** (320, 160, 80, 40, 20 nM); **Albi-3** (960, 800, 640, 480, 320, 160, 80, 40 nM). First lane - relaxed pBR322; second lane - DNA gyrase. 40 nM albicidin was used as positive control (third lane). Different concentrations were tested and representative gels are shown.

**Extended Data Fig. 9:**
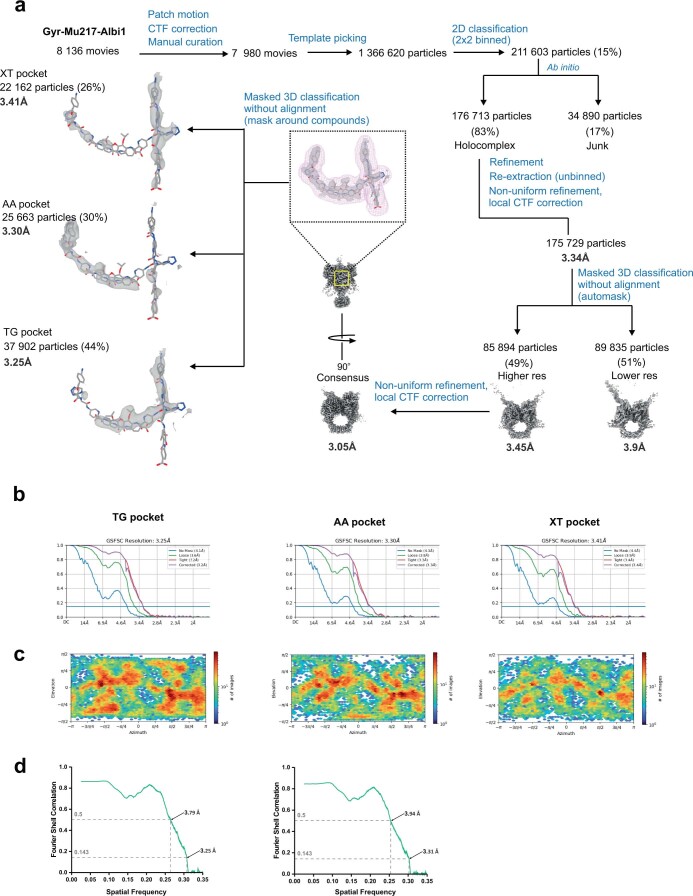
Cryo-EM data processing for Gyr-Mu217-Albi-1. **a**. Processing scheme (see *Methods* for description). **b**. FSC curves as output by cryoSPARC. **c**. Euler angle distributions as output by cryoSPARC. **d**. Map-to-model plots.

Encouraged by our results, we moved to further characterize the structure–activity relationship (SAR) of *para*-aminobenzoic acid antibiotics. We started from a potentiated albicidin derivative **Albi-1** (Fig. 3a and Extended Data Fig. 7) with features leading to improved pharmacological characteristics and nanomolar-range activity towards FQ-resistant pathogens^26^ (a minimum inhibitory concentration (MIC) ≤ 0.016 µg ml^−1^ for *E. coli;* see also Table 1 and Extended Data Fig. 8). This compound has the residues *p*ABA2 and *p*ABA4 substituted by pyridines, aza-histidine replacing Cya3, and an isopropoxy moiety in residue 5. The compound also has the N-terminal MCA1 replaced by *p*-cyanobenzoic acid, resulting in a shortened molecule. Initial analysis of collected cryo-EM data (**Gyr–Mu217–Albi-1**) converged with a consensus ensemble map consistent with a mixture of states in which **Albi-1** occupied either the AA or the TG pocket with partial occupancy (AA and TG states). Moreover, an additional state of low occupancy was populated with the N-terminal segment of **Albi-1** intercalated to the TG pocket, but with its C-terminal half oriented externally (XT state) and pointing towards the protein surface, instead of wedging the GyrA/GyrA′ dimer interface (see Extended Data Fig. 9 for data processing and Extended Data Fig. 3 for compound density). As it was not sterically possible for these states to coexist in the same molecule, we used a masked three-dimensional (3D) classification to separate the data into three maps, representing the three binding modes (Fig. 3b and Extended Data Fig. 9). We refined models for AA and TG binding states separately, while the XT state density was not sufficient for unambiguous refinement. Hence, we only tentatively modelled the XT-bound state based on the rings’ orientation observed in all other structures to obtain the likely conformation depicted in Fig. 3b. AA and TG maps also suffered from orientation bias and low number of particles; however, the density was sufficiently clear to allow unambiguous compound modelling. In both reported (AA and TG) **Albi-1** models, the incorporated aza-histidine group was directed towards the catalytic Mg^2+^ ion at a 2.7 Å distance between the presumed metal-coordinated water and the N2 atom of the triazole. We believe the coordination provided by the triazole might additionally stabilize the bound state. Further stabilization is provided by the larger isopropoxy group in residue 5 that can better occupy hydrophobic pockets at the dimer interface (Fig. 3c) and the pyridine N9 atom of residue 2 orienting towards the phosphate of pTyr122 at a 3.4 Å distance (Fig. 3c). As the residue 6 of **Albi-1** does not have methoxy or isopropoxy substituents, it appears that a single isopropoxy group on residue 5 is sufficient to anchor the compound in the hydrophobic pocket.

**Table 1:** MIC (µg ml^−1^) of albicidin derivatives used in this study against selected Gram-negative and Gram-positive microorganisms (see Extended Data Fig. 7 for structures of all compounds)

	*E. coli* BW25113	*E. coli* DSM116	*B. subtilis* DSM10	*M. luteus* DSM1790	*M. phlei* DSM750	*S. typhimurium* TA100
Albicidin^a^	0.063 0.063	0.063 0.063	<0.25 0.25	1.0 1.0	2.0 2.0	0.063
**Azahis–Albi**^a^	<0.016 <0.016	<0.016 <0.016	0.031 0.063	0.031 0.031	0.125 0.5	<0.016 <0.016
**Albi-1**^a^	<0.016 <0.016	0.031 0.031	0.031 0.063	<0.016 <0.016	0.25 0.5	<0.016 <0.016
**Albi-2**^b^	4.0 4.0	2.0 4.0	>8.0 >8.0	>8.0 >8.0	>8.0 >8.0	0.5 0.5
**Albi-3**^a^	<0.016 <0.016	<0.016 <0.016	0.063 0.125	0.063 0.063	0.5 1.0	<0.016 <0.016
**Alkyne–Albi**^a^	<0.016 <0.016	<0.016 <0.016	0.063 0.125	<0.016 <0.016	0.25 0.5	<0.016 <0.016
**AE-1**^c^	>8.0 >8.0	>8.0 >8.0	>8.0 >8.0	>8.0 >8.0	>8.0 >8.0	>8.0 >8.0
**AE-2**^c^	>8.0 >8.0	>8.0 >8.0	>8.0 >8.0	>8.0 >8.0	>8.0 >8.0	>8.0 >8.0
**AE-3**^c^	>8.0 >8.0	>8.0 >8.0	>8.0 >8.0	>8.0 >8.0	>8.0 >8.0	>8.0 >8.0
**AE-4**^c^	>8.0 >8.0	>8.0 >8.0	>8.0 >8.0	>8.0 >8.0	>8.0 >8.0	>8.0 >8.0
**AE-5**^c^	>8.0 >8.0	>8.0 >8.0	>8.0 >8.0	>8.0 >8.0	>8.0 >8.0	>8.0 >8.0

One or two independent biological replicates are reported, with each done in technical triplicates. Superscript letters indicate three activity groups: ^a^most potent; ^b^intermediate; ^c^least active.

The observation that a shorter N-terminal *p*-cyanobenzoic acid of **Albi-1** allowed it to occupy both the TG and AA pockets, along with the tolerance for hydroxy/methoxy groups in residue 6, prompted us to synthesize C-terminally truncated derivatives with the idea that two copies of shortened molecules could bind symmetrically and simultaneously to the gyrase homodimer. We removed *p*MBA6 and used only a substituted aniline as a C-terminal building block to avoid steric hindrance between the potentially opposing binders (Extended Data Fig. 7, AE-series). We observed severely decreased activity for these compounds in both MIC and gyrase assays (Table 1 and Supplementary Fig. 9). Therefore, both residues 5 and 6 are important to block the DNA gate and trap DNA gyrase. In contrast, the N-terminal arm of albicidin, which does not make any specific protein contacts apart from Lys447 of GyrB, could clearly endure substantial refactoring with the main requirement of being flat and providing *π*–*π* stacking interactions with DNA bases. Indeed, we found the alkyne derivative **alkyne–Albi**, which is resistant to (*E*)–(*Z*) photoisomerization^44^, to be as active as albicidin in a DNA cleavage assay (Table 1, Extended Data Fig. 7 and Supplementary Fig. 9).

**Fig. 4:**
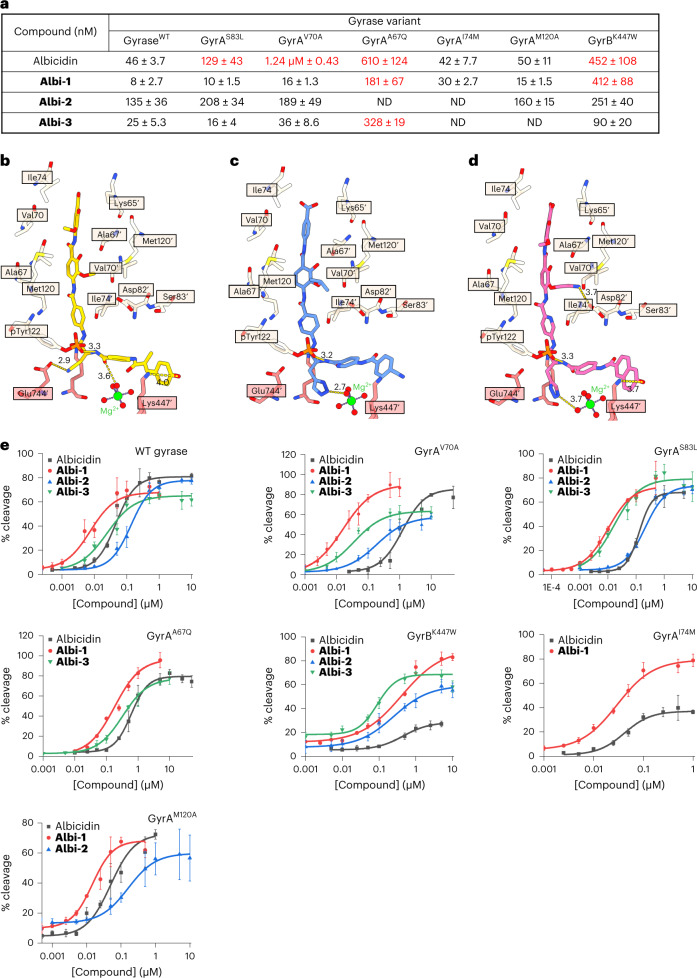
Effects of GyrA and GyrB mutations on susceptibility to albicidins. **a**, CC_50_ values for albicidin, **Albi-1**, **Albi-2** and **Albi-3** determined for WT *E. coli* gyrase and selected mutants. The mutations that notably increased resistance to the compound are shown in red. **b–d**, Mutated residues of GyrA (beige) and GyrB (coral), and albicidin (**b**, gold), **Albi-1** (**c**, blue) and **Albi-2** (**d**, pink) in stick representation. Main interactions are shown with the corresponding distances in Å. **e**, Plots used for CC_50_ determination. Data plotted are means of triplicate measurements; error bars represent s.d. ND, not determined. Source data

**Extended Data Fig. 10:**
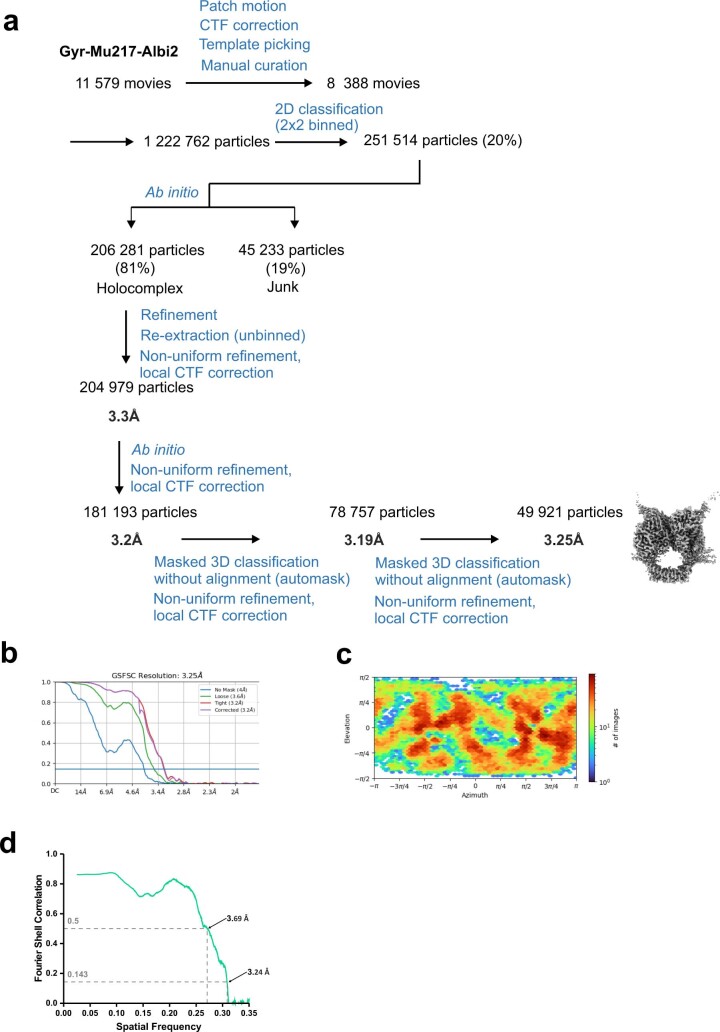
Cryo-EM data processing for Gyr-Mu217-Albi-2. **a**. Processing scheme (see *Methods* for description). **b**. FSC curve as output by cryoSPARC. **c**. Euler angle distribution as output by cryoSPARC. **d**. Map-to-model plot.

Taking account of these data, we synthesized two further compounds **Albi-2** and **Albi-3 (**Fig. 3a and Extended Data Fig. 7) where we replaced residue MCA1 of albicidin by decorated quinoline or naphthalene moieties with known ability to intercalate in DNA as flat aromatic molecules^49,50^. With **Albi-2** we additionally explored electrostatic interactions with GyrA Asp82 by replacing the methoxy group of *p*MBA5 with an aminoethyl. **Albi-3** was found to be highly bactericidal (MIC for *E. coli*, <0.016 µg ml^−1^), whilst **Albi-2** showed severely diminished activity compared with both **Albi-3** and albicidin in MIC assays (4 µg ml^−1^, that is, 60-fold increase in MIC for *E. coli* compared with the parent compound; Table 1). However, **Albi-2** had ~4-fold better activity in the gyrase inhibition assay than **Albi-3**, indicating that the aminoethyl moiety compromises cell entry (Table 1, Fig. 4a, Extended Data Fig. 8 and Supplementary Fig. 8). Therefore, to better understand the effects of different structural variations of albicidin, we selected **Albi-2** rather than **Albi-3** to determine its conformation in the gyrase–DNA complex (**Gyr–Mu217–Albi-2**) at 3.25 Å resolution (Fig. 3c and Extended Data Figs. 3 and 10). The binding of **Albi-2** displayed a degree of heterogeneity, but in contrast to **Albi-1**, the vast majority of particles fell into a single class corresponding to the occupied TG site (that is, similar to parent albicidin). We did not try to resolve alternative binding modes in this case. It seemed that the size of the N-terminal segment indeed controlled the positioning of the compound, with the larger N-terminal residues of albicidin and **Albi-2** preventing intercalation into the smaller AA pocket. The amino group introduced in residue 5 of **Albi-2** was not found to interact with Asp82 of GyrA, but instead rotated away towards the hydroxy group of residue 5. Non-optimal contact with GyrA Met120 and exclusion from a largely hydrophobic pocket is the likely reason.

### Site-directed mutagenesis confirms albicidin’s binding mode

It was impossible to select for natural albicidin-resistant gyrase variants due to the exclusive emergence of knock-out mutants of the nucleoside transporter Tsx, which is a known entry point of albicidin into Gram-negative bacteria^51^. We also could not observe any mutants when Tsx was externally supplemented on a pBAD plasmid. We therefore designed several gyrase mutants based on the **Gyr–Mu217–albicidin** structure to specifically impact albicidin binding. We also aimed to determine the effect of clinically relevant FQ-resistance mutations on albicidin’s ability to inhibit gyrase. Hence, we targeted the main albicidin binding pocket, that is, the α3/α3′ interface between GyrA monomers, and other neighbouring GyrA and GyrB residues (Fig. 4 and Supplementary Table 3). To avoid introducing substitutions not existing in nature, we used multiple sequence alignments of GyrA subunits provided by the ConSurf server^52^. We then produced the gyrase variants, tested their enzymatic activity in supercoiling and cleavage assays and determined CC_50_ (concentration required to achieve half of maximum DNA cleavage) values for albicidin and its derivatives **Albi-1–3** (Fig. 4 and Supplementary Figs. 3–8).

Three GyrA mutations affected gyrase activity: GyrA^A67Q^ showed a 4-fold decrease in activity in both supercoiling and cleavage assays (Supplementary Figs. 3 and 5), while mutations GyrA^R68A^ and GyrA^M120A^ affected the supercoil setpoint of the enzyme (induced relaxation of negatively supercoiled DNA substrate in the presence of ATP) (Supplementary Figs. 3 and 4). This relaxation effect could be ascribed to the weakening of the GyrA/GyrA′ interface, potentially affecting the directionality of strand passage. Interestingly, the variant GyrA^D82N^, previously reported to decrease DNA cleavage with FQs and related drugs^53^, had no visible effect on gyrase cleavage activity in our hands nor did it affect cleavage with **Albi-1** (Supplementary Figs. 3, 4 and 7).

The FQ-resistance mutation GyrA^S83L^ led to a modest 3-fold increase in CC_50_ values for the parent compound and a small decrease in the maximum amount of cleavage observed, in line with previous observations^20^, but had virtually no effect on activity of improved derivatives **Albi-1** and **Albi-3** (Fig. 4a,e and Supplementary Figs. 7 and 8). This is a striking result, because the majority of clinically relevant resistant variants are the alterations of GyrA Ser83 or Asp87^5,54,55^. Unlike FQs, binding of albicidins is not controlled by the metal-dependent anchoring to GyrA Asp87 and Ser83, meaning that albicidins are unaffected by the quinolone-resistance determining region (QRDR) of GyrA. In contrast, when the main hydrophobic α3/α3′ binding pocket was altered in variants GyrA^A67Q^ (smaller and more polar) and GyrA^V70A^ (larger), the CC_50_ for albicidin increased more than 10- and 25-fold, respectively. Testing the resistance of these mutants to **Albi-1** and **Albi-3** showed that GyrA^V70A^ is fully susceptible to these derivatives, while GyrA^A67Q^ is still resistant (Fig. 4a and Supplementary Figs. 4, 7 and 8). Loss of susceptibility to the Val to Ala substitution can be rationalized by stronger binding as a result of a bulkier isopropoxy group (**Albi-1**) or a quinoline core (**Albi-3**). At the same time, the steric hindrance introduced by the Ala to Gln replacement dramatically increased resistance to all albicidins (Fig. 4). GyrA^I74M^, GyrA^D72N^ and GyrA^M120A^ did not show increased resistance to either **Albi-1** or albicidin (Fig. 4a and Supplementary Figs. 4 and 7) judged by the CC_50_, but GyrA^I74M^ demonstrated ~2-fold reduction in the maximal amount of cleavage observed with the parent compound (Supplementary Table 4).

In GyrB, we targeted three residues: Lys447, implicated in FQ resistance^56^, and located 4 Å from the N-terminus of albicidin, Glu744 and Lys740 (Fig. 4b). Variants GyrB^E744A^ and GyrB^K740A^ were incapable of fully supercoiling DNA, with the GyrB^K740A^ variant causing increased DNA cleavage, and therefore were not used in further assays (Supplementary Figs. 3, 5 and 6). For Lys447 we initially screened three naturally occurring substitutions (Arg, Glu and Trp) (Supplementary Figs. 3 and 4 and Supplementary Table 3) and selected GyrB^K447W^ for further assays because of more pronounced effects. GyrB^K447W^ showed a 10-fold increase in CC_50_ compared with the parent compound and a more than 50-fold increase relative to the **Albi-1**; it also decreased the maximal cleavage observed with the parent compound 3-fold (Supplementary Table 4). However, the same variant was much more susceptible to the quinoline analogue **Albi-3** with a bulkier bicyclic residue 1 (CC_50_ = 90 nM, 69% maximal DNA cleavage) and to **Albi-1 (**Fig. 4a,c, Supplementary Figs. 5, 7 and 8 and Supplementary Table 4**)**.

As the aminoethyl derivative **Albi-2** was slightly less active (CC_50_ = 135 nM) against wild-type (WT) gyrase compared to parent albicidin (Fig. 4a and Supplementary Fig. 8), we only tested quinolone-resistant variants GyrA^S83L^ and GyrB^K447W^, and variant GyrA^V70A^ which has shown high resistance to parent albicidin. Surprisingly, none of the mutations substantially increased resistance to **Albi-2** measured by the CC_50_ although the maximal amount of cleavage was lower than with other compounds (Fig. 4e and Supplementary Table 4). Additionally, in a bid to understand the lower activity of the compound, we tested the GyrA^M120A^ variant which reduces the potential steric hindrance caused by the aminoethyl moiety (Fig. 3a). However, the CC_50_ of **Albi-2** against GyrA^M120A^ was not affected by the mutation (Fig. 4a and Supplementary Fig. 8). The probable reason for the lower activity is the exclusion of the amine from the hydrophobic pocket.

We demonstrated that the activity of albicidin derivatives remains almost completely unaffected by the known mutations in the QRDR of GyrA that confer quinolone resistance. While the Lys447 mutation in GyrB QRDR offered some resistance, especially to the parent compound, its effect could be successfully overcome by the alterations of the DNA-intercalating moiety as demonstrated by **Albi-3**. While the designed GyrA^A67Q^ mutation provided some resistance to **Albi-1** or **Albi-3**, this variant is unlikely to naturally evolve as it disrupts gyrase function. Notably, we could not generate this or any other gyrase mutation in selection screens.

### Albicidins target *E. coli* Topo IV

One of the reasons for the difficulties encountered in selecting resistant mutants for albicidin could be dual-targeting, that is, the ability to target Topo IV alongside gyrase in bacteria. To test this possibility, we carried out initial *E. coli* Topo IV activity and cleavage complex stabilization assays in the presence of albicidin, **Albi-1**, **Albi-2** and **Albi-3** (Supplementary Fig. 11). All compounds, with the exception of **Albi-2**, could stabilize the cleavage complex with Topo IV, the most potent being **Albi-1**. A linear DNA band could be observed at 1 µM concentration of **Albi-1** compared with 10 µM required for the parent compound. In the inhibition assays, 10 µM of **Albi-1** and **Albi-3** was required for full inhibition of Topo IV relaxation activity, compared with 100 µM for **Albi-2** or the WT albicidin. Therefore, the albicidin derivatives described in this study can clearly inhibit Topo IV, albeit with lower efficiency than DNA gyrase, and chemical modifications can additionally increase the potency of these derivatives against this target.

## Discussion

Natural product inhibitors of DNA gyrase represent a largely untapped reservoir of molecules that have the potential to be developed into novel classes of highly effective antibiotics^57^. To achieve this, a structural understanding of their mechanisms of action is very useful. Traditionally, such structural studies have employed a crystallographic approach where truncated, stabilized constructs and carefully optimized DNA oligonucleotides were developed to determine structures of FQs, NBTIs and other small molecules^43^. Despite many successes, it seems that these systems are not suitable for inhibitors that, similarly to albicidin, target transient conformations of topoisomerases only sampled during the enzyme’s catalytic cycle in quasi-native conditions. At the same time, cryo-EM allowed us to successfully determine the mechanism of action of this natural product, discovered more than three decades ago.

In this work we have shown that *para*-aminobenzoic acid antibiotics form a special class of topoisomerase inhibitors. Unlike multiple known gyrase poisons that, similarly to FQs, symmetrically intercalate between the cleaved DNA fragments, preventing religation of both DNA strands, a single molecule of albicidin binds asymmetrically to the gyrase tetramer and intercalates only from one side of the DNA break. At the same time, wedging of the GyrA/GyrA′ interface by the C-terminal fragment of albicidin efficiently prevents the protomer movement required for religation of the opposite DNA strand. Both DNA-binding and protein-binding fragments of the inhibitor are thus essential to stall the enzyme, as evidenced by our data with truncated derivatives.

**Fig. 5:**
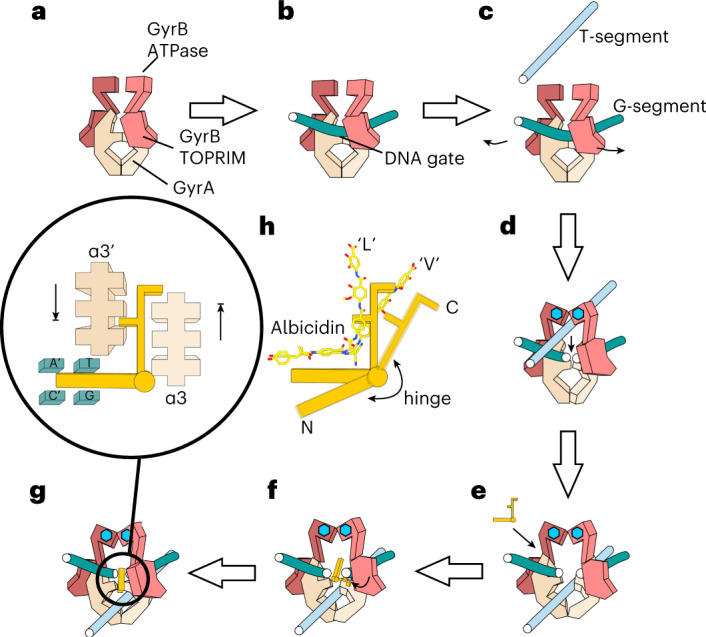
Mechanistic model of gyrase inhibition by albicidin. **a**, Initial state: an apo-gyrase complex. **b**, G-segment DNA fragment is engaged and bound. **c**, T-segment DNA fragment is captured by the ATPase domains of GyrB and the DNA is cleaved. **d**, The T-segment is transported through the enzyme, stimulated by the ATP binding (blue hexagons), causing dimerization of the ATPase domains. **e**, The T-segment is transported to the bottom chamber of the enzyme and the DNA has to be religated to release the T-segment. Albicidin intercalates in DNA in outer-rotated conformation (XT state). **f**, Albicidin rotates and occupies the binding pocket between two GyrA monomers. **g**, Albicidin effectively jams the enzyme movement, blocking the escape into any productive state. Inset: an enlarged scheme of albicidin–DNA–gyrase interaction in the locked state. **h**, Scheme of albicidin molecule, consisting of two rigid fragments (N- and C-terminal), connected by a flexible hinge residue, While in our structure the compound has L shape (‘L’), it adopts a more open wide-angle V shape (‘V’) in complex with the AlbA resistance protein (PDB: 6ET8)^27^.

According to our proposed mechanism for the inhibition of the catalytic cycle of gyrase (Fig. 5), the GyrA/GyrA′ binding pocket utilized by albicidin forms only transiently during large-scale conformational changes of the enzyme associated with DNA strand passage. This raises the question of how albicidin is able to recognize the transient site so efficiently and remain stably bound. Our structure was obtained with the use of ADPNP, a nucleotide analogue that stabilizes gyrase after T-segment capture. Albicidin binding was strongly stimulated by a long (>150 bp) DNA fragment. We conclude that the albicidin-bound state most probably represents the conformation after strand passage (Fig. 5). Indeed, if the compound were to bind before strand passage (for example, at steps b or c in Fig. 5), it would be pushed away by the captured T-segment and subsequent opening of the DNA gate. It has been proposed by Schmidt et al.^48^ that in order to preserve its integrity, cleaved DNA is religated before the C-terminal gate opens and releases the T-segment (Fig. 5, step e). We suggest that albicidin binds at this stage. Based on the structure of **Albi-1** in the XT state, we believe that albicidin might initially bind as a DNA intercalator using its N-terminal segment (Fig. 5, step f), while its C-terminal arm is able to freely rotate around the central hinge residue. Once the DNA-gate pocket is formed by the enzyme closing and trying to religate, the C-terminal arm swings into place, jamming the enzyme and preventing the ‘sliding doors movement’ in any direction (Fig. 5, step g). In the absence of the religation event, gyrase cannot proceed to the next catalytic cycle, remaining in a trapped state. Albicidin is most active with ATP present, and therefore nucleotide binding and hydrolysis on its own is not sufficient to dislodge the drug and escape into the productive state. The inhibitory mechanism of albicidin thus could be compared to a spanner thrown between two rotating gears. In this respect it is interesting to consider the pentapeptide repeat protein (PRP) AlbG that provides protection against gyrase poisoning by albicidin^20,33,34^. This protein and other PRPs were suggested to act as T-segment DNA mimics^58,59^. The AlbG protein transported through the gyrase can lead to the GyrA/GyrA′ interface opening up and dislodging the bound poison—thus interaction with a specialized protein is required to exit the trapped state. The heavily post-translationally modified peptide toxin microcin B17 is another DNA gyrase inhibitor that requires DNA strand passage for activity. Analogously to cystobactamide (CysO)^60^ and albicidin (AlbG), a PRP McbG providing toxin immunity is located within the microcin B17 biosynthetic gene cluster^61^. This points to a likely similar mechanism for microcin B17 action wherein it may also trap a temporarily open gyrase complex.

In summary, our work provides the structural framework explaining multiple previous observations based solely on chemical synthesis and activity profiling. Albicidin derivatives, particularly of the **Albi-1** or **Albi-3** type, have already demonstrated safety and efficacy in animal infection models^26^. The α3/α3′ binding pocket is unique and does not overlap with any other known classes of topoisomerase inhibitors. The most potent derivatives **Albi-1** and **Albi-3** have nanomolar activity in cleavage complex stabilization against Gram-negative *E. coli* gyrase, and are more potent than NBTIs. Compared to the parent compound, they also stabilize a high proportion of cleaved complexes for all tested gyrase variants apart from the WT which means albicidins can be successfully chemically modified to escape target-mediated resistance. Similarly to FQs, albicidins target both gyrase and Topo IV in bacteria, which makes resistance harder to develop; whilst the activity of current derivatives against Topo IV is lower, it could be arguably improved with future modifications. The emergence of knock-out mutants of the nucleoside transporter Tsx, which is hijacked by albicidins to enter into Gram-negative bacteria, is somewhat alarming. However, we do not assess this as a significant liability because the *tsx* mutations provide only limited resistance^62^.

For successful clinical application of albicidins, their properties such as solubility, plasma stability and plasma-protein binding can be improved while toxicity towards eukaryotic topoisomerase II should be monitored. Our work now enables a rational structure-based design which preserves albicidin’s distinct and potent inhibition mechanism.

## Methods

### Cloning

Plasmids pET28b-EcGyrATWS and pET28b-EcGyrBTWS encoding 10-His- and Twin-Strep tagged *E. coli* gyrase A and B subunits^40^ were gifts from V. Lamour (IGBMC and Strasbourg University Hospitals). *E. coli* GyrA and GyrB point mutations were made using the QuikChange (Stratagene) method and a variation thereof involving amplification of two halves of the plasmid and ligation using the NEBuilder HiFi assembly kit^63,64^. Briefly, forward or reverse primers from the ColE1 site of *E. coli* were used in two separate polymerase chain reaction (PCR) reactions with forward or reverse primers from the opposite side of the plasmid at the point of mutagenesis to create two plasmid fragments. The two separate PCR fragments were ligated using NEBuilder HiFi DNA Assembly Master Mix. Primers were synthesized by Merck and are described in Supplementary Table 1. Mutations were verified by Sanger sequencing (Eurofins).

A pUC plasmid containing eight repeats of phage Mu 217 bp SGS (pUC-8xMuSGS) was constructed as previously described^65^ using NEB Stable cells for cloning. Mu217 SGS was PCR amplified from plasmid pMP1000^41^ using primers Mu217_HindIIIBglIIEcoRV_for and Mu217_BamHIEcoRV_rev; the sequence of Mu SGS is as previously reported^42^ and the full sequence of the 217 bp fragment is given in Supplementary Table 2. All plasmids are listed in Supplementary Table 2.

### MIC determination

MIC values were determined according to the ninth edition of the Approved Standard M07-A9. The test was carried out for six different bacterial strains (*E. coli* DSM 1116 (Gram-negative), *E. coli* BW25113 (Gram-negative), *B. subtilis* DSM 10 (Gram-positive), *M. luteus* DSM 1790 (Gram-positive), *M. phlei* DSM 750 (Gram-positive), *S. typhimurium* TA 100 (Gram-negative). First, 20 µl of glycerol stock of each strain was inoculated in 20 ml LB (Lennox) followed by incubation overnight at 37 °C, 200 r.p.m. The test inoculum was adjusted by the 0.5 McFarland Standard. Within 15 min of preparation, the adjusted inoculum suspension was diluted in MHBII so that each well contained approximately 5 × 10^5^ c.f.u. ml^−1^ in a final volume of 100 µl. Then, 95 µl of the inoculum was applied per well and 5 µl of the (diluted) albicidins was added. For dilutions, the dry powders were dissolved in dimethylsulfoxide (DMSO) with a concentration of 2.56 mg ml^−1^, and the obtained stock solutions were further diluted in DMSO. Next, 5 µl of each antibiotic dilution was applied to the microdilution tray to reach final concentrations of 8 to 0.016 µg ml^−1^. One row of each well plate served as a growth control without antibiotic and another row of the microdilution tray served as a sterility control (only MHBII media). The antimicrobial effect of the solvent was tested by adding 5 µl DMSO to several wells. Purity check and cell titre control were performed according to International Standard M07-A9 on Mueller–Hinton II Agar. Both microdilution trays and agar plates were incubated at 37 °C for 20 h.

### *E. coli* DNA gyrase expression and purification

Plasmids pET28b-EcGyrATWS and pET28b-EcGyrBTWS were transformed into BL21(DE3) Star (Thermo) competent cells. 2xYT media (0.5 litres for GyrA and 1 litre for GyrB) was supplemented with 30 µg ml^−1^ kanamycin and inoculated with overnight culture in a 1:100 ratio. Cultures were grown to an optical density (OD_600_) of 0.6 at 37 °C with shaking (200 r.p.m.) before induction of protein expression with 0.5 mM isopropylthiogalactoside. The temperature was reduced to 20 °C and the cultures were incubated overnight with shaking.

Cells were collected at 6,500*g* at 4 °C for 15 min. Pellets were resuspended in HisTrap lysis buffer (50 mM Tris–Cl pH 7.5, 300 mM NaCl, 10% glycerol, 20 mM imidazole), 1 mg ml^−1^ lysozyme, 50 µg ml^−1^ DNase I and cOmplete EDTA-free Protease Inhibitor Cocktail (Roche) and incubated on ice for 30 min. Cells were lysed by sonication, controlling the temperature to below 10 °C throughout. The resulting lysate was clarified at 50,000*g* for 30 min at 4 °C.

Clarified lysate was manually loaded onto a pre-equilibrated 5 ml HisTrap FF Crude (Cytiva) column. The column was washed with 10 column volumes of ice-cold HisTrap lysis buffer. Protein was eluted with ice-cold HisTrap elution buffer (50 mM Tris–Cl pH 7.5, 300 mM NaCl, 10% glycerol, 250 mM imidazole). Protein fractions were pooled and loaded manually onto a 1 ml StrepTrap HP (Cytiva) column pre-equilibrated with ice-cold StrepTrap buffer (20 mM Na-HEPES pH 8, 60 mM NaCl, 10% glycerol, 1 mM EDTA pH 8, 1 mM dithiothreitol (DTT)). The column was washed with 15 column volumes of ice-cold StrepTrap buffer and protein was eluted with ice-cold StrepTrap Elution buffer (20 mM Na-HEPES pH 8, 60 mM NaCl, 10% glycerol, 1 mM EDTA, 1 mM DTT, 3 mM desthiobiotin). His- and Strep-tags were cleaved overnight at 4 °C using TEV and 3C proteases in a 1:100 protease:protein ratio. After cleavage, tag-free protein was loaded onto a 5 ml HiTrap Q HP (Cytiva) column pre-equilibrated with HGED buffer (50 mM Na-HEPES pH 8, 10% glycerol, 1 mM EDTA, 2 mM DTT). The column was washed extensively with HGED, washed by steps of increasing NaCl concentrations and eluted using a gradient of NaCl. Chromatography was carried out at 4 °C. Pure fractions were buffer exchanged to HGED, concentrated using Amicon ultrafiltration (Merck), snap-frozen in liquid nitrogen and stored at −80 °C. Protein purity can be assessed from an SDS–PAGE gel (Supplementary Fig. 10).

### Topoisomerase assays

For DNA gyrase supercoiling assays to characterize gyrase mutants, 15 µl reactions were set up containing 250 ng of relaxed DNA, *E. coli* gyrase supercoiling buffer (35 mM Tris–Cl pH 7.5, 24 mM KCl, 4 mM MgCl_2_, 2 mM DTT, 1.8 mM spermidine, 1 mM ATP, 6.0% (w/v) glycerol, and 0.1 mg ml^−1^ BSA), gyrase subunits and inhibitors. Concentrations of enzyme and inhibitor are stated in the figure legends. Inhibitors were diluted in the following way to reduce the chance of precipitation: stock compound in 100% DMSO was diluted to 5 mM in 100% DMSO. This compound solution was diluted to 1 mM by stepwise addition of 1 µl of 50% DMSO with thorough mixing via pipetting after each addition. All subsequent dilutions up to 100 µM were performed in the same way using H_2_O. After this point, compounds were stable and could be diluted in the normal way. Reactions were incubated at 37 °C for 30 min before stopping with 15 µl chloroform:isoamyl alcohol (24:1 v/v) and 15 µl of STEB (40% (w/v) sucrose, 100 mM Tris–Cl pH 8, 10 mM EDTA, 0.5 mg ml^−1^ bromophenol blue).

Cleavage assays were set up in the same way as supercoiling assays with some modifications. The concentration of enzyme was increased 4-fold relative to the supercoiling assay and reactions were incubated for 1 h before addition of 0.2% SDS and proteinase K (0.2 mg ml^−1^) and further incubation at 37 °C for 30 min to trap cleavage complexes. Reactions were stopped in the same way as supercoiling assays.

*E. coli* Topo IV relaxation assays were set up in the same way as supercoiling assays with some modifications. The substrate was negatively supercoiled DNA, *E. coli* Topo IV relaxation buffer (50 mM K-HEPES pH 7.6, 100 mM potassium glutamate, 10 mM magnesium acetate, 10 mM DTT, 1 mM ATP and 0.05 mg ml^−1^ BSA) and reactions were incubated for 1 h. Reactions were stopped in the same way as supercoiling assays.

Topo IV cleavage assays were set up in the same way as relaxation assays with some modifications. The concentration of enzyme was increased by 20% and cleavage complexes were trapped by the addition of 0.2% SDS and proteinase K (0.2 mg ml^−1^) and further incubation at 37 °C for 30 min. Reactions were stopped in the same way as supercoiling assays.

Results were visualized by agarose gel electrophoresis: 1% agarose Tris-acetate-EDTA (TAE) gels were run without ethidium bromide (supercoiling) or with 10 µg ml^−1^ ethidium bromide (cleavage) at 8 V cm^−1^.

For initial compound IC_50_ determination, DNA supercoiling experiments with DNA gyrase were performed in a total volume of 30 μl of gyrase buffer (Inspiralis). The reactions contained 500 ng relaxed pBR322 plasmid DNA, 1.5 U DNA gyrase (6 U μl^−1^) and albicidin derivatives at a final concentration of between 2.5 and 320 nM. The final DMSO concentration used was 3%. Samples were incubated at 37 °C for 30 min and subsequently loaded on an agarose gel. Electrophoretic analysis was performed using a 1% agarose gel (100 V, 90 min). DNA bands were stained with ethidium bromide and subsequently quantified using ImageJ 1.53k software.

### Photocrosslinking

Mu217 DNA (25 nM) was incubated with 0.4 µM gyrase and indicated concentrations (1 or 10 µM) of diazirine-labelled albicidin (photo-Albi, φAlbi) in a volume of 20 µl (~70 ng DNA) at 37 °C for 30 min in gyrase cleavage buffer (see section 'Topoisomerase assays'). For CFX competition experiments, 30 µM CFX was added first, followed by the albicidin. Reactions were either irradiated with ultraviolet 365 nm (benchtop transilluminator) with cooling (ice) for 30 min or left on ice for 30 min (‘dark’). Then, 0.2% SDS and 0.2 mg ml^−1^ proteinase K were added to both samples, which were incubated for another 30 min. Reactions were mixed with loading dye (STEB) and chloroform:isoamyl alcohol (24:1 v/v) before vortexing and centrifugation. The aqueous phase was loaded on 4–20% TBE (Tris–borate–EDTA) polyacrylamide gel (Thermo) and ran for 1 h at 150 V. DNA was visualized with SYBR Gold (Thermo) staining.

### CC_50_ data analysis

DNA cleavage gels were analysed using densitometry (ImageJ). Experiments were carried out in triplicate and expressed as mean ± s.d. Curves were fitted using OriginPro 2021 (Origin Lab) according to a modified Hill equation:

1 $$y = {\mathrm{cI}} + ({\mathrm{cF}} - {\mathrm{cI}})/\left( {x^n/(k^n + x^n)} \right.$$

where *y* is the percentage of cleaved DNA, cI is the initial cleavage, cF is the final cleavage, *k* is the CC_50_ (the concentration of inhibitor that results in cleavage of 50% of DNA), *n* is a slope factor and *x* is the inhibitor concentration.

### Preparation of 217 bp DNA fragment

For DNA preparation, NEB Stable *E. coli* cells were transformed with pUC-8xMuSGS. LB media (2 l) was supplemented with 100 µg ml^−1^ ampicillin and inoculated with overnight culture in a 1:100 ratio. Cultures were grown at 37 °C overnight with shaking (200 r.p.m.). Cells were collected at 6,500*g* at 4 °C for 15 min and plasmid was purified using the PureLink HiPure Expi Plasmid Gigaprep Kit (Thermo) following the manufacturer’s instructions. Final purified plasmid was dissolved in mQ H_2_O to a final concentration of 1–2 mg ml^−1^. Plasmid was digested using EcoRV (Thermo) (500 U mg^−1^ DNA) overnight at 37 °C in buffer R. The 217 bp fragment was separated from the digested plasmid backbone using size-exclusion chromatography (Sephacryl S-500 16/60, Cytiva) in DNA SEC buffer (20 mM Na-HEPES pH 8, 120 mM KCl). DNA fragments containing fractions were pooled and concentrated via isopropanol precipitation to a final concentration of ~30 µM in TE buffer.

### Cryo-EM sample preparation

*E. coli* GyrA and GyrB subunits were mixed in equimolar proportions in the storage buffer to reconstitute the full DNA gyrase enzyme. Then, 217 bp DNA was added to the complex in a 1:1 ratio to a final gyrase–DNA complex concentration of ~15 μM. The reconstituted complex was buffer exchanged using dialysis at 4 °C overnight to cryo-EM buffer (25 mM Na-HEPES pH 8, 30 mM potassium acetate, 2.5 mM magnesium acetate, 0.5 mM Tris (2-carboxyethyl) phosphine (TCEP)). After buffer exchange, the sample was concentrated to ~30 µM. The sample was supplemented with 100 µM compound, 1 mM ADPNP and incubated for 15 min at 37 °C. CHAPSO (8 mM) was added, and the sample was centrifuged (60 min at 21,000*g*) to remove potential aggregates.

### Cryo-EM data collection and analysis

Aliquots of 4 μl of reconstituted complexes (~12 mg ml^−1^) were applied to freshly glow-discharged (Leica, 60 s/8 mA) Quantifoil holey carbon grids (R2/1, 300 copper mesh). After 30 s of incubation with 95% chamber humidity at 10 °C, the grids were blotted for 6 s and plunge-frozen in liquid ethane using a Vitrobot Mark IV (Thermo).

Cryo-EM data were collected at the Polish National cryo-EM facility SOLARIS on a Titan Krios G3i microscope (Thermo) operated at 300 kV. For the main **Gyr–Mu217–albicidin** dataset, images were collected at a 105,000× nominal magnification, resulting in a calibrated physical pixel size of 0.86 Å per pixel, with a range of defoci set as −2.1, −2.1, −1.8, −1.5, −1.2, −0.9 μm using EPU v.2.10.0.1941REL. Movies were recorded in counting mode on a K3 camera (Gatan) equipped with Gatan BioQuantum energy filter operated with a slit width of 20 eV. Total dose on vacuum was 15.28 electrons per pixel per second and the exposure time was set to generate a total dose of ∼39.08 electrons per Å^2^ over 40 movie frames. Movies were saved using physical pixel size as gain-corrected TIFF files. Statistics for cryo-EM data collection for this and other datasets are listed in Table 2.

**Table 2:** Data collection statistics

Dataset	EcGyr–Mu217–albicidin	EcGyr–Mu217-Albi-1	EcGyr–Mu217–Albi-2
Microscope	Titan Krios G3i	Titan Krios G3i	Titan Krios G3i
Magnification	105,000×	105,000×	105,000×
Voltage (kV)	300	300	300
Electron dose (*e*^−^ Å^−^^2^)	39.08	41.57	39.59
Detector	K3	K3	K3
Defocus range (−µm)	2.1–0.9	3–0.9	2.4–0.9
Pixel size (Å)	0.86	0.86	0.86
Symmetry imposed	*C*1	*C*1	*C*1
Micrographs (no.)	14,469	8,136	11,579
Initial particle images (no.)	2,040,353	1,366,620	1,222,762
Final particle images (no.)	147,019 (core)	37,902 (TG)	49,921
	23,435 (insert)	25 663 (AA)	
		22 162 (XT)	
Global map resolution (Å)	2.6 (core)	3.25 (TG)	3.25
		3.30 (AA)	
	3.06 (insert)	3.41 (XT)	
Fourier shell correlation (FSC) threshold	0.143	0.143	0.143

For the **Gyr–Mu217–albicidin** dataset, all processing was done in cryoSPARC 3+^66^. In total, 14,469 movies were dose weighted and motion and contrast transfer function (CTF) corrected in patch mode. The resulting micrographs were manually curated and 14,121 were selected for further analysis. Particles were picked with the cryoSPARC template picker and 2 × 2 binned particles (2,040,353) were subjected to several rounds of 2D classification. Cleaned particles (314,230) underwent one round of 3D classification (CS3 ab initio, 254,727 particles retained) and were re-extracted as unbinned particles using updated coordinates. Non-uniform refinement^67^ of this particle set with local CTF correction^68^ resulted in 2.72 Å global resolution (the resulting map captures the cleavage–reunion core of the enzyme with other parts much more flexible). To further clean the particle stack, it was subjected to several rounds of heterogenous refinement in cryoSPARC where a low-pass filtered model was used as a sink for lower-quality particles^69^. This approach led to a further increase in resolution up to 2.5 Å (with Ewald sphere correction and anisotropic magnification correction) from 147,019 particles. Local resolution in the vicinity of the drug-binding site reached 2.5 Å.

The resulting map suffered from disorder in the region corresponding to the GyrB TOPRIM insert domain, similarly to what was previously reported^40^. To obtain a better insert map, further 3D classification (CS 3D classification without alignment) was carried out with a mask around one of the insert domains that was better resolved. After multiple rounds of classification, a subset of 23,435 particles was obtained that refined to 3.06 Å resolution and allowed us to build a complete chain for GyrB, including all looped regions. Both maps (2.6 Å and 3.0 Å) were used in the refinement.

For **Gyr–Mu217–Albi-1**, 8,136 movies were collected and 7,980 selected for further processing. In total, 1,366,620 particles were picked using a template picker, extracted (2 × 2 binned) and subjected to two rounds of 2D classification to yield 211,603 cleaned particles. The ab initio holocomplex structure was modelled using 176,713 particles and the rest were discarded as junk. After refinement, unbinned particles were re-extracted using updated coordinates and refined to 3.34 Å. This particle stack was further cleaned up in one round of classification without alignment using automasking and two classes. Approximately half of the particles of lower quality were removed and the resulting particles were refined to 3.05 Å. Inspection of the active site density and unsupervised masked 3D classification analysis revealed that the map represents a mixture of three different binding modes (named TG, AA and XT in the main text). To separate these classes, **Albi-1** was modelled into the density to occupy all three pockets and a mask was created based on this model. Input volumes were then created, based on each individual position of **Albi-1** possible. Focused 3D classification using this mask (3D classification beta, not to be confused with Heterogenous Refinement) in cryoSPARC was then carried out to separate particles between these three structures based on the density within the mask. Each of the resulting classes was independently refined to high resolution (TG, 3.25 Å, 37,902 particles; AA, 3.25 Å, 25,663 particles; XT, 3.30 Å, 22,162 particles) and represented a unique way of compound binding.

For the **Gyr–Mu217–Albi-2**, 11,579 movies were collected. After manual curation, 8,388 were chosen for further processing. In total, 1,222,762 particles were picked using a template picker, and binned particles were extracted and cleaned in two rounds of 2D classification. The 251,514 particles that were retained were cleaned in one round of 3D classification (ab initio) and re-extracted after consensus refinement. Unbinned particles were further cleaned up first in another round of ab initio (two classes) and subsequently by 3D classification without alignment (two classes). This resulted in a 3.19 Å consensus map (non-uniform refinement with local CTF correction) from 78,757 particles. To remove contaminating particles from alternative positions of the compound, another round of 3D classification without alignment was carried out, yielding the final 3.25 Å map from 49,921 particles that was used for model refinement.

### Model building and refinement

The initial model (based on the **Gyr–Mu217–albicidin** map) was built manually in Coot^70^ using the closest available structure (PDB: 6RKV) as a starting point. Large domains were rigid-body fitted in ChimeraX^71^ and connecting elements were rebuilt if necessary; the insert domain of GyrB was rebuilt de novo in Coot. Symmetry-related chains were built using symmetry operation and refined without symmetry in real space using Phenix.refine^72^ (Ramachandran restrains and secondary structure restraints). Local geometry, particularly in poorly resolved or looped regions, was optimized using ISOLDE^73^ followed by the further rounds of refinement in Phenix with explicit hydrogens. To model the postulated Mg^2+^ ion, ideal geometric restraints were used to build and refine an octahedral water shell around the centre of the observed density blob^74^. The density at this resolution did not allow an unambiguous assignment of water molecules. The albicidin model was publicly available under PDB ligand code BWH; restraints were obtained using Grade server (http://grade.globalphasing.org). The model was refined first against the 2.6 Å map and subsequently against the 3.06 Å map. MolProbity^75^ was used to validate the structures. Statistics for the final model (refined against the 3.06 Å map at 3 Å) are reported in Table 3. For **Albi-1** (TG, AA and XT), the restraints were obtained using Grade server and models were refined in Phenix using the albicidin structure as a starting model. For **Albi-2**, the restraints were obtained using Grade server and the model was similarly refined in real space in Phenix against the 3.25 Å map; final statistics are reported in Table 3.

**Table 3:** Refinement statistics

Model	EcGyr–Mu217–albicidin	EcGyr–Mu217–Albi-1-TG	EcGyr–Mu217–Albi-1-AA	EcGyr–Mu217–Albi-2
Model resolution (Å)	3.06 (3.47)	3.25 (3.77)	3.31 (0.143)	3.24 (3.69)
FSC threshold	0.143 (0.5)		3.93 (0.5)	0.143 (0.5)
Map sharpening *B* factor (Å^2^)	37.2	65.1	56.4	68.1
Model composition				
Non-hydrogen atoms	15,966	15,966	15,966	15,972
Protein residues	1,838	1,838	1,838	1,838
Nucleotides	64	64	64	64
Ligands	3	3	3	3
B factors (Å^2^)				
Protein	55.4	74.3	100.7	40.6
Nucleotide	73.9	96.8	118.2	63.1
Ligands	45.5	48.8	61.3	29.9
R.M.S. deviations				
Bond lengths (Å)	0.003	0.003	0.003	0.003
Bond angles (°)	0.577	0.568	0.601	0.559
Validation				
MolProbity score	1.31	1.28	1.33	1.24
Clashscore	1.59	1.79	1.79	1.56
Ramachandran plot				
Favoured (%)	93.97	95.07	94.13	95.18
Allowed (%)	6.03	4.93	5.87	4.82
Disallowed (%)	0.00	0.00	0.00	0.00

### Reporting summary

Further information on research design is available in the Nature Portfolio Reporting Summary linked to this article.

## Supplementary information

Supplementary Information Supplementary Tables 1–4, Figs. 1–11, Note 1 and Methods.

Reporting Summary

Supplementary Video 1 A ChimeraX movie demonstrating conformational transition between ‘pre-cleavage*’ E. coli* gyrase conformation (PDB: 6RKV) and albicidin-bound conformation (PDB: 7Z9C).

## Data Availability

All data needed to evaluate the conclusions in the paper are present in the paper and/or the Supplementary Materials or from the authors upon reasonable request. The **Gyr-Mu217-Albi**, **Gyr-Mu217-Albi-1-TG**, **Gyr-Mu217-Albi-1-AA** and **Gyr-Mu217-Albi-2** coordinates have been submitted to the Protein Data Bank (https://www.rcsb.org/) with PDB IDs 7Z9C, 7Z9K, 7Z9M and 7Z9G, respectively. Corresponding EM maps have been submitted to the Electron Microscopy Data Bank (https://www.ebi.ac.uk/pdbe/emdb/) with IDs EMD-14570, EMD-14573, EMD-14574, and EMD-14572, respectively. Raw data were submitted to the Electron Microscopy Public Image Archive (https://www.ebi.ac.uk/pdbe/emdb/empiar/) with IDs EMPIAR-11244 (albicidin), EMPIAR-11245 (Albi-1) and EMPIAR-11246 (Albi-2). Source data are provided with this paper.

## Source data

Source Data Fig. 4 Uncropped gels and calculations used to determine CC_50_ values for Fig. 4e (single replicates).

## References

[1] Antimicrobial Resistance Collaborators. (2022). Global burden of bacterial antimicrobial resistance in 2019: a systematic analysis. Lancet.

[2] Rawson TM, Ming D, Ahmad R, Moore LSP, Holmes AH (2020). Antimicrobial use, drug-resistant infections and COVID-19. Nat. Rev. Microbiol..

[3] Nowak J (2017). High incidence of pandrug-resistant *Acinetobacter baumannii* isolates collected from patients with ventilator-associated pneumonia in Greece, Italy and Spain as part of the MagicBullet clinical trial. J. Antimicrob. Chemother..

[4] Lewis K (2020). The science of antibiotic discovery. Cell.

[5] Aldred KJ, Kerns RJ, Osheroff N (2014). Mechanism of quinolone action and resistance. Biochemistry.

[6] Bates AD, Berger JM, Maxwell A (2011). The ancestral role of ATP hydrolysis in type II topoisomerases: prevention of DNA double-strand breaks. Nucleic Acids Res..

[7] Wohlkonig A (2010). Structural basis of quinolone inhibition of type IIA topoisomerases and target-mediated resistance. Nat. Struct. Mol. Biol..

[8] Blower TR, Williamson BH, Kerns RJ, Berger JM (2016). Crystal structure and stability of gyrase-fluoroquinolone cleaved complexes from *Mycobacterium tuberculosis*. Proc. Natl Acad. Sci. USA.

[9] Bush NG, Diez-Santos I, Abbott LR, Maxwell A (2020). Quinolones: mechanism, lethality and their contributions to antibiotic resistance. Molecules.

[10] Kalghatgi S (2013). Bactericidal antibiotics induce mitochondrial dysfunction and oxidative damage in mammalian cells. Sci. Transl. Med..

[11] Sankar A (2021). Association of fluoroquinolone prescribing rates with black box warnings from the US Food and Drug Administration. JAMA Netw. Open.

[12] Scangarella-Oman NE (2022). Dose selection for phase III clinical evaluation of gepotidacin (GSK2140944) in the treatment of uncomplicated urinary tract infections. Antimicrob. Agents Chemother..

[13] Taylor SN (2018). Gepotidacin for the treatment of uncomplicated urogenital gonorrhea: A phase 2, randomized, dose-ranging, single-oral dose evaluation. Clin. Infect. Dis..

[14] Bradford PA, Miller AA, O’Donnell J, Mueller JP (2020). Zoliflodacin: an oral spiropyrimidinetrione antibiotic for the treatment of *Neisseria gonorrheae*, including multi-drug-resistant isolates. ACS Infect. Dis..

[15] Hossain, M., Zhou, M., Tiffany, C., Dumont, E. & Darpo, B. A phase I, randomized, double-blinded, placebo- and moxifloxacin-controlled, four-period crossover study to evaluate the effect of gepotidacin on cardiac conduction as assessed by 12-lead electrocardiogram in healthy volunteers. *Antimicrob. Agents Chemother*. **61**, e02385-16 (2017). 10.1128/AAC.02385-16 PMC5404516 28223381

[16] Hashimi SM (2019). Albicidin, a potent DNA gyrase inhibitor with clinical potential. J. Antibiot. (Tokyo).

[17] Birch RG, Patil SS (1987). Evidence that an albicidin-like phytotoxin induces chlorosis in sugarcane leaf scald disease by blocking plastid DNA replication. Physiol. Mol. Plant.

[18] Wall MK, Mitchenall LA, Maxwell A (2004). *Arabidopsis thaliana* DNA gyrase is targeted to chloroplasts and mitochondria. Proc. Natl Acad. Sci. USA.

[19] Evans-Roberts KM (2016). DNA gyrase is the target for the quinolone drug ciprofloxacin in *Arabidopsis thaliana*. J. Biol. Chem..

[20] Hashimi SM, Wall MK, Smith AB, Maxwell A, Birch RG (2007). The phytotoxin albicidin is a novel inhibitor of DNA gyrase. Antimicrob. Agents Chemother..

[21] Cociancich S (2015). The gyrase inhibitor albicidin consists of *p*-aminobenzoic acids and cyanoalanine. Nat. Chem. Biol..

[22] Kretz J (2015). Total synthesis of albicidin: a lead structure from *Xanthomonas albilineans* for potent antibacterial gyrase inhibitors. Angew. Chem. Int. Ed. Engl..

[23] Grätz S (2016). Synthesis and antimicrobial activity of albicidin derivatives with variations of the central cyanoalanine building block. ChemMedChem.

[24] Kerwat D (2016). Synthesis of albicidin derivatives: assessing the role of N-terminal acylation on the antibacterial activity. ChemMedChem.

[25] Behroz I (2019). Extensive structure–activity relationship study of albicidin’s C-terminal dipeptidic *p*-aminobenzoic acid moiety. Eur. J. Chem..

[26] Zborovsky L (2021). Improvement of the antimicrobial potency, pharmacokinetic and pharmacodynamic properties of albicidin by incorporation of nitrogen atoms. Chem. Sci..

[27] Rostock L (2018). Molecular insights into antibiotic resistance—how a binding protein traps albicidin. Nat. Commun..

[28] Zhang L, Xu J, Birch RG (1998). High affinity binding of albicidin phytotoxins by the AlbA protein from *Klebsiella oxytoca*. Microbiology (Reading).

[29] Sikandar A (2018). Adaptation of a bacterial multidrug resistance system revealed by the structure and function of AlbA. J. Am. Chem. Soc..

[30] Zhang L, Birch RG (1997). Mechanisms of biocontrol by *Pantoea dispersa* of sugar cane leaf scald disease caused by *Xanthomonas albilineans*. J. Appl. Microbiol..

[31] Kleebauer L (2021). Overcoming AlbD protease resistance and improving potency: synthesis and bioactivity of antibacterial albicidin analogues with amide bond isosteres. Org. Lett..

[32] Vieweg L (2015). The albicidin resistance factor AlbD is a serine endopeptidase that hydrolyzes unusual oligoaromatic-type peptides. J. Am. Chem. Soc..

[33] Vetting MW, Hegde SS, Zhang Y, Blanchard JS (2011). Pentapeptide-repeat proteins that act as topoisomerase poison resistance factors have a common dimer interface. Acta Crystallogr. F.

[34] Mazurek Ł (2021). Pentapeptide repeat protein QnrB1 requires ATP hydrolysis to rejuvenate poisoned gyrase complexes. Nucleic Acids Res..

[35] Baumann S (2014). Cystobactamids: myxobacterial topoisomerase inhibitors exhibiting potent antibacterial activity. Angew. Chem. Int. Ed. Engl..

[36] Kampranis SC, Maxwell A (1996). Conversion of DNA gyrase into a conventional type II topoisomerase. Proc. Natl Acad. Sci. USA.

[37] Srikannathasan V (2015). Crystallization and initial crystallographic analysis of covalent DNA-cleavage complexes of *Staphyloccocus aureus* DNA gyrase with QPT-1, moxifloxacin and etoposide. Acta Crystallogr. F.

[38] Heddle JG (2001). The antibiotic microcin B17 is a DNA gyrase poison: characterisation of the mode of inhibition. J. Mol. Biol..

[39] Pierrat OA, Maxwell A (2005). Evidence for the role of DNA strand passage in the mechanism of action of microcin B17 on DNA gyrase. Biochemistry.

[40] Vanden Broeck A, Lotz C, Ortiz J, Lamour V (2019). Cryo-EM structure of the complete *E. coli* DNA gyrase nucleoprotein complex. Nat. Commun..

[41] Scheirer KE, Higgins NP (1997). The DNA cleavage reaction of DNA gyrase. Comparison of stable ternary complexes formed with enoxacin and CcdB protein. J. Biol. Chem..

[42] Sutormin D, Rubanova N, Logacheva M, Ghilarov D, Severinov K (2019). Single-nucleotide-resolution mapping of DNA gyrase cleavage sites across the *Escherichia coli* genome. Nucleic Acids Res..

[43] Bax BD, Murshudov G, Maxwell A, Germe T (2019). DNA topoisomerase inhibitors: trapping a DNA-cleaving machine in motion. J. Mol. Biol..

[44] Behroz I (2021). Acetylenic replacement of albicidin’s methacrylamide residue circumvents detrimental *E*/*Z* photoisomerization and preserves antibacterial activity. Eur. J. Chem..

[45] Birch RG, Patil SS (1985). Preliminary characterization of an antibiotic produced by *Xanthomonas albilineans* which inhibits DNA synthesis in *Escherichia coli*. J. Gen. Microbiol..

[46] Chen SF (2018). Structural insights into the gating of DNA passage by the topoisomerase II DNA-gate. Nat. Commun..

[47] Soczek KM, Grant T, Rosenthal PB, Mondragón A (2018). CryoEM structures of open dimers of gyrase A in complex with DNA illuminate mechanism of strand passage. eLife.

[48] Schmidt BH, Burgin AB, Deweese JE, Osheroff N, Berger JM (2010). A novel and unified two-metal mechanism for DNA cleavage by type II and IA topoisomerases. Nature.

[49] Sartorius J, Schneider HJ (1997). Supramolecular chemistry. 71. Intercalation mechanisms with ds-DNA: binding modes and energy contributions with benzene, naphthalene, quinoline and indole derivatives including some antimalarials. J. Chem. Soc. Perkin.

[50] Banks TM, Clay SF, Glover SA, Schumacher RR (2016). Mutagenicity of *N*-acyloxy-*N*-alkoxyamides as an indicator of DNA intercalation part 1: evidence for naphthalene as a DNA intercalator. Org. Biomol. Chem..

[51] Birch RG, Pemberton JM, Basnayake WVS (1990). Stable albicidin resistance in *Escherichia coli* involves an altered outer-membrane nucleoside uptake system. Microbiology.

[52] Ashkenazy H (2016). ConSurf 2016: an improved methodology to estimate and visualize evolutionary conservation in macromolecules. Nucleic Acids Res..

[53] Germe T (2018). A new class of antibacterials, the imidazopyrazinones, reveal structural transitions involved in DNA gyrase poisoning and mechanisms of resistance. Nucleic Acids Res..

[54] Chenia HY, Pillay B, Pillay D (2006). Analysis of the mechanisms of fluoroquinolone resistance in urinary tract pathogens. J. Antimicrob. Chemother..

[55] Marcusson LL, Frimodt-Møller N, Hughes D (2009). Interplay in the selection of fluoroquinolone resistance and bacterial fitness. PLoS Pathog..

[56] Heddle J, Maxwell A (2002). Quinolone-binding pocket of DNA gyrase: role of GyrB. Antimicrob. Agents Chemother..

[57] Collin F, Karkare S, Maxwell A (2011). Exploiting bacterial DNA gyrase as a drug target: current state and perspectives. Appl. Microbiol. Biot..

[58] Feng L (2021). The pentapeptide-repeat protein, MfpA, interacts with mycobacterial DNA gyrase as a DNA T-segment mimic. Proc. Natl Acad. Sci. USA.

[59] Shah S, Heddle JG (2014). Squaring up to DNA: pentapeptide repeat proteins and DNA mimicry. Appl. Microbiol. Biotechnol..

[60] Panter F, Krug D, Baumann S, Müller R (2018). Self-resistance guided genome mining uncovers new topoisomerase inhibitors from myxobacteria. Chem. Sci..

[61] Garrido MC, Herrero M, Kolter R, Moreno F (1988). The export of the DNA replication inhibitor Microcin B17 provides immunity for the host cell. EMBO J..

[62] Fsihi H, Kottwitz B, Bremer E (1993). Single amino acid substitutions affecting the substrate specificity of the *Escherichia coli* K-12 nucleoside-specific Tsx channel. J. Biol. Chem..

[63] Heydenreich FM (2017). High-throughput mutagenesis using a two-fragment PCR approach. Sci. Rep..

[64] Gibson DG (2009). Enzymatic assembly of DNA molecules up to several hundred kilobases. Nat. Methods.

[65] Dyer PN (2004). Reconstitution of nucleosome core particles from recombinant histones and DNA. Methods Enzymol..

[66] Punjani A, Rubinstein JL, Fleet DJ, Brubaker MA (2017). cryoSPARC: algorithms for rapid unsupervised cryo-EM structure determination. Nat. Methods.

[67] Punjani A, Zhang H, Fleet DJ (2020). Non-uniform refinement: adaptive regularization improves single-particle cryo-EM reconstruction. Nat. Methods.

[68] Zivanov J, Nakane T, Scheres SHW (2020). Estimation of high-order aberrations and anisotropic magnification from cryo-EM data sets in RELION-3.1. IUCrJ.

[69] Gong X (2016). Structural insights into the Niemann–Pick C1 (NPC1)-mediated cholesterol transfer and ebola infection. Cell.

[70] Emsley P, Lohkamp B, Scott WG, Cowtan K (2010). Features and development of Coot. Acta Crystallogr. D.

[71] Pettersen EF (2021). UCSF ChimeraX: Structure visualization for researchers, educators, and developers. Protein Sci..

[72] Liebschner D (2019). Macromolecular structure determination using X-rays, neutrons and electrons: recent developments in Phenix. Acta Crystallogr. D.

[73] Croll TI (2018). ISOLDE: a physically realistic environment for model building into low-resolution electron-density maps. Acta Crystallogr. D.

[74] Katz AK, Glusker JP, Beebe SA, Bock CW (1996). Calcium ion coordination: a comparison with that of beryllium, magnesium, and zinc. J. Am. Chem. Soc..

[75] Williams CJ (2018). MolProbity: more and better reference data for improved all-atom structure validation. Protein Sci..

[76] Laskowski RA, Swindells MB (2011). LigPlot+: multiple ligand-protein interaction diagrams for drug discovery. J. Chem. Inf. Model..

