# Supplementary material for: Molecular mechanism of topoisomerase poisoning by the peptide antibiotic albicidin
Source: Nat Catal. 2023 Jan 23;6(1):52–67. doi: 10.1038/s41929-022-00904-1 (PMC9886550; doi:10.1038/s41929-022-00904-1)
Supplement: Supplementary file 1 — Supplementary Tables 1–4, Figs. 1–11, Note 1 and Methods. [file 41929_2022_904_MOESM1_ESM.pdf]

nature catalysis

Article https://doi.org/10.1038/s41929-022-00904-1

Molecular mechanism of topoisomerase poisoning by the peptide antibiotic albicidin

In the format provided by the authors and unedited

SUPPLEMENTARY INFORMATION

Molecular mechanism of topoisomerase poisoning by the peptide antibiotic albicidin

Elizabeth Michalczyk[†1], Kay Hommernick[†2], Iraj Behroz[2], Marcel Kulike[2], Zuzanna Pakosz-Stępień[1,3], Lukasz Mazurek[1,3], Maria Seidel[2], Maria Kunert[2], Karine Santos[4,5], Holger von Moeller[4,5], Bernhard Loll[4,5], John B. Weston[2], Andi Mainz[2], Jonathan G. Heddle[1*], Roderich D. Süssmuth[2*] and Dmitry Ghilarov[1,6*]

[1] Malopolska Centre of Biotechnology, Jagiellonian University, 30-387 Krakow, Poland

[2]Institut für Chemie, Technische Universität Berlin, Straße des 17. Juni 124, 10623 Berlin, Germany

[3]Postgraduate School of Molecular Medicine, Warsaw, Poland

[4]moloX GmbH, Takustr. 6, 14195 Berlin, Germany

[5]Institut für Chemie und Biochemie, Freie Universität Berlin, Takustr. 6, 14195 Berlin, Germany

[6]John Innes Centre, Norwich Research Park, Norwich NR4 7UH, UK

[†]These authors have contributed equally to this work.

*Correspondence should be addressed to:

dmitry.ghilarov@jic.ac.uk

jonathan.heddle@uj.edu.pl

roderich.suessmuth@tu-berlin.de

TABLE OF CONTENTS

Supplementary Tables

**Supplementary Table 1**. Primers used in this study	4
**Supplementary Table 2**. Plasmids used in this study	6
**Supplementary Table 3**. DNA gyrase variants characterised in this study	8
**Supplementary Table 4.** Maximal percent cleavage values for albicidin, **Albi-1**, **Albi-2** and **Albi-3** determined for WT *E. coli* gyrase and selected mutants	9

Supplementary Figures

**Supplementary Figure 1**. Comparison of **Gyr-Mu217-albicidin** and 6RKV	10
**Supplementary Figure 2**. Comparison of CTD orientation in **Gyr-Mu217-albicidin** and 6RKW	11
**Supplementary Figure 3**. Supercoiling activity assays for gyrase variants	12
**Supplementary Figure 4**. **Albi-1** supercoiling inhibition activity assays for gyrase variants	13
**Supplementary Figure 5**. Calcium cleavage activity assays for gyrase mutants	14
**Supplementary Figure 6**. Cleavage activity assays for GyrB^{K740A} gyrase variant	15
**Supplementary Figure 7**. **Albi-1** cleavage activity assays for gyrase variants	16
**Supplementary Figure 8**. **Albi-2** and **Albi-3** cleavage activity assays for gyrase variants	17
**Supplementary Figure 9**. Cleavage assays for **AE-series** compounds and **alkyne-Albi**	18
**Supplementary Figure 10**. SDS-PAGE gel of gyrase proteins used in this study	19
**Supplementary Figure 11**. Activity of albicidin and **Albi-1/2/3** on *E. coli* topoisomerase IV	20
**Source Data Supplementary Figure 3**	21
**Source Data Supplementary Figure 10**	21

Supplementary Notes

**Supplementary Note 1**. Reagents and general information	22

Supplementary Methods

**Supplementary Method 1**. Standard procedures	24
**Supplementary Method 2**. Synthesis of **photo-Albi**	27
**Supplementary Method 3**. Synthesis of **Albi-1**	30
**Supplementary Method 4**. Synthesis of **Albi-2**	36
**Supplementary Method 5**. Synthesis of **Albi-3**	43
**Supplementary Method 6**. Synthesis of **alkyne-Albi**	46
**Supplementary Method 7**. Synthesis of **AE-1**, **AE-2** and **AE-3**	47
**Supplementary Method 8**. Synthesis of **AE-4**	51
**Supplementary Method 9**. Synthesis of **AE-5**	54
**Supplementary Method 10**. Spectral data	57

Supplementary References 104

Supplementary Tables

Supplementary Table 1. List of primers used in the study

Primer name	Primer sequence (5' – 3')	Purpose
agilentR68A-for	AGCCTATAAAAAATCTGCCGCTGTCGTTGG TGACGTAATC	Introducing mutation in *E. coli gyrA* for residue R68
agilentR68A-rev	GATTACGTCACCAACGACAGCGGCAGATTT TTTATAGGCT	Introducing mutation in *E. coli gyrA* for residue R68
GyrA-K65A-for	ACAAAGCCTATAAAGCCCTGCCCGTGTCGT TG	Introducing mutation in *E. coli gyrA* for residue K65
GyrA-K65A-rev	CAACGACACGGGCAGAGGCTTTATAGGCTT TGT	Introducing mutation in *E. coli gyrA* for residue K65
GyrA-A67Q-for	GCCTATAAAAAATCTCAGCGTGTCGTTGGT GAC	Introducing mutation in *E. coli gyrA* for residue A67
GyrA-A67Q-rev	GTCACCAACGACACGCTGAGATTTTTTATA GGC	Introducing mutation in *E. coli gyrA* for residue A67
GyrA-V70A-for	AAAATCTGCCCGTGTCGCCGGTGACGTAAT CGG	Introducing mutation in *E. coli gyrA* for residue V70
GyrA-V70A-rev	CCGATTACGTCACCGGCGACACGGGCAGA TTTT	Introducing mutation in *E. coli gyrA* for residue V70
GyrA-D72K-for	GCCCGTGTCGTTGGTAAAGTAATCGGTAAA TAC	Introducing mutation in *E. coli gyrA* for residue D72
GyrA-D72K-rev	GTATTTACCGATTACTTTACCAACGACACGG GC	Introducing mutation in *E. coli gyrA* for residue D72
GyrA-I74M-for	GTCGTTGGTGACGTAATGGGTAAATACCAT CCC	Introducing mutation in *E. coli gyrA* for residue I74
GyrA-I74M-rev	GGGATGGTATTTACCCATTACGTCACCAAC GAC	Introducing mutation in *E. coli gyrA* for residue I74
GyrA-D82N-for	TACCATCCCCATGGTAACTCGGCGGTCTAT GAC	Introducing mutation in *E. coli gyrA* for residue D82
GyrA-D82N-rev	GTCATAGACCGCCGAGTTACCATGGGGATG GTA	Introducing mutation in *E. coli gyrA* for residue D82
GyrA-S83L-for no.2	TACCATCCCCATGGTGACCTGGCGGTCTAT GACACGAT	Introducing mutation in *E. coli gyrA* for residue S83
GyrA-S83L-rev no.2	ACGATCGTGTCATAGACCGCCAGGTCACCA TGGGGAT	Introducing mutation in *E. coli gyrA* for residue S83

GyrA-M120A-for	GACTCTGCGGCGGCAGCGCGTTATACGGAAATC	Introducing mutation in *E. coli gyrA* for residue M120
GyrA-M120A-rev	GATTTCCGTATAACGCGCTGCCGCCGCAGAGTC	Introducing mutation in *E. coli gyrA* for residue M120
GyrB-K447E-for	GCGATTCTGCCGCTGGAGGGTAAAATCCTCAACG	Introducing mutation in *E. coli gyrB* for residue K447
GyrB-K447E-rev	CGTTGAGGATTTTACCCTCCAGCGGCAGAATCGC	Introducing mutation in *E. coli gyrB* for residue K447
GyrB-K447R-for no.2	AGGCGATTCTGCCGCTGCGCGGTAAAATCCTCAA	Introducing mutation in *E. coli gyrB* for residue K447
GyrB-K447R-rev no.2	CGTTGAGGATTTTACCGCGCAGCGGCAGAATCGC	Introducing mutation in *E. coli gyrB* for residue K447
GyrB-K447W-for	GCGATTCTGCCGCTGTGGGGTAAAATCCTCAACG	Introducing mutation in *E. coli gyrB* for residue K447
GyrB-K447W-rev	CGTTGAGGATTTTACCCCACAGCGGCAGAATCGC	Introducing mutation in *E. coli gyrB* for residue K447
GyrB-E744A-for	TATAAAGGTCTGGGCGCGATGAACCCGGAACAG	Introducing mutation in *E. coli gyrB* for residue E744
GyrB-E744A-rev	CTGTTCCGGGTTCATCGCGCCCAGACCTTTATA	Introducing mutation in *E. coli gyrB* for residue E744
GyrB-K740A-for	TCCATCCAGCGTTATGCCGGTCTGGGCGAGATG	Introducing mutation in *E. coli gyrB* for residue K740
GyrB-K740A-rev	CATCTCGCCCAGACCGGCATAACGCTGGATGGA	Introducing mutation in *E. coli gyrB* for residue K740
ColE1-for	GGAGCGAACGACCTACACCGAACTGAGATACCTACAGCG	Introducing point mutations in *E. coli gyrA* and *E. coli gyrB*
ColE1-rev	CGCTGTAGGTATCTCAGTTCGGTGTAGGTCGTTCGCTCC	Introducing point mutations in *E. coli gyrA* and *E. coli gyrB*
Mu217_HindIIIBglIIEcoRV_for	AATAAAGCTTAGATCTGATATCGGAGAAAGAAAGTGAAAGGAAG	Cloning of Mu217 fragment
Mu217_BamHIEcoRV_rev	AATAGGATCCGATATCTTCCTGCGCGTCCTTATATG	Cloning of Mu217 fragment

Supplementary Table 2. List of plasmids used in the study

Name	Backbone	Source	Purpose
pET28b-EcGyrATWS	pET28b(+)	Gift of Dr Valérie Lamour (University of Strasbourg)	Purification of 10xHis- and 2xSTREP-tagged *E. coli* GyrA
pET28b-EcGyrBTWS	pET28b(+)	Gift of Dr Valérie Lamour (University of Strasbourg)	Purification of 10xHis- and 2xSTREP-tagged *E. coli* GyrB
pET28b-EcGyrA-R68A-TWS	pET28b(+)	This study	Purification of 10xHis- and 2xSTREP-tagged *E. coli* GyrA-R68A
pET28b-EcGyrA-K65A-TWS	pET28b(+)	This study	Purification of 10xHis- and 2xSTREP-tagged *E. coli* GyrA-K65A
pET28b-EcGyrA-A67Q-TWS	pET28b(+)	This study	Purification of 10xHis- and 2xSTREP-tagged *E. coli* GyrA-A67Q
pET28b-EcGyrA-V70A-TWS	pET28b(+)	This study	Purification of 10xHis- and 2xSTREP-tagged *E. coli* GyrA-V70A
pET28b-EcGyrA-D72K-TWS	pET28b(+)	This study	Purification of 10xHis- and 2xSTREP-tagged *E. coli* GyrA-D72K
pET28b-EcGyrA-I74M-TWS	pET28b(+)	This study	Purification of 10xHis- and 2xSTREP-tagged *E. coli* GyrA-I74M
pET28b-EcGyrA-D82N-TWS	pET28b(+)	This study	Purification of 10xHis- and 2xSTREP-tagged *E. coli* GyrA-D82N
pET28b-EcGyrA-S83L-TWS	pET28b(+)	This study	Purification of 10xHis- and 2xSTREP-tagged *E. coli* GyrA-S83L
pET28b-EcGyrA-M120A-TWS	pET28b(+)	This study	Purification of 10xHis- and 2xSTREP-tagged *E. coli* GyrA-M120A
pET28b-EcGyrB-K447E-TWS	pET28b(+)	This study	Purification of 10xHis- and 2xSTREP-tagged *E. coli* GyrB-K447E
pET28b-EcGyrB-K447R-TWS	pET28b(+)	This study	Purification of 10xHis- and 2xSTREP-tagged *E. coli* GyrB-K447R
pET28b-EcGyrB-K447W-TWS	pET28b(+)	This study	Purification of 10xHis- and 2xSTREP-tagged *E. coli* GyrB-K447W
pET28b-EcGyrB-E774A-TWS	pET28b(+)	This study	Purification of 10xHis- and 2xSTREP-tagged *E. coli* GyrB-E774A

pET28b-EcGyrB-K740A-TWS	pET28b(+)	This study	Purification of 10xHis- and 2xSTREP-tagged *E. coli* GyrB-K740A
pUC-8xMuSGS	pUC19	This study	Purification of 217 bp Mu SGS

Mu217 sequence

Underlined is the central fragment represented in **Figure 2**

5’-
GGAGAAAGAAAGTGAAAGGAAGATAAAACGGGATTCATACACCGTTAAATACCG
GTTTAAAAATCCCGTGGCGCGTTTTAAAAAATCTGTGCGGGT**GATT**TTATGCCT
GATTCTGTTTATTGCCTCAGAGCGGCGCTGACGCGTTTTCTGATGGCATCAAAA
ATTTCCTGTTCCCCGGTCTTATCCAGCCCCATATAAGGACGCGCAGGAA-3’

Supplementary Table 3. DNA gyrase variants characterised in this study

Mutation	Observations
**$GyrA^{R68A}$**	Relaxation activity in the presence of ATP
**$GyrA^{K65A}$**	Toxicity
**$GyrA^{A67Q}$**	4-fold less active in supercoiling and cleavage activity when compared to WT gyrase<br>Relaxation activity in the presence of ATP
**$GyrA^{V70A}$**	Activity comparable to WT
**$GyrA^{D72K}$**	Activity comparable to WT
**$GyrA^{I74M}$**	Activity comparable to WT
**$GyrA^{D82N}$**	Naturally occurring mutation<br>No reduced cleavage observed
**$GyrA^{S83L}$**	Naturally occurring mutation (quinolone resistance)<br>Activity comparable to WT
**$GyrA^{M120A}$**	Relaxation activity in the presence of ATP
**$GyrB^{K447E}$**	Naturally occurring mutation (quinolone resistance)<br>Activity comparable to WT
**$GyrB^{K447R}$**	Naturally occurring mutation (quinolone resistance)<br>Activity comparable to WT
**$GyrB^{K447W}$**	Naturally occurring mutation (quinolone resistance)<br>Activity comparable to WT
**$GyrB^{E774A}$**	Not active
**$GyrB^{K740A}$**	Increased cleavage activity in the absence of inhibitor

Supplementary Table 4. Maximal percent cleavage values for albicidin, **Albi-1**, **Albi-2** and **Albi-3** determined for WT *E. coli* gyrase and selected mutants. Values are an average of 3 independent assays carried out for CC_{50} determination. Severely impaired values are indicated in red.

**Compound (% maximal cleavage)**	**Gyrase variant**						
	$Gyrase^{WT}$	$GyrA^{S83L}$	$GyrA^{V70A}$	$GyrA^{A67Q}$	$GyrA^{I74M}$	$GyrA^{M120A}$	$GyrB^{K447W}$
**Albicidin**	81±2	68±5	80±2	83±1	40±10	72±3	27±2
**Albi-1**	73±6	80±14	90±2	96±8	79±5	68±3	83±3
**Albi-2**	78±2	72±7	58±2	n.d.	n.d.	59±17	59±6
**Albi-3**	66±4	85±7	63±2	78±6	n.d.	n.d.	69±2

Supplementary Figures

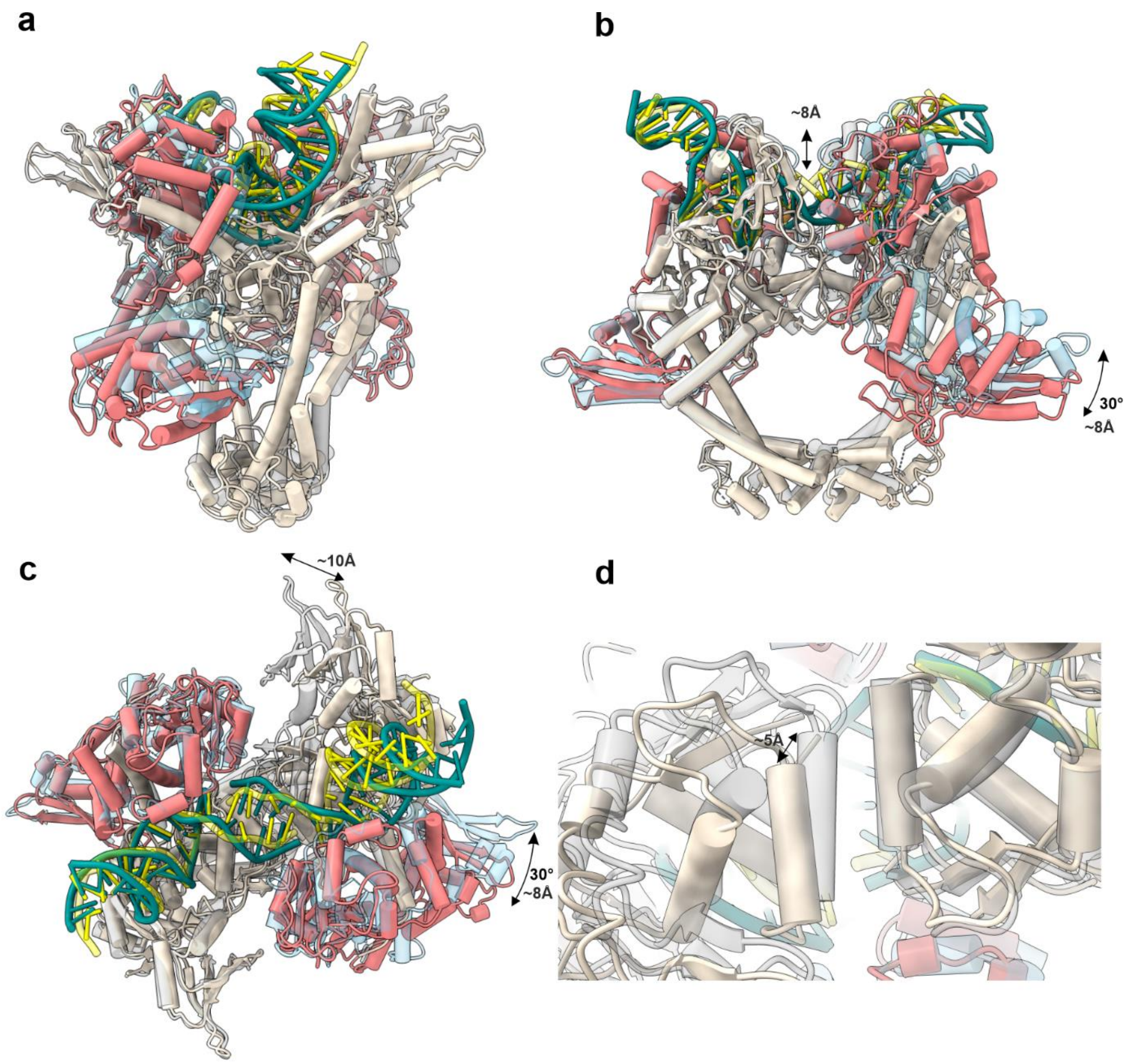

Supplementary Figure 1. Comparison of *E. coli* DNA gyrase cleavage-reunion domain in the pre-open state (PDB: 6RKV) and Gyr-Mu217-albicidin. Tube representation of **Gyr-Mu217-albicidin** (color scheme as in **Figure 1**) and overlayed **6RKV** (transparent blue). **a**: side view, **b**: front view, **c**: top view. Overall opening of the enzyme consists of movements of TOPRIM insert (downward rotation) and sliding doors movement of GyrA (see view from the bottom, **d**). The biggest movements are observed near the extremities of the enzyme i.e., 8-10 Å shifts of GyrB insert and GyrA TOWER domains. DNA cleavage leads to the 8 Å movements of DNA ends.

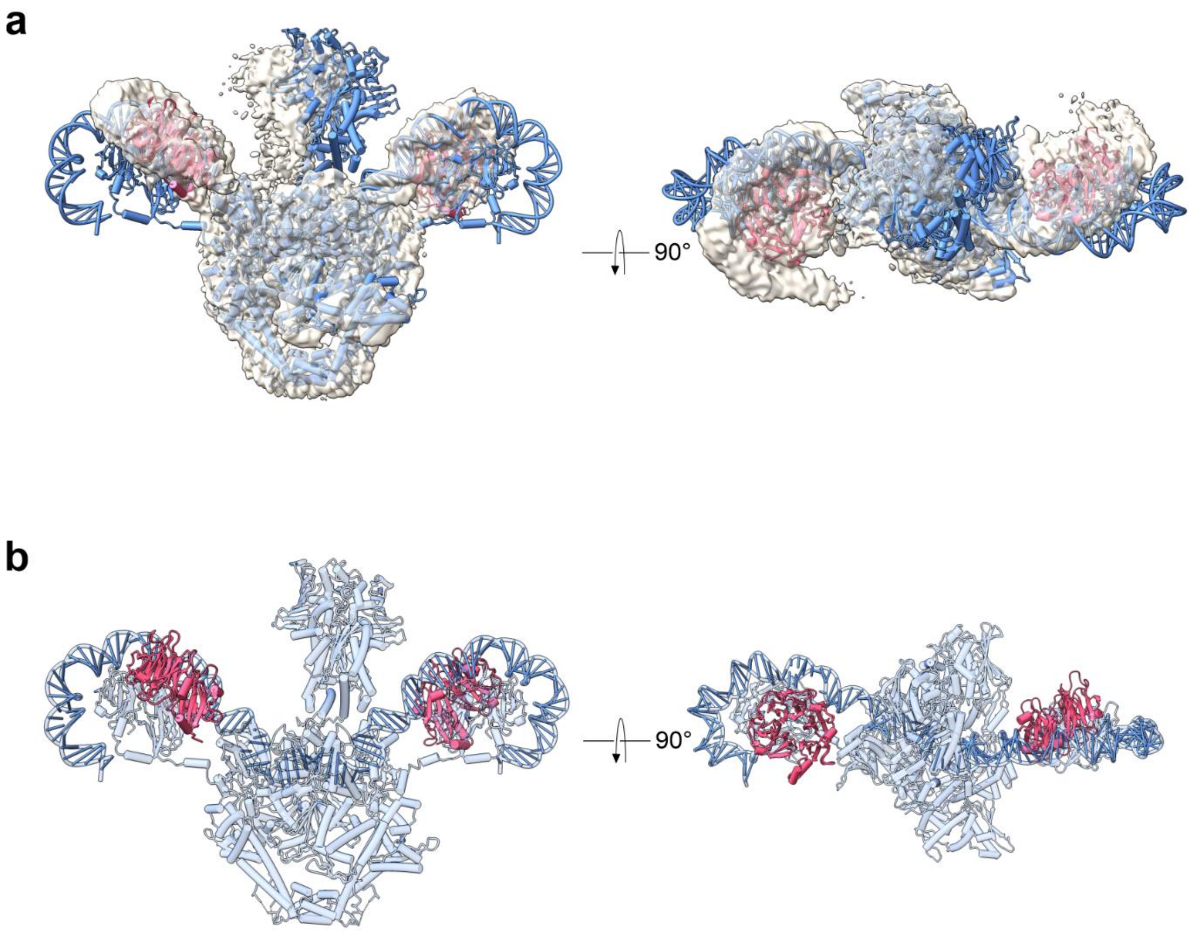

Supplementary Figure 2. Comparison of position of GyrA CTD between *E. coli* DNA gyrase-gepotidacin complex composite map (PDB:6RKW) and Gyr-Mu217-albicidin structures. a. CTD (535-875) of 6RKW was rigid-body fit in the **Gyr-Mu217-Albi2** map, low-passed to 5 Å (this dataset was selected because of better CTD density). **b.** Cartoon representations of **6RKW** (blue) and CTD from albicidin structures (magenta). Note the ~30 Å shift and 37-° rotation from one side and 35 ° rotation and 20 Å shift from another. Position of the GyrB ATPase domain is also shifted towards the centre to yield a more symmetrical structure, however, this was not modelled.

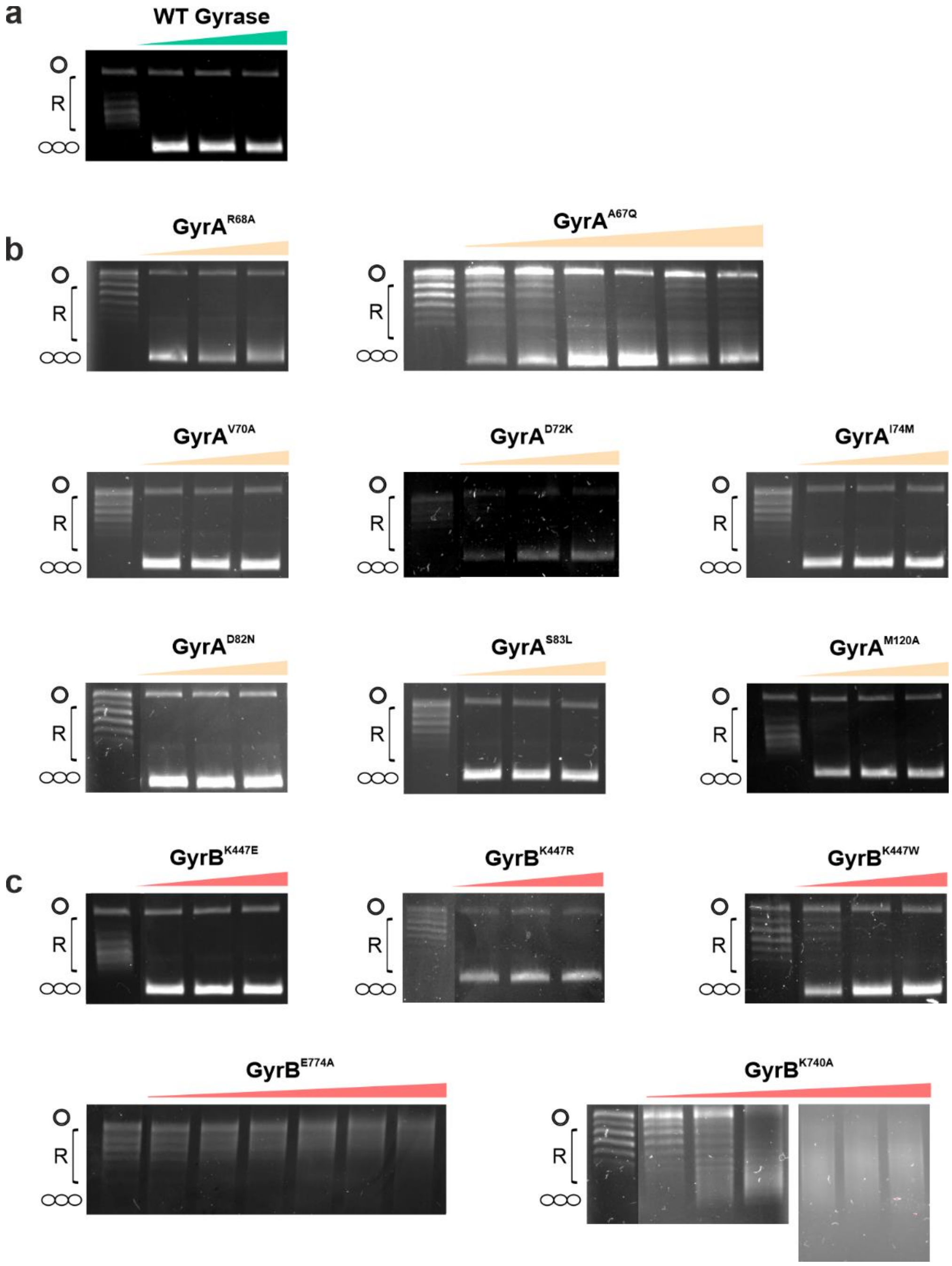

Supplementary Figure 3. Supercoiling activity assays for gyrase variants. a. Plasmid supercoiling (SC) assay showing the activity of WT gyrase. First lane: relaxed pBR322, subsequent lanes: increasing enzyme concentration (5, 10, 15 nM). Positions of nicked, relaxed and sc DNA are indicated to the left of each gel. **b**. SC assay showing the activity of GyrA variants (in presence of WT GyrB). First lane: relaxed pBR322, subsequent lanes: increasing enzyme (A_2B_2) concentration (5, 10, 15 nM). *Note: GyrA^{A67Q} shows increasing enzyme concentration to a higher amount (5, 10, 15, 20, 25, 30 nM).* **c**.SC assay showing the activity of GyrB variants in presence of equal amount of WT GyrA. First lane: relaxed pBR322, subsequent lanes: increasing enzyme (A_2B_2) concentration (5, 10, 15 nM). *Note: GyrB^{E774A} and GyrB^{K740A} show increasing enzyme concentration to higher amounts (5, 10, 15, 20, 25, 30 nM). There was no activity observed for GyrB^{E774A}.* All assays were repeated at least twice and representative gels are shown.

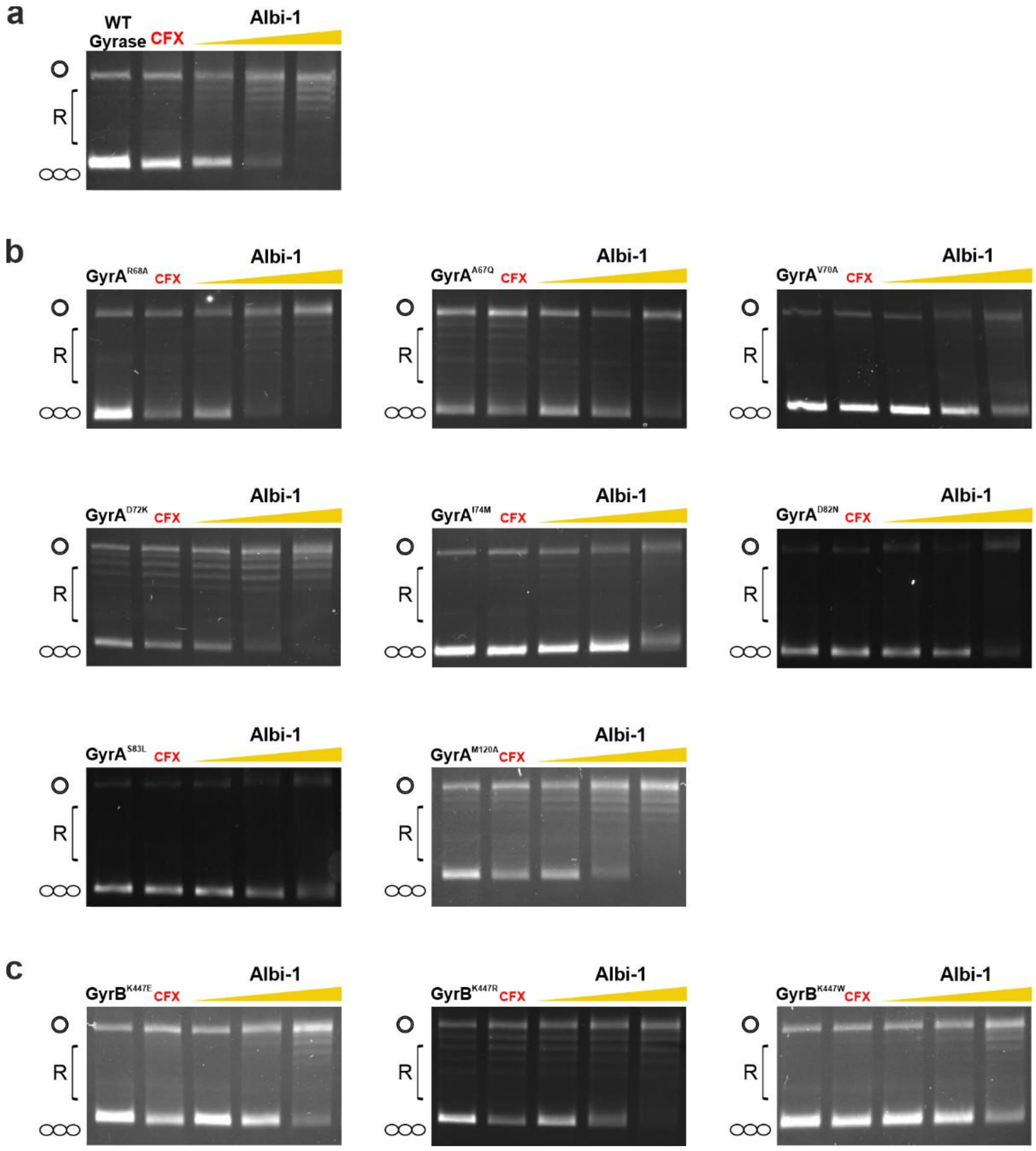

Supplementary Figure 4. Albi-1 supercoiling inhibition activity assays. a. SC assay showing inhibition of WT gyrase by **Albi-1**. First lane: relaxed pBR322, second lane: effect of 5 µM ciprofloxacin (CFX) on WT gyrase (5 nM) activity, subsequent lanes: effect of increasing **Albi-1** concentration (0.1, 1, 10 µM) on WT gyrase activity. Positions of nicked, relaxed and sc DNA are indicated to the left of each gel. **b**. SC assay showing the inhibitory activity of **Albi-1** against GyrA variants in presence of WT GyrB (concentrations are given for A_2B_2). First lane: relaxed pBR322 with specified variant (GyrA^{R68A}: 5 nM; GyrA^{A67Q}: 30 nM; GyrA^{V70A}: 5 nM; GyrA^{D72K}: 8 nM; GyrA^{I74M}: 5 nM; GyrA^{D82N}: 5 nM; GyrA^{S83L}: 5 nM; GyrA^{M120A}: 5 nM), second lane: effect of 5 µM CFX on specified variant activity, subsequent lanes: effect of increasing **Albi-1** concentration (0.1, 1, 10 µM) on specified variant activity. **c**.SC assay showing the inhibitory activity **Albi-1** against GyrB variants in presence of WT GyrA. Concentrations are given for A_2B_2. First lane: relaxed pBR322 with specified variant (GyrB^{K447E}: 5 nM; GyrB^{K447R}: 5 nM; GyrB^{K447W}: 8 nM), second lane: effect of 5 µM CFX on specified variant activity, subsequent lanes: effect of increasing **Albi-1** concentration (0.1, 1, 10 µM) on specified variant activity. All assays were repeated at least twice and representative gels are shown.

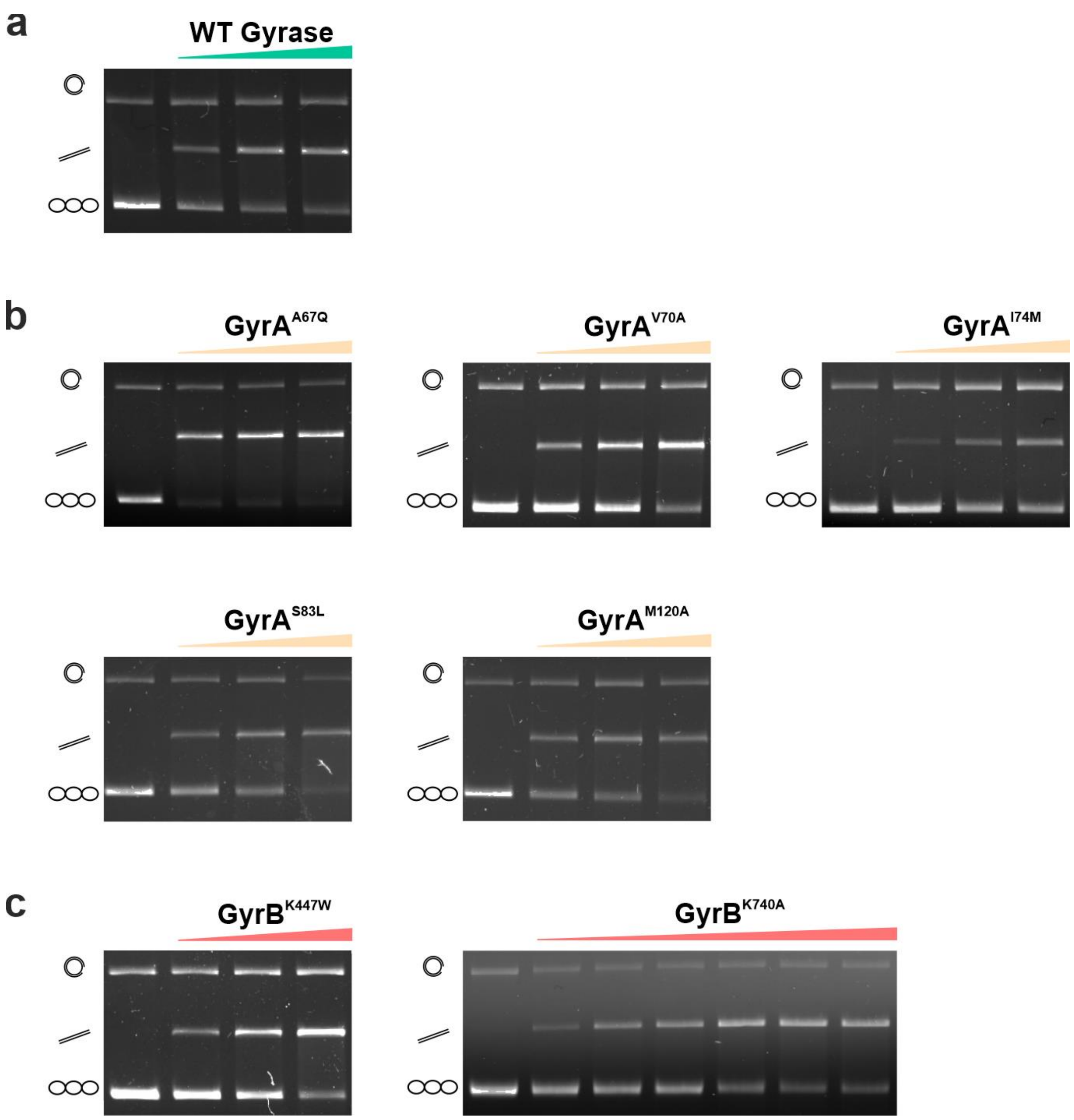

Supplementary Figure 5. Intrinsic cleavage activity assays for gyrase variants. **a**. Cleavage activity of WT gyrase. First lane: negatively supercoiled pBR322, subsequent lanes: increasing enzyme (A_2B_2) concentration (10, 20, 40 nM) in the presence of 4 mM $CaCl_2$. Positions of nicked, linear and supercoiled DNA are indicated to the left of each gel. **b**. Cleavage activity of specified GyrA variants in presence of WT GyrB. First lane: negatively supercoiled, subsequent lanes: increasing enzyme concentration ($GyrA^{A67Q}$: 100, 200, 300 nM; $GyrA^{V70A}$, $GyrA^{S83L}$, $GyrA^{M120A}$: 15, 25, 50 nM; $GyrA^{I74M}$: 10, 20, 40 nM) in the presence of 4 mM $CaCl_2$. **c**. Plasmid cleavage assay showing cleavage activity of GyrB variants in presence of WT GyrA. First lane: negatively supercoiled pBR322, subsequent lanes: increasing enzyme (A_2B_2) concentration ($GyrB^{K447W}$: 15, 25, 50 nM; $GyrB^{K740A}$: 5, 10, 15, 20, 25, 30 nM) in the presence of 4 mM $CaCl_2$. All assays were repeated at least twice and representative gels are shown.

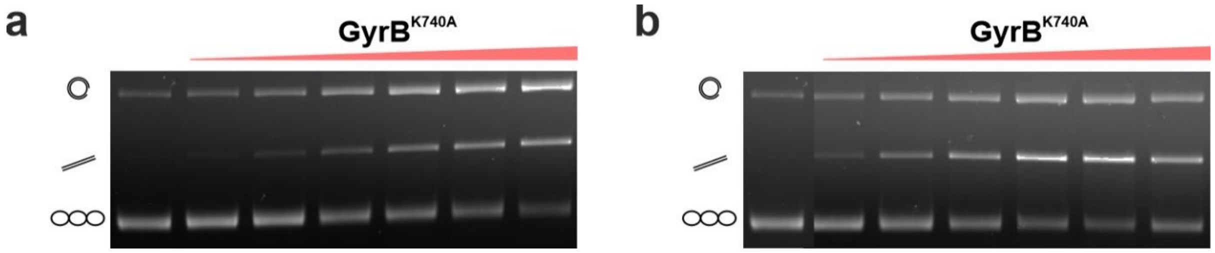

Supplementary Figure 6. Cleavage activity assays for GyrB^{K740A} variant. **a**. Cleavage activity of GyrB^{K740A} in presence of WT GyrA. First lane: negatively supercoiled pBR322, subsequent lanes: effect of increasing enzyme (A_2B_2) concentration (5, 10, 15, 20, 25, 30 nM) in the presence of 4 mM $MgCl_2$. Positions of nicked, linear and supercoiled DNA are indicated to the left of each gel. **b**. Cleavage activity of GyrB^{K740A} in presence of WT GyrA. First lane: negatively supercoiled pBR322, subsequent lanes: effect of increasing enzyme (A_2B_2) concentration (5, 10, 15, 20, 25, 30 nM) in the presence of 4 mM $MgCl_2$ and 3 µM CFX. All assays were repeated at least twice and representative gels are shown.

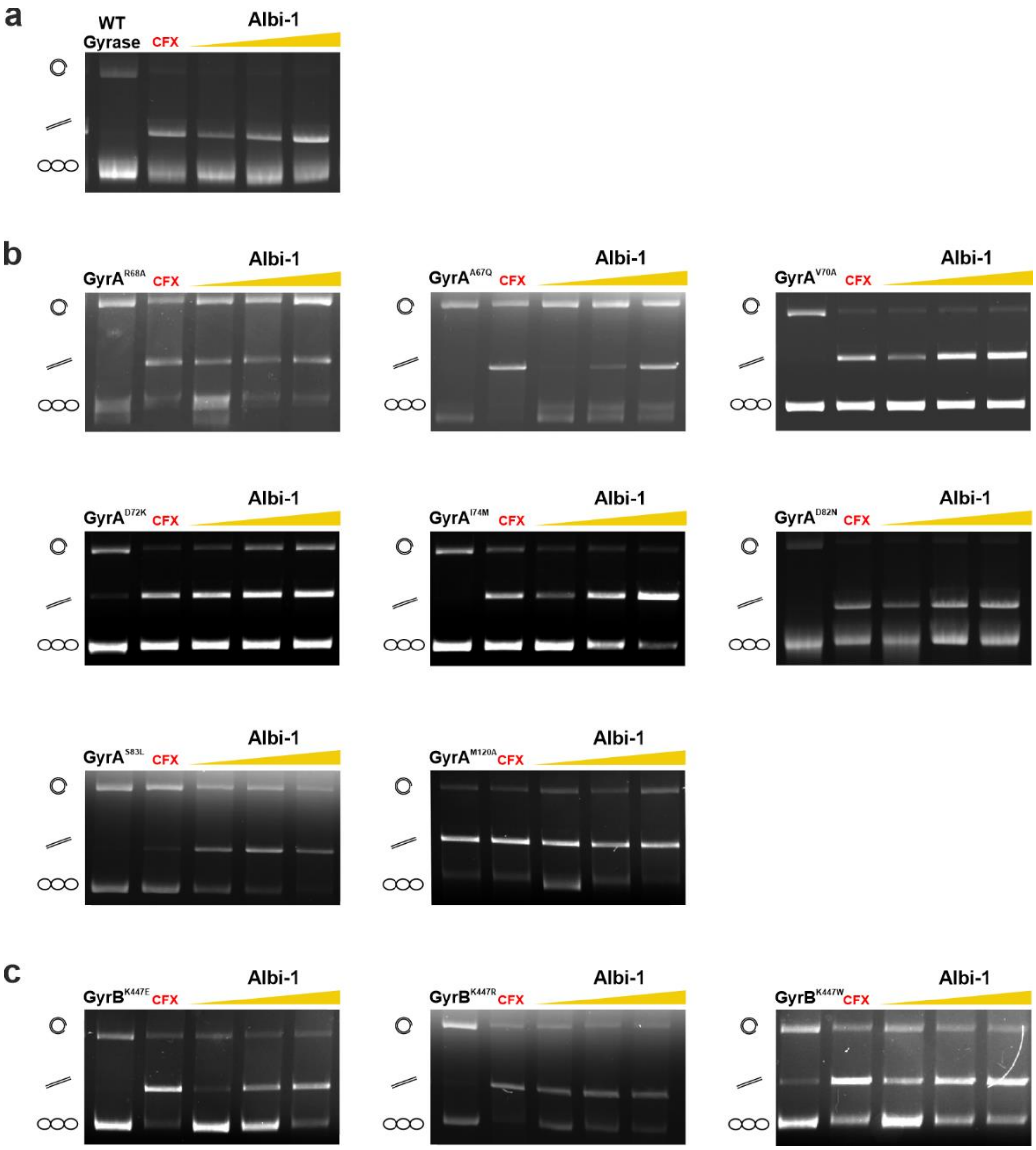

Supplementary Figure 7. Albi-1 cleavage activity assays for gyrase variants. a. Cleavage activity of WT gyrase. First lane: negatively supercoiled pBR322 with 20 nM WT gyrase (here and below all concentrations given for A_2B_2), second lane: effect of 5 µM ciprofloxacin (CFX) on WT gyrase activity, subsequent lanes: effect of increasing **Albi-1** concentration (0.1, 1, 10 µM) on WT gyrase activity. Positions of nicked, linear and sc DNA are indicated to the left of each gel. **b**. Cleavage activity of GyrA variants in presence of WT GyrB and **Albi-1**. First lane: negatively supercoiled pBR322 with specified variant (GyrA^{R68A}: 20 nM; GyrA^{A67Q}: 100 nM; GyrA^{V70A}: 15 nM; GyrA^{D72K}: 20 nM; GyrA^{I74M}: 20 nM; GyrA^{D82N}: 20 nM; GyrA^{S83L}: 25 nM; GyrA^{M120A}: 25 nM), second lane: effect of 5 µM CFX on specified variant activity, subsequent lanes: effect of increasing **Albi-1** concentration (0.1, 1, 10 µM) on specified variant activity. **c**. Cleavage activity of GyrB variants in presence of WT GyrA and **Albi-1**. First lane: negatively supercoiled pBR322 with specified variant (GyrB^{K447E}: 25 nM; GyrB^{K447R}: 20 nM; GyrB^{K447W}: 25 nM), second lane: effect of 5 µM CFX on specified variant activity, subsequent lanes: effect of increasing **Albi-1** concentration (0.1, 1, 10 µM) on specified variant activity. All assays were repeated at least twice and representative gels are shown.

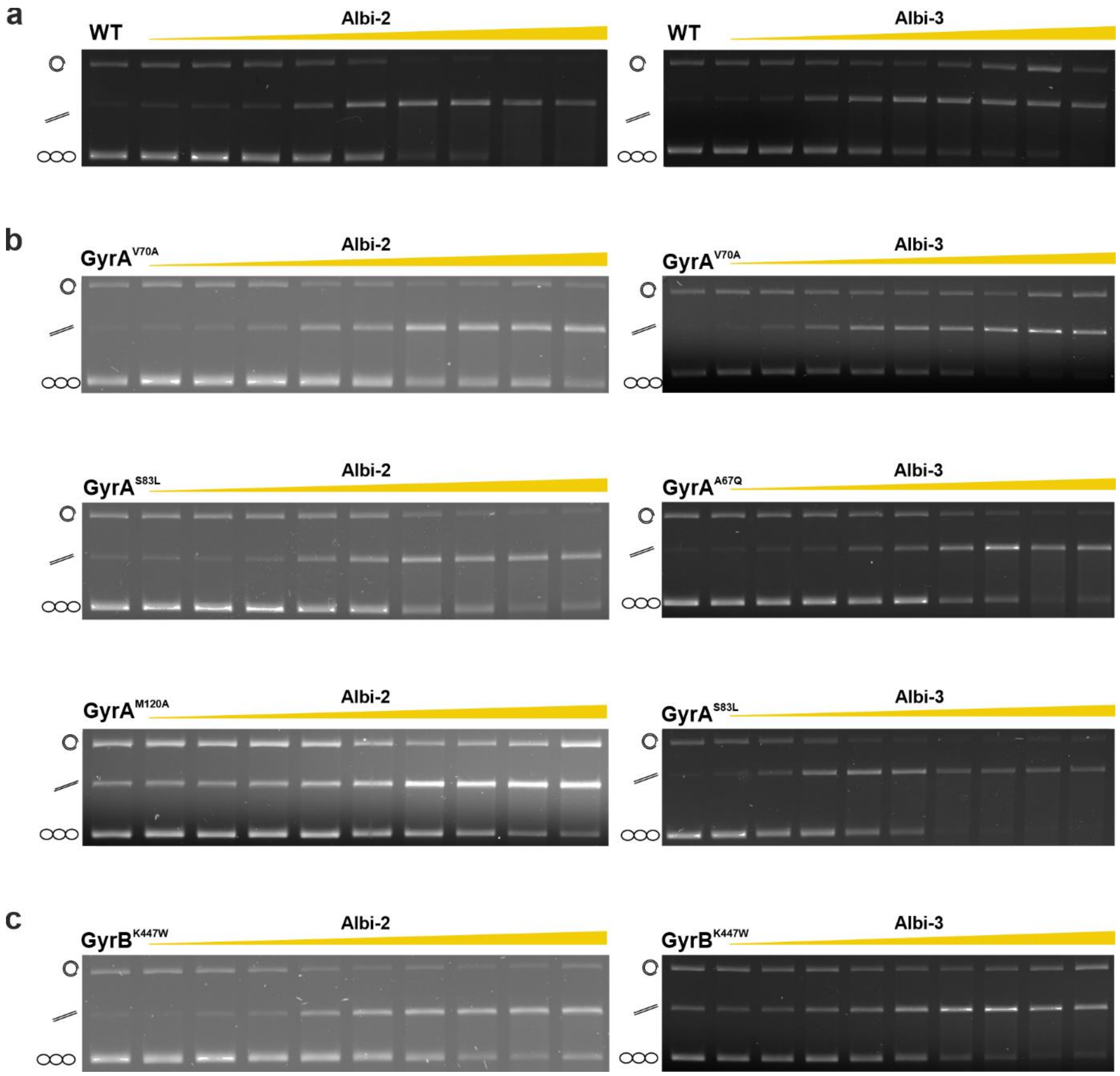

Supplementary Figure 8. Albi-2 and Albi-3 cleavage activity assays for gyrase variants. a. Cleavage activity of WT gyrase in the presence of **Albi-2** or **Albi-3**. First lane: innate cleavage activity of WT gyrase (20 nM) on negatively supercoiled pBR322; subsequent lanes: effect of increasing compound concentration (0.001, 0.005, 0.01, 0.05, 0.1, 0.5, 1, 5, 10 µM). Positions of nicked, linear and sc DNA are indicated to the left of each gel. **b**. Cleavage activity of GyrA variants in the presence of **Albi-2** or **Albi-3** and WT GyrB (concentrations given for A_2B_2). First lane: innate cleavage activity of specified variant (GyrA^{V70A}: 15 nM; GyrA^{S83L}: 25 nM; GyrA^{M120A}: 25 nM; GyrA^{A67Q}: 100 nM) on negatively supercoiled pBR322; subsequent lanes: effect of increasing compound concentration (0.001, 0.005, 0.01, 0.05, 0.1, 0.5, 1, 5, 10 µM). **c**. Cleavage activity of GyrB^{K447W} in the presence of **Albi-2** or **Albi-3** and WT GyrA. First lane: innate cleavage activity GyrB^{K447W} (25 nM) on negatively supercoiled pBR322; subsequent lanes: effect of increasing compound concentration (0.001, 0.005, 0.01, 0.05, 0.1, 0.5, 1, 5, 10 µM). All assays were carried out in triplicates and representative gels are shown.

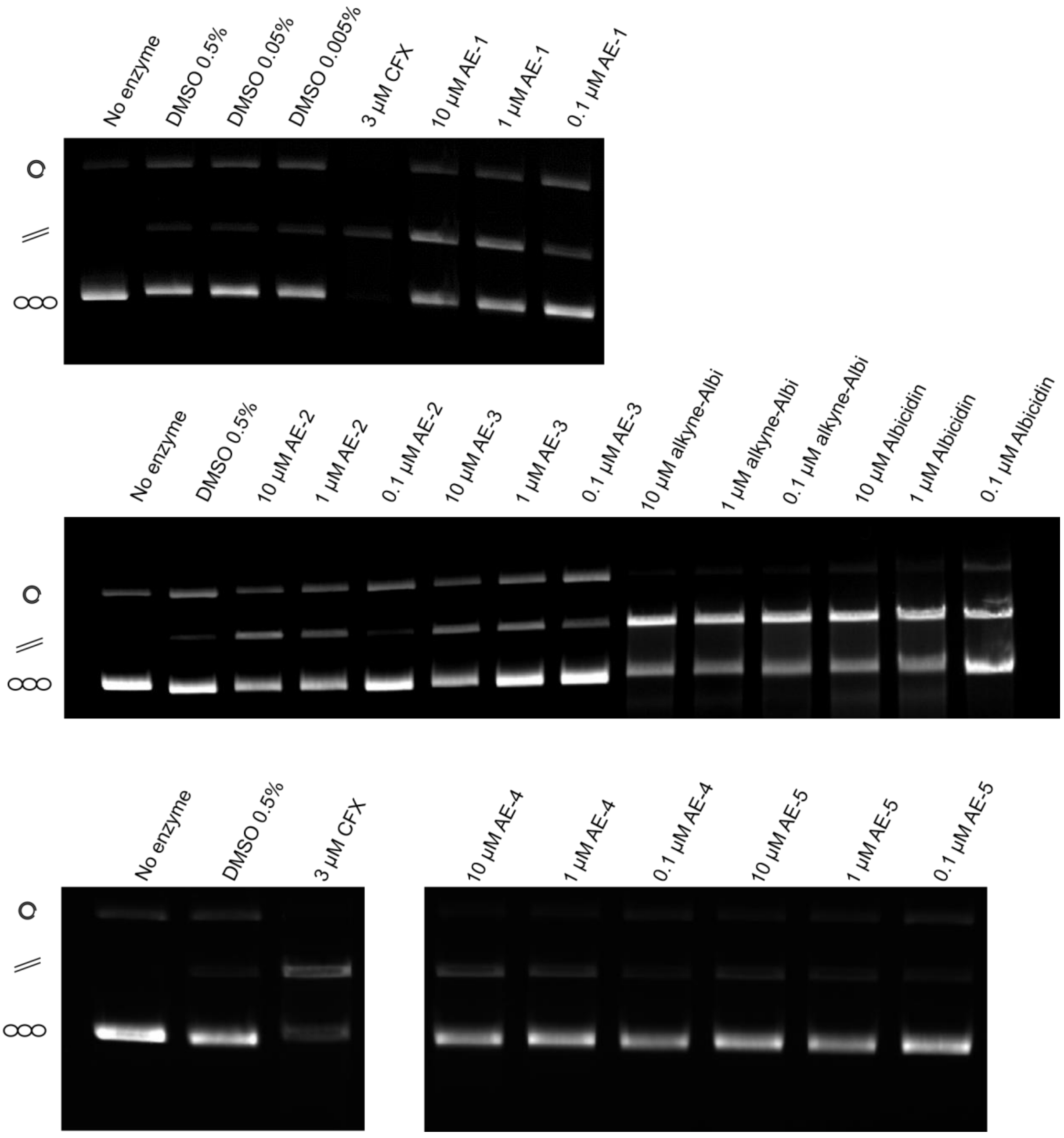

Supplementary Figure 9. Cleavage assays for AE-series compounds and alkyne-Albi. Cleavage assays with WT gyrase (20 nM) were carried out as described in the *Methods* section, concentrations of tested compounds are indicated. Nicked circular, linear and supercoiled plasmid DNA bands are labelled. All assays were repeated twice and representative gels are shown.

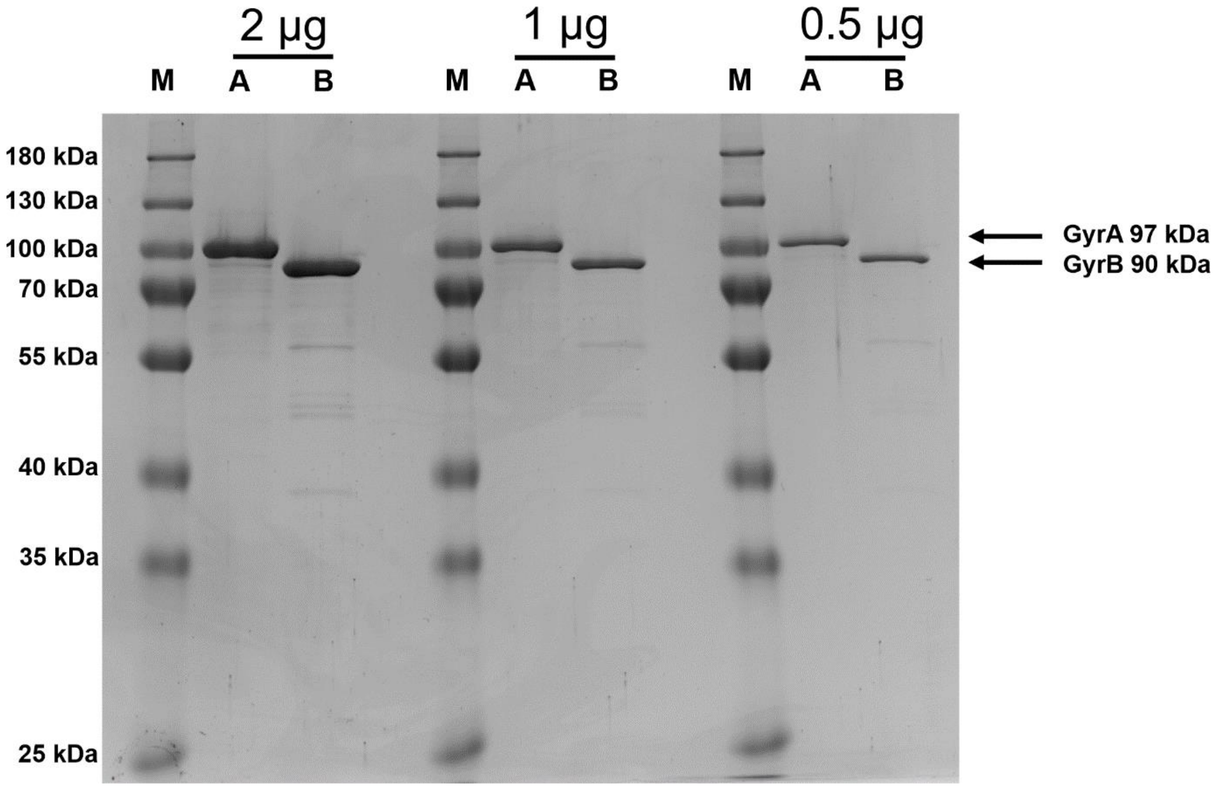

Supplementary Figure 10. SDS-PAGE gel of gyrase proteins used in this study. Three concentrations of GyrA and GyrB proteins were loaded alongside with Thermo PageRuler protein molecular weight marker as indicated. Positions of GyrA and GyrB are indicated.

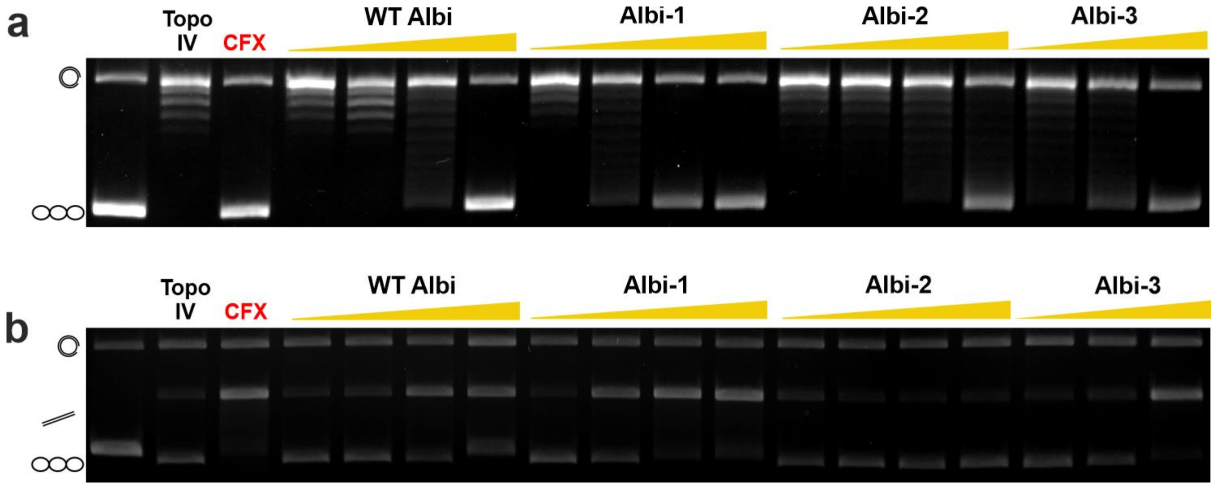

Supplementary Figure 11. **Activity of albicidin and Albi-1/2/3 on *E. coli* topoisomerase IV. a**. Plasmid relaxation activity of *E. coli* topo IV in the presence of albicidin, **Albi-1**, **Albi-2** and **Albi-3**. First lane: negatively supercoiled pBR322; second lane: innate relaxation activity of *E. coli* topo IV (12.5 nM); third lane: effect of 20 µM CFX; subsequent lanes: effect of increasing compound concentration (0.1, 1, 10, 100 µM). Note: 100 µM **Albi-3** was omitted from the assay due to solubility issues at the higher concentration. **b.** Cleavage activity of *E. coli* topo IV in the presence of albicidin, **Albi-1**, **Albi-2** and **Albi-3**. First lane: negatively supercoiled pBR322; second lane: innate cleavage activity of *E. coli* topo IV (15 nM); third lane: effect of 20 µM CFX; subsequent lanes: effect of increasing compound concentration (0.1, 1, 10, 100 µM). Note: 100 µM **Albi-3** was omitted from the assay due to solubility issues at the higher concentration. All experiments were carried out in triplicates and representative gels are shown

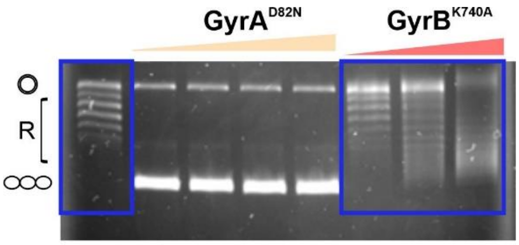

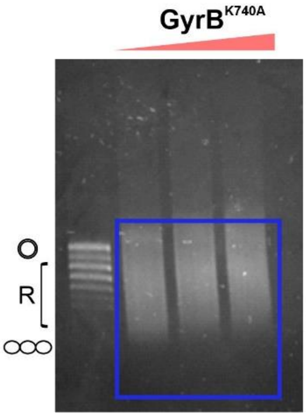

Source Data Supplementary Figure 3. Uncropped gels for **Supplementary Figure 3C**. Blue boxes indicate parts of the gels displayed in the figure.

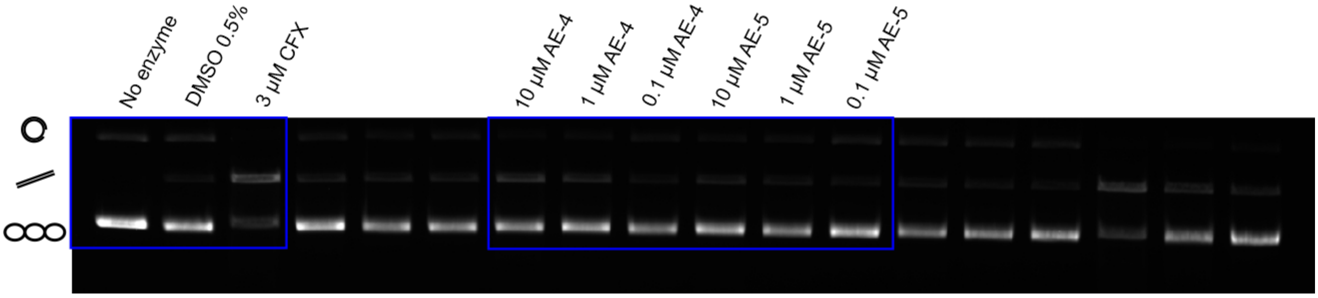

Source Data Supplementary Figure 10. Uncropped gel for **Supplementary Figure 9**. Blue boxes indicate parts of the gels displayed in the figure.

Supplementary Notes

Supplementary Note 1. Reagents and general information

Commercially available reagents (*Carl Roth GmbH* and *Co. KG*, Karlsruhe, Germany; *Sigma-Aldrich*, Taufkirchen, Germany; *Iris Biotech GmbH*, Marktredwitz, Germany; *Orpegen*, Heidelberg, Germany; *ABCR*, Karlsruhe, Germany; *Alfa Aesar*, Karlsruhe, Germany; *Merk*, Darmstadt, Germany; *TCI*, Eschborn, Germany; *VWR International GmBH*, Darmstadt, Germany; *Acros*, Geel, Belgium) and solvents (*Fisher Scientific-Acros*, Schwerte, Germany) were used without further purification. Whenever necessary, reactions were carried out under an atmosphere of argon or nitrogen and in dry solvents. HPLC solvents (*Fisher Scientific-Acros*, Schwerte, Germany) and NMR solvents (*Deutero GmbH*, Kastellaun, Germany; *Sigma-Aldrich*, Taufkirchen, Germany) were used without further purification. ^{1}H and ^{13}C NMR spectra were recorded at 298 K using Bruker Avance-II 400 MHz, Bruker Avance-III 500 MHz or Bruker Avance-III 700 MHz instruments (*Bruker*, Karlsruhe, Germany). The chemical shifts are reported in parts per million (ppm) using the residual solvent peak as an internal reference (DMSO-d_6, $CDCl_3$). Multiplicity (br. s = broad singlet, s = singlet, d = doublet, dd = doublet of doublet, ddd = doublet of doublets of doublets, t = triplet, q = quartet, m = multiplet, dt = doublet of triplets, dddt = doublet of doublets of doublets of triplets) and coupling constants (*J* in Hz) are quoted where possible. NMR spectra were analyzed using TopSpin3.1 (*Bruker Biospin, Karlsruhe, Germany*), the ACD/Spectrus Processor (*ACD/Labs*, Toronto, Ontario, Canada) or MestReNova (*Mestrelab Research S.L.*, Santiago de Compostela, Spain). 2D NOESY and ROESY spectra were recorded with mixing times of 400 ms and 300 ms, respectively.

HPLC-ESI-HRMS spectra were recorded on a QTrap LTQ XL (*Thermo Fisher Scientific*, Waltham, Massachusetts, USA) with an Agilent 1200 Series HPLC-System (*Agilent Technologies*, Waldbronn, Germany) using a reversed-phase C18 column (Hypersil 100, 150 x 4.6 mm, particle size 5 µm, *Thermo Fisher Scientific*, Waltham, Massachusetts, USA). Eluent A comprised water with 0.1% formic acid; eluent B consisted of methanol with 0.1% formic acid. A flow rate of 3 mL min^{-1} was used. HRMS spectra were analyzed using Xcalibur (*Thermo Fisher Scientific GmbH*, Bremen, Germany). All biologically tested compounds had a purity of >95%.

Reactions and purifications were monitored by analytical thin layer chromatography (TLC) on aluminium-backed plates coated with *Macherey-Nagel* silica gel (60, F254)

using solvent systems based on ethyl acetate, *n*-hexane, dichloromethane, and methanol. Analysis was performed by visualizing under UV light (λ = 254 nm), by staining with $KMnO_4$-solution ($KMnO_4$ (3 g), K_2CO_3 (20 g, H_2O (300 mL), 5% $NaOH_{(aq.)}$ (5 mL)) and with ninhydrin-solution (ninhydrin (0.3 g), AcOH (3 mL), *n*-BuOH (100 mL)). Flash chromatography was performed on silica gel (particle size 40-63 μm, *VWR Chemicals*, Darmstadt, Germany) and solvent mixtures based on ethyl acetate, *n*-hexane, dichloromethane, and methanol. Preparative HPLC was carried out on a 1260 Infinity (*Agilent Technologies*, Waldbronn, Germany) system with a polymeric reversed phase column (PLRP-S 100A, 300 x 50 mm, particle size 10 μm, *Agilent Technologies*, Waldbronn, Germany). Eluent A was water with 0.1% trifluoroacetic acid and eluent B was acetonitrile with 0.1% trifluoroacetic acid. A flow rate of 70 mL min^{-1} was used.

Supplementary Methods

Supplementary Method 1. Standard procedures

Standard Procedure A – Reduction of nitro compounds using zinc

To a solution or suspension of the aromatic nitro compound in either EtOH (abs.) or a mixture of EtOH (abs.) and THF was added AcOH. The resulting mixture was cooled down to 0°C and zinc powder was added carefully in portions. After fading of the exothermic reaction, the ice bath was removed, allowing the reaction mixture to warm up to r.t. Upon completion of the reaction (TLC monitoring), the solids were removed by filtration through a pad of Celite, washed with little EtOAc and THF, and the filtrate was concentrated *in vacuo*. The acidic residue was taken up in EtOAc and carefully washed with saturated aqueous $NaHCO_3$ (3×) and brine. After drying over anhydrous Na_2SO_4, the solvent was removed under reduced pressure to obtain either the analytically pure product or a crude product that required purification by column chromatography.

Standard Procedure B – Hydrolysis of benzoic esters

To a solution of the benzoic acid ester in a mixture of THF and MeOH was slowly added 3 N $KOH_{(aq)}$. After complete conversion of the starting material (TLC monitoring), the reaction mixture was acidified to pH ~2 by the addition of 3 N $HCl_{(aq.)}$. Workup method A: In case a precipitate was formed, it was isolated by filtration through a sintered funnel, washed with little water and dried under high vacuum to obtain the analytically pure product. Workup method B: In case no precipitate was formed, MeOH and THF were evaporated under reduced pressure and the aq. residue was extracted with EtOAc (3×). The combined organic layers were washed with brine, dried over anhydrous Na_2SO_4, and concentrated under reduced pressure to yield the analytically pure product.

Standard Procedure C – Peptide coupling via benzoyl chloride formation using BTC

Bis(trichloromethyl) carbonate (BTC, triphosgene) was added to a solution of the benzoic acid in dry THF and the solution was cooled down to 0°C. 2,4,6-Collidine was

added dropwise and the resulting suspension was stirred at that temperature for 45 min. Subsequently, a premixed solution of the aniline and DIPEA in dry THF was added dropwise to the suspension and the reaction mixture was stirred for 16 h while warming up to r.t. After removing all volatiles under reduced pressure, the residue was taken up in EtOAc and washed with 1 N $HCl_{(aq.)}$ (3×), saturated aqueous $NaHCO_3$ (3×), and brine. The organic layer was dried over anhydrous Na_2SO_4 and concentrated *in vacuo* to obtain the crude product, which was purified by column chromatography on silica gel.

Standard Procedure D – Peptide coupling via mixed anhydride formation using EEDQ

To a solution of the amino acid in dry THF was added EEDQ and the reaction mixture was stirred at r.t. for 15 min. A premixed solution of the tripeptide in dry THF was added slowly and the reaction mixture was stirred at r.t. for 72 h. The organic solvent was evaporated under reduced pressure and the residue was partitioned between EtOAc and 1 N $HCl_{(aq.)}$. The organic layer was washed with 1 N $HCl_{(aq.)}$ (2×), brine, dried over anhydr. Na_2SO_4, and concentrated *in vacuo* to afford the crude product, which was purified by column chromatography.

Standard Procedure E – Boc/*t*Bu-deprotection using 4 N HCl in 1,4-dioxane

A solution of the Boc/*t*Bu-protected tetrapeptide in 4 N HCl in 1,4-dioxane was stirred at r.t. for 1 h. Subsequently, all volatiles were removed under reduced pressure to obtain the crude product, which was taken up in H_2O and little CH_3CN and freeze-dried to afford the analytically pure desired product without further purification.

Standard Procedure F – Pd-mediated allyl-deprotection

The allyl-protected tetrapeptide was dissolved in dry THF and morpholine was added, followed by either $Pd(PPh_3)_4$ or $Pd(Ph_3)_2Cl_2$. The reaction mixture was stirred at r.t. for 16 h under the exclusion of light (aluminum wrap). After removing all volatiles under reduced pressure, the residue was taken up in EtOAc and washed with 1 N $HCl_{(aq.)}$ (3×) and brine. The organic phase was dried over anhydrous Na_2SO_4 and concentrated *in vacuo* to obtain the crude product, which was purified by column chromatography on silica gel.

Standard Procedure G – Peptide coupling via active ester formation using PCP

The amine and the PCP active ester were dissolved in a mixture of anhydrous DMF and Et_3N was added. The reaction mixture was stirred at r.t. for 16 h under the exclusion of light. All volatiles were removed *in vacuo* and the residue was taken up in a mixture of THF and MeOH (1:1 v/v), and 3 N $KOH_{(aq)}$ was added dropwise at 0 °C. The ice bath was removed and after 15 min of stirring the suspension was acidified to pH ≈ 2 by the addition of 3 N $HCl_{(aq.)}$. The resulting suspension was concentrated under reduced pressure and the crude material was dissolved in DMSO and purified by HPLC (PLRP-S column, CH_3CN in H_2O).

Standard Procedure H – Formation of acid chloride

The carboxylic acid (1.00 eq) was dissolved in $SOCl_2$. The reaction mixture was stirred at r.t. or reflux. The thionyl chloride was removed *in vacuo* until the residue was dry. The crude product was used in the next reaction without further treatment.

Standard Procedure I – Peptide coupling via active ester formation using acid chloride

The amine (or alcohol) and the acid chloride active ester were dissolved in anhydrous THF and Et_3N or DIPEA was added. The reaction mixture was stirred at r.t. for 16 h. All volatiles were removed *in vacuo,* the residue was taken up in ethyl acetate and washed with 1 N $HCl_{(aq.)}$ (2×), water (2x) and brine. The organic phase was dried over anhydrous Na_2SO_4 and concentrated *in vacuo* to obtain the crude product, which was purified by column chromatography on silica gel.

Supplementary Method 2. Synthesis of Photo-Albi

Synthesis of AB fragment

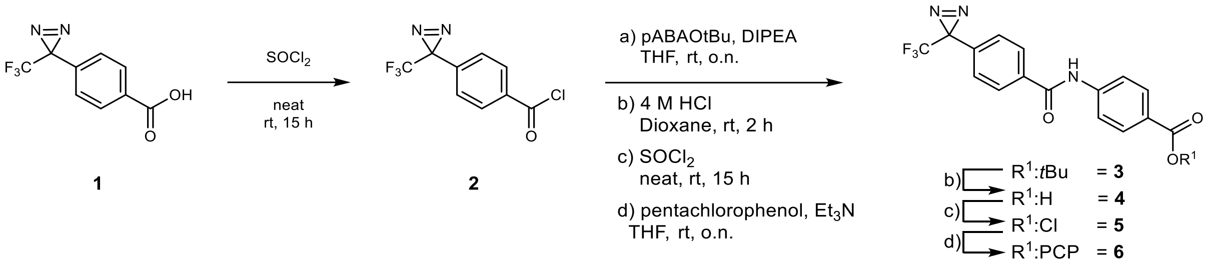

Compound 2

Compound **2** was synthesized from carboxylic acid **1** (500 mg, 2.17 mmol) according to s*tandard procedure H* - r.t., 15 h).

Compound 3

Compound **3** was synthesized from acid chloride **2** (540 mg, 2.17 mmol, 1.00 eq.), *p*ABAO*t*Bu (274 mg, 1.81 mmol, 1.00 eq.) and DIPEA (1.14 mL, 6.42 mmol, 3.00 eq.) according to s*tandard procedure I* - column chromatography: SiO_2, *n*-hexane/EtOAc, 10:3. Compound **3** (631 mg, 80%) was obtained as a light-yellow solid. ^{1}H NMR (DMSO-d_6, 500 MHz): δ = 10.65 (s, 1 H), 8.08 (d, *J*=8.7 Hz, 2 H), 7.90 (s, 4 H), 7.46 (d, *J*=8.1 Hz, 2 H), 1.55 ppm (s, 9 H) ^{13}C NMR (DMSO-d_6, 126 MHz): δ = 165.3, 165.0, 130.4, 129.2, 127.0, 120.0, 80.85, 28.3 ppm ^{19}F NMR (DMSO-d_6, 471 MHz): δ = -64.44 ppm. HRMS (ESI): m/z calculated for $C_{20}H_{19}F_3N_3O_3$ $(M+H)^+$ 406.1373, found 406.1368.

Compound 4

Compound **4** was synthesized from *t*Bu-ester protected dipeptide **3** (620 mg, 1.53 mmol, 1.00 eq.) with 4 N HCl in dioxane (8 mL) according to *standard procedure E*. Compound **4** (534 mg, quant.) was obtained as colorless solid. HRMS (ESI): m/z calculated for $C_{16}H_{11}F_3N_3O_3$ $(M+H)^+$ 350.0747, found 350.0746.

Compound 5

Compound **5** was synthesized at r.t., 15 h from carboxylic acid **4** (505 mg, 1.45 mmol) according to s*tandard procedure H*.

Compound 6

Compound **6** was synthesized from acid chloride **5** (532 mg, 1.45 mmol, 1.00 eq.), pentachlorophenol (578 mg, 2.17 mmol, 1.50 eq.) and Et_3N (0.806 mL, 5.78 mmol, 4.00 eq.) according to s*tandard procedure I* - column chromatography: SiO_2, *n*-hexane/EtOAc, 10:1. Compound **6** (565 mg, 68%) was obtained as a colorless solid. ^{1}H NMR (DMSO-d^6, 500 MHz): δ = 10.87 (s, 1 H), 8.20 - 8.24 (m, 2 H), 8.06 - 8.13 (m, 4 H), 7.49 ppm (d, *J*=8.2 Hz, 2 H) HRMS (ESI): m/z calculated for $C_{22}H_{10}Cl_5F_3N_3O_3$ $(M+H)^+$ 597.9082, found 597.9082.

Synthesis of tetrapeptide 10

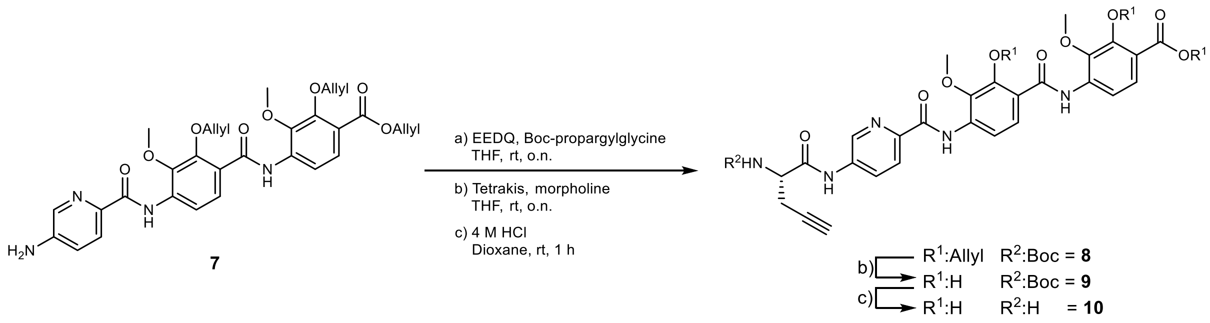

Compound 8

Compound **8** was synthesized from Boc-propargyl glycine (120 mg, 0.561 mmol, 1.1 eq.), tripeptide **7**[1] (300 mg, 0.510 mmol, 1.00 eq.) and EEDQ (378 mg, 1.53 mmol, 3.00 eq.) according to s*tandard procedure D* - column chromatography: SiO_2, 1-5% MeOH in DCM. Compound **8** (225 mg, 56%) was obtained as a yellow solid. ^{1}H NMR (DMSO-d_6, 500 MHz): δ = 10.63 (s, 1 H), 10.44 (s, 1 H), 8.31 - 8.41 (m, 2 H), 7.97 - 8.04 (m, 2 H), 7.84 (d, *J*=8.9 Hz, 1 H), 7.87 (d, *J*=8.5 Hz, 1 H), 7.56 (d, *J*=8.9 Hz, 1 H), 7.06 (dd, *J*=8.5, 2.7 Hz, 1 H), 6.25 (s, 2 H), 5.99 - 6.15 (m, 3 H), 5.36 - 5.43 (m, 3 H), 5.22 - 5.31 (m, 3 H), 4.83 (d, *J*=6.3 Hz, 2 H), 4.76 (d, *J*=5.5 Hz, 2 H), 4.54 (d, *J*=5.6 Hz, 2 H), 3.96 (s, 3 H), 3.92 ppm (s, 3 H) ^{13}C NMR (DMSO-d_6, 126 MHz): δ = 133.1, 127.0, 120.8, 119.8, 118.6, 118.3, 115.2, 75.6, 75.0, 65.6, 61.5 ppm. HRMS (ESI): m/z calculated for $C_{41}H_{46}N_5O_{11}$ $(M+H)^+$ 784.3188, found 784.3195.

Compound 9

Compound **9** was synthesized from allyl-protected tetrapeptide **8** (200 mg, 255 µmol, 1.00 eq.), $Pd(PPh_3)_4$ (118 mg, 102 µmol, 0.400 eq.) and morpholine (440 µl, 5.10 mmol, 20.0 eq.) according to *standard procedure F* - column chromatography:

SiO_2, 1-7% MeOH in DCM. Compound **9** (210 mg, 98%) was obtained as a yellow solid. HRMS (ESI): m/z calculated for $C_{41}H_{46}N_5O_{11}$ $(M+H)^+$ 664.2249, found 664.2274.

Compound 10

Compound **10** was synthesized from Boc-protected tetrapeptide **9** (210 mg, 316 µmol, 1.00 eq.) with 4 N HCl in dioxane (7 mL) according to *standard procedure E*. Compound **10** (178 mg, quant.) was obtained as light-yellow solid. HRMS (ESI): m/z calculated for $C_{27}H_{26}N_5O_9$ $(M+H)^+$ 564.1725, found 564.1722.

Final coupling of Photo-Albi

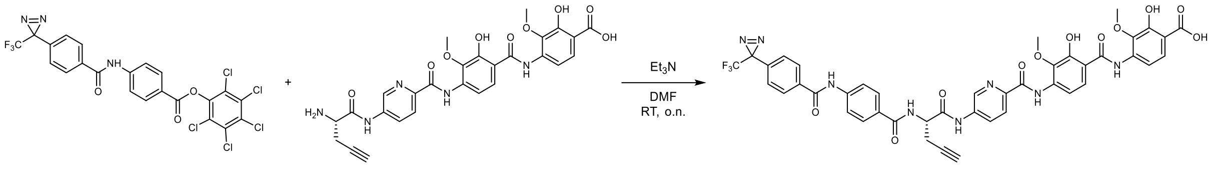

Photo-Albi was synthesized from PCP-ester **6** (110 mg, 184 µmol, 1.40 eq.), tetrapeptide **10** (74.1 mg, 132 µmol, 1.00 eq.) and Et_3N (92 µl, 0.66 mmol) according to *standard procedure G.* Final derivative **Photo-Albi** (12 mg, 10%) was obtained as a colorless solid. ^1H NMR (DMSO-d_6, 400 MHz): δ = 11.76 (br. s., 1 H), 11.04 (br. s., 1 H), 10.99 (br. s., 1 H), 10.66 (s, 1 H), 10.49 (s, 1 H), 9.00 (br. s., 1 H), 8.86 (d, *J*=7.3 Hz, 1 H), 8.38 (d, *J*=7.0 Hz, 1 H), 8.21 (d, *J*=8.8 Hz, 1 H), 8.10 (d, *J*=8.5 Hz, 2 H), 7.94 - 8.02 (m, 2 H), 7.84 - 7.94 (m, 3 H), 7.75 (s, 1 H), 7.55 (d, *J*=8.5 Hz, 1 H), 7.46 (d, *J*=7.5 Hz, 2 H), 4.76 - 4.90 (m, 1 H), 3.90 (s, 3 H), 3.88 ppm (s, 3 H) ^{13}C NMR (DMSO-d_6, 126 MHz): δ = 163.1, 140.1, 133.9, 129.3, 129.0, 129.0, 127.7, 127.1, 126.9, 125.9, 123.5, 120.2, 120.0, 119.7, 110.6, 110.2, 61.0, 60.3, 53.7, 45.7 ^{19}F NMR (DMSO-d_6, 471 MHz): δ = -64.43 ppm. HRMS (ESI): m/z calculated for $C_{43}H_{34}F_3N_8O_{11}$ $(M+H)^+$ 895.2294, found 895.2303

Supplementary Method 3. Synthesis of Albi-1

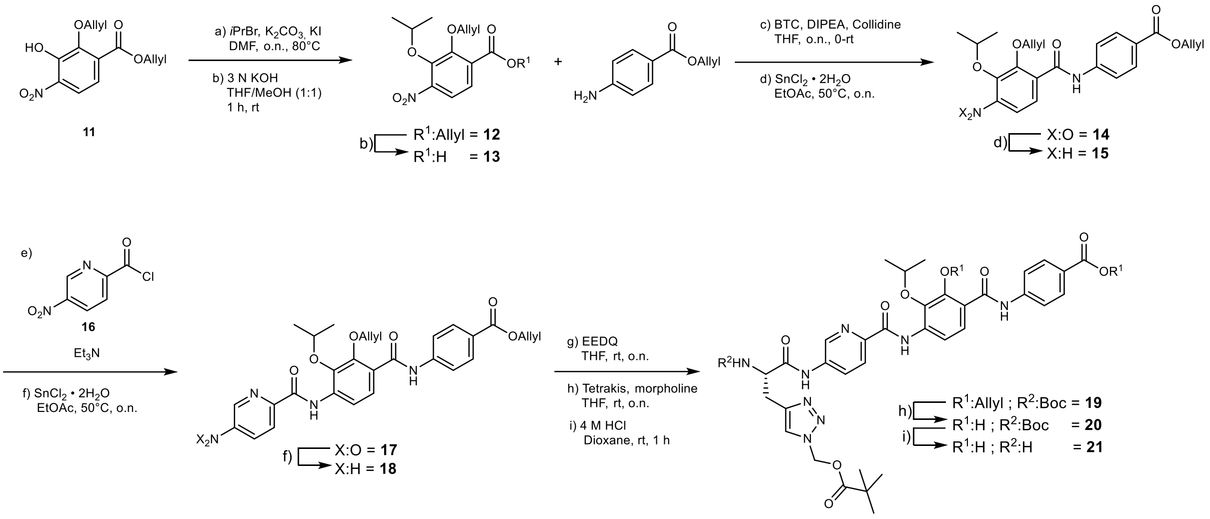

Compound 12

Phenol **11**[2] (1.29 g, 4.62 mmol, 1.00 eq) was dissolved in DMF (40 mL) and successively treated with K_2CO_3 (702 mg, 5.08 mmol, 1.10 eq), *i*PrBr (0.739 mL, 6.01 mmol, 1.30 eq) and KI (7.67 mg, 46.2 µmol, cat.). After stirring at 80 °C for 16 h, the reaction was stopped by the addition of ice water and the aq. solution was extracted with EtOAc (3×). The combined organic phases were washed with brine (5×), dried over anhydrous Na_2SO_4, and concentrated under reduced pressure. Purification of the crude product by column chromatography on silica gel (*n*-hexane/EtOAc, 9:1) afforded the title compound **12** (528 mg, 1.64 mmol, 36%) as a yellow oil. ^{1}H NMR (DMSO-d$_6$, 400 MHz): δ = 7.70 (d, *J*=8.8 Hz, 1 H), 7.57 (d, *J*=8.5 Hz, 1 H), 5.95 - 6.12 (m, 2 H), 5.22 - 5.47 (m, 3 H), 4.81 (dt, *J*=5.6, 1.3 Hz, 2 H), 4.60 - 4.70 (m, 1 H), 4.53 - 4.60 (m, 2 H), 1.13 - 1.27 ppm (m, 6 H) ^{13}C NMR (DMSO-d$_6$, 101 MHz): δ = 164.0, 151.7, 147.8, 143.8, 133.2, 132.0, 130.4, 124.8, 119.1, 118.7, 118.4, 77.2, 74.7, 65.9, 21.9, 21.9 ppm. HRMS (ESI): m/z calculated for $C_{16}H_{20}NO_6$ $(M+H)^+$ 322.1285, found 322.1282.

Compound 13

Compound **13** was synthesized from ester-protected compound **12** (18.4 g, 57.3 mmol, 1.00 eq.) with 3 N KOH in H_2O (36 mL) according to *standard procedure B*. Compound **13** (14.52 mg, 90%) was obtained as yellow oil. ^{1}H NMR (DMSO-d$_6$, 400 MHz): δ = 7.65 (dd, *J*=8.5, 0.8 Hz, 1 H), 7.48 - 7.55 (m, 1 H), 5.97 - 6.11 (m, 1 H), 5.38 (dd, *J*=17.3, 1.3 Hz, 1 H), 5.25 (dd, *J*=10.4, 1.1 Hz, 1 H), 4.60 - 4.72 (m, 1 H), 4.56 (d, *J*=5.5 Hz, 2 H), 1.19 ppm (d, *J*=6.3 Hz, 6 H) ^{13}C NMR (DMSO-d$_6$, 101 MHz):

δ = 166.0, 151.4, 147.3, 143.8, 133.4, 132.1, 124.4, 118.9, 118.1, 77.1, 74.6, 22.0 ppm. HRMS (ESI): m/z calculated for $C_{13}H_{14}NO_6$ $(M-H)^-$ 280.0287, found 280.0826.

Compound 14

Compound **14** was synthesized from benzoic acid **13** (7.84 g, 27.9 mmol, 1.30 eq.), allyl-protected *p*ABA (3.80 g, 21.4 mmol, 1.00 eq.), BTC (2.48 mg, 8.36 mmol, 0.400 eq.), DIPEA (37.4 mL, 215 mmol, 10.0 eq.) and 2,4,6-collidine (29.4 mL, 223 mmol, 10.4 eq.) according to *standard procedure C* - column chromatography: SiO_2, *n*-hexane:EtOAc, 5:1. Compound **14** (5.39 mg, 12.2 mmol, 57%) was obtained as brown oil. ^{1}H NMR (DMSO-d_6, 400 MHz): δ = 10.78 (s, 1 H), 7.97 - 8.04 (m, 2 H), 7.82 - 7.88 (m, 2 H), 7.73 (d, *J*=8.5 Hz, 1 H), 7.46 (d, *J*=8.5 Hz, 1 H), 5.90 - 6.11 (m, 2 H), 5.25 - 5.45 (m, 3 H), 5.18 (dd, *J*=10.5, 1.8 Hz, 1 H), 4.80 (dt, *J*=5.3, 1.5 Hz, 2 H), 4.64 - 4.73 (m, 1 H), 4.58 - 4.63 (m, 2 H), 1.24 ppm (d, *J*=6.3 Hz, 6 H) ^{13}C NMR (DMSO-d_6, 101 MHz): δ = 164.9, 163.9, 149.9, 146.8, 143.4, 143.0, 136.0, 133.1, 132.7, 130.4, 124.6, 123.2, 119.2, 119.1, 118.2, 117.8, 77.2, 74.6, 64.9, 22.0 ppm. HRMS (ESI): m/z calculated for $C_{23}H_{23}N_2O_7$ $(M-H)^-$ 439.1511, found 439.1507.

Compound 15

$SnCl_2{\cdot}2H_2O$ (22.2 g, 98.3 mmol, 6.00 eq.) was added to a solution of the aromatic nitro compound **14** (5.35 g, 12.1 mmol, 1.00 eq.) in EtOAc (180 mL) and the reaction mixture was stirred at 50 °C for 16 h. The reaction was quenched by the addition of saturated aqueous $NaHCO_3$, followed by the separation of the two layers and extraction of the aqueous layer with EtOAc (4×). The combined organic layers were washed with brine, dried over anhydrous Na_2SO_4, and concentrated *in vacuo.* Purification of the crude product by column chromatography on silica gel (*n*-hexane/EtOAc, 3:1) afforded the title compound **15** (3.19 g, 8.50 mmol, 70%) as a yellow oil. ^{1}H NMR (DMSO-d_6, 400 MHz): δ = 10.27 (s, 1 H), 7.95 (d, *J*=8.8 Hz, 2 H), 7.81 (d, *J*=8.8 Hz, 2 H), 7.40 (d, *J*=8.5 Hz, 1 H), 6.56 (d, *J*=8.8 Hz, 1 H), 5.97 - 6.16 (m, 2 H), 5.59 (s, 2 H), 5.35 - 5.51 (m, 2 H), 5.26 (s, 2 H), 4.76 - 4.85 (m, 2 H), 4.62 (d, *J*=5.5 Hz, 2 H), 4.40 - 4.51 (m, 1 H), 1.27 ppm (d, *J*=6.3 Hz, 6 H) ^{13}C NMR (DMSO-d_6 ,101 MHz): δ = 164.9, 163.9, 150.6, 147.8, 143.6, 135.3, 133.5, 132.8, 130.3, 126.0, 123.6, 118.8, 118.1, 117.7, 114.5, 109.9, 74.4, 73.7, 64.8, 22.2 ppm.

Compound 16

Compound **16** was synthesized from 4-nitropicolinic acid (2.1 g, 13 mmol) according to s*tandard procedure H* - r.t., 12 h).

Compound 17

Compound **17** was synthesized from acid chloride **16** (3.05 mg, 16.4 mmol, 2.00 eq.) amine **15** (3.36 g, 8.19 mmol, 1.00 eq.) and Et_3N (3.41 mL, 24.6 mmol, 3.00 eq.) according to s*tandard procedure I* - column chromatography: SiO_2, *n*-hexane/EtOAc, 3:1. Compound **17** (4.53 mg, 8.08 mmol, 99%) was obtained as a yellow powder. ^{1}H NMR (DMSO-d_6, 400 MHz): δ = 10.73 (s, 1 H), 10.56 (s, 1 H), 9.56 (dd, *J*=2.5, 0.5 Hz, 1 H), 8.87 (dd, *J*=8.5, 2.5 Hz, 1 H), 8.41 - 8.46 (m, 1 H), 8.34 (d, *J*=8.5 Hz, 1 H), 7.95 - 8.03 (m, 2 H), 7.87 (d, *J*=8.8 Hz, 2 H), 7.50 (d, *J*=8.5 Hz, 1 H), 5.96 - 6.13 (m, 2 H), 5.35 - 5.48 (m, 2 H), 5.19 - 5.31 (m, 2 H), 4.79 (dt, *J*=5.4, 1.3 Hz, 2 H), 4.65 - 4.73 (m, 1 H), 4.63 (d, *J*=5.8 Hz, 2 H), 1.34 - 1.42 ppm (m, 6 H) ^{13}C NMR (DMSO-d_6, 101 MHz): δ = 164.9, 164.3, 159.6, 152.7, 149.2, 146.1, 144.8, 144.3, 143.4, 139.8, 134.7, 134.1, 133.5, 133.1, 132.7, 130.3, 126.6, 125.4, 124.5, 124.2, 123.0, 119.0, 118.0, 117.8, 114.1, 76.5, 74.3, 64.8, 62.0, 22.3 ppm. HRMS (ESI): m/z calculated for $C_{29}H_{29}N_4O_8$ $(M+H)^+$ 561.1980, found 561.1983.

Compound 18

$SnCl_2 \times 2\ H_2O$ (9.04 g, 40.1 mmol, 5.00 eq.) was added to a solution of the aromatic nitro compound **17** (4.49 g, 8.02 mmol, 1.00 eq.) in EtOAc (120 mL) and the reaction mixture was stirred at 50 °C for 16 h. The reaction was quenched by the addition of saturated aqueous $NaHCO_3$, followed by the separation of the two layers and extraction of the aqueous layer with EtOAc (4×). The combined organic layers were washed with brine, dried over anhydrous Na_2SO_4, and concentrated *in vacuo.* Purification of the crude product by column chromatography on silica gel (*n*-hexane/EtOAc, 1:2) afforded the title compound **18** (4.04 g, 7.61 mmol, 95%) as an orange solid. ^{1}H NMR (DMSO-d_6, 400 MHz): δ = 10.53 (d, *J*=17.3 Hz, 2 H), 8.31 - 8.40 (m, 1 H), 7.95 - 8.07 (m, 3 H), 7.87 (d, *J*=8.5 Hz, 3 H), 7.43 - 7.52 (m, 1 H), 6.24 (br. s., 1 H), 6.04 (dddt, *J*=17.2, 10.6, 9.5, 5.3 Hz, 2 H), 5.35 - 5.47 (m, 2 H), 5.16 - 5.32 (m, 2 H), 4.80 (dt, *J*=5.3, 1.5 Hz, 2 H), 4.57 - 4.67 (m, 3 H), 1.35 ppm (d, *J*=6.3 Hz, 6 H) ^{13}C NMR (DMSO-d_6, 101 MHz): δ = 164.9, 164.4, 162.1, 149.3, 148.4, 143.5, 139.1, 136.2, 136.1, 134.5, 133.5, 132.7, 130.3, 124.8, 124.6, 124.1, 123.5, 119.3,

119.0, 118.0, 117.8, 113.5, 76.2, 74.2, 64.8, 22.3 ppm. HRMS (ESI): m/z calculated for $C_{29}H_{31}N_4O_6$ $(M+H)^+$ 531.2238, found 531.2240.

Compound 19

Compound **19** was synthesized from Boc/POM-protected azahistidine (1.32 g, 3.55 mmol, 1.2 eq.), tripeptide **18** (1.57 g, 2.96 mmol, 1.00 eq.) and EEDQ (1.46 mg, 5.92 mmol, 2.00 eq.) according to s*tandard procedure D* - column chromatography: SiO_2, 1-5% MeOH in DCM. Compound **19** (1.15 mg, 1.30 mmol, 44%) was obtained as a yellow solid. ^{1}H NMR (DMSO-d$_6$,400 MHz): δ = 10.73 (s, 1 H), 10.68 (s, 1 H), 10.54 (s, 1 H), 8.97 (d, *J*=2.3 Hz, 1 H), 8.36 (d, *J*=8.5 Hz, 1 H), 8.24 - 8.32 (m, 1 H), 8.19 (d, *J*=8.5 Hz, 1 H), 7.94 - 8.05 (m, 3 H), 7.88 (d, *J*=8.8 Hz, 2 H), 7.49 (d, *J*=8.5 Hz, 1 H), 7.31 (d, *J*=7.8 Hz, 1 H), 6.29 (s, 2 H), 5.94 - 6.14 (m, 2 H), 5.34 - 5.48 (m, 2 H), 5.16 - 5.32 (m, 2 H), 4.79 (d, *J*=5.3 Hz, 2 H), 4.59 - 4.71 (m, 3 H), 4.38 - 4.50 (m, 1 H), 2.96 - 3.24 (m, 2 H), 1.37 (s, 9 H), 1.07 ppm (s, 6 H) ^{13}C NMR (DMSO-d$_6$,101 MHz): δ = 176.4, 171.3, 164.9, 164.4, 161.1, 155.3, 149.2, 143.4, 143.3, 143.2, 139.4, 139.4, 138.7, 135.5, 133.5, 132.7, 130.3, 127.1, 125.7, 124.6, 124.2, 124.1, 122.8, 119.0, 118.0, 117.8, 113.8, 78.4, 76.3, 74.3, 69.8, 64.8, 54.9, 38.1, 28.1, 27.6, 26.4, 22.3, 22.3 ppm. HRMS (ESI): m/z calculated for $C_{45}H_{55}N_8O_{11}$ $(M+H)^+$ 883.3985, found 883.3998.

Compound 20

Compound **20** was synthesized from allyl-protected tetrapeptide **19** (1.11 g, 1.25 mmol, 1.00 eq.), $Pd(PPh_3)_4$ (434 mg, 376 µmol, 0.3 eq.) and morpholine (2.16 mL, 25.1 mmol, 20.0 eq.) according to *standard procedure F* - column chromatography: SiO_2, 10% MeOH in DCM. Compound **20** (851 mg, 2.09 mmol, 84%) was obtained as a yellow solid. ^{1}H NMR (DMSO-d$_6$, 400 MHz): δ = 10.86 (s, 1 H), 10.73 (s, 1 H), 8.98 (d, *J*=2.3 Hz, 1 H), 8.30 (dd, *J*=8.7, 2.1 Hz, 1 H), 8.18 (d, *J*=8.8 Hz, 1 H), 8.05 - 8.12 (m, 1 H), 8.01 (s, 1 H), 7.92 - 7.99 (m, 3 H), 7.84 - 7.91 (m, 2 H), 7.30 (d, *J*=7.8 Hz, 1 H), 6.28 (s, 2 H), 4.65 - 4.75 (m, 1 H), 4.39 - 4.51 (m, 1 H), 2.98 - 3.22 (m, 2 H), 1.36 (s, 9 H), 1.07 ppm (s, 6 H) ^{13}C NMR (DMSO-d$_6$, 101 MHz): δ = 176.4, 171.3, 168.6, 166.9, 161.2, 155.3, 143.3, 142.1, 139.4, 138.8, 136.6, 134.0, 130.1, 127.1, 126.1, 124.2, 123.6, 122.8, 120.7, 111.7, 78.4, 74.6, 69.8, 68.5, 55.8, 55.0, 38.1, 32.1, 29.6,

29.0, 28.1, 27.6, 26.4, 22.3, 22.3 ppm. HRMS (ESI): m/z calculated for $C_{39}H_{47}N_8O_{11}$ $(M+H)^+$ 803.3359, found 803.3399.

Compound 21

Compound **21** was synthesized from Boc-protected tetrapeptide **20** (820 mg, 1.02 mmol, 1.00 eq.) with 4 N HCl in dioxane (4 mL) according to *standard procedure E*. Compound **21** (750 mg, 1.00 mmol, quant.) was obtained as light-yellow solid. ^{1}H NMR (DMSO-d_6, 400 MHz): δ = 12.53 (s, 1 H), 11.87 (s, 1 H), 10.75 (s, 1 H), 10.71 (s, 1 H), 8.98 (d, *J*=2.3 Hz, 1 H), 8.70 (br. s., 2 H), 8.31 (d, *J*=2.5 Hz, 1 H), 8.16 - 8.24 (m, 2 H), 8.07 - 8.13 (m, 1 H), 8.00 - 8.05 (m, 1 H), 7.95 (s, 2 H), 7.85 - 7.92 (m, 2 H), 6.27 (s, 2 H), 4.68 (s, 1 H), 4.50 (br. s., 1 H), 3.40 (d, *J*=6.0 Hz, 2 H), 1.34 (dd, *J*=6.0, 2.3 Hz, 6 H), 1.04 ppm (s, 9 H) ^{13}C NMR (DMSO-d_6, 101 MHz): δ = 176.3, 168.7, 167.4, 166.8, 161.1, 154.1, 143.9, 141.9, 140.7, 139.4, 138.1, 136.6, 133.9, 130.1, 127.4, 126.3, 125.0, 123.7, 122.9, 120.8, 111.6, 108.3, 74.7, 69.8, 52.6, 38.1, 26.4, 22.3, 22.3 ppm.

Final coupling of Albi-1

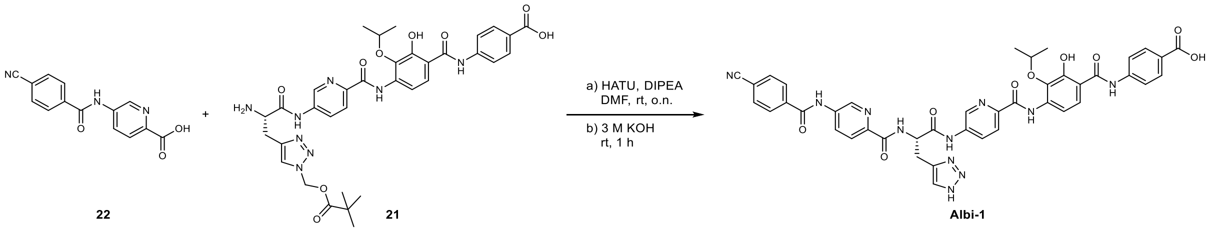

HATU (64.9 mg, 171 µmol, 1.20 eq.) was added to a solution of AB building block **22**[3] (45.6 mg, 171 µmol, 1.20 eq.) in anhydrous DMF (2 mL) and the resulting solution was stirred at r.t for 45 min. A solution of tetrapeptide **21** (100 mg, 142 µmol, 1.00 eq.) and DIPEA (0.149 mL) in anhydrous DMF (1 mL) was added dropwise and the reaction mixture was stirred at r.t. for 16 h. All volatiles were removed *in vacuo* and the residue was taken up in a mixture of THF (1 mL) and MeOH (1 mL), and 3 N $KOH_{(aq.)}$ (1 mL) was added dropwise. After 45 min of stirring, 3 N $HCl_{(aq.)}$ (1.1 mL) was added and the resulting suspension was evaporated under reduced pressure. The crude material was dissolved in DMSO, centrifuged, and the supernatant purified by HPLC (PLRP-S column, CH_3CN in H_2O). The title compound **Albi-1** (11 mg, 9% over two steps) was obtained as a colorless solid. ^{1}H NMR (DMSO-d_6, 400 MHz): δ = 12.46 (s, 1 H), 11.01

(s, 1 H), 10.90 (s, 1 H), 10.75 (s, 1 H), 10.62 (s, 1 H), 9.05 (d, *J*=2.3 Hz, 1 H), 8.98 (d, *J*=2.3 Hz, 1 H), 8.89 (d, *J*=7.3 Hz, 1 H), 8.42 (dd, *J*=8.7, 2.4 Hz, 1 H), 8.25 - 8.33 (m, 1 H), 8.13 - 8.22 (m, 3 H), 7.89 - 8.00 (m, 3 H), 7.85 (d, *J*=8.8 Hz, 2 H), 7.54 - 7.67 (m, 1 H), 4.96 - 5.07 (m, 1 H), 4.61 - 4.72 (m, 1 H), 1.34 ppm (dd, *J*=5.8, 4.3 Hz, 6 H) H,C-HSQC NMR (DMSO-d_6, 101 MHz): δ = 140.9, 140.1, 133.2, 130.7, 129.3, 128.4, 128.0, 124.1, 123.3, 123.1, 121.3, 108.7, 75.3, 29.2, 22.7 ppm. HRMS (ESI): m/z calculated for $C_{42}H_{36}N_{11}O_9$ $(M+H)^+$ 838.2692, found 838.2702.

Supplementary Method 4. Synthesis of Albi-2

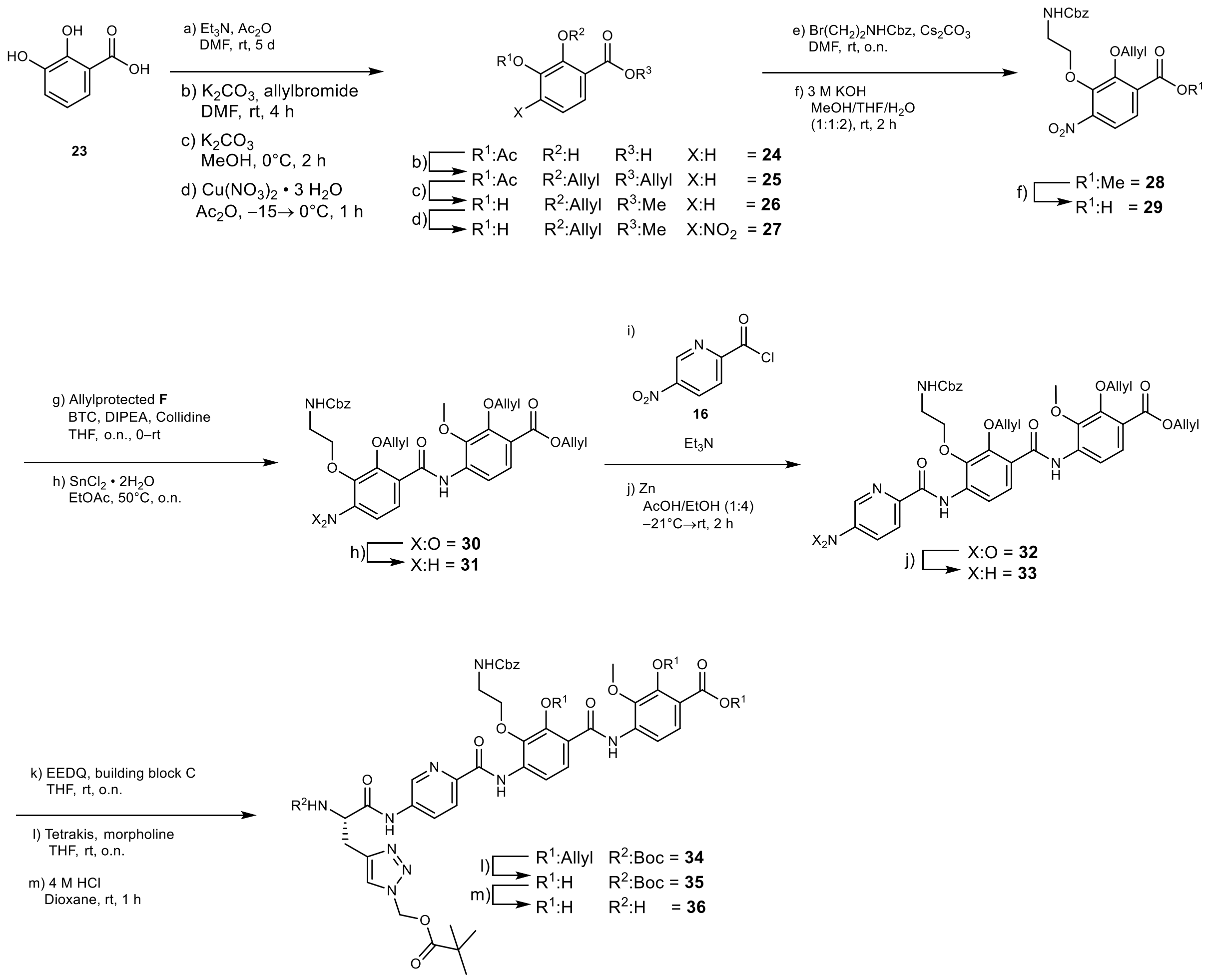

Compound 24

2,3-Dihydroxybenzoic acid (**23**) (20.0 g, 130 mmol, 1.00 eq.) was dissolved in DMF (200 mL) and cooled down to 0 °C. Et_3N (36.2 mL, 260 mmol, 2.00 eq) was added dropwise followed by acetic anhydride (12.9 mL, 136 mmol, 1.05 eq). The reaction mixture was stirred at r.t. for 5 d. After removing the solvent under reduced pressure, the residue was taken up in EtOAc and washed with brine (3x). The organic phase was dried over anhydrous Na_2SO_4 and concentrated *in vacuo* to afford the pure title compound **24** (18.4 g, 93.8 mmol, 72%) as a light-brown solid. ^1H NMR (DMSO-d_6, 500 MHz): δ = 7.70 (dd, *J*=8.1, 1.2 Hz, 1 H), 7.35 (dd, *J*=7.9, 1.1 Hz, 1 H), 6.93 (t, *J*=7.9 Hz, 1 H), 2.28 ppm (s, 3 H) ^{13}C NMR (DMSO-d_6, 126 MHz): δ = 172.2, 168.9, 153.7, 139.2, 129.1, 128.0, 119.0, 115.0, 20.8 ppm.

Compound 25

Benzoic acid **24** (8.50 g, 43.3 mmol, 1.00 eq.) was dissolves in DMF (250 mL) and treated with K_2CO_3 (18.0 g, 130 mmol, 3.00 eq.). The solution was cooled down to 0 °C and allyl bromide (7.49 mL, 86.7 mmol, 2.00 eq.) was added dropwise. After stirring at r.t. for 16 h, the mixture was extracted with EtOAc (3×) and washed with brine (2×). The combined organic layers were dried over anhydrous Na_2SO_4 and concentrated under reduced pressure. Purification of the crude product by column chromatography on silica gel (*n*-hexane/EtOAc,8:1) afforded the title compound **25** (11.0 g, 39.8 mmol, 92%) as a colorless oil. ^{1}H NMR (DMSO-d$_6$, 500 MHz): δ = 7.64 (dd, *J*=7.9, 1.7 Hz, 1 H), 7.42 (dd, *J*=8.0, 1.8 Hz, 1 H), 7.26 (t, *J*=7.9 Hz, 1 H), 6.01 (dd, *J*=17.2, 8.6 Hz, 2 H), 6.03 (dd, *J*=17.2, 8.7 Hz, 2 H), 5.42 (dd, *J*=17.2, 1.7 Hz, 1 H), 5.19 - 5.38 (m, 4 H), 4.80 (dt, *J*=5.6, 1.5 Hz, 2 H), 4.45 (dt, *J*=5.6, 1.4 Hz, 2 H), 2.30 ppm (s, 3 H) ^{13}C NMR (DMSO-d$_6$, 126 MHz): δ = 169.1, 165.1, 150.3, 145.0, 134.2, 132.8, 128.5, 128.1, 126.8, 124.7, 118.8, 118.1, 75.5, 65.9, 21.0 ppm.

Compound 26

To a solution of the allyl ester **25** (10.0 g, 36.2 mmol, 1.00 eq.) in MeOH (250 mL) was added K_2CO_3 (10.0 g, 72.4 mmol, 2 eq.) and the reaction mixture was stirred at r.t. for 2 d. After removing the solvent under reduced pressure, the residue was taken up in EtOAc, washed with 1 N $HCl_{(aq.)}$ (2×), brine (3×), and dried over anhydrous Na_2SO_4. The solvent was removed under reduced pressure and the crude product was purified by column chromatography on silica gel (*n*-hexane/EtOAc, 8:1) to afford the title compound **26** (7.32 g, 31.3 mmol, 86%) as a yellow oil. ^{1}H NMR (DMSO-d$_6$, 500 MHz): δ = 9.69 (s, 1 H), 7.04 - 7.09 (m, 2 H), 6.95 - 7.02 (m, 1 H), 5.98 - 6.14 (m, 1 H), 5.31 (dd, *J*=17.3, 1.6 Hz, 1 H), 5.18 (dd, *J*=10.5, 1.1 Hz, 1 H), 4.50 (d, *J*=5.6 Hz, 2 H), 3.79 ppm (s, 3 H) ^{13}C NMR (DMSO-d$_6$, 126 MHz): δ = 167.0, 151.6, 145.8, 135.0, 126.9, 124.3, 120.5, 117.6, 74.1, 60.2, 52.3, 21.1, 14.5 ppm. HRMS (ESI): *m/z* calculated for $C_{11}H_{13}O_4$ $(M+H)^+$ 209.0808, found 209.0806.

Compound 27

A solution of compound **26** (7.1 g, 34.1 mmol, 1.00 eq.) in acetic anhydride (300 mL) was cooled down to 0 °C and $Cu(NO_3)_2{\cdot}3H_2O$ (4.94 g, 20.5 mmol, 0.600 eq.) was added in several portions. After stirring at 0 °C for 4 h, the reaction mixture was partitioned between ice water and EtOAc. The aq. phase was extracted with EtOAc

(2×) and the combined organic phases were washed with 3 N $HCl_{(aq.)}$, dried over Na_2SO_4, and concentrated under reduced pressure. Purification of the crude product by column chromatography on silica gel (*n*-hexane/EtOAc, 9:1) gave the title compound **27** (7.40 g, 29.2 mmol, 86%) as a yellow solid. ^{1}H NMR (DMSO-d_6, 400 MHz): δ = 10.82 (br. s., 1 H), 7.71 (d, *J*=8.8 Hz, 1 H), 7.21 (d, *J*=8.8 Hz, 1 H), 5.98 - 6.12 (m, 1 H), 5.27 - 5.38 (m, 1 H), 5.15 - 5.27 (m, 1 H), 4.53 (dt, *J*=6.0, 1.3 Hz, 2 H), 3.85 ppm (s, 3 H) ^{13}C NMR (DMSO-d_6, 101 MHz): δ= 165.1, 147.7, 146.6, 139.5, 133.5, 130.5, 119.5, 119.1, 118.5, 74.9, 52.5 ppm. HRMS (ESI): *m/z* calculated for $C_{11}H_{12}NO_6$ $(M+H)^+$ 254.0659, found 254.06.

Compound 28

Phenol **27** (1.50 g, 5.92 mmol, 1.00 eq.) was dissolved in DMF (30 mL) and successively treated with Cs_2CO_3 (4.09 g, mmol, 5.00 eq.) and Cbz-protected bromoethane amine (3.21 g, 12.4 mmol, 2.10 eq.). After stirring at 60°C for 16 h, the reaction was stopped by the addition of ice water and the aq. solution was extracted with EtOAc (3×). The combined organic phases were washed with brine (2×), dried over anhydrous Na_2SO_4, and concentrated under reduced pressure. Purification of the crude product by column chromatography on silica gel (*n*-hexane/EtOAc, 9:1) afforded the title compound **28** (2.12 g, 4.93 mmol, 83%) as a yellow oil. ^{1}H NMR (DMSO-d_6, 500 MHz): δ = 7.72 (d, *J*=8.5 Hz, 1 H), 7.57 (s, 1 H), 7.44 (t, *J*=4.5 Hz, 1 H), 7.33 - 7.40 (m, 5 H), 5.96 - 6.10 (m, 1 H), 5.35 (dd, *J*=17.2, 1.4 Hz, 1 H), 5.22 (d, *J*=10.5 Hz, 1 H), 5.04 (s, 2 H), 4.55 (d, *J*=6.0 Hz, 2 H), 4.14 (t, *J*=5.6 Hz, 2 H), 3.86 (s, 3 H), 3.35 - 3.42 ppm (m, 6 H) ^{13}C NMR (DMSO-d_6, 126 MHz): δ = 165.2, 156.7, 152.2, 147.0, 146.0, 137.6, 133.6, 131.3, 129.3, 129.1, 128.8, 128.2, 128.2, 125.6, 119.8, 119.3, 75.7, 73.9, 65.8, 53.2 ppm. HRMS (ESI): *m/z* calculated for $C_{21}H_{23}N_2O_8$ $(M+H)^+$ 431.1449, found 431.1436.

Compound 29

To a solution of the benzoic acid ester **28** in a 1:1 mixture of THF and MeOH was slowly added LiOH (419 mg, 9.99 mmol, 10.0 eq.) After complete conversion of the starting material (TLC monitoring), the reaction mixture was acidified to pH ~2 by the addition of 3 N $HCl_{(aq.)}$. The precipitate was isolated by filtration through a sintered funnel, washed with water and dried under high vacuum to obtain the analytically pure product **29** (340 mg, 0.816 mmol, 82%). ^{1}H NMR (DMSO-d_6, 500 MHz): δ = 7.69 (d,

J=8.5 Hz, 1 H), 7.54 (d, *J*=8.5 Hz, 1 H), 7.44 (t, *J*=5.5 Hz, 1 H), 7.29 - 7.39 (m, 5 H), 5.98 - 6.08 (m, 1 H), 5.35 (d, *J*=17.1 Hz, 1 H), 5.21 (d, *J*=10.2 Hz, 1 H), 5.04 (s, 2 H), 4.56 (d, *J*=5.8 Hz, 2 H), 4.13 (t, *J*=5.6 Hz, 2 H), 3.35 - 3.43 ppm (m, 2 H) ^{13}C NMR (DMSO-d_6, 126 MHz): δ = 166.4, 156.7, 151.9, 146.5, 145.9, 137.6, 133.8, 133.1, 128.8, 128.2, 128.2, 125.3, 119.7, 119.1, 75.6, 73.8, 65.8 ppm. HRMS (ESI): *m/z* calculated for $C_{20}H_{21}N_2O_8$ $(M+H)^+$ 417.1292, found 417.1285.

Compound 30

Compound **30** was synthesized from benzoic acid **29** (780 mg, 1.87 mmol, 1.00 eq.), allyl-protected F building block (1.14 g, 2.06 mmol, 1.10 eq.), BTC (278 mg, 0.937 mmol, 0.500 eq.), DIPEA (2.61 mL, 15.0 mmol, 8.00 eq.) and 2,4,6-collidine (1.49 mL, 11.2 mmol, 6.00 eq.) according to *standard procedure C* - column chromatography: SiO_2, *n*-hexane:EtOAc, 6:1. Compound **30** (834 mg, 1.26 mmol, 67%) was obtained as an orange solid. ^{1}H NMR (DMSO-d_6, 400 MHz): δ = 10.41 (s, 1 H), 8.19 (d, *J*=8.8 Hz, 1 H), 7.76 - 7.82 (m, 1 H), 7.70 - 7.75 (m, 1 H), 7.57 (d, *J*=8.8 Hz, 1 H), 7.50 (s, 1 H), 7.28 - 7.40 (m, 5 H), 6.85 (s, 1 H), 5.91 - 6.15 (m, 3 H), 5.18 - 5.46 (m, 6 H), 5.05 (s, 2 H), 4.78 (dt, *J*=5.5, 1.4 Hz, 1 H), 4.72 (d, *J*=6.3 Hz, 1 H), 4.53 (d, *J*=5.8 Hz, 1 H), 4.15 - 4.24 (m, 2 H), 3.87 (s, 2 H), 3.42 ppm (d, *J*=5.8 Hz, 2 H) ^{13}C NMR (DMSO-d_6, 101 MHz): δ = 170.3, 164.4, 162.3, 156.7, 156.2, 151.2, 150.3, 147.0, 146.2, 145.0, 143.3, 137.1, 135.9, 133.9, 133.5, 132.6, 132.3, 128.3, 127.7, 127.7, 126.0, 124.9, 121.1, 120.8, 120.1, 119.6, 118.1, 117.8, 115.6, 75.5, 74.5, 73.3, 65.4, 65.1, 61.1, 59.7, 50.2, 23.7, 20.7, 20.2, 14.0 ppm. HRMS (ESI): *m/z* calculated for $C_{34}H_{36}N_3O_{11}$ $(M+H)^+$ 662.2344, found 662.2339.

Compound 31

Compound **31** was synthesized from nitro compound **30** (580 mg, 877 µmol, 1.00 eq.) and Zn (5.16 g, 78.9 mmol, 90.0 eq.) according to *standard procedure A*. The title compound **31** (550 mg, 871 mmol, 99%) was obtained as yellow solid. ^{1}H NMR (DMSO-d_6, 400 MHz): δ = 10.60 (s, 1 H), 8.34 (d, *J*=8.8 Hz, 1 H), 7.48 - 7.68 (m, 3 H), 7.28 - 7.41 (m, 5 H), 6.58 (d, *J*=8.5 Hz, 1 H), 5.95 - 6.16 (m, 3 H), 5.88 (s, 2 H), 5.19 - 5.45 (m, 6 H), 5.06 (s, 2 H), 4.72 - 4.80 (m, 4 H), 4.53 (d, *J*=5.8 Hz, 2 H), 3.94 (t, *J*=5.4 Hz, 2 H), 3.88 (s, 2 H), 3.44 (d, *J*=5.5 Hz, 2 H), 3.32 (s, 1 H), 1.99 ppm (s, 2 H) ^{13}C NMR (DMSO-d_6, 101 MHz): δ = 170.3, 164.4, 163.0, 156.3, 151.1, 150.3, 147.4, 142.0, 137.2, 137.1, 136.5, 134.0, 132.8, 132.7, 128.3, 127.7, 127.7, 127.1, 126.3, 120.8,

119.8, 119.3, 118.0, 117.7, 114.3, 113.0, 110.2, 74.7, 74.5, 71.4, 65.3, 65.0, 60.8, 59.7, 20.7, 14.0 ppm. HRMS (ESI): *m/z* calculated for $C_{34}H_{38}N_3O_9$ $(M+H)^+$ 632.2603, found 632.2610.

Compound 32

Compound **32** was synthesized from acid chloride **16** (400 mg, 2.14 mmol, 2.26 eq.), amine **31** (600 mg, 0.950 mmol, 1.00 eq.) and Et_3N (0.66 mL, 4.8 mmol, 5.00 eq.) according to s*tandard procedure I* - column chromatography: SiO_2, *n*-hexane/EtOAc, 7:1. Compound **32** (312 mg, 0.399 mmol, 42%) was obtained as orange solid. ^{1}H NMR (DMSO-d$_6$, 400 MHz): δ = 10.59 (d, *J*=12.5 Hz, 1 H), 9.08 (d, *J*=2.5 Hz, 1 H), 8.67 (dd, *J*=8.5, 2.5 Hz, 1 H), 8.40 (d, *J*=8.5 Hz, 1 H), 8.26 (d, *J*=8.8 Hz, 2 H), 7.79 (d, *J*=8.5 Hz, 1 H), 7.57 (d, *J*=8.8 Hz, 1 H), 7.20 - 7.38 (m, 6 H), 5.87 - 6.16 (m, 3 H), 5.13 - 5.50 (m, 6 H), 4.90 - 5.02 (m, 2 H), 4.75 - 4.86 (m, 3 H), 4.66 (d, *J*=6.5 Hz, 1 H), 4.51 - 4.58 ppm (m, 2 H) ^{13}C NMR (DMSO-d$_6$, 101 MHz): δ = 170.3, 168.5, 164.3, 147.5, 145.0, 143.6, 136.9, 132.6, 128.3, 127.7, 118.1, 106.0, 74.5, 65.3, 61.0, 59.7, 20.7, 14.1 ppm. HRMS (ESI): *m/z* calculated for $C_{40}H_{40}N_5O_{12}$ $(M+H)^+$ 782.2668, found 782.2691.

Compound 33

Compound **33** was synthesized from nitro compound **32** (700 mg, 895 µmol, 1.00 eq.) and Zn (4.39 g, 67.2 mmol, 75.0 eq.) according to *standard procedure A*. The title compound **33** (642 mg, 854 mmol, 95%) was obtained as yellow solid. ^{1}H NMR (DMSO-d$_6$, 400 MHz): δ = 10.62 (s, 1 H), 10.55 (s, 1 H), 8.41 (s, 1 H), 8.33 (d, *J*=8.8 Hz, 1 H), 7.47 - 7.63 (m, 2 H), 7.16 - 7.41 (m, 5 H), 6.27 (s, 1 H), 5.93 - 6.17 (m, 3 H), 5.17 - 5.52 (m, 4 H), 4.72 - 4.87 (m, 2 H), 4.54 (d, *J*=5.8 Hz, 1 H), 4.16 (s, 1 H), 3.84 - 3.99 ppm (m, 2 H). HRMS (ESI): m/z calculated for $C_{40}H_{42}N_5O_{10}$ $(M+H)^+$ 752.2926, found 752.2925.

Compound 34

Compound **34** was synthesized from Boc/POM-protected azahistidine (527 mg, 1.42 mmol, 1.75 eq.), tripeptide **33** (611 mg, 813 mmol, 1.00 eq.) and EEDQ (352 mg, 1.42 mmol, 1.75 eq.) according to s*tandard procedure D* - column chromatography: SiO_2, 1-5% MeOH in DCM. Compound **34** (352 mg, 0.319 mmol, 39%) was obtained as a yellow solid. ^{1}H NMR (DMSO-d$_6$, 500 MHz): δ = 10.75 (br. s., 1 H), 10.69 (s, 1 H), 10.61 (s, 1 H), 8.99 (br. s., 1 H), 8.41 (d, *J*=8.9 Hz, 1 H), 8.33 (d, *J*=8.9 Hz, 1 H), 8.18

(d, *J*=8.5 Hz, 1 H), 8.00 (s, 1 H), 7.88 (d, *J*=9.0 Hz, 1 H), 7.58 (d, *J*=8.8 Hz, 1 H), 7.52 (t, *J*=5.3 Hz, 1 H), 7.23 - 7.34 (m, 6 H), 6.29 (s, 2 H), 5.98 - 6.15 (m, 3 H), 5.20 - 5.47 (m, 7 H), 4.97 (s, 2 H), 4.75 - 4.86 (m, 4 H), 4.55 (d, *J*=5.8 Hz, 2 H), 4.40 - 4.50 (m, 1 H), 4.22 (t, *J*=5.2 Hz, 2 H), 3.92 (s, 3 H), 3.54 (d, *J*=5.3 Hz, 2 H), 2.96 - 3.19 (m, 2 H), 1.09 ppm (s, 9 H) ^{13}C NMR (DMSO-d_6 ,126 MHz): δ = 176.9, 170.8, 164.9, 162.0, 151.6, 143.0, 137.5, 137.0, 134.4, 133.1, 132.9, 128.7, 128.1, 127.6, 126.8, 122.3, 121.1, 120.7, 118.6, 118.3, 115.3, 78.8, 75.6, 75.0, 70.3, 65.8, 65.6, 61.5, 60.2, 28.6, 26.9, 21.2, 14.6 ppm. HRMS (ESI): m/z calculated for $C_{56}H_{66}N_9O_{15}$ $(M+H)^+$ 1104.4673, found 1104.4675.

Compound 35

Compound **35** was synthesized from allyl-protected tetrapeptide **34** (190 mg, 172 µmol, 1.00 eq.), $Pd(PPh_3)_4$ (79.5 mg, 68.8 µmol, 0.400 eq.) and morpholine (297 µl, 3.44 mmol, 20.0 eq.) according to *standard procedure F* - column chromatography: SiO_2, 1-10% MeOH in DCM. Compound **35** (112 mg, 114 mmol, 66%) was obtained as a yellow solid. ^{1}H NMR (DMSO-d_6, 400 MHz): δ = 11.77 (br. s., 1 H), 10.82 (br. s., 2 H), 10.55 (s, 1 H), 8.98 (s, 1 H), 8.31 (d, *J*=8.8 Hz, 1 H), 8.16 (d, *J*=8.8 Hz, 1 H), 8.09 (br. s., 1 H), 8.00 (s, 1 H), 7.87 (d, *J*=9.3 Hz, 1 H), 7.53 - 7.75 (m, 2 H), 7.49 (d, *J*=8.8 Hz, 2 H), 7.24 - 7.35 (m, 6 H), 6.28 (s, 2 H), 4.99 (s, 2 H), 4.44 (br. s., 1 H), 4.08 (br. s., 2 H), 3.87 (s, 3 H), 3.54 (d, *J*=4.8 Hz, 2 H), 3.51 (s, 6 H), 1.36 (s, 9 H), 1.08 ppm (s, 9 H). HRMS (ESI): m/z calculated for $C_{47}H_{54}N_9O_{15}$ $(M+H)^+$ 984.3734, found 984.3757.

Compound 36

Compound **36** was synthesized from Boc-protected tetrapeptide **35** (109 mg, 111 µmol, 1.00 eq.) with 4 N HCl in dioxane (4 mL) according to *standard procedure E*. Compound **36** (97 mg, 110 µmol, quant.) was obtained as light-yellow solid. HRMS (ESI): m/z calculated for $C_{42}H_{46}N_9O_{13}$ $(M+H)^+$ 884.3210, found 884.3212.

Final coupling of Albi-2

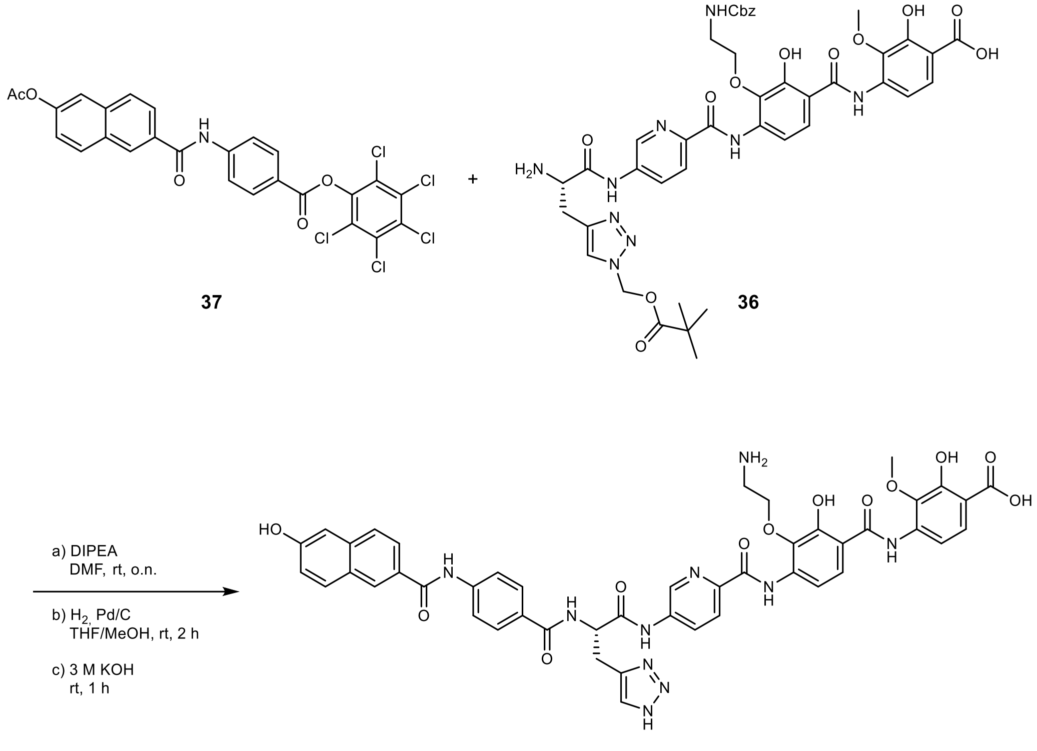

Albi-2

Albi-2 was synthesized from PCP-ester **37** (78.7 mg, 136 µmol, 1.20 eq.), tetrapeptide **36** (97.0 mg, 110 µmol, 1.00 eq.) and Et_3N (115 µl, 659 mmol, 6.00 eq.) according to *standard procedure G.* Final derivative **Albi-2** (4.6 mg, 5% over three steps) was obtained as a colorless solid. ^{1}H NMR (DMSO-d_6, 700 MHz): δ = 10.85 (br. s., 1 H), 10.53 (br. s., 1 H), 10.44 (br. s., 1 H), 10.13 (br. s., 1 H), 9.47 (br. s., 1 H), 9.03 (br. s., 1 H), 8.82 (br. s., 1 H), 8.77 (d, *J*=7.9 Hz, 1 H), 8.44 - 8.57 (m, 2 H), 8.30 (d, *J*=8.3 Hz, 2 H), 8.16 - 8.25 (m, 2 H), 7.94 (br. s., 7 H), 7.78 - 7.88 (m, 3 H), 7.16 - 7.25 (m, 3 H), 4.94 (br. s., 1 H), 4.17 (br. s., 2 H), 3.95 (br. s., 3 H), 3.90 ppm (br. s., 2 H) ^{13}C NMR (DMSO-d_6, 126 MHz): δ =145.2, 139.8, 133.5, 133.3, 132.1, 131.3, 131.0, 129.0, 128.6, 128.5, 127.8, 126.7, 126.5, 126.0, 125.9, 125.8 125.1, 125.0, 123.3, 120.0, 119.8, 119.6, 119.5, 119.5, 109.0, 70.1, 69.7, 64.1, 63.1, 60.4, 60.3, 60.2, 60.2, 54.8, 53.9, 53.5, 52.4, 51.5, 44.1, 42.8, 41.1, 40.2, 40.2, 40.1, 40.0, 39.8, 39.6, 37.9, 34.1, 33.9, 31.7, 30.8, 30.7, 29.3, 29.2, 28.5, 28.4, 27.9, 27.7, 27.7, 27.1, 26.5, 26.4, 24.9, 24.6, 23.0, 22.5, 21.9, 19.8, 18.5, 14.4 ppm. HRMS (ESI): m/z calculated for $C_{46}H_{41}N_{10}O_{12}$ $(M+H)^+$ 925.2900, found 925.2904.

Supplementary Method 5. Synthesis of Albi-3

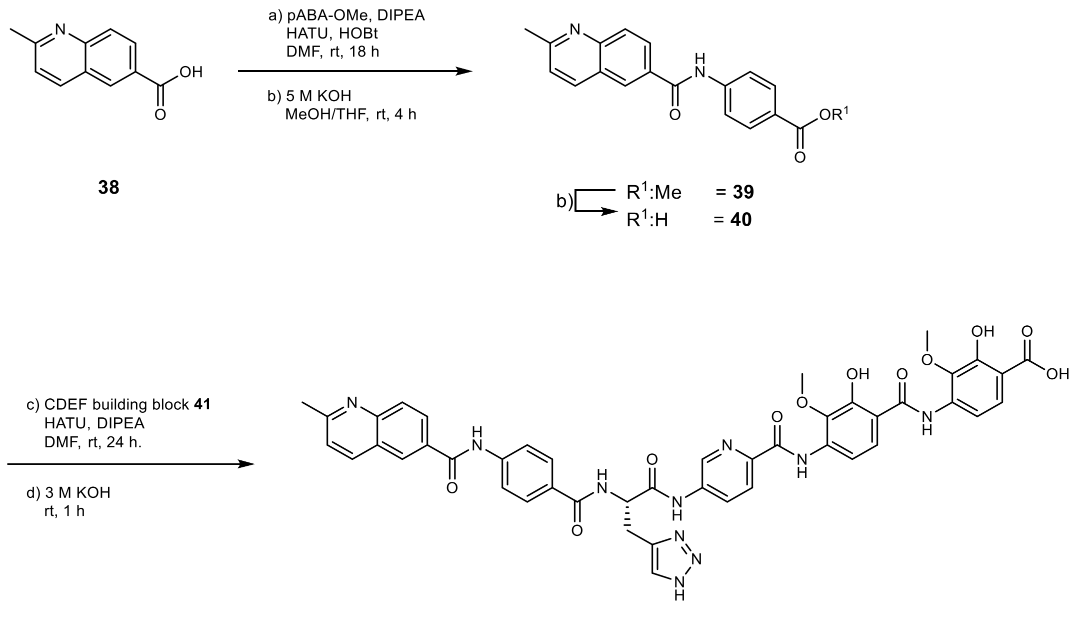

Albi-3

Compound 39

2-Methylquinoline-6-carboxylic acid (**38**, 100 mg, 534 µmol, 1.00 eq.) was dissolved in DMF (1 mL) and HOBt (36.1 mg, 267 µmol, 0.50 eq.), HATU (304 mg, 801 µmol, 1.50 eq.) and DIPEA (279 µL, 1.60 mmol, 3.00 eq.) were added and the reaction mixture was stirred for 1 h at room temperature. 4-Methyl-aminobenzoate (121 mg, 801 µmol, 1.50 eq.) was added and the reaction mixture was stirred 18 h at room temperature. The reaction mixture was diluted by EtOAc (30 mL) and the organic layer was washed by a saturated aqueous $NaHCO_3$ solution (3 × 20 mL) and by a saturated aqueous NaCl solution (1 × 20 mL). The organic layer was dried over $MgSO_4$, filtered and the solvent was removed under reduced pressure by rotary evaporation. The crude material was purified by flash column chromatography (SiO_2, EtOAc/Hex 1:1) and afforded methyl 4-(2-methylquinoline-6-carboxamido)benzoate (**39**, 85.0 mg, 267 µmol, 50%) as a white solid. ^{1}H NMR (400 MHz, DMSO-d_6): δ = 2.71 (s, 3 H) 3.85 (s, 3 H) 7.54 (d, *J*=8.53 Hz, 1 H) 7.99 (s, 4 H) 8.04 (d, *J*=8.78 Hz, 1 H) 8.22 (dd, *J*=8.78, 2.01 Hz, 1 H) 8.42 (d, *J*=8.53 Hz, 1 H) 8.60 (d, *J*=1.76 Hz, 1 H) 10.77 (s, 1 H) ^{13}C NMR (DMSO-d_6, 101 MHz): δ = 165.8, 165.6, 161.0, 148.5, 143.6, 137.1, 131.5, 130.1, 128.4, 128.0, 125.3, 124.4, 123.1, 119.6, 51.9, 38.2, 25.0 ppm. HRMS (ESI): m/z calculated for $C_{19}H_{16}N_2O_3$ $(M+H)^+$ 321.1229, found 321.1234.

Compound 40

Methyl 4-(2-methylquinoline-6-carboxamido)benzoate (**39**, 85.0 mg, 267 µmol, 1.00 eq.) was dissolved in MeOH/THF (2 mL, 1:1) and 5 M KOH solution (1 mL) was added and the reaction mixture was stirred for 18 h at room temperature. The volatiles were removed under reduced pressure by rotary evaporation and a 3 M HCl solution (2 mL) was added. The precipitated solid was filtered and washed by 1 M HCl solution. After drying at high vacuum 4-(2-methylquinoline-6-carboxamido)benzoic acid (**40**, 81.0 mg, 267 µmol, 100%) was obtained as brownish solid. HRMS (ESI): m/z calculated for $C_{18}H_{14}N_2O_3$ $(M+H)^+$ 307.1074, found 307.1077.

Final coupling of Albi-3

HATU (67.0 mg, 176 µmol, 1.35 eq.) was added to a solution of AB building block **40** (51.9 mg, 169 µmol, 1.30 eq.) in anhydrous DMF (1 mL) and the resulting solution was stirred at r.t for 45 min. A solution of tetrapeptide **41**[1] (94.0 mg, 130 µmol, 1.00 eq.) and DIPEA (136 µL, 780 µmol, 6.00 eq.) in anhydrous DMF (1 mL) was added dropwise and the reaction mixture was stirred at r.t for 16 h. All volatiles were removed in vacuo and the residue was taken up in a mixture of THF (1 mL) and MeOH (1 mL), and 3 N $KOH_{(aq.)}$ (1 mL) was added dropwise. After 45 min of stirring, 3 N $HCl_{(aq.)}$ (1.1 mL) was added and the resulting suspension was evaporated under reduced pressure. The crude material was dissolved in DMSO, centrifuged, and the supernatant purified by HPLC (PLRP-S column, CH_3CN in H_2O). The title compound **Albi-3** (13 mg, 9% over two steps) was obtained as a colorless solid. ^1H NMR (DMSO-d_6, 700 MHz): δ = 11.71 (br. s., 1 H), 11.58 (br. s., 1 H), 11.12 (s, 1 H), 10.86 (s, 1 H), 10.82 (s, 1 H), 10.49 (s, 1 H), 8.94 - 9.01 (m, 1 H), 8.84 (d, *J*=7.5 Hz, 2 H), 8.77 (s, 1 H), 8.40 (d, *J*=8.8 Hz, 1 H), 8.34 (dd, *J*=8.5, 2.1 Hz, 1 H), 8.21 (d, *J*=8.5 Hz, 1 H), 8.17 (d, *J*=8.8 Hz, 1 H), 8.11 (d, *J*=8.8 Hz, 1 H), 8.03 (d, *J*=9.0 Hz, 1 H), 7.92 - 7.98 (m, 4 H), 7.88 (d, *J*=9.0 Hz, 1 H), 7.82 (d, *J*=8.1 Hz, 1 H), 7.72 (br. s., 1 H), 7.59 (d, *J*=8.8 Hz, 1 H), 4.96 (br. s., 2 H), 3.92 (s, 3 H), 3.88 (s, 3 H), 3.34 (dd, *J*=14.8, 5.7 Hz, 1 H), 3.28 (dd, *J*=14.8, 9.3 Hz, 1 H), 2.86 ppm (s, 3 H) H,C-HSQC NMR (DMSO-d_6, 101 MHz): δ =172.4, 171.6, 171.6, 171.6, 171.5, 166.4, 166.4, 165.3, 165.3, 165.2, 163.9, 163.8, 161.8, 160.9, 154.8, 150.0, 143.9, 143.8, 142.3, 140.0, 139.9, 139.9, 139.9, 139.3, 139.2, 138.2, 137.8, 137.7, 137.7, 136.6, 136.6, 136.6, 135.9, 133.6, 130.8, 129.1, 129.0, 127.6, 127.6, 127.6, 126.1, 126.1, 126.1, 124.3, 119.8, 119.8,

119.8, 119.8, 115.9, 115.9, 110.8, 110.8, 110.8, 110.7, 110.7, 110.7, 109.4, 60.6, 60.6, 54.7, 54.6, 39.9, 27.5, 23.4, 23.4 ppm. HRMS (ESI): m/z calculated for $C_{45}H_{38}N_{10}O_{11}$ (M+H)+: 895.2786, found 895.2794.

Supplementary Method 6. Synthesis of alkyne-Albi

Final coupling of alkyne-Albi

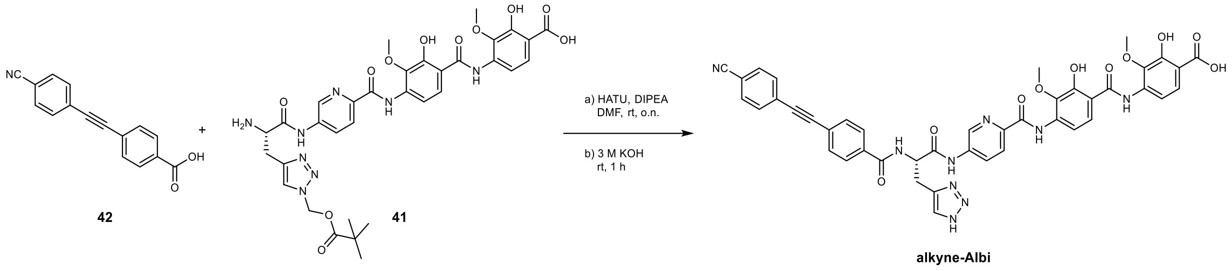

HATU (84.5 mg, 222 µmol, 1.4 eq.) was added to a solution of biaryl alkyne **42**[4] (51.0 mg, 275 µmol, 1.3 eq.) in anhydrous DMF (3 mL) and the resulting solution was stirred at r.t for 45 min. A solution of tetrapeptide **41**[1] (114 mg, 159 µmol, 1.0 eq.) and DIPEA (0.138 mL) in anhydrous DMF (1 mL) was added dropwise and the reaction mixture was stirred at r.t for 16 h. All volatiles were removed in vacuo and the residue was taken up in a mixture of THF (1 mL) and MeOH (1 mL), and 3 N $KOH_{(aq.)}$ (1 mL) was added dropwise. After 45 min of stirring, 3 N $HCl_{(aq.)}$ (1.1 mL) was added and the resulting suspension was evaporated under reduced pressure. The crude material was dissolved in DMSO, centrifuged, and the supernatant purified by HPLC (PLRP-S column, CH_3CN in H_2O). The title compound **alkyne-Albi** (13 mg, 10% over two steps) was obtained as a colorless solid. ^{1}H NMR (DMSO-d_6, 500 MHz): δ = 11.70 (br. s., 1 H), 11.59 (br. s., 1 H), 11.11 (s, 1 H), 10.86 (s, 1 H), 10.48 (s, 1 H), 9.05 (d, J=7.2 Hz, 1 H), 8.97 (s, 1 H), 8.33 (d, J=8.5 Hz, 1 H), 8.20 (d, J=8.4 Hz, 1 H), 8.11 (d, J=8.9 Hz, 1 H), 7.99 - 8.05 (m, 2 H), 7.96 (d, J=8.1 Hz, 3 H), 7.92 (d, J=8.2 Hz, 3 H), 7.88 (d, J=8.9 Hz, 1 H), 7.78 (d, J=8.2 Hz, 2 H), 7.72 (d, J=8.1 Hz, 3 H), 7.59 (d, J=8.9 Hz, 1 H), 4.91 - 5.00 (m, 1 H), 3.92 (s, 3 H), 3.87 (s, 3 H), 3.24 - 3.37 ppm (m, 2 H) ^{13}C NMR (DMSO-d_6, 126 MHz): δ 139.2, 132.4, 132.0, 131.4, 129.1, 127.9, 127.8, 127.0, 126.3, 125.4, 122.7, 110.1, 60.5, 60.0, 54.1, 52.1, 39.4 ppm. HRMS (ESI): m/z calculated for $C_{43}H_{34}N_9O_{10}$ $(M+H)^+$ 836.2423, found 836.2402.

Supplementary Method 7. Synthesis of AE-1, AE-2 and AE-3

Synthesis of o-Anisidine variations

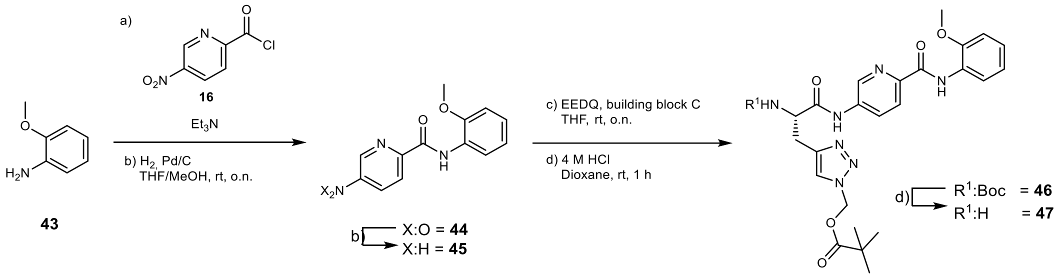

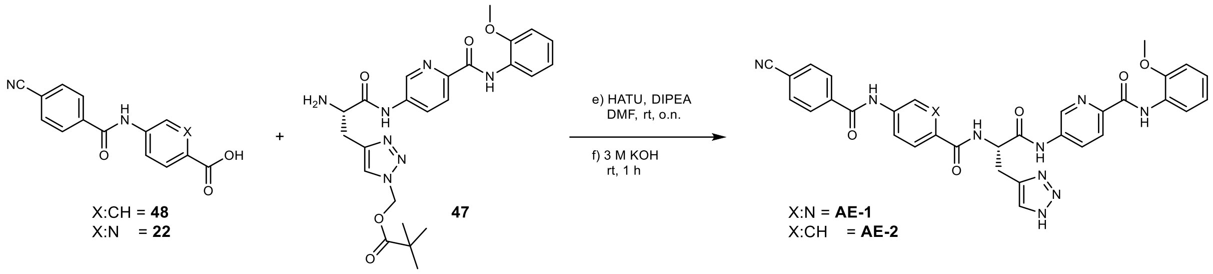

Compound 44

Compound **44** was synthesized from acid chloride **16** (5.79 g, 31.0 mmol, 1.00 eq.), amine **43** (4.20, 34.1 mmol, 1.10 eq.) and Et_3N (8.65 mL, 62.1 mmol, 2.00 eq.) according to *standard procedure I* - column chromatography: SiO_2, *n*-hexane/EtOAc, 7:1. Compound **44** (8.2 g, 97%) was obtained as a yellow solid. ^{1}H NMR (500 MHz, DMSO-d_6): δ = 10.36 (s, 1 H), 9.48 (dd, J=2.5, 0.5 Hz, 1 H), 8.83 (dd, J=8.5, 2.6 Hz, 1 H), 8.36 - 8.43 (m, 13 H), 7.12 - 7.23 (m, 14 H), 7.02 (ddd, J=8.2, 5.9, 2.7 Hz, 7 H), 3.95 ppm (s, 3 H) ^{13}C NMR (126 MHz, DMSO-d_6): δ = 160.0, 153.7, 149.2, 146.4, 144.6, 134.4, 126.8, 125.4, 123.4, 121.2, 119.8, 111.6, 56.6 ppm. HRMS (ESI): m/z calculated for $C_{13}H_{12}N_3O_4$ $(M+H)^+$ 274.0822, found 274.0830.

Compound 45

A solution of the nitro compound **44** (7.10 g, 26.0 mmol, 1.00 eq) in a mixture of MeOH (75 mL) and THF (75 mL) was purged with N_2 for 5 min, then Pd (10 wt.% on activated carbon, 1.38 g) was added. The resulting suspension was purged with N_2 for 5 min followed by H_2 for 5 min. The reaction mixture was stirred at r.t. under a H_2-atmosphere overnight. The suspension was filtered through a pad of Celite® and the filtrate concentrated under reduced pressure to afford the title compound **45** (5.84 g, 20.6 mmol, 79%) as a colorless solid. ^{1}H NMR (DMSO-d_6, 500 MHz): δ = 10.20 (s, 1

H), 8.44 (dd, *J*=7.9, 1.4 Hz, 1 H), 8.12 (d, *J*=2.6 Hz, 1 H), 7.94 (d, *J*=8.5 Hz, 1 H), 7.30 (d, *J*=7.6 Hz, 1 H), 7.23 (dd, *J*=8.6, 2.7 Hz, 1 H), 7.04 - 7.12 (m, 2 H), 6.94 - 7.00 (m, 1 H), 5.39 (td, *J*=7.0, 4.0 Hz, 1 H), 3.92 ppm (s, 3 H) ^{13}C NMR (DMSO-d_6, 126 MHz): δ = 162.4, 148.6, 146.6, 138.6, 135.0, 127.9, 123.9, 123.5, 121.1, 119.8, 118.9, 111.3, 83.9, 66.3, 56.4, 31.5 ppm. HRMS (ESI): m/z calculated for $C_{13}H_{14}N_3O_2$ (M+H)$^+$ 244.1081, found 244.1077.

Compound 46

Compound **46** was synthesized from Boc-protected azahistidine (1.71 g, 4.62 mmol, 1.50 eq.), dipeptide **45** (750 mg, 3.08 mmol, 1.00 eq.) and EEDQ (1.14 g, 4.62 mmol, 1.50 eq.) according to *standard procedure D* - column chromatography: SiO_2, Hexane/EtOAc (1:4). Compound **46** (1.80 g, 98%) was obtained as a yellow solid. ^{1}H NMR (DMSO-d_6, 500 MHz): δ = 10.70 (s, 1 H), 10.31 (s, 1 H), 8.86 (d, J=2.1 Hz, 1 H), 8.39 - 8.49 (m, 1 H), 8.30 (dd, J=8.5, 2.4 Hz, 1 H), 8.16 (d, J=8.7 Hz, 1 H), 7.99 (s, 1 H), 7.28 (d, J=7.9 Hz, 1 H), 7.07 - 7.18 (m, 2 H), 7.01 (dd, J=7.9, 2.4 Hz, 1 H), 6.28 (s, 2 H), 4.37 - 4.49 (m, 1 H), 3.95 (s, 3 H), 2.99 - 3.19 (m, 2 H), 1.37 (s, 8 H), 1.08 ppm (s, 9 H) ^{13}C NMR (DMSO-d_6, 126 MHz): δ = 176.9, 171.7, 161.5, 155.8, 148.9, 144.3, 139.9, 138.9, 127.6, 127.4, 124.7, 124.5, 123.1, 121.1, 119.3, 111.4, 78.9, 70.3, 60.2, 56.5, 55.4, 28.6, 28.1, 26.9 ppm. HRMS (ESI): m/z calculated for $C_{29}H_{38}N_7O_7$ (M+H)$^+$ 596.2827, found 596.2831.

Compound 47

Compound **47** was synthesized from Boc-protected tripeptide **45** (1.65 g, 2.77 mmol, 1.00 eq.) with 4 N HCl in dioxane (40 mL) according to *standard procedure E*. Compound **47** (1.47 g, quant.) was obtained as light-yellow solid. ^{1}H NMR (DMSO-d_6, 500 MHz): δ = 11.61 (s, 1 H), 10.32 (s, 1 H), 8.91 (dd, J=2.4, 0.5 Hz, 1 H), 8.62 (d, J=4.9 Hz, 3 H), 8.42 - 8.46 (m, 1 H), 8.29 (dd, J=8.6, 2.4 Hz, 1 H), 8.16 - 8.21 (m, 2 H), 7.08 - 7.17 (m, 2 H), 7.01 (ddd, J=8.2, 6.1, 2.5 Hz, 1 H), 6.28 (d, J=1.2 Hz, 2 H), 4.39 - 4.49 (m, 1 H), 3.95 (s, 3 H), 3.57 (s, 2 H), 1.06 ppm (s, 9 H) ^{13}C NMR (DMSO-d_6, 126 MHz): δ = 176.8, 167.8, 161.4, 148.9, 145.0, 141.2, 140.0, 138.1, 128.0, 127.4, 125.5, 124.6, 123.2, 121.1, 119.3, 111.5, 70.4, 66.8, 56.5, 53.2, 26.9 ppm HRMS (ESI): m/z calculated for $C_{24}H_{30}N_7O_5$ (M+H)$^+$ 496.2303, found 496.2299.

AE-1

HATU (229 mg, 602 µmol, 2.00 eq.) was added to a solution of AB dipeptide **48**[3] (120 mg, 451 µmol, 1.5 eq.) in anhydrous DMF (3 mL) and the resulting solution was stirred at r.t for 45 min. A solution of tripeptide **47** (160 mg, 301 µmol, 1.0 eq.) and DIPEA (0.524 mL) in anhydrous DMF (1 mL) was added dropwise and the reaction mixture was stirred at r.t for 16 h. All volatiles were removed in vacuo and the residue was taken up in a mixture of THF (1 mL) and MeOH (1 mL), and 3 N $KOH_{(aq.)}$ (1 mL) was added dropwise. After 45 min of stirring, 3 N $HCl_{(aq.)}$ (1.1 mL) was added and the resulting suspension was evaporated under reduced pressure. The crude material was dissolved in DMSO, centrifuged, and the supernatant purified by HPLC (PLRP-S column, CH_3CN in H_2O). The title compound **AE-1** (49 mg, 26% over two steps) was obtained as a colourless solid. ^1H NMR (DMSO-d_6, 500 MHz): δ = 10.98 (s, 1 H), 10.85 (s, 1 H), 10.32 (s, 1 H), 9.05 (d, J=2.1 Hz, 1 H), 8.84 - 8.92 (m, 2 H), 8.39 - 8.50 (m, 2 H), 8.26 - 8.34 (m, 1 H), 8.13 - 8.21 (m, 3 H), 8.08 (dd, J=8.4, 2.9 Hz, 3 H), 7.66 (s, 1 H), 7.06 - 7.19 (m, 2 H), 6.94 - 7.06 (m, 1 H), 5.03 (d, J=7.5 Hz, 1 H), 3.95 (s, 3 H), 3.38 ppm (d, J=6.4 Hz, 2 H) ^{13}C NMR (DMSO-d_6, 126 MHz): δ = 170.7, 165.3, 163.8, 161.5, 158.9, 148.9, 144.8, 144.6, 140.9, 140.0, 138.7, 138.7, 138.5, 133.1, 129.2, 128.3, 127.9, 127.4, 124.6, 123.2, 123.0, 121.1, 119.4, 118.7, 114.8, 111.4, 56.5, 53.9 ppm. HRMS (ESI): m/z calculated for $C_{32}H_{27}N_{10}O_5$ $(M+H)^+$ 631.2160, found 631.2155.

AE-2

HATU (229 mg, 602 µmol, 2.00 eq.) was added to a solution of AB dipeptide **48**[3] (120 mg, 451 µmol, 1.5 eq.) in anhydrous DMF (3 mL) and the resulting solution was stirred at r.t for 45 min. A solution of tripeptide **47** (160 mg, 301 µmol, 1.0 eq.) and DIPEA (0.524 mL) in anhydrous DMF (1 mL) was added dropwise and the reaction mixture was stirred at r.t for 16 h. All volatiles were removed *in vacuo* and the residue was taken up in a mixture of THF (1 mL) and MeOH (1 mL), and 3 N $KOH_{(aq.)}$ (1 mL) was added dropwise. After 45 min of stirring, 3 N $HCl_{(aq.)}$ (1.1 mL) was added and the resulting suspension was evaporated under reduced pressure. The crude material was dissolved in DMSO, centrifuged, and the supernatant purified by HPLC (PLRP-S column, CH_3CN in H_2O). The title compound **AE-2** (64 mg, 34% over two steps) was obtained as a colorless solid. ^1H NMR (DMSO-d_6, 500 MHz): δ = 10.82 (s, 1 H), 10.71 (s, 1 H), 10.32 (s, 1 H), 8.91 (br. s., 1 H), 8.83 (d, J=7.5 Hz, 1 H), 8.44 (d, J=7.9 Hz, 1 H), 8.34 (d, J=8.2 Hz, 1 H), 8.17 (d, J=8.7 Hz, 1 H), 8.10 - 8.15 (m, J=7.8 Hz, 2 H),

8.01 - 8.09 (m, J=7.9 Hz, 2 H), 7.84 - 7.98 (m, 4 H), 7.71 (br. s., 1 H), 7.13 (br. s., 2 H), 7.00 (t, J=6.6 Hz, 1 H), 4.95 (d, J=6.9 Hz, 1 H), 3.95 (s, 3 H), 3.21 - 3.41 ppm (m, 2 H) ^{13}C NMR (DMSO-d$_6$, 126 MHz): δ = 171.5, 166.5, 164.9, 161.5, 148.9, 144.4, 142.2, 139.9, 139.1, 138.9, 133.0, 129.4, 129.1, 128.9, 127.7, 127.4, 124.5, 123.2, 121.1, 120.0, 119.3, 118.7, 114.5, 111.4, 56.5, 54.8 ppm. HRMS (ESI): m/z calculated for $C_{33}H_{28}N_9O_5$ $(M+H)^+$ 630.2208, found 630.2209.

Final coupling of AE-3

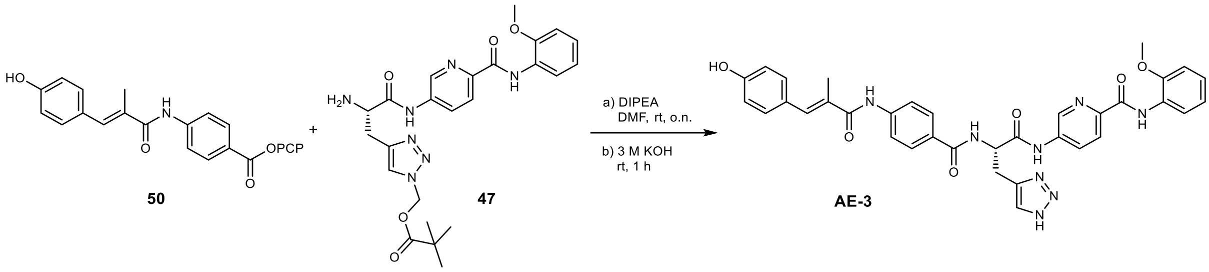

AE-3

Compound **AE-3** was synthesized from PCP-ester **50**[2] (100 mg, 183 µmol, 1.10 eq.), tripeptide **47** (88.6 mg, 167 µmol, 1.00 eq.) and DIPEA (145 µl, 0.833 mmol) according to *standard procedure G.* Final derivative **AE-3** (29 mg, 22%) was obtained as a colorless solid. ^{1}H NMR (DMSO-d$_6$, 500 MHz): δ = 10.81 (br. s., 1 H), 10.32 (br. s., 1 H), 10.08 (br. s., 1 H), 9.78 (br. s., 1 H), 8.90 (br. s., 1 H), 8.76 (d, *J*=7.2 Hz, 1 H), 8.44 (d, *J*=7.3 Hz, 1 H), 8.34 (d, *J*=7.9 Hz, 1 H), 8.17 (d, *J*=8.1 Hz, 1 H), 7.77 - 7.92 (m, 4 H), 7.71 (br. s., 1 H), 7.32 - 7.41 (m, *J*=8.1 Hz, 2 H), 7.27 (br. s., 1 H), 7.13 (br. s., 2 H), 7.00 (br. s., 1 H), 6.80 - 6.89 (m, *J*=7.9 Hz, 2 H), 4.94 (d, *J*=6.7 Hz, 1 H), 3.95 (s, 3 H), 3.21 - 3.39 (m, 2 H), 2.12 ppm (br. s., 3 H) ^{13}C NMR (DMSO-d$_6$, 126 MHz): δ = 171.6, 169.3, 166.6, 161.5, 158.0, 148.9, 144.4, 143.0, 139.9, 138.9, 134.3, 131.7, 130.0, 128.7, 128.5, 127.7, 127.4, 127.0, 124.5, 123.2, 121.1, 119.5, 119.3, 115.9, 111.4, 56.5, 54.7, 15.0 ppm. HRMS (ESI): m/z calculated for $C_{35}H_{33}N_8O_6$ $(M+H)^+$ 661.2518, found 661.2521.

Supplementary Method 8. Synthesis of AE-4

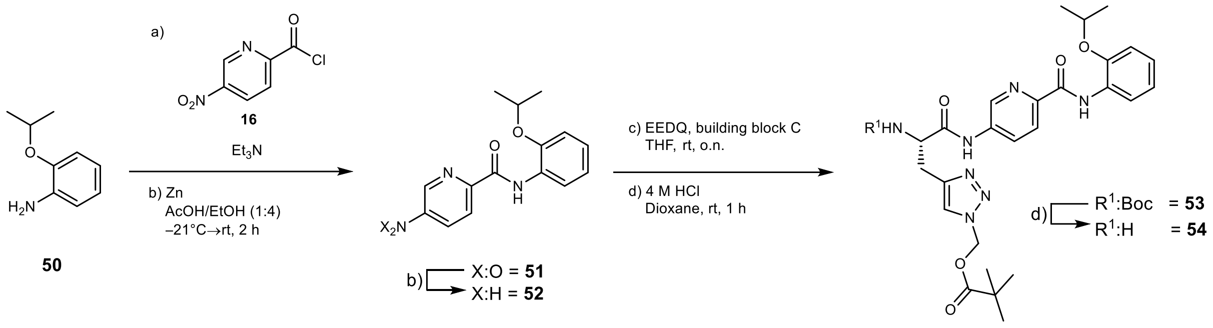

Compound 51

Compound **51** was synthesized from acid chloride **16** (2.90 g, 15.6 mmol, 1.00 eq.), amine **50** (2.59 g, 17.1 mmol, 1.10 eq.) and Et_3N (4.33 mL, 31.1 mmol, 2.00 eq.) according to *standard procedure I* - column chromatography: SiO_2, *n*-hexane/EtOAc, 4:6. Compound **51** (2.88 g, 62%) was obtained as a red solid. ^{1}H NMR ($CDCl_3$-d, 500 MHz): δ = 10.54 (br. s., 1 H), 9.39 (dd, *J*=2.6, 0.6 Hz, 1 H), 8.60 (dd, *J*=8.5, 2.4 Hz, 1 H), 8.50 (dd, *J*=8.0, 1.6 Hz, 1 H), 8.43 (dd, *J*=8.6, 0.7 Hz, 1 H), 7.00 - 7.06 (m, 1 H), 6.95 (td, *J*=7.7, 1.2 Hz, 1 H), 6.90 (dd, *J*=8.1, 1.4 Hz, 1 H), 4.51 - 4.65 (m, 1 H), 1.53 (s, 1 H), 1.38 - 1.41 (m, 3 H), 1.37 ppm (s, 3 H) ^{13}C NMR ($CDCl_3$-d, 126 MHz): δ = 159.5, 154.8, 147.2, 145.6, 143.9, 132.8, 128.2, 124.7, 122.9, 121.3, 119.9, 113.5, 72.0, 22.2 ppm. HRMS (ESI): m/z calculated for $C_{15}H_{16}N_3O_4$ $(M+H)^+$ 302.1135, found 302.1134.

Compound 52

Compound **52** was synthesized from nitro compound **51** (770 mg, 2.56 mmol, 1.00 eq.) and Zn (8.35 g, 128 mmol, 50.0 eq.) according to *standard procedure A*. The title compound **52** (653 mg, 92%) was obtained as yellow solid. HRMS (ESI): m/z calculated for $C_{15}H_{18}N_3O_2$ $(M+H)^+$ 272.1394, found 272.1395.

Compound 53

Compound **53** was synthesized from Boc-protected azahistidine (751 mg, 2.03 mmol, 1.1 eq.), dipeptide **52** (500 mg, 1.84 mmol, 1.00 eq.) and EEDQ (547 mg, 2.21 mmol, 1.20 eq.) according to s*tandard procedure D* - column chromatography: SiO_2, EtOAc/Hexane (2:6). Compound **53** (880 mg, 77%) was obtained as a yellow solid. ^{1}H NMR (DMSO-d_6, 400 MHz): δ = 10.67 (s, 1 H), 10.47 (s, 1 H), 8.91 (d, *J*=2.3 Hz, 1 H), 8.45 (dd, *J*=8.0, 1.8 Hz, 1 H), 8.25 (dd, *J*=8.7, 2.4 Hz, 1 H), 8.15 (d, *J*=8.5 Hz, 1 H),

8.00 (s, 1 H), 7.29 (d, *J*=7.8 Hz, 1 H), 7.12 - 7.18 (m, 1 H), 7.08 (td, *J*=7.7, 1.6 Hz, 1 H), 6.95 - 7.03 (m, 1 H), 6.28 (s, 3 H), 4.69 (quin, *J*=6.0 Hz, 1 H), 4.43 (d, *J*=6.0 Hz, 1 H), 3.15 (dd, *J*=14.4, 5.6 Hz, 1 H), 3.02 (dd, *J*=14.6, 9.0 Hz, 1 H), 1.33 - 1.40 (m, 18 H), 1.07 ppm (s, 11 H) ^{13}C NMR (DMSO-d_6 ,101 MHz): δ = 176.9, 171.7, 161.4, 155.8, 146.9, 144.4, 143.7, 139.9, 138.8, 128.9, 127.6, 124.7, 124.3, 123.0, 121.5, 119.2, 114.8, 78.9, 72.0, 70.3, 60.2, 55.4, 28.6, 26.9, 22.4, 14.6 ppm. HRMS (ESI): m/z calculated for $C_{31}H_{42}N_7O_7$ $(M+H)^+$ 624.3140, found 624.3130.

Compound 54

Compound **54** was synthesized from Boc-protected tripeptide **53** (400 mg, 641 µmol, 1.00 eq.) with 4 N HCl in dioxane (15 mL) according to *standard procedure E*. Compound **54** (322 mg, 96%) was obtained as light-yellow solid. HRMS (ESI): m/z calculated for $C_{26}H_{34}N_7O_5$ $(M+H)^+$ 524.2616, found 524.2594.

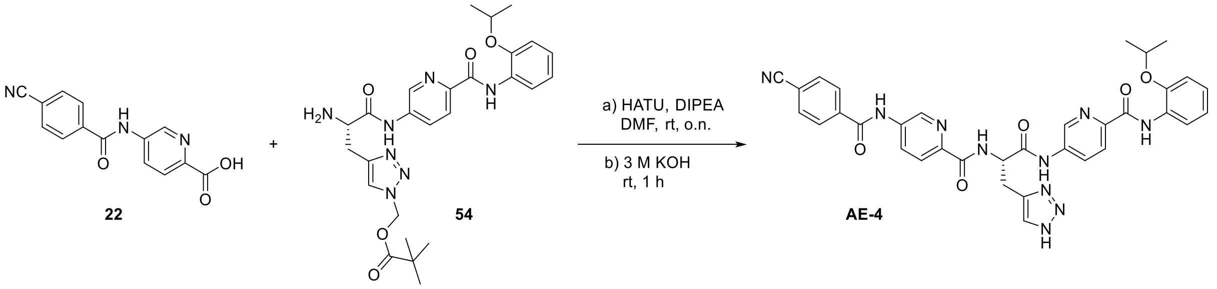

Final coupling of AE-4

HATU (136 mg, 357 µmol, 2.00 eq.) was added to a solution of AB dipeptide **22**[3] (66.8 mg, 250 µmol, 1.4 eq.) in anhydrous DMF (3 mL) and the resulting solution was stirred at r.t for 45 min. A solution of tripeptide **47** (100 mg, 179 µmol, 1.0 eq.) and DIPEA (0.249 mL) in anhydrous DMF (1 mL) was added dropwise and the reaction mixture was stirred at r.t. for 16 h. All volatiles were removed in vacuo and the residue was taken up in a mixture of THF (1 mL) and MeOH (1 mL), and 3 N $KOH_{(aq.)}$ (1 mL) was added dropwise. After 45 min of stirring, 3 N $HCl_{(aq.)}$ (1.1 mL) was added and the resulting suspension was evaporated under reduced pressure. The crude material was dissolved in DMSO, centrifuged, and the supernatant purified by HPLC (PLRP-S column, CH_3CN in H_2O). The title compound **AE-4** (75 mg, 64% over two steps) was obtained as a colorless solid. ^{1}H NMR (DMSO-d_6, 400 MHz): δ = 11.00 (s, 1 H), 10.84 (s, 1 H), 10.49 (s, 1 H), 9.06 (d, *J*=2.0 Hz, 1 H), 8.85 - 8.97 (m, 2 H), 8.39 - 8.49 (m, 3 H), 8.25 (dd, *J*=8.5, 2.3 Hz, 1 H), 8.16 (d, *J*=8.5 Hz, 3 H), 8.04 - 8.11 (m, 3 H), 7.66

(s, 1 H), 7.10 - 7.17 (m, 1 H), 7.03 - 7.10 (m, 1 H), 6.94 - 7.02 (m, 1 H), 4.96 - 5.09 (m, 1 H), 4.60 - 4.74 (m, 1 H), 3.38 (d, *J*=6.3 Hz, 2 H), 2.88 (s, 1 H), 1.36 ppm (dd, *J*=5.8, 1.3 Hz, 6 H) ^{13}C NMR (DMSO-d_6, 101 MHz): δ = 170.7, 165.3, 163.8, 161.4, 146.9, 144.8, 140.8, 140.1, 138.7, 138.5, 133.0, 129.2, 128.9, 128.3, 127.8, 124.3, 123.0, 123.0, 121.5, 119.2, 118.7, 114.8, 72.1, 53.9, 28.0, 22.4 ppm. HRMS (ESI): m/z calculated for $C_{34}H_{31}N_{10}O_5$ $(M+H)^+$ 659.2473, found 659.2478.

Supplementary Method 9. Synthesis of AE-5

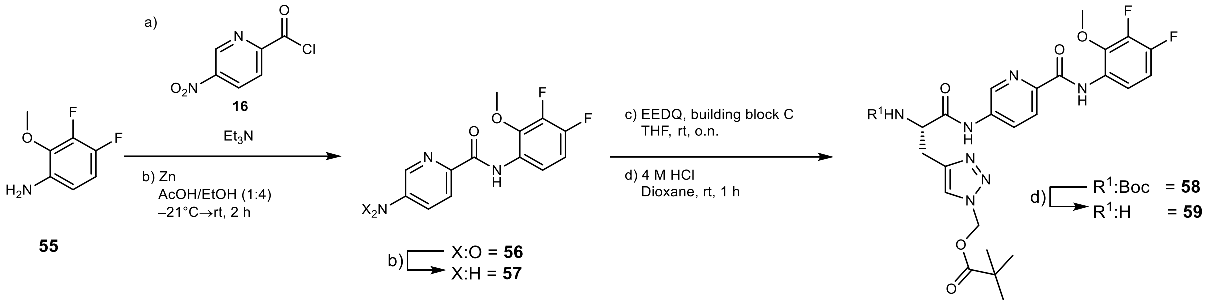

Compound 56

Compound **56** was synthesized from acid chloride **16** (2.90 g, 15.6 mmol, 2.00 eq.), amine **55** (1.36 g, 8.55 mmol, 1.10 eq.) and Et_3N (2.17 mL, 2.00 eq.) according to s*tandard procedure I* - column chromatography: SiO_2, *n*-hexane/EtOAc, 7:1. Compound **56** (2.04 g, 85%) was obtained as a colorless solid. ^1H NMR (DMSO-d_6, 500 MHz): δ = 10.39 (s, 1 H), 9.50 (d, *J*=2.6 Hz, 1 H), 8.84 (dd, *J*=8.5, 2.6 Hz, 1 H), 8.40 (d, *J*=8.5 Hz, 1 H), 8.04 (ddd, *J*=9.2, 5.6, 2.3 Hz, 1 H), 7.18 - 7.31 (m, 1 H), 4.06 ppm (s, 3 H) ^{13}C NMR (DMSO-d_6, 126 MHz): δ = 160.7, 153.4, 148.9, 148.8, 147.0, 146.9, 146.5, 144.8, 144.6, 142.8, 142.7, 140.0, 139.9, 134.4, 127.8, 123.6, 116.4, 116.4, 116.3, 116.3, 111.4, 111.2, 62.4, 62.3 ppm. HRMS (ESI): m/z calculated for $C_{13}H_{10}F_2N_3O_4$ $(M+H)^+$ 310.0634, found 310.0629.

Compound 57

Compound **57** was synthesized from nitro compound **56** (700 mg, 2.26 mmol, 1.00 eq.) and Zn (7.40 g, 113 mmol, 50.0 eq.) according to *standard procedure A*. The title compound **57** (620 mg, 98%) was obtained as yellow solid. ^1H NMR ($CDCl_3$-d, 400 MHz): δ = 10.15 (br. s., 1 H), 8.23 (ddd, J=9.3, 5.5, 2.5 Hz, 1 H), 7.95 - 8.01 (m, 2 H), 7.00 (dd, J=8.4, 2.9 Hz, 1 H), 6.83 (td, J=9.7, 8.3 Hz, 1 H), 4.02 (d, J=2.3 Hz, 4 H), 1.54 ppm (s, 2 H) ^{13}C NMR ($CDCl_3$-d, 101 MHz): δ = 162.8, 145.3, 142.5, 140.2, 135.2, 128.3, 123.6, 121.1, 114.1, 114.0, 113.9, 110.8, 110.6, 61.8 ppm. HRMS (ESI): m/z calculated for $C_{13}H_{12}F_2N_3O_2$ $(M+H)^+$ 280.0892, found 280.0891.

Compound 58

Compound **58** was synthesized from Boc-protected azahistidine (730 mg, 1.97 mmol, 1.10 eq.), dipeptide **57** (500 mg, 1.79 mmol, 1.00 eq.) and EEDQ (509 mg, 2.06 mmol, 1.15 eq.) according to s*tandard procedure D* - column chromatography: SiO_2,

Hexane/EtOAc (8:1). Compound **58** (550 mg, 49%) was obtained as a yellow solid. ^{1}H NMR (DMSO-d_6, 400 MHz): δ = 10.72 (s, 1 H), 10.28 (s, 1 H), 8.89 (d, *J*=2.3 Hz, 1 H), 8.26 - 8.34 (m, 1 H), 8.10 - 8.18 (m, 2 H), 8.00 (s, 2 H), 7.29 (d, *J*=7.8 Hz, 1 H), 7.20 (d, *J*=9.5 Hz, 1 H), 6.29 (s, 2 H), 4.39 - 4.49 (m, 1 H), 4.05 (d, *J*=1.8 Hz, 3 H), 3.16 (dd, *J*=14.6, 5.3 Hz, 1 H), 3.03 (dd, *J*=14.7, 9.2 Hz, 1 H), 1.36 (s, 8 H), 1.08 ppm (s, 9 H) ^{13}C NMR (DMSO-d_6, 101 MHz): δ = 176.9, 171.8, 170.8, 161.9, 155.8, 151.0, 148.2, 146.1, 146.0, 145.0, 144.8, 143.9, 143.7, 139.9, 139.2, 139.1, 136.4, 129.9, 129.4, 128.5, 128.5, 127.5, 127.0, 124.7, 123.3, 121.9, 115.2, 111.4, 111.2, 78.9, 70.3, 62.3, 62.3, 60.2, 55.4, 28.6, 28.1, 26.9, 21.2, 14.5 ppm. HRMS (ESI): m/z calculated for $C_{29}H_{38}F_2N_7O_7$ $(M+H)^+$ 632.2639, found 632.2635.

Compound 59

Compound **59** was synthesized from Boc-protected tripeptide **58** (320 mg, 507 µmol, 1.00 eq.) with 4 N HCl in dioxane (20 mL) according to *standard procedure E*. Compound **59** (266 mg, 99%) was obtained as light-yellow solid. HRMS (ESI): m/z calculated for $C_{24}H_{28}F_2N_7O_5$ $(M+H)^+$ 532.2114, found 532.2101.

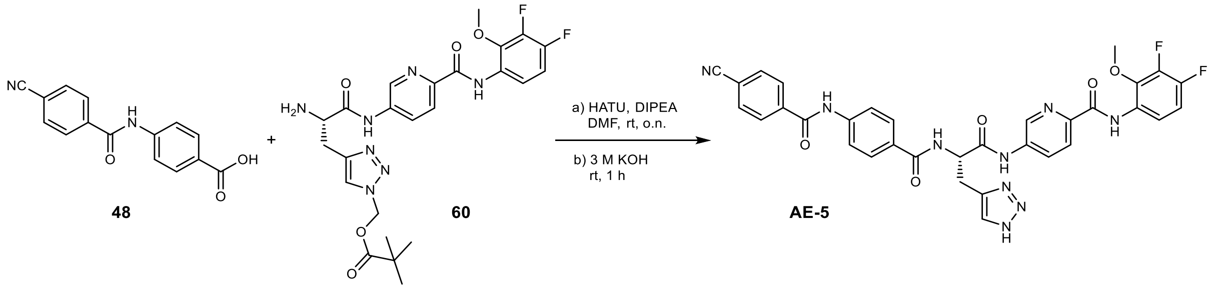

Final coupling of AE-5

HATU (134 mg, 352 µmol, 2.00 eq.) was added to a solution of AB dipeptide **48**[3] (70.3 mg, 264 µmol, 1.5 eq.) in anhydrous DMF (3 mL) and the resulting solution was stirred at r.t for 45 min. A solution of tripeptide **47** (100 mg, 176 µmol, 1.0 eq.) and DIPEA (0.245 mL) in anhydrous DMF (1 mL) was added dropwise and the reaction mixture was stirred at r.t for 16 h. All volatiles were removed in vacuo and the residue was taken up in a mixture of THF (1 mL) and MeOH (1 mL), and 3 N $KOH_{(aq.)}$ (1 mL) was added dropwise. After 45 min of stirring, 3 N $HCl_{(aq.)}$ (1.1 mL) was added and the resulting suspension was evaporated under reduced pressure. The crude material was dissolved in DMSO, centrifuged, and the supernatant purified by HPLC (PLRP-S

column, CH_3CN in H_2O). The title compound **AE-5** (33 mg, 28% over two steps) was obtained as a colorless solid. ^1H NMR (DMSO-d_6, 400 MHz): δ = 14.69 (br. s., 1 H), 10.86 (s, 1 H), 10.72 (s, 1 H), 10.29 (s, 1 H), 8.93 (d, *J*=2.0 Hz, 1 H), 8.85 (d, *J*=7.5 Hz, 1 H), 8.29 - 8.38 (m, 1 H), 8.10 - 8.20 (m, 4 H), 8.05 (d, *J*=8.3 Hz, 2 H), 7.84 - 7.98 (m, 4 H), 7.70 (br. s., 1 H), 7.22 (q, *J*=9.4 Hz, 1 H), 4.87 - 5.01 ppm (m, 1 H) ^{13}C NMR (DMSO-d_6, 101 MHz): δ = 171.1, 166.0, 164.4, 161.5, 143.4, 141.7, 139.4, 138.6, 132.5, 128.9, 128.6, 128.4, 128.0, 127.1, 122.9, 119.5, 118.3, 114.9, 114.0, 110.9, 110.7, 61.9, 61.8, 54.3 ppm. HRMS (ESI): m/z calculated for $C_{33}H_{26}F_2N_9O_5$ $(M+H)^+$ 666.2019, found 666.2016.

Supplementary Method 10. Spectral data

Compound 3

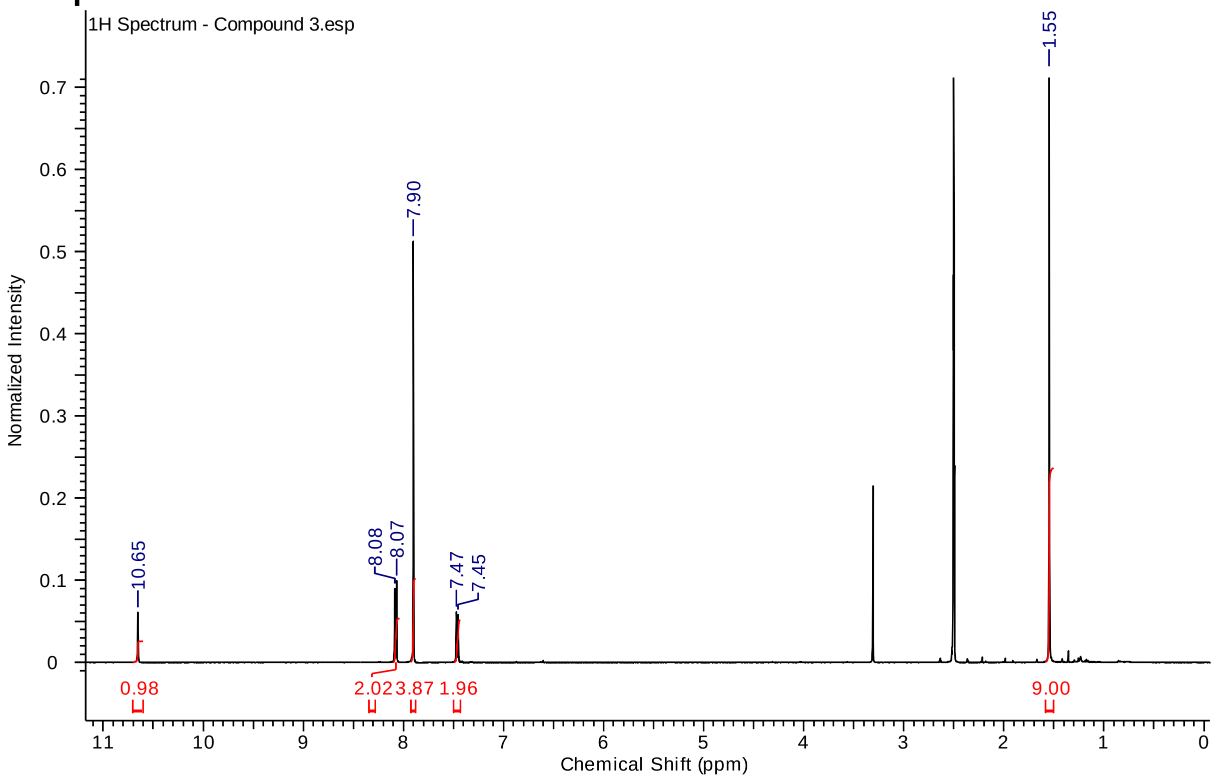

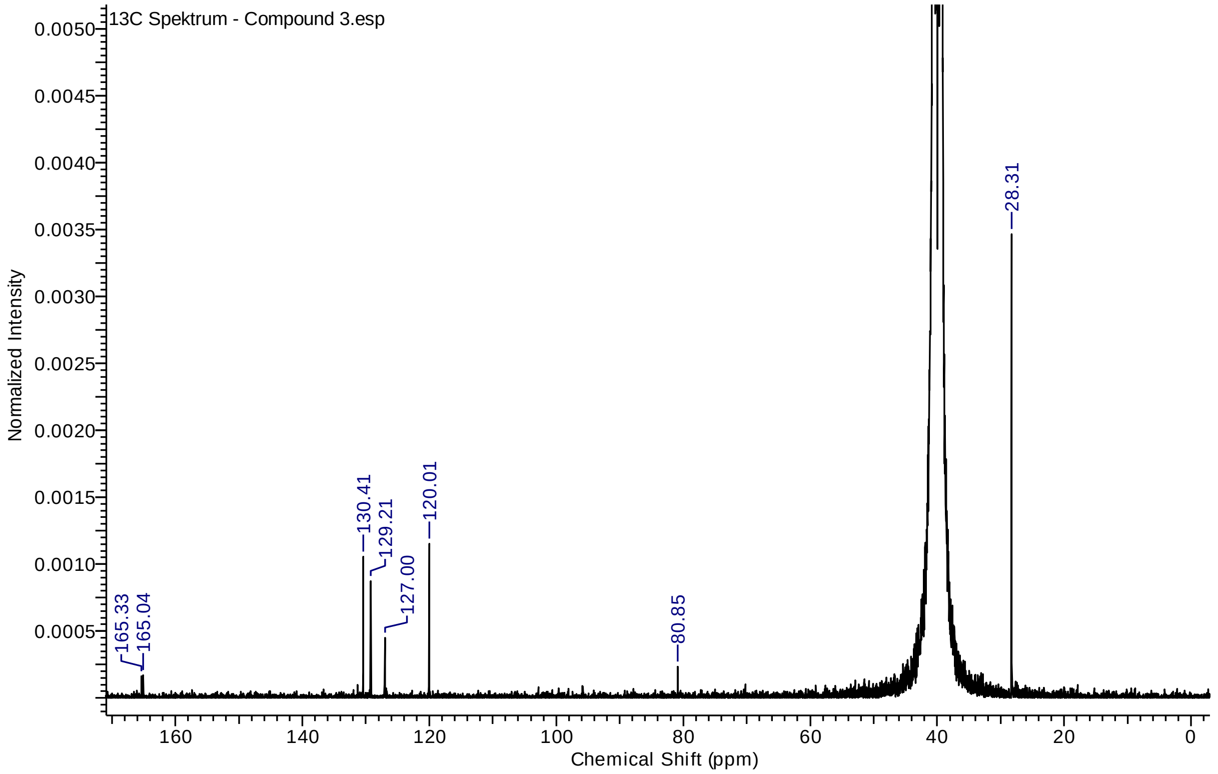

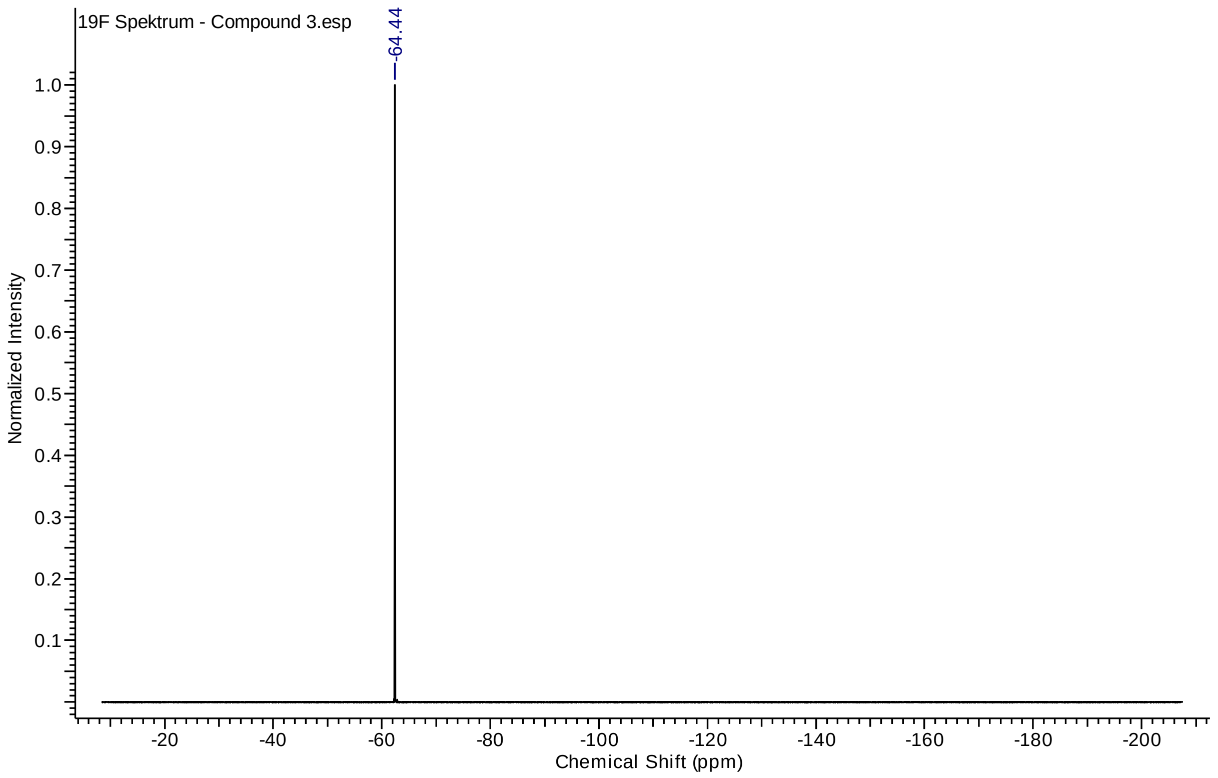
19F Spektrum - Compound 3.esp
-64.44
Normalized Intensity
Chemical Shift (ppm)

Compound 6

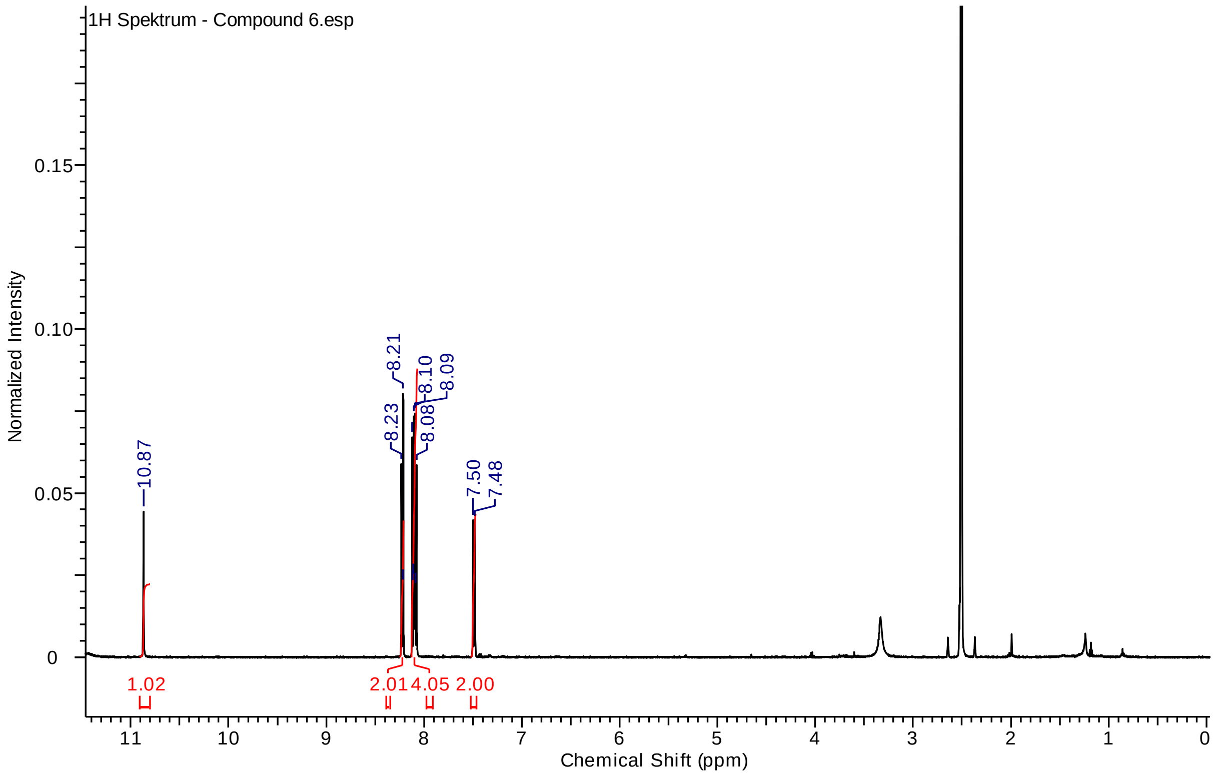
1H Spektrum - Compound 6.esp
10.87
8.23
8.21
8.10
8.09
8.08
7.50
7.48
1.02
2.01
4.05
2.00
Normalized Intensity
Chemical Shift (ppm)

Compound 8

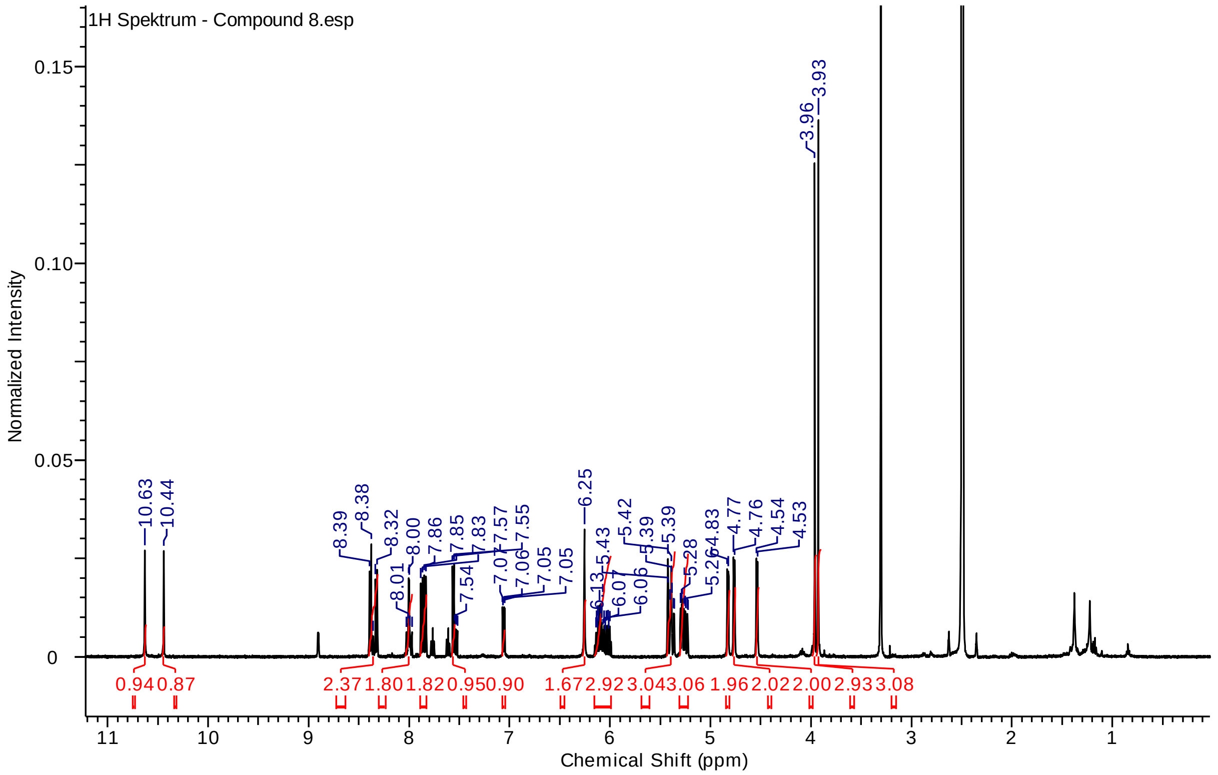

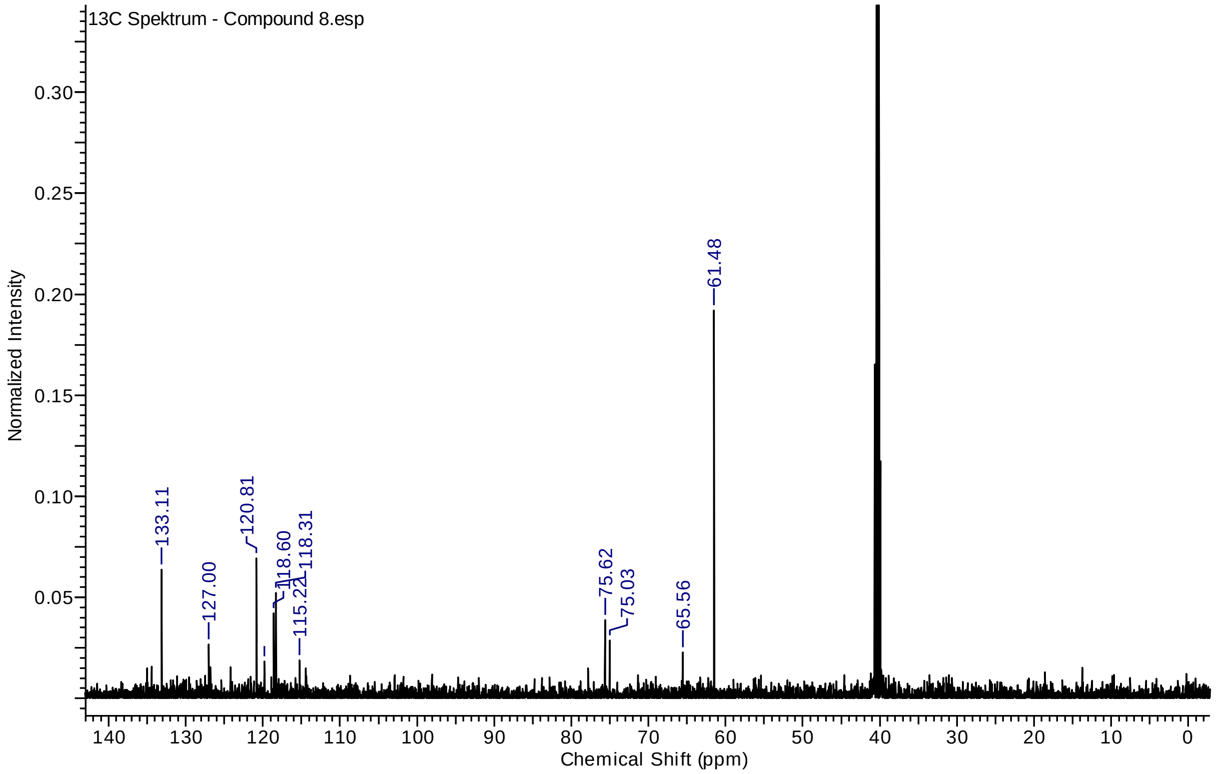

Photo-Albi

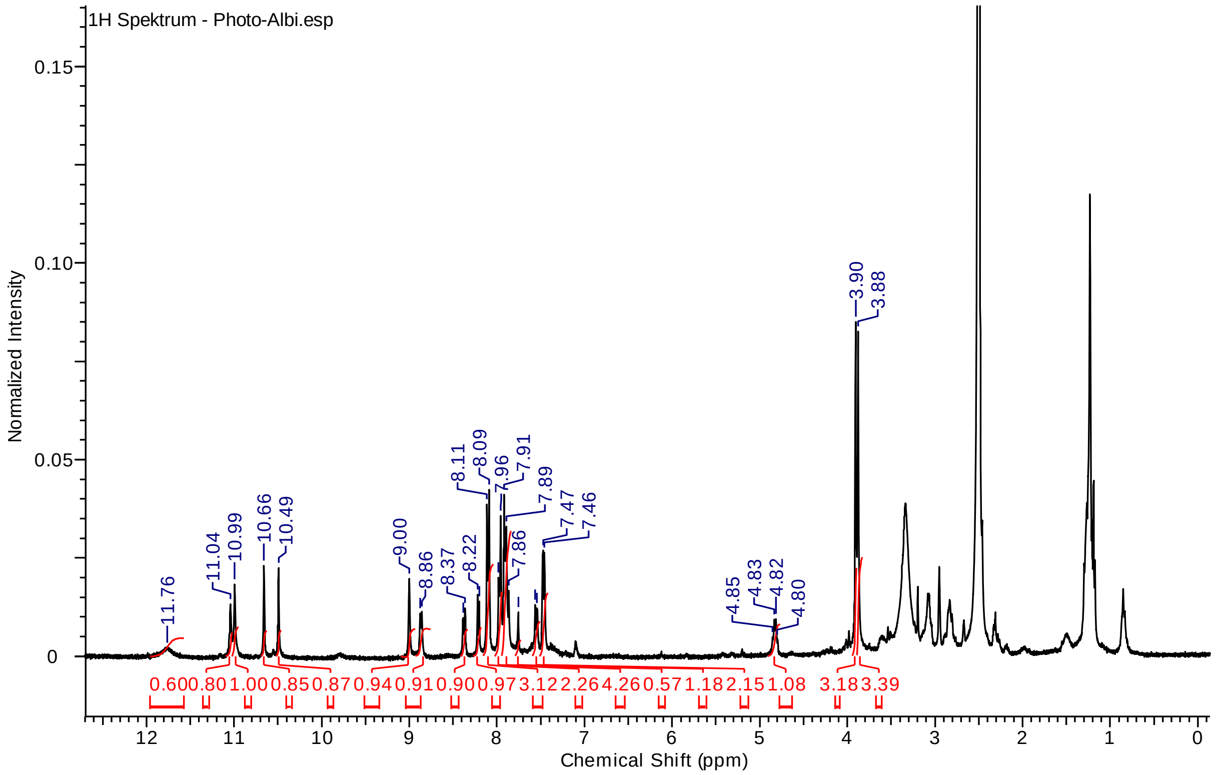

(^{1}H, ^{13}C)-HMBC - Photo-Albi

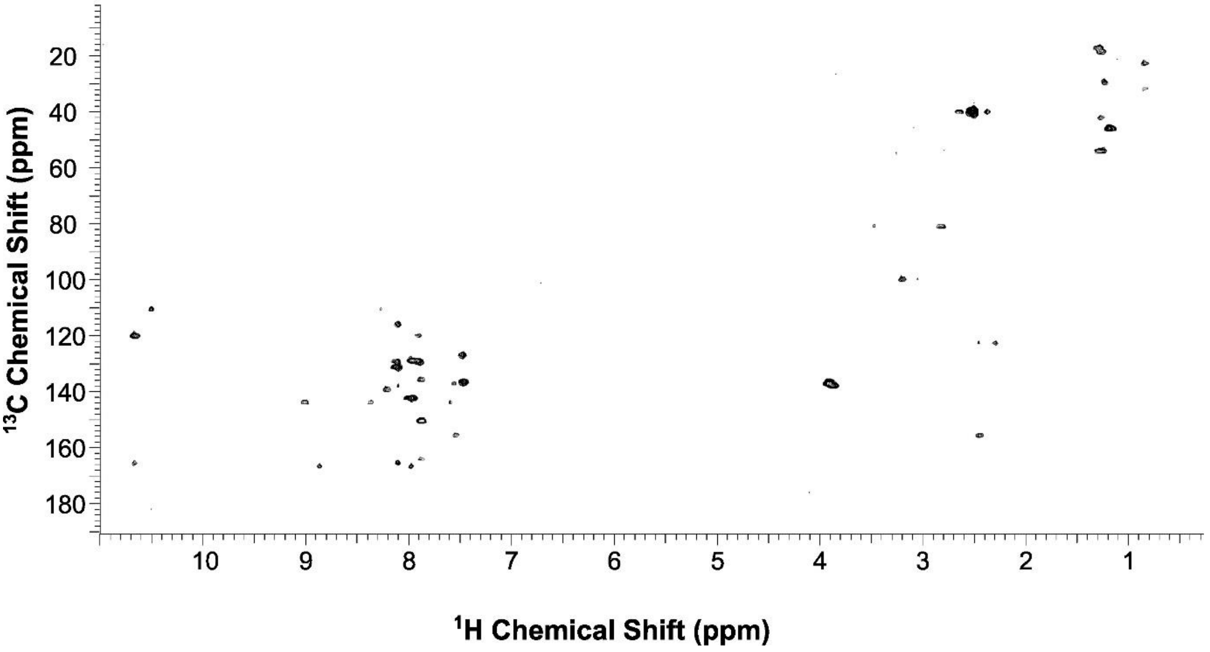

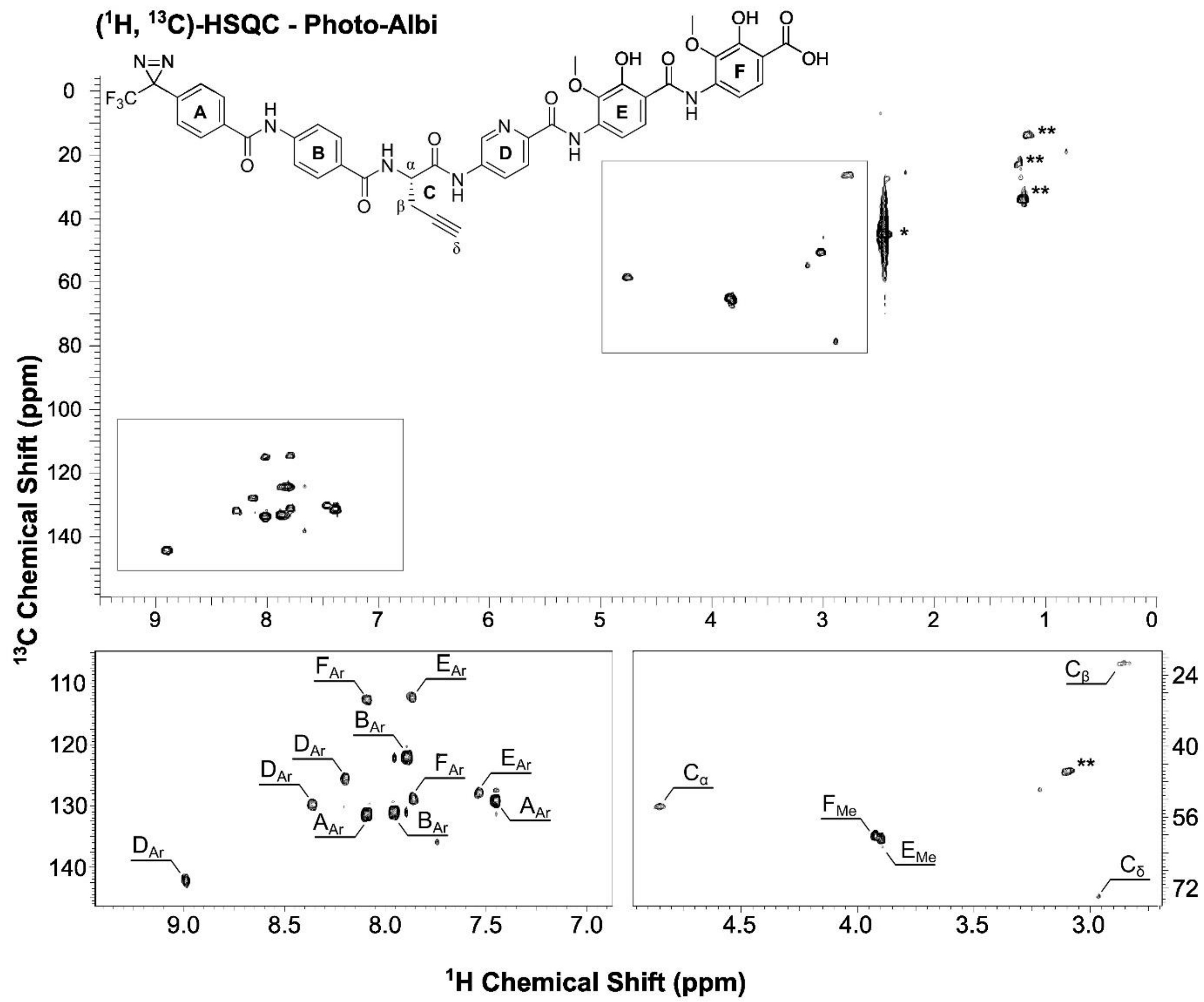

^1H-^{13}C-HSQC with magnification of relevant sections and annotations of signals (Ar: Aromatic, Me: Methyl, *: DMSO, ** impurity).

Compound 12

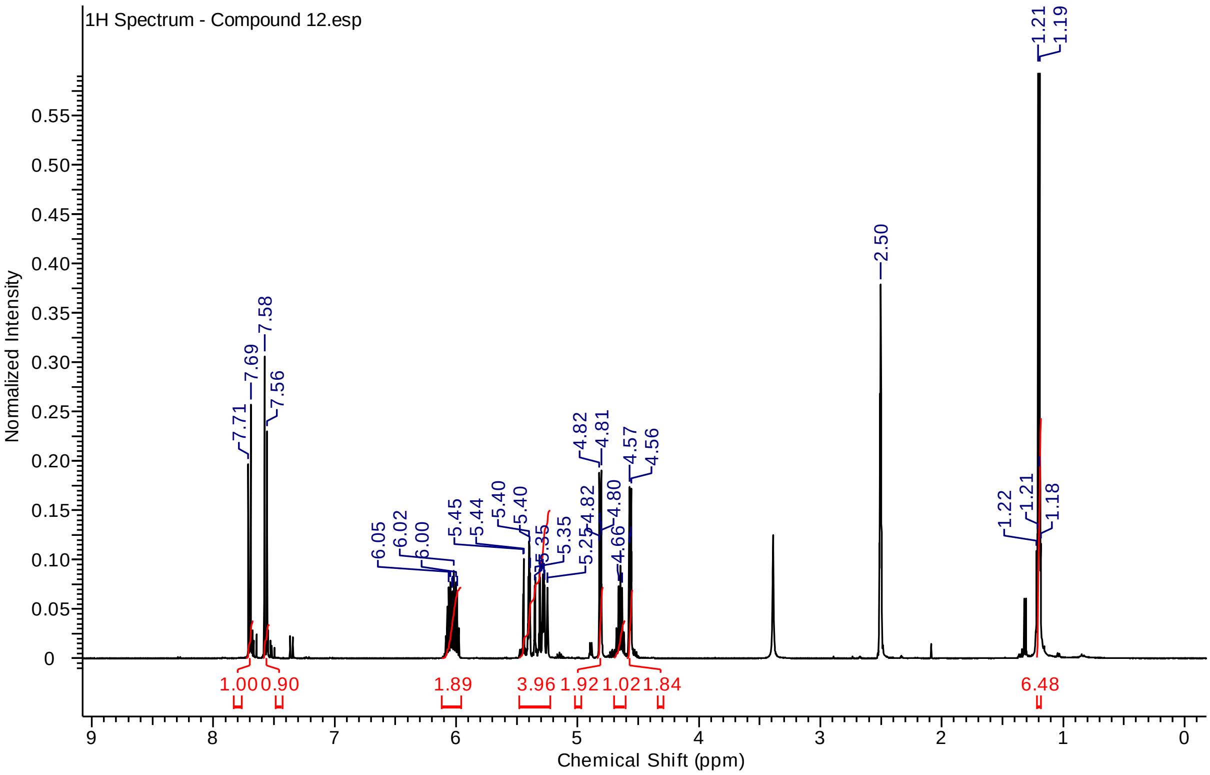
1H Spectrum - Compound 12.esp
Normalized Intensity
Chemical Shift (ppm)
7.71
7.69
7.58
7.56
6.05
6.02
6.00
5.45
5.44
5.40
5.40
5.35
5.35
5.25
4.82
4.82
4.81
4.80
4.66
4.57
4.56
2.50
1.22
1.21
1.21
1.19
1.18
1.00
0.90
1.89
3.96
1.92
1.02
1.84
6.48

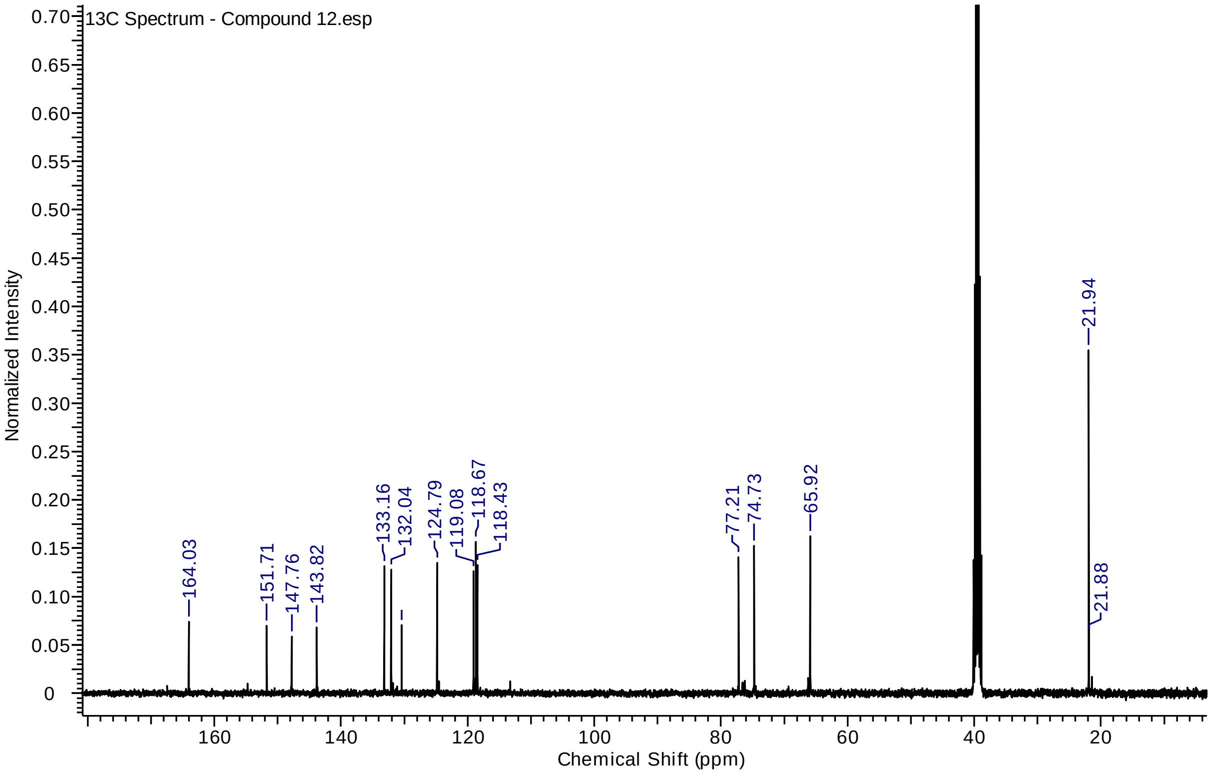
13C Spectrum - Compound 12.esp
Normalized Intensity
Chemical Shift (ppm)
164.03
151.71
147.76
143.82
133.16
132.04
124.79
119.08
118.67
118.43
77.21
74.73
65.92
21.94
21.88

Compound 13

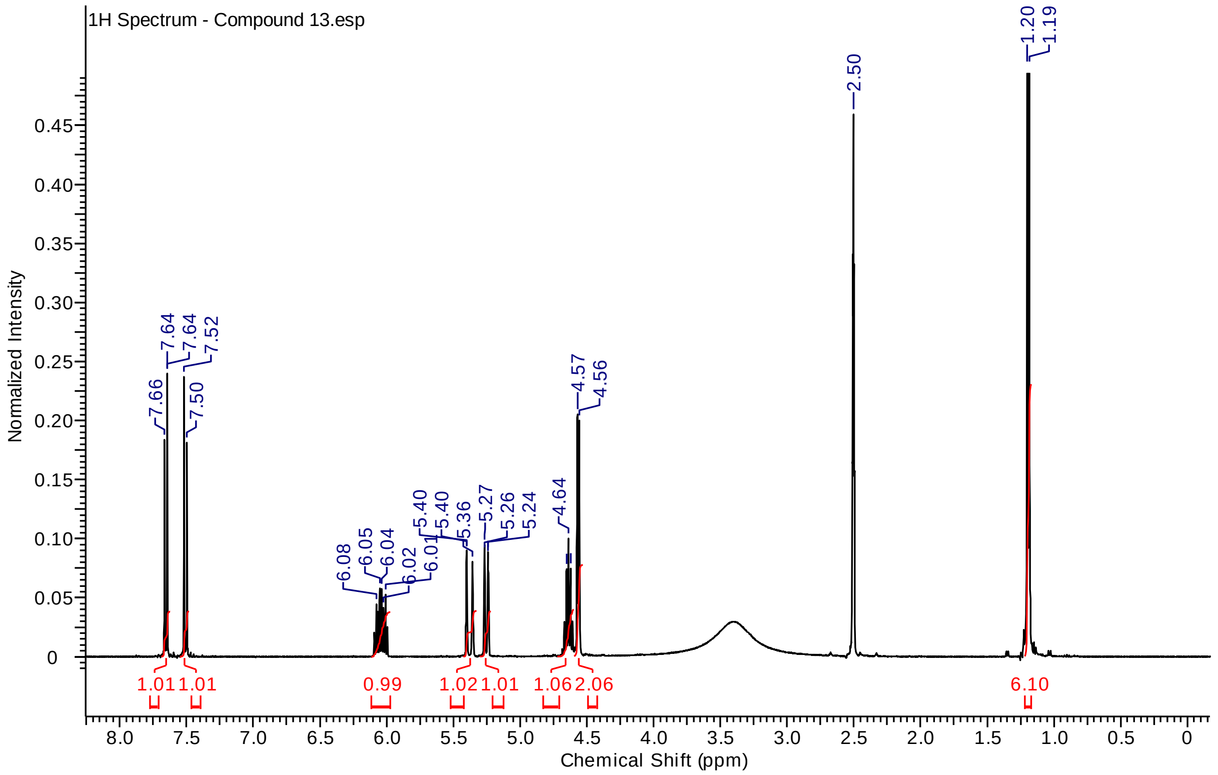
1H Spectrum - Compound 13.esp
Normalized Intensity
Chemical Shift (ppm)
7.66
7.64
7.64
7.52
7.50
6.08
6.05
6.04
6.02
6.01
5.40
5.40
5.36
5.27
5.26
5.24
4.64
4.57
4.56
2.50
1.20
1.19
1.01
1.01
0.99
1.02
1.01
1.06
2.06
6.10

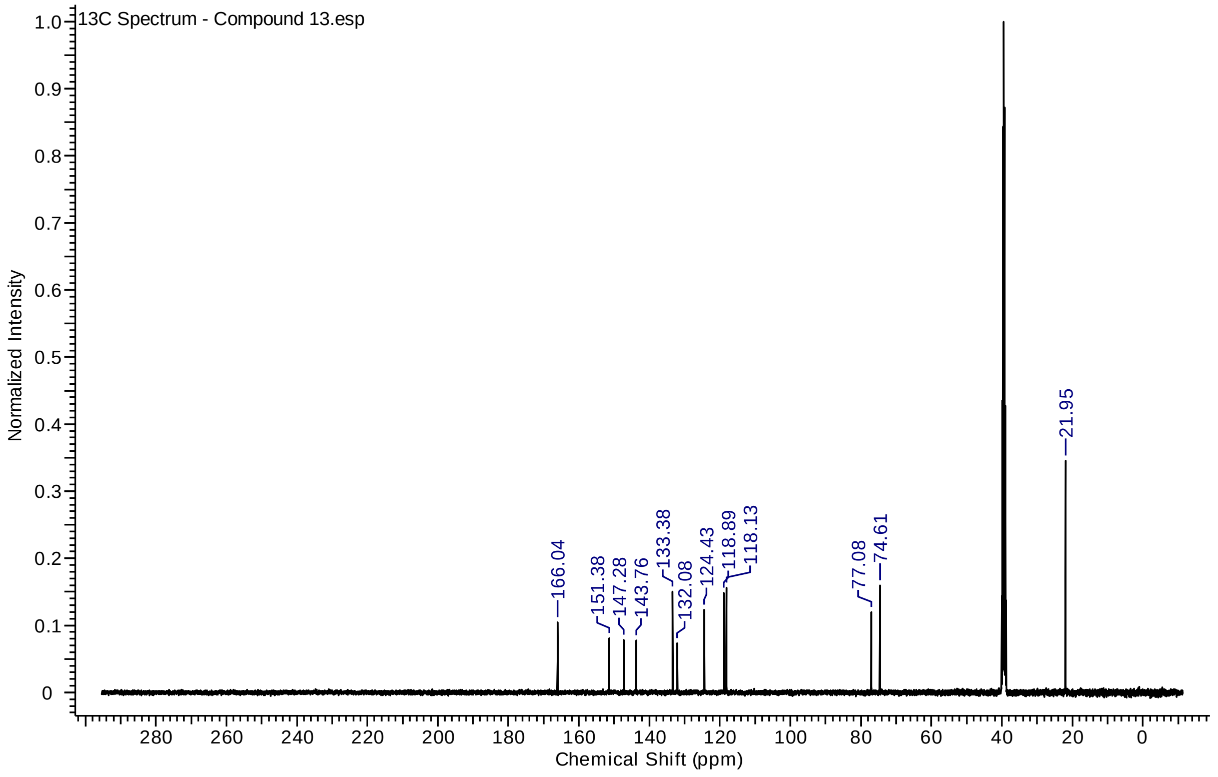
13C Spectrum - Compound 13.esp
Normalized Intensity
Chemical Shift (ppm)
166.04
151.38
147.28
143.76
133.38
132.08
124.43
118.89
118.13
77.08
74.61
21.95

Compound 14

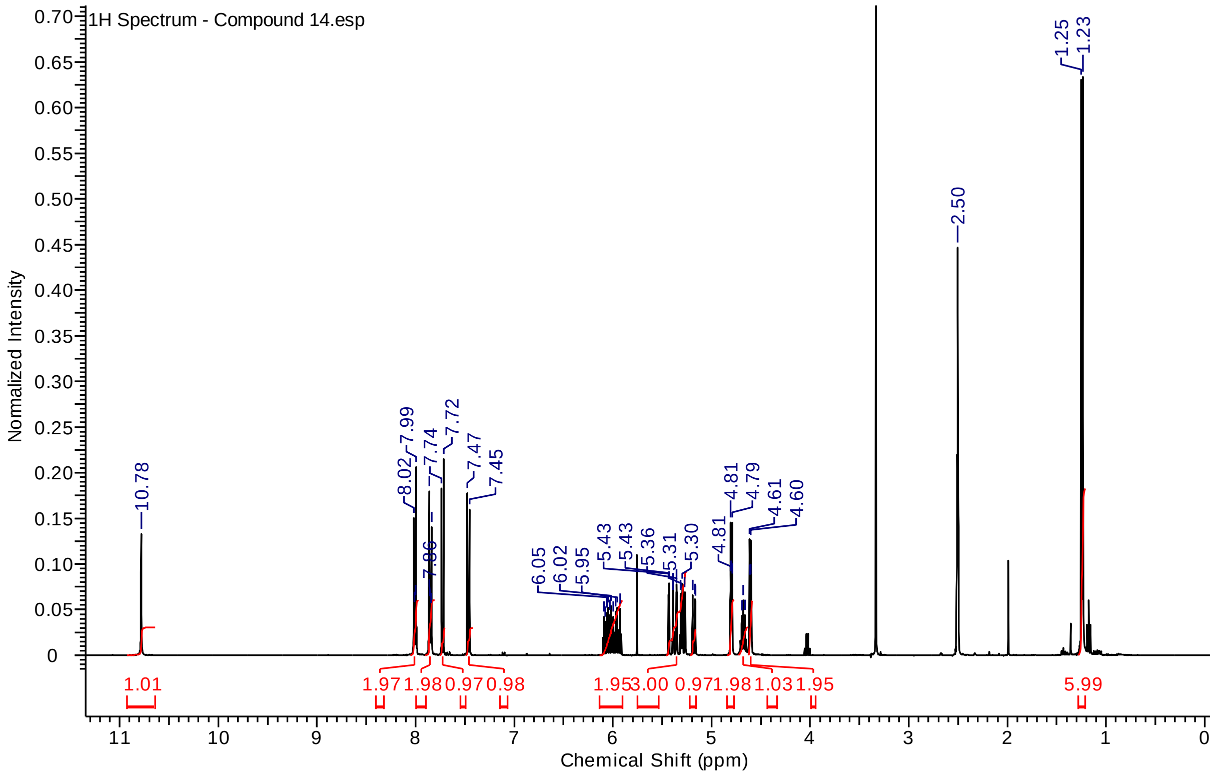
1H Spectrum - Compound 14.esp
Normalized Intensity
Chemical Shift (ppm)
10.78
8.02
7.99
7.86
7.74
7.72
7.47
7.45
6.05
6.02
5.95
5.43
5.43
5.36
5.31
5.30
4.81
4.81
4.79
4.61
4.60
2.50
1.25
1.23
1.01
1.97
1.98
0.97
0.98
1.95
3.00
0.97
1.98
1.03
1.95
5.99

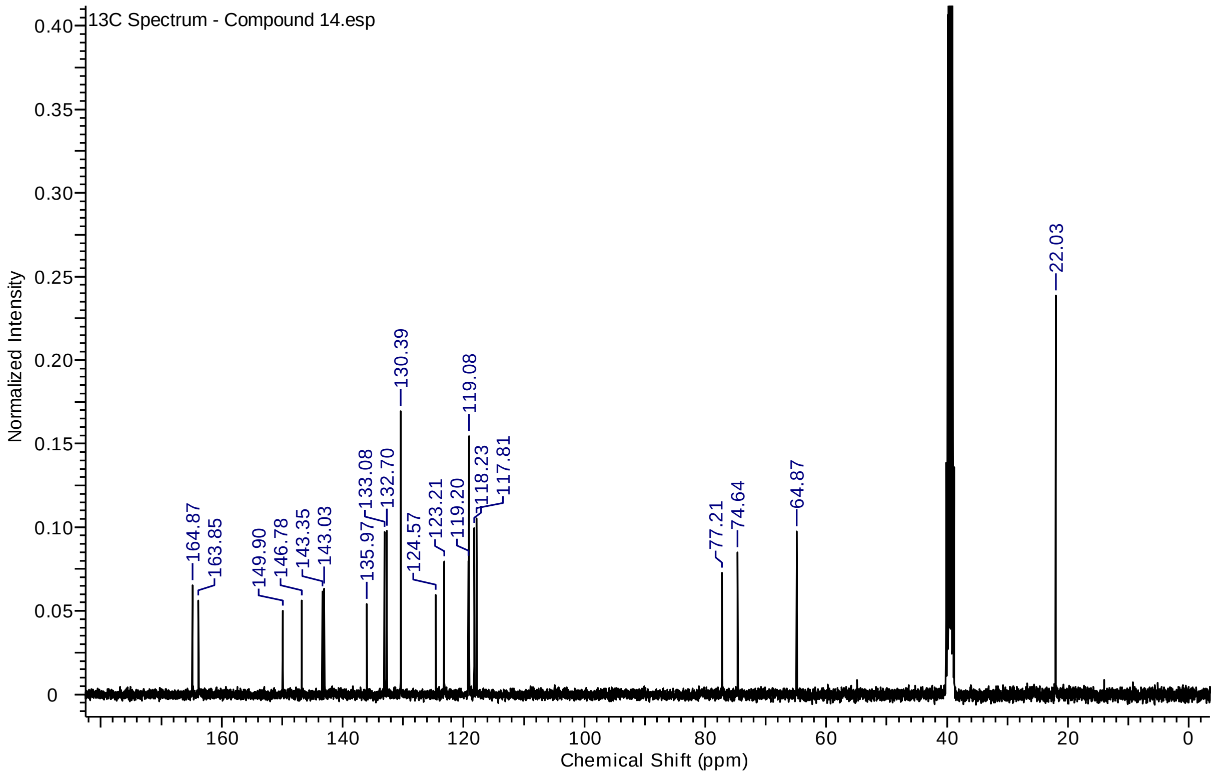
13C Spectrum - Compound 14.esp
Normalized Intensity
Chemical Shift (ppm)
164.87
163.85
149.90
146.78
143.35
143.03
135.97
133.08
132.70
130.39
124.57
123.21
119.20
119.08
118.23
117.81
77.21
74.64
64.87
22.03

Compound 15

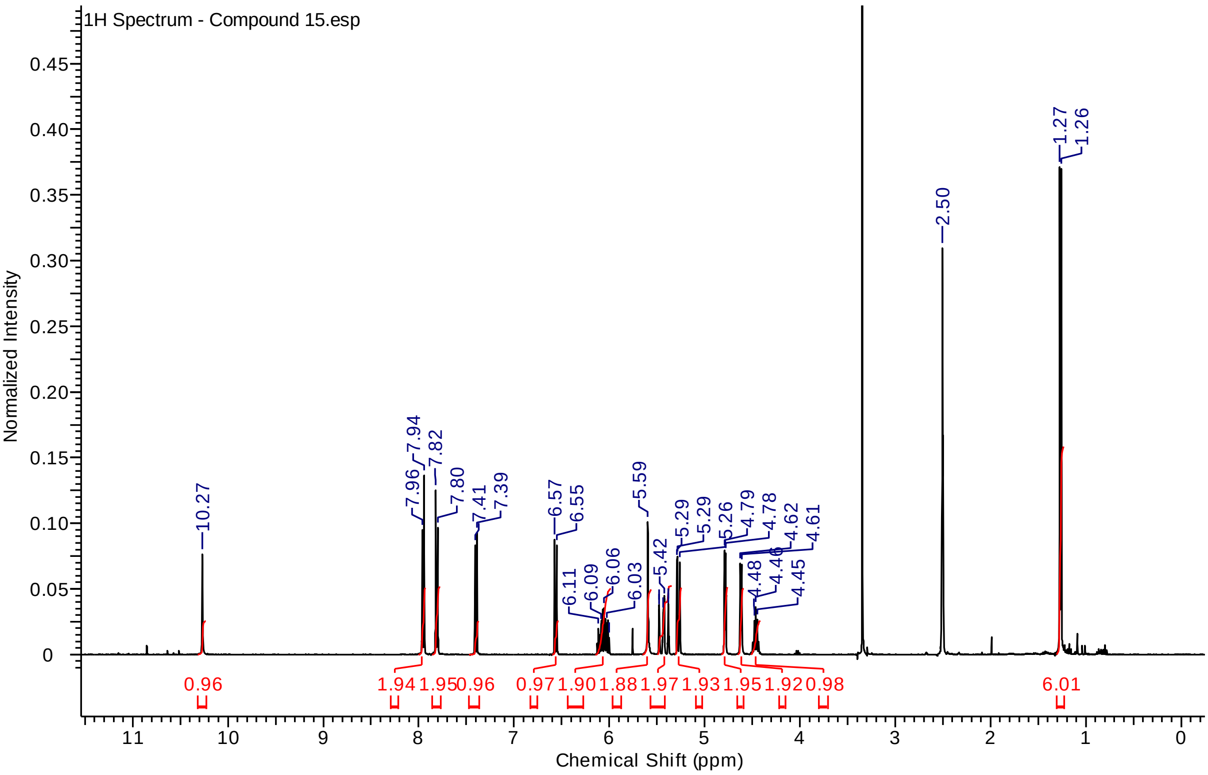

1H Spectrum - Compound 15.esp
Normalized Intensity
Chemical Shift (ppm)
10.27
7.96
7.94
7.82
7.80
7.41
7.39
6.57
6.55
6.11
6.09
6.06
6.03
5.59
5.42
5.29
5.29
5.26
4.79
4.78
4.62
4.61
4.48
4.46
4.45
2.50
1.27
1.26
0.96
1.94
1.95
0.96
0.97
1.90
1.88
1.97
1.93
1.95
1.92
0.98
6.01

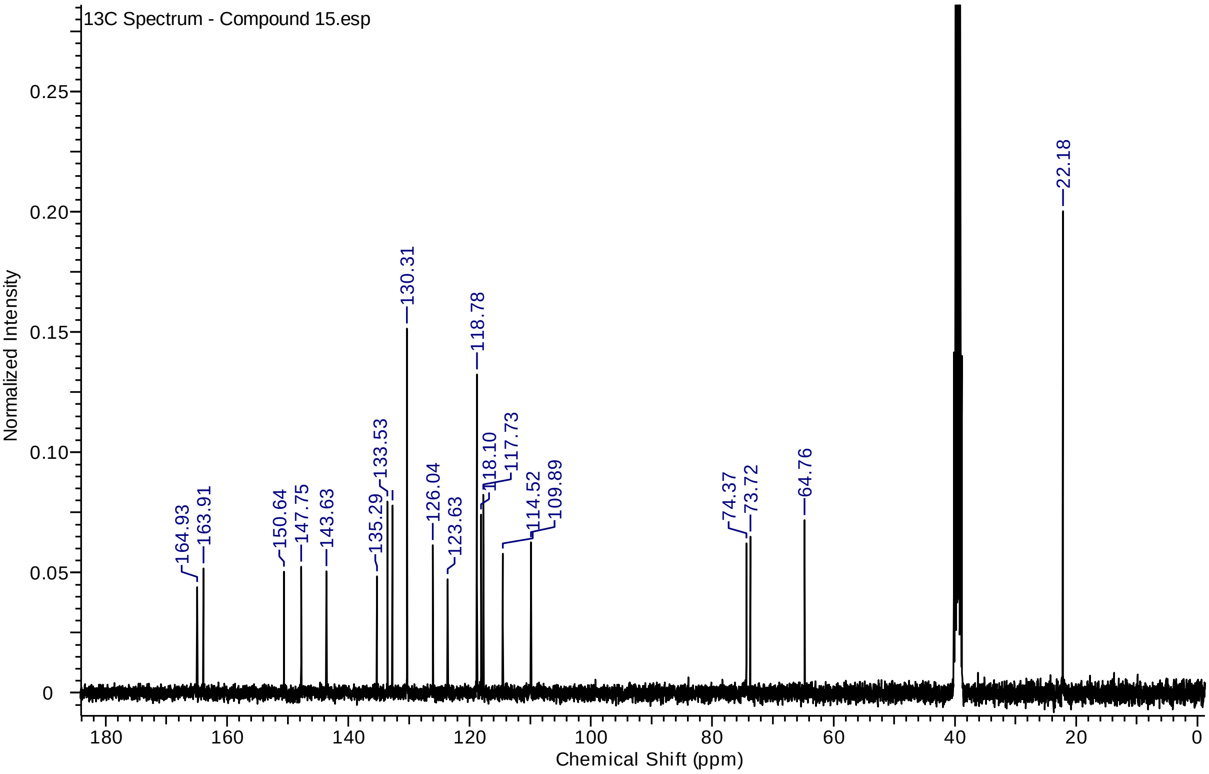

13C Spectrum - Compound 15.esp
Normalized Intensity
Chemical Shift (ppm)
164.93
163.91
150.64
147.75
143.63
135.29
133.53
130.31
126.04
123.63
118.78
118.10
117.73
114.52
109.89
74.37
73.72
64.76
22.18

Compound 17

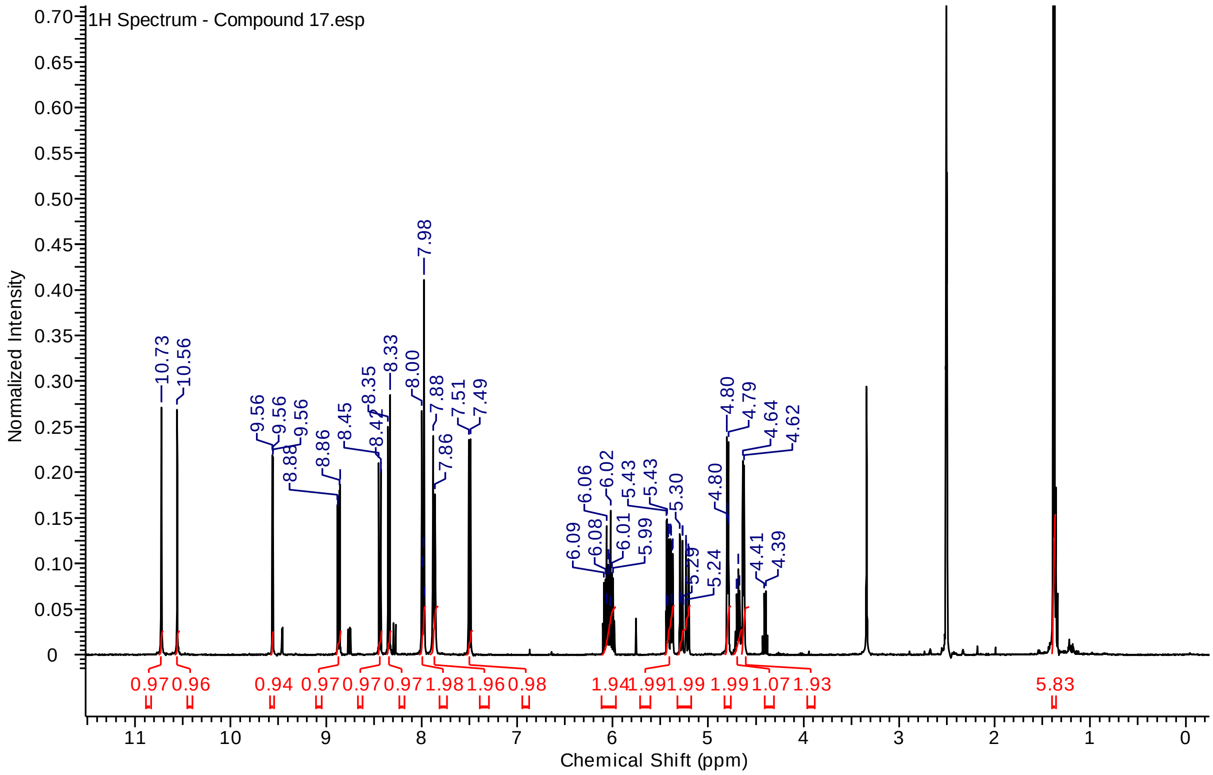
1H Spectrum - Compound 17.esp
Normalized Intensity
Chemical Shift (ppm)
10.73
10.56
9.56
9.56
9.56
8.88
8.86
8.45
8.42
8.35
8.33
8.00
7.98
7.88
7.86
7.51
7.49
6.09
6.08
6.06
6.02
6.01
5.99
5.43
5.43
5.30
5.29
5.24
4.80
4.80
4.79
4.64
4.62
4.41
4.39
0.97
0.96
0.94
0.97
0.97
0.97
1.98
1.96
0.98
1.94
1.99
1.99
1.99
1.07
1.93
5.83

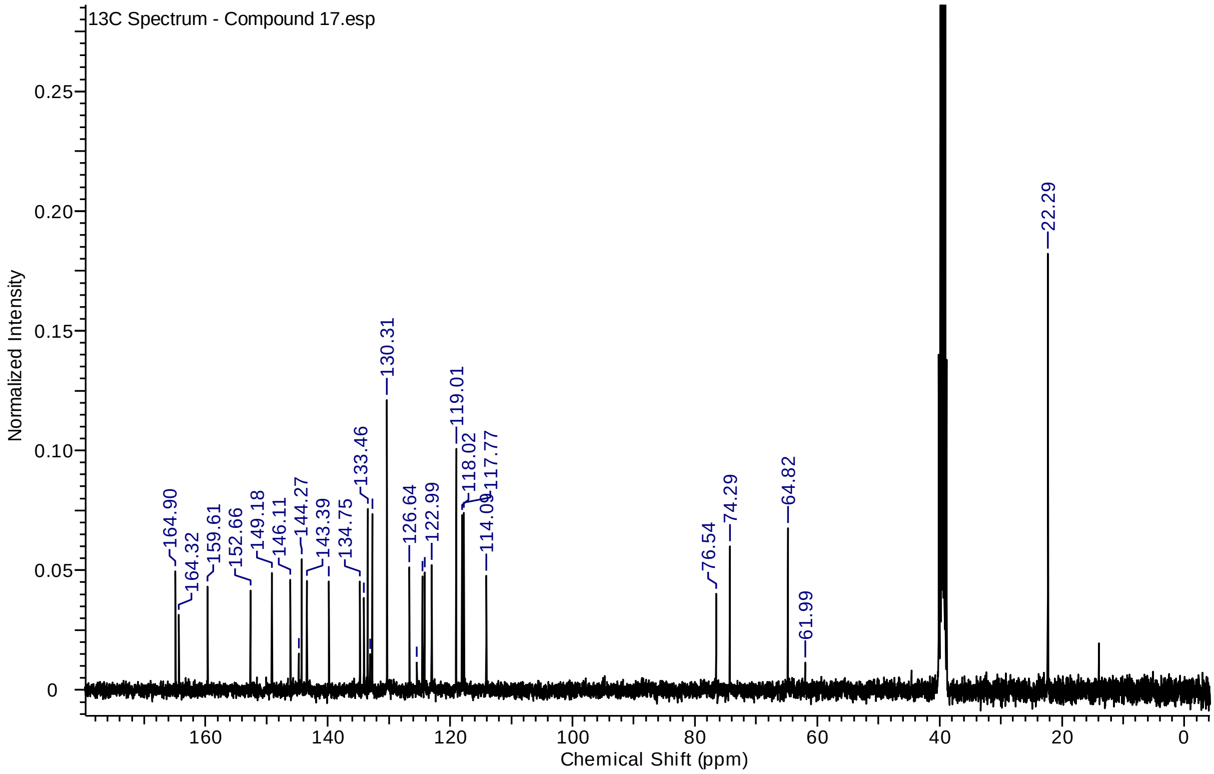
13C Spectrum - Compound 17.esp
Normalized Intensity
Chemical Shift (ppm)
164.90
164.32
159.61
152.66
149.18
146.11
144.27
143.39
134.75
133.46
130.31
126.64
122.99
119.01
118.02
117.77
114.09
76.54
74.29
64.82
61.99
22.29

Compound 18

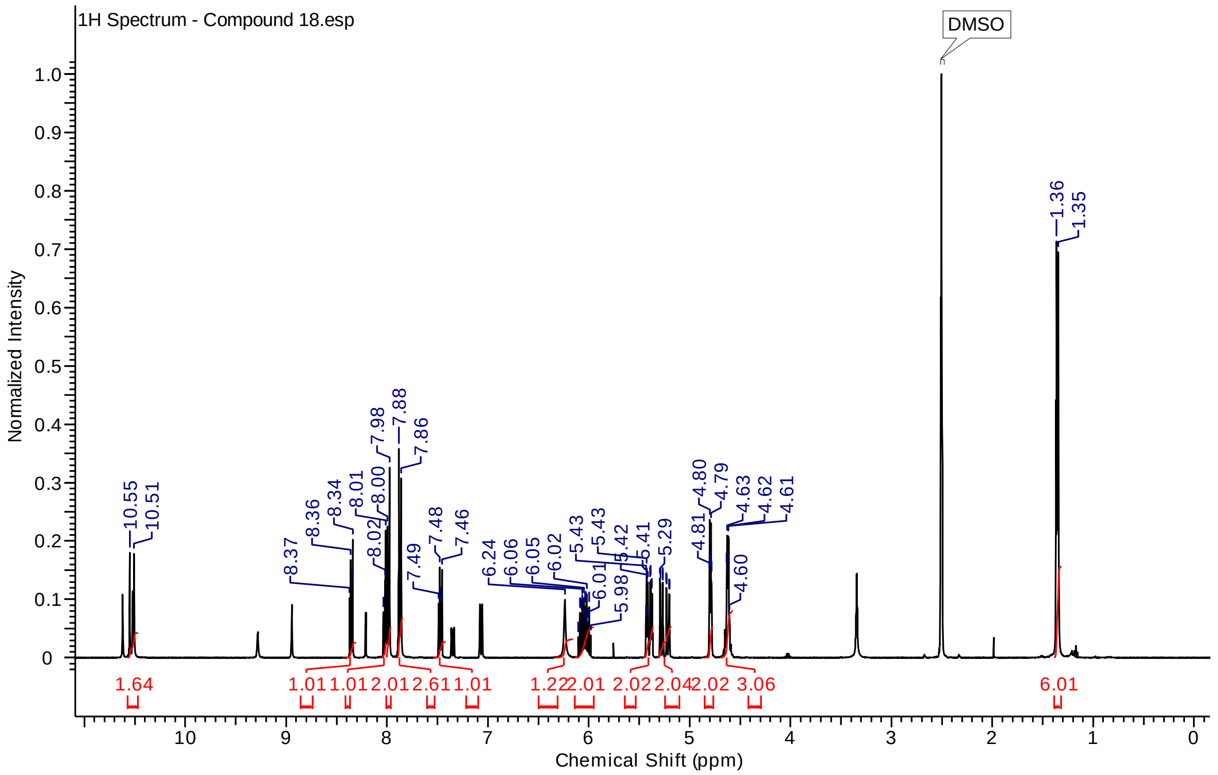

1H Spectrum - Compound 18.esp
DMSO
Normalized Intensity
Chemical Shift (ppm)
10.55
10.51
8.37
8.36
8.34
8.02
8.01
8.00
7.98
7.88
7.86
7.49
7.48
7.46
6.24
6.06
6.05
6.02
6.01
5.98
5.43
5.43
5.42
5.41
5.29
4.81
4.80
4.79
4.63
4.62
4.61
4.60
1.36
1.35
1.64
1.01
1.01
2.01
2.61
1.01
1.22
2.01
2.02
2.04
2.02
3.06
6.01

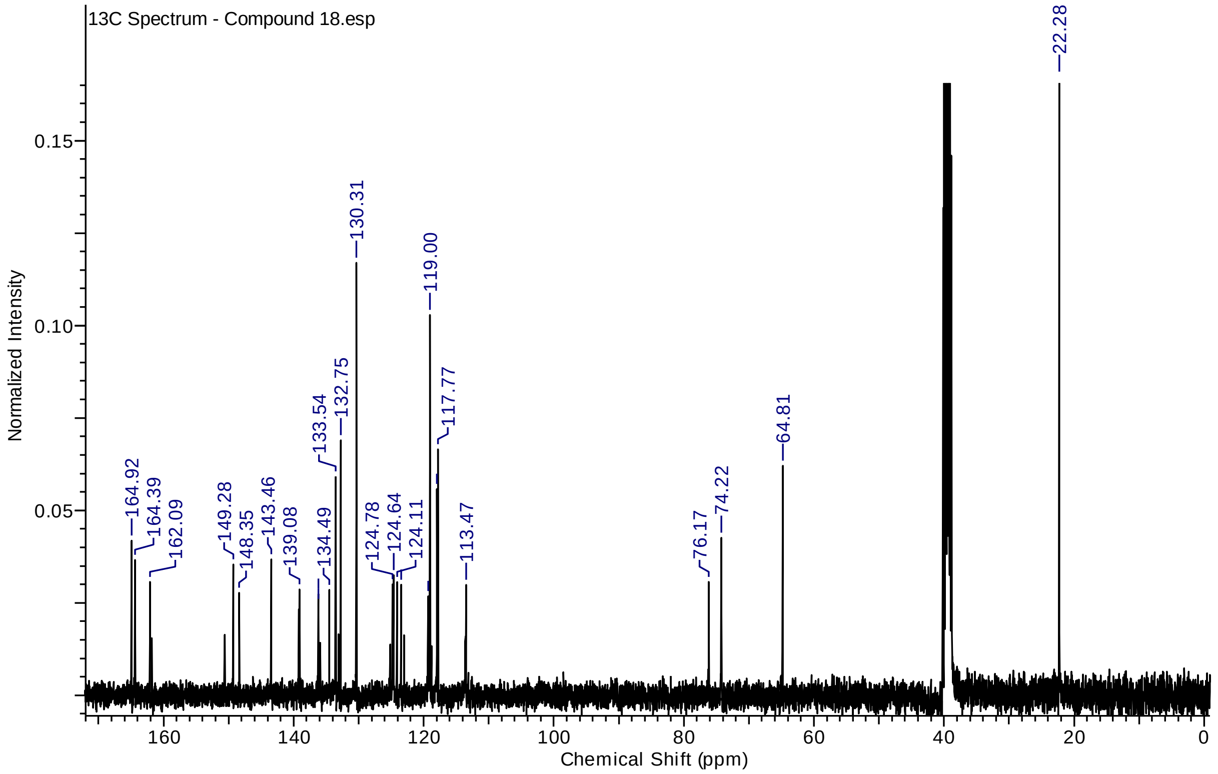

13C Spectrum - Compound 18.esp
Normalized Intensity
Chemical Shift (ppm)
164.92
164.39
162.09
149.28
148.35
143.46
139.08
134.49
133.54
132.75
130.31
124.78
124.64
124.11
119.00
117.77
113.47
76.17
74.22
64.81
22.28

Compound 19

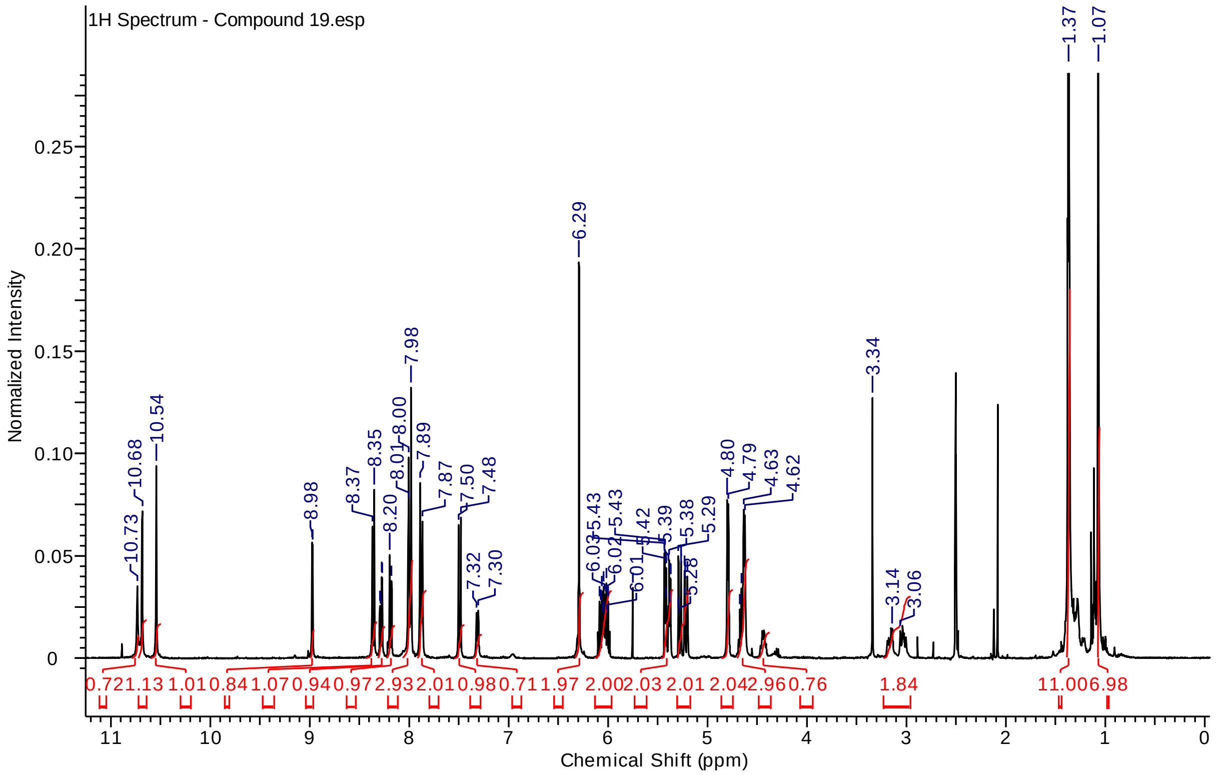

1H Spectrum - Compound 19.esp
Normalized Intensity
Chemical Shift (ppm)

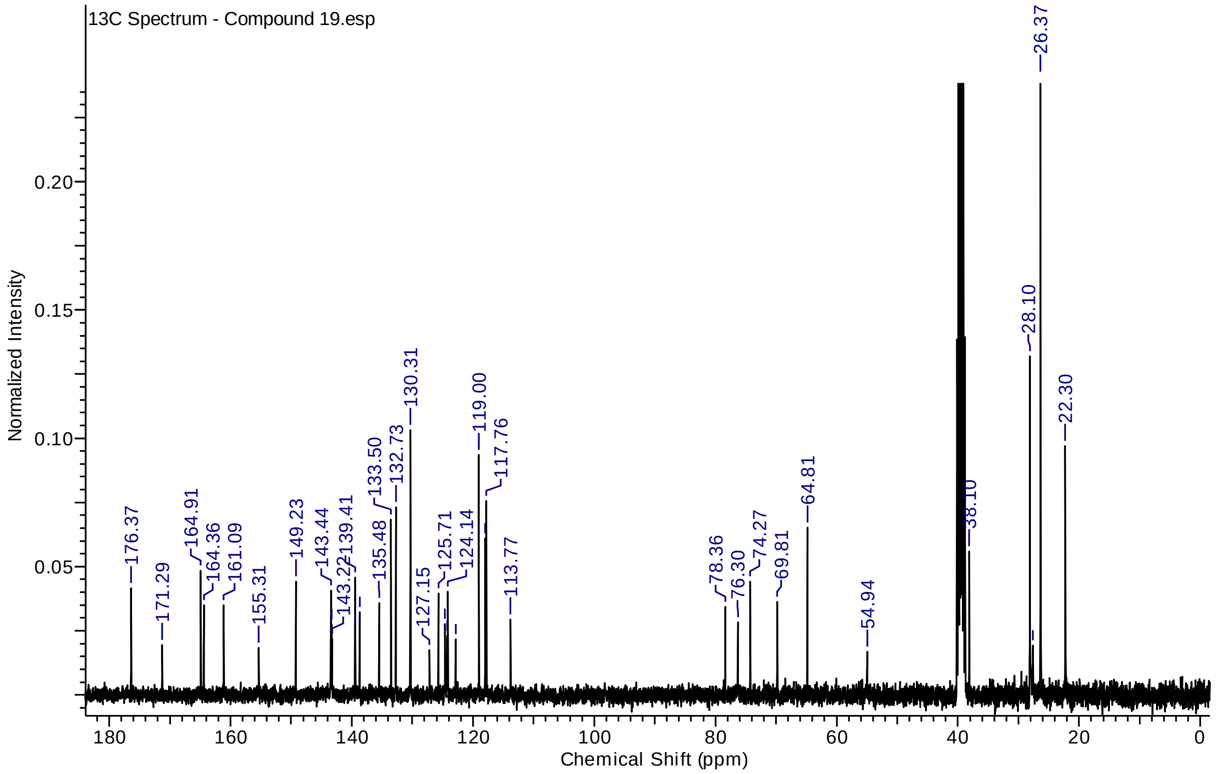

13C Spectrum - Compound 19.esp
Normalized Intensity
Chemical Shift (ppm)

Compound 20

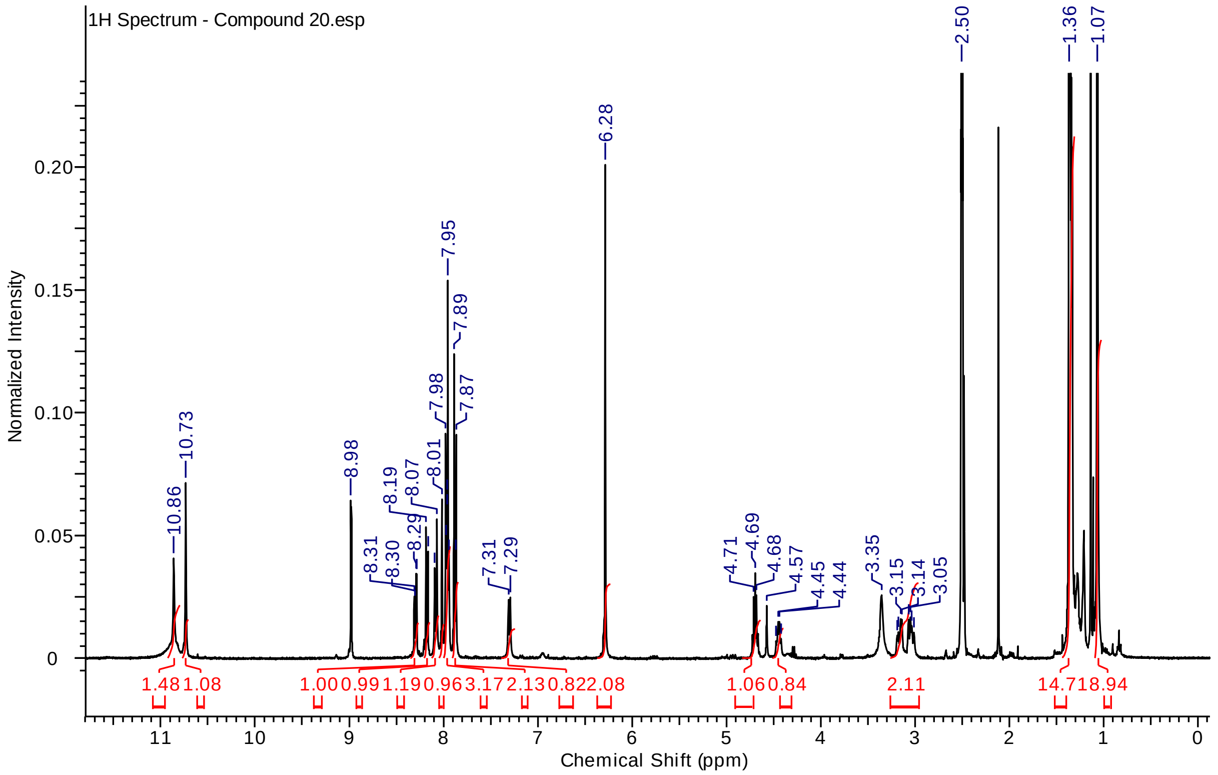
1H Spectrum - Compound 20.esp
Normalized Intensity
Chemical Shift (ppm)
10.86
10.73
8.98
8.31
8.30
8.29
8.19
8.07
8.01
7.98
7.95
7.89
7.87
7.31
7.29
6.28
4.71
4.69
4.68
4.57
4.45
4.44
3.35
3.15
3.14
3.05
2.50
1.36
1.07
1.48
1.08
1.00
0.99
1.19
0.96
3.17
2.13
0.82
2.08
1.06
0.84
2.11
14.71
8.94

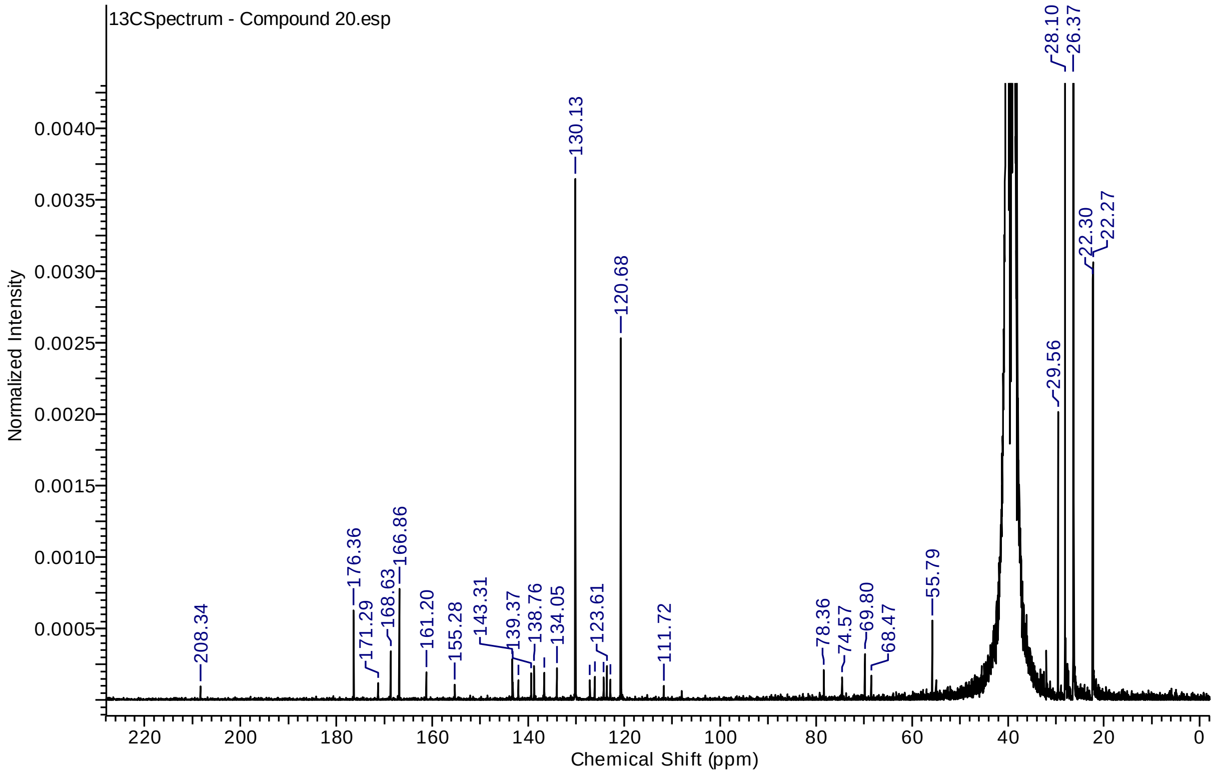
13CSpectrum - Compound 20.esp
Normalized Intensity
Chemical Shift (ppm)
208.34
176.36
171.29
168.63
166.86
161.20
155.28
143.31
139.37
138.76
134.05
130.13
123.61
120.68
111.72
78.36
74.57
69.80
68.47
55.79
29.56
28.10
26.37
22.30
22.27

Compound 21

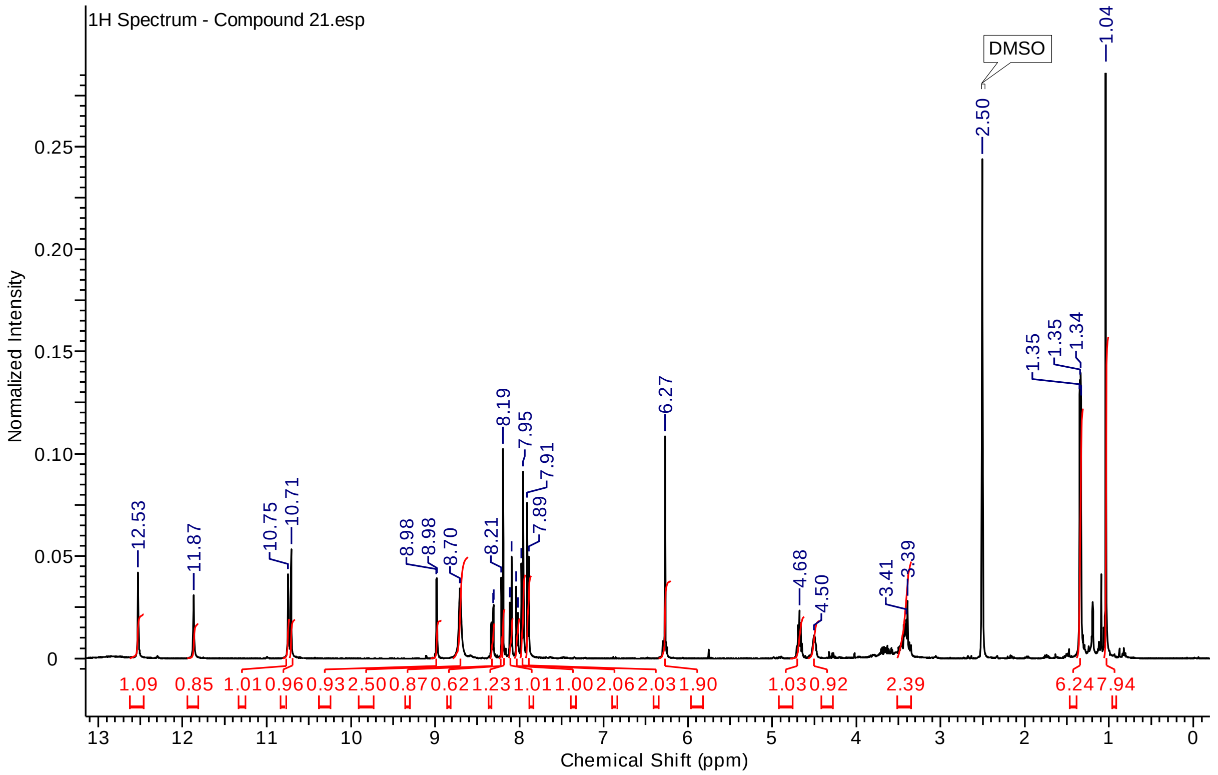
1H Spectrum - Compound 21.esp
DMSO
Normalized Intensity
Chemical Shift (ppm)

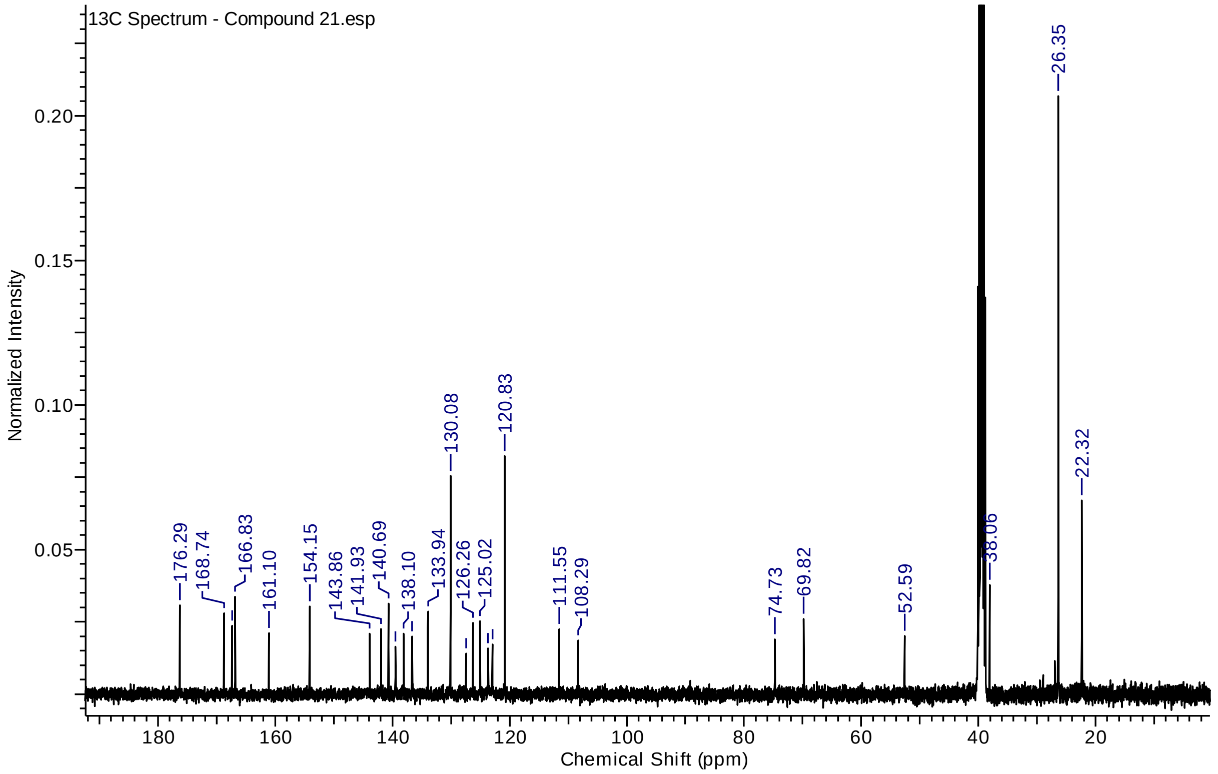
13C Spectrum - Compound 21.esp
Normalized Intensity
Chemical Shift (ppm)

Albi-1

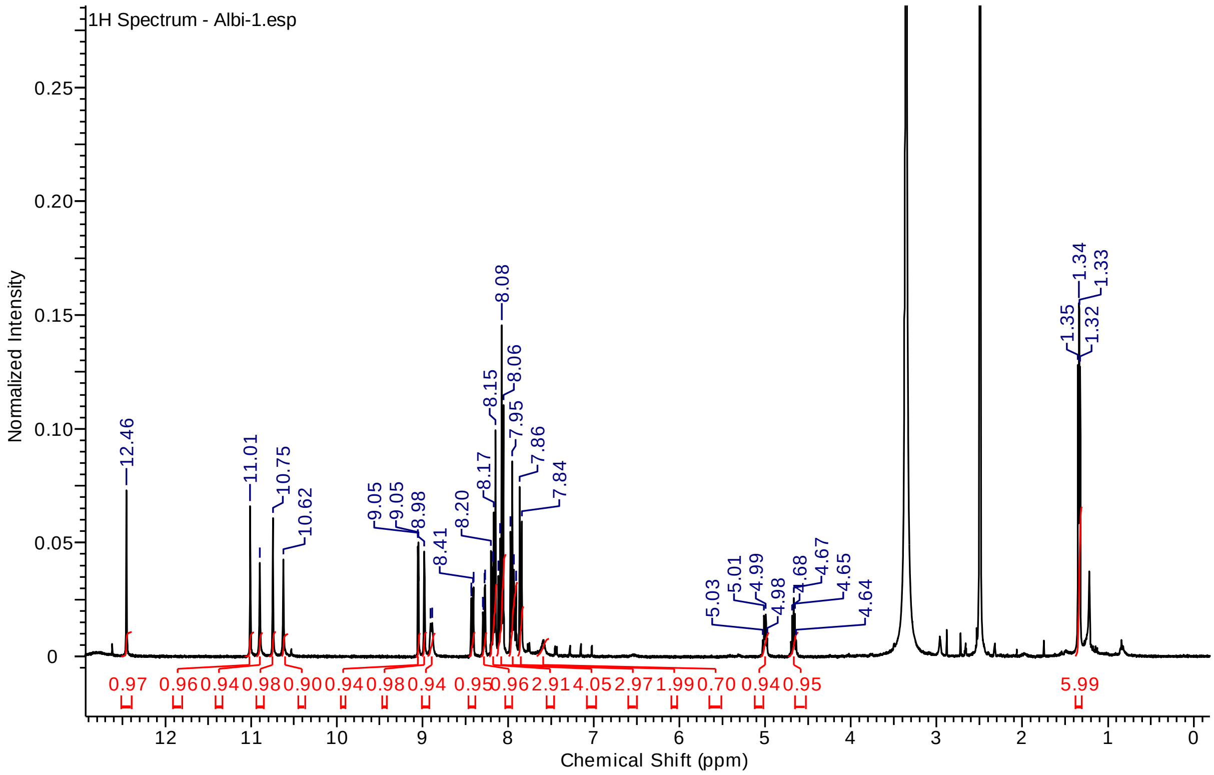

(^{1}H, ^{13}C)-HMBC - Albi-1

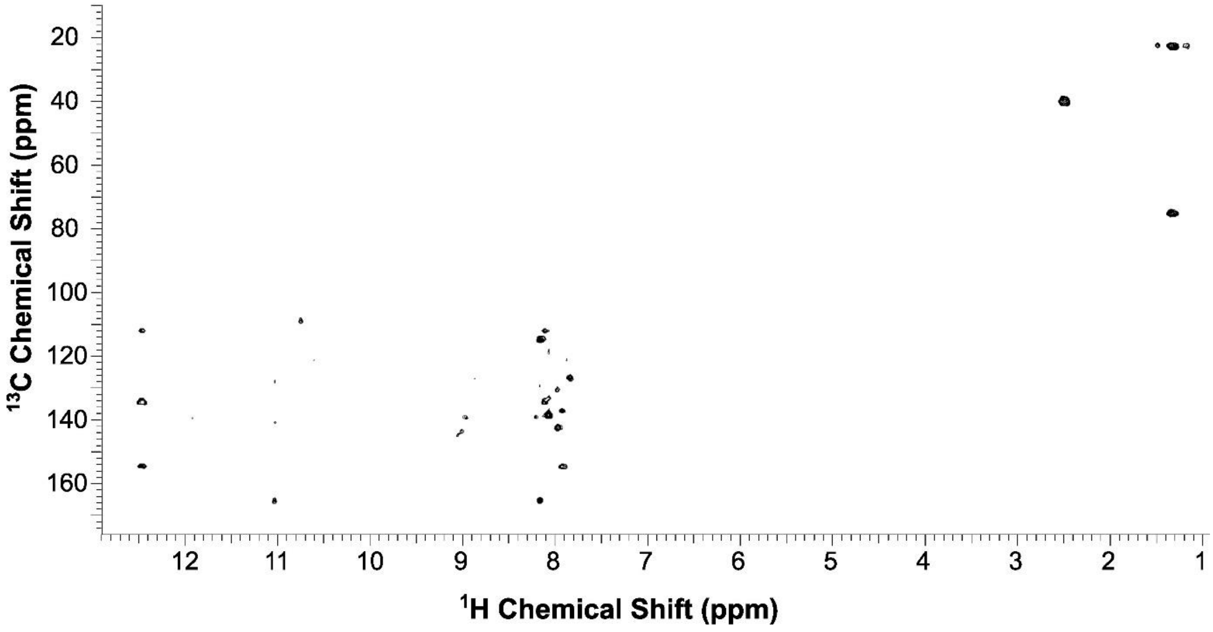

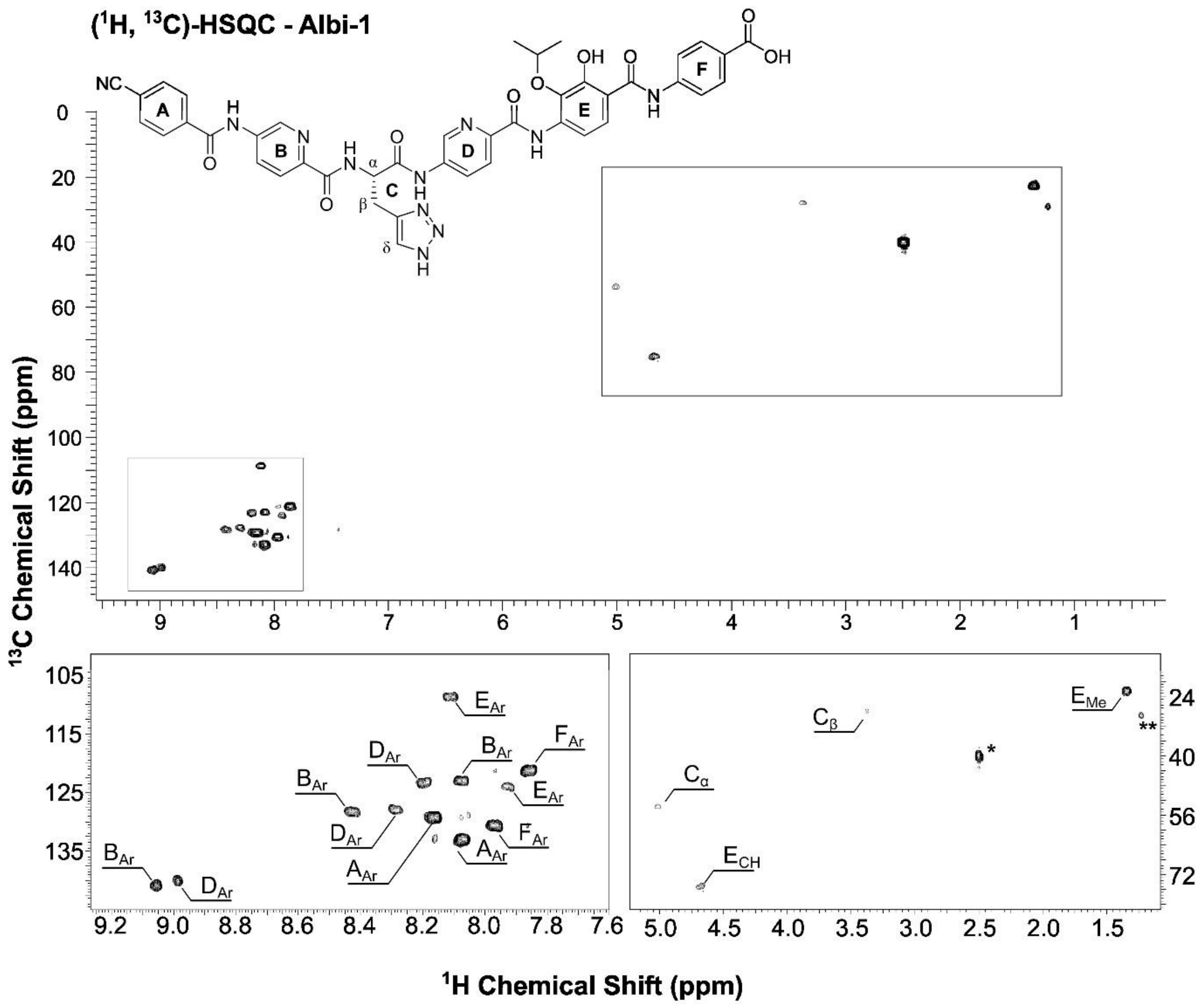

^{1}H-^{13}C-HSQC with magnification of relevant sections and annotations of signals (Ar: Aromatic, Me: Methyl, *: DMSO, ** impurity).

Compound 24

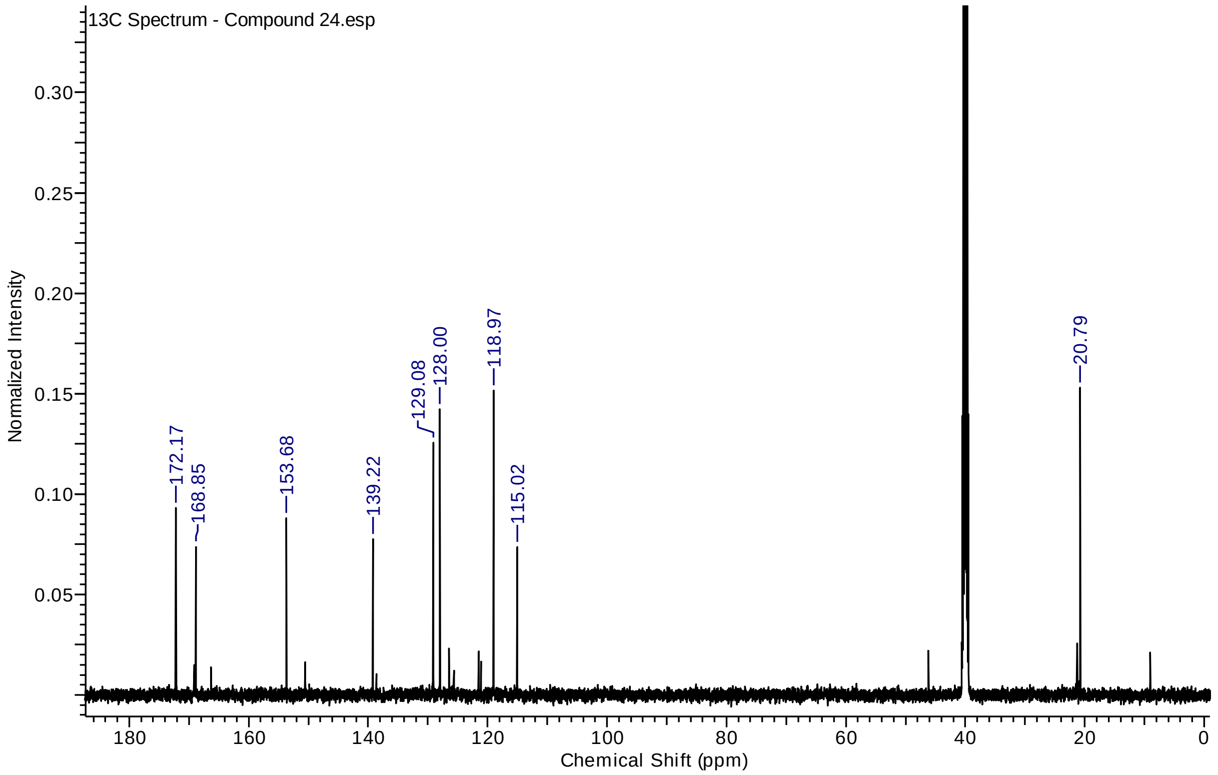

13C Spectrum - Compound 24.esp
Normalized Intensity
Chemical Shift (ppm)
172.17
168.85
153.68
139.22
129.08
128.00
118.97
115.02
20.79

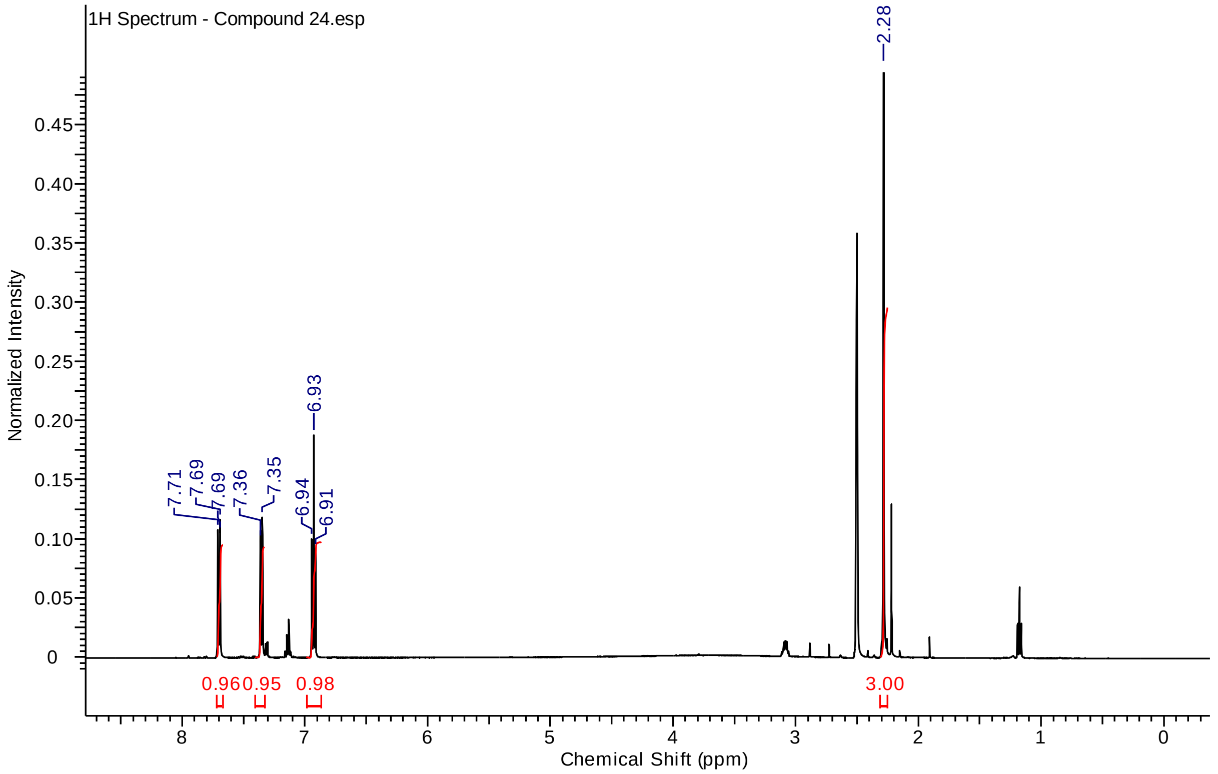

1H Spectrum - Compound 24.esp
Normalized Intensity
Chemical Shift (ppm)
7.71
7.69
7.69
7.36
7.35
6.94
6.93
6.91
2.28
0.96
0.95
0.98
3.00

Compound 25

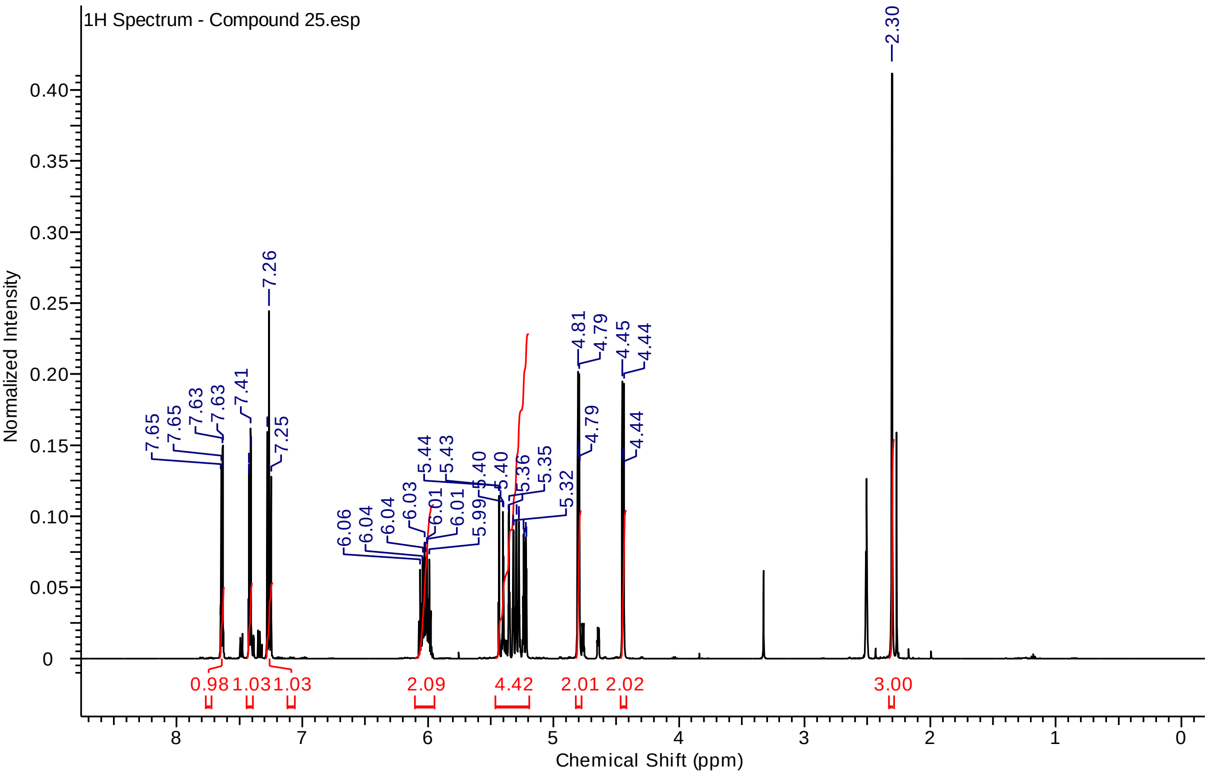
1H Spectrum - Compound 25.esp
Normalized Intensity
Chemical Shift (ppm)
7.65
7.65
7.63
7.63
7.41
7.26
7.25
6.06
6.04
6.04
6.03
6.01
6.01
5.99
5.44
5.43
5.40
5.40
5.36
5.35
5.32
4.81
4.79
4.79
4.45
4.44
4.44
2.30
0.98
1.03
1.03
2.09
4.42
2.01
2.02
3.00

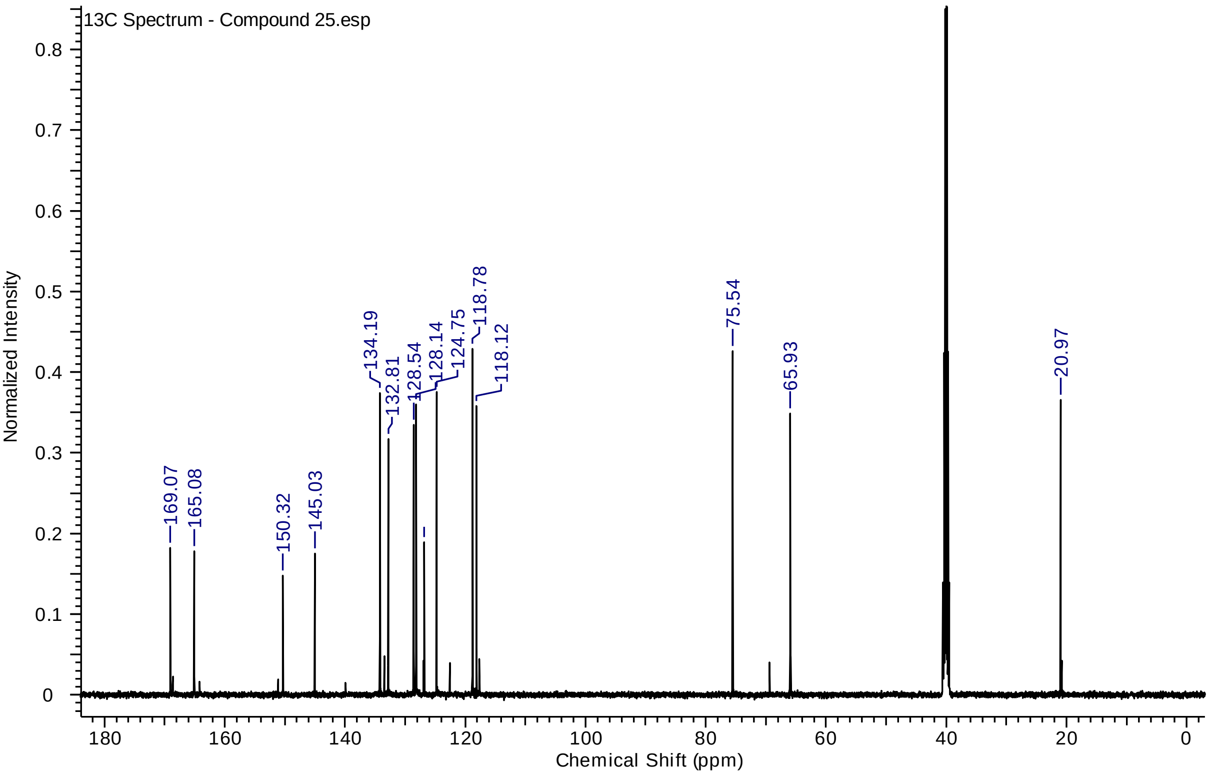
13C Spectrum - Compound 25.esp
Normalized Intensity
Chemical Shift (ppm)
169.07
165.08
150.32
145.03
134.19
132.81
128.54
128.14
124.75
118.78
118.12
75.54
65.93
20.97

Compound 26

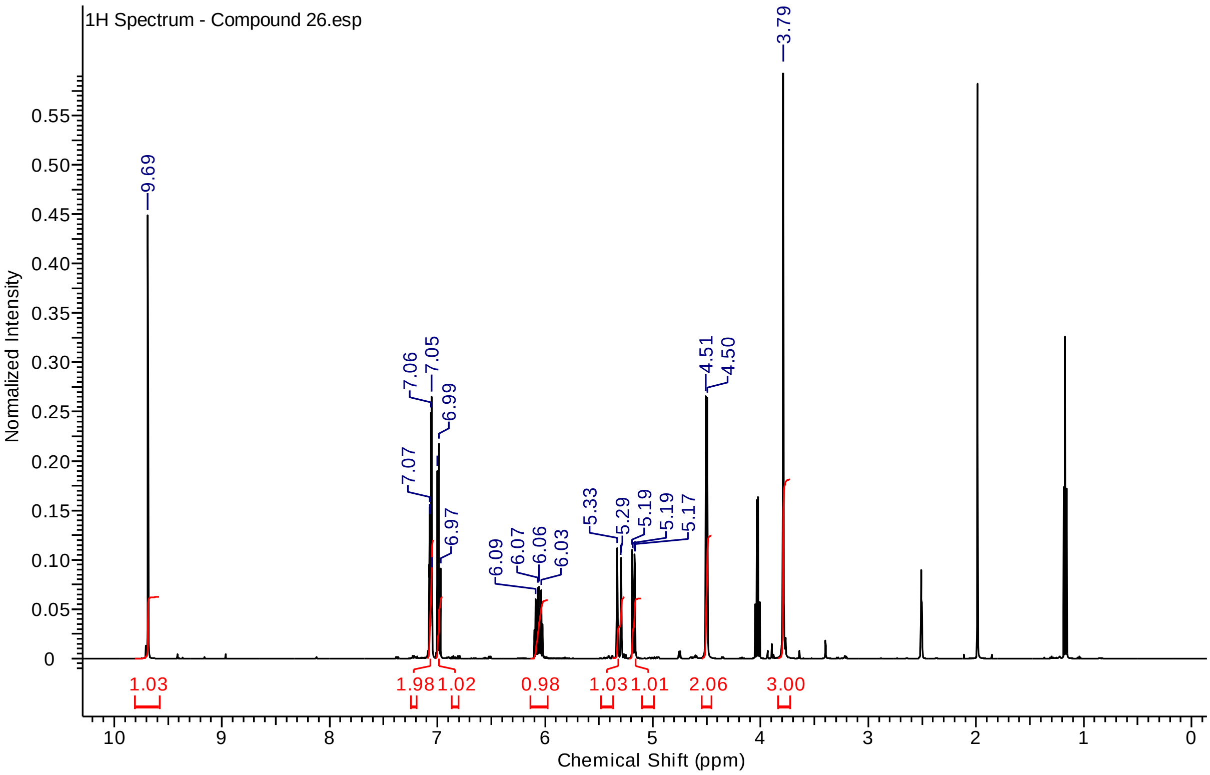
1H Spectrum - Compound 26.esp
Normalized Intensity
Chemical Shift (ppm)
9.69
7.07
7.06
7.05
6.99
6.97
6.09
6.07
6.06
6.03
5.33
5.29
5.19
5.19
5.17
4.51
4.50
3.79
1.03
1.98
1.02
0.98
1.03
1.01
2.06
3.00

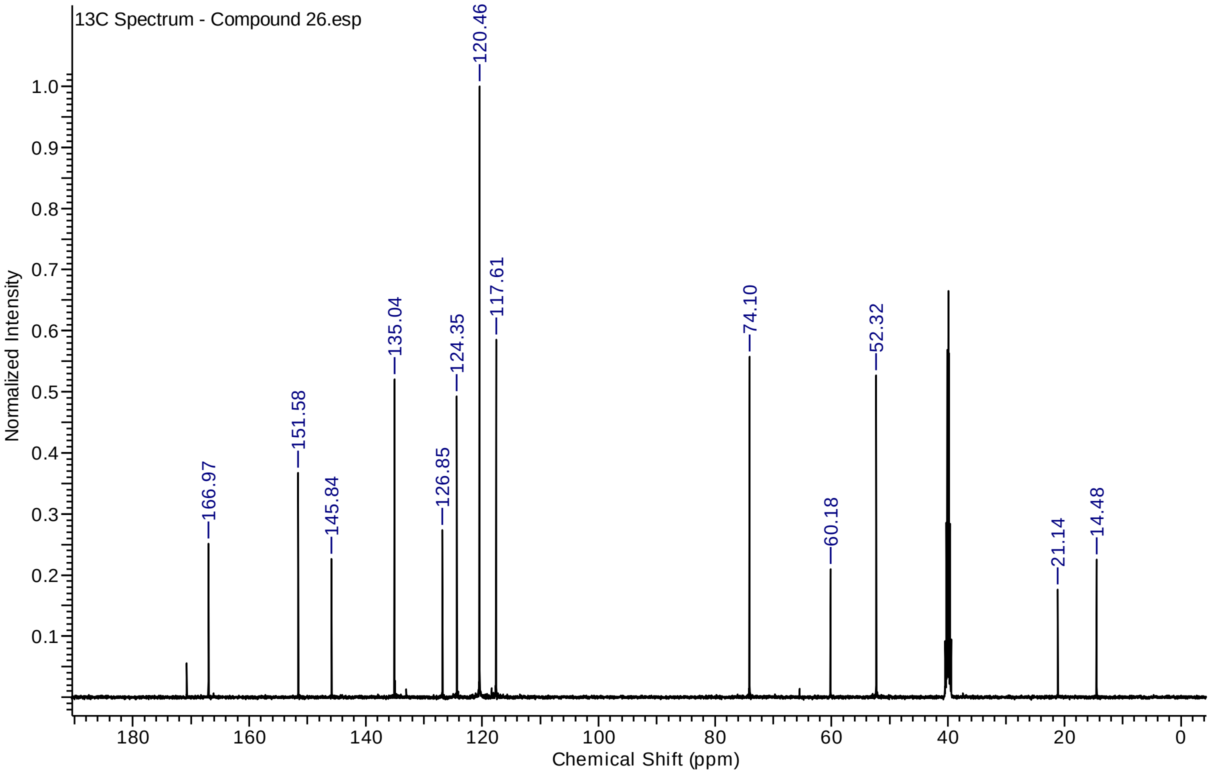
13C Spectrum - Compound 26.esp
Normalized Intensity
Chemical Shift (ppm)
166.97
151.58
145.84
135.04
126.85
124.35
120.46
117.61
74.10
60.18
52.32
21.14
14.48

Compound 27

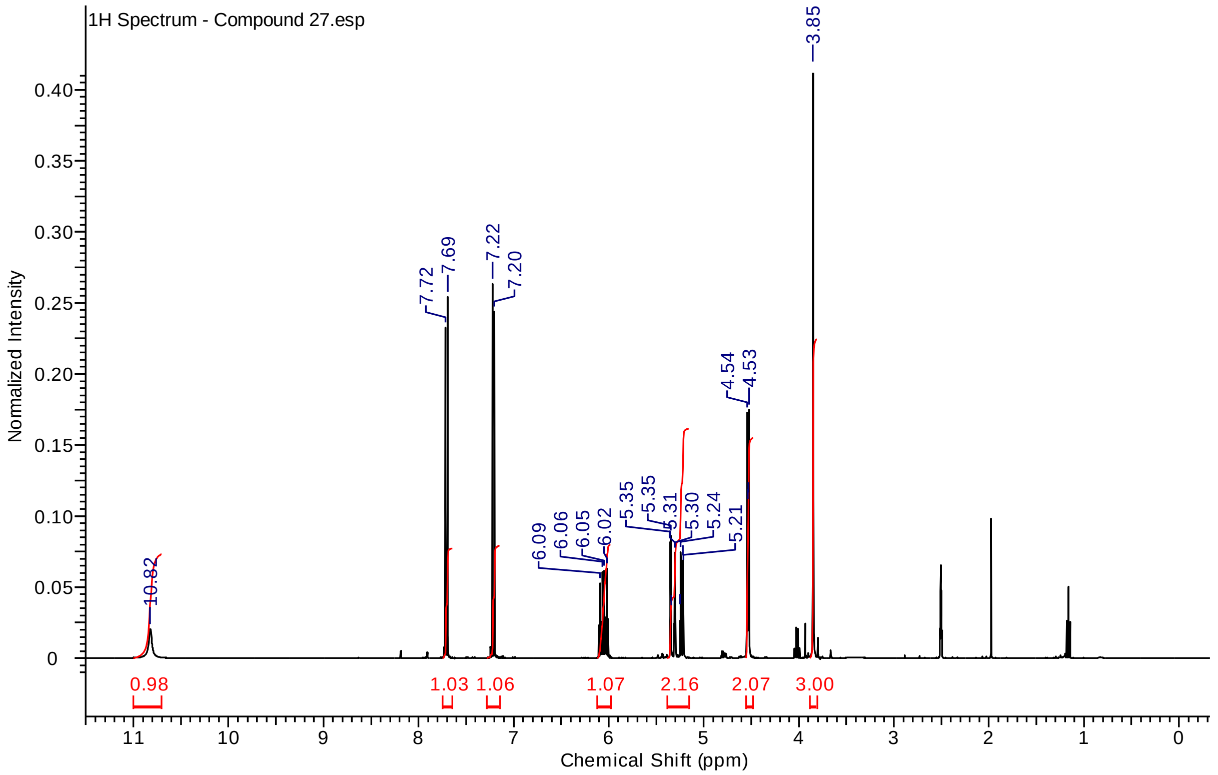
1H Spectrum - Compound 27.esp
Normalized Intensity
Chemical Shift (ppm)
10.82
7.72
7.69
7.22
7.20
6.09
6.06
6.05
6.02
5.35
5.35
5.31
5.30
5.24
5.21
4.54
4.53
3.85
0.98
1.03
1.06
1.07
2.16
2.07
3.00

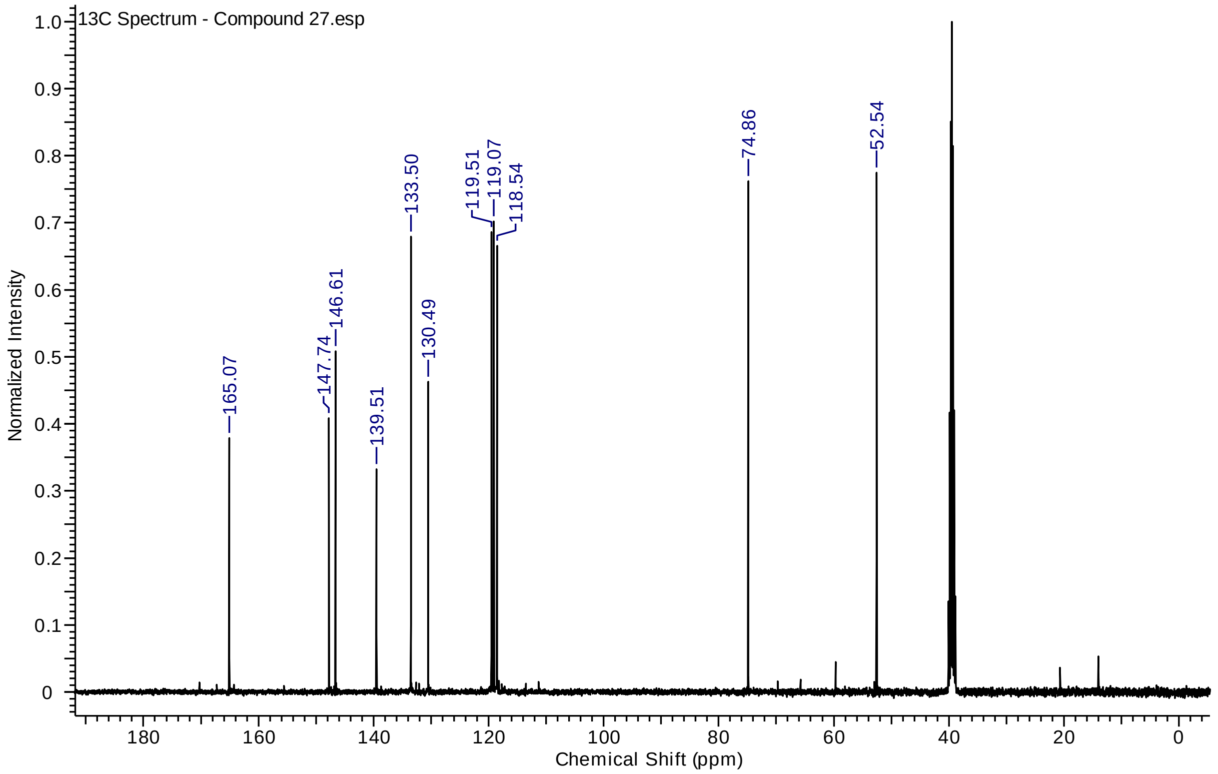
13C Spectrum - Compound 27.esp
Normalized Intensity
Chemical Shift (ppm)
165.07
147.74
146.61
139.51
133.50
130.49
119.51
119.07
118.54
74.86
52.54

Compound 28

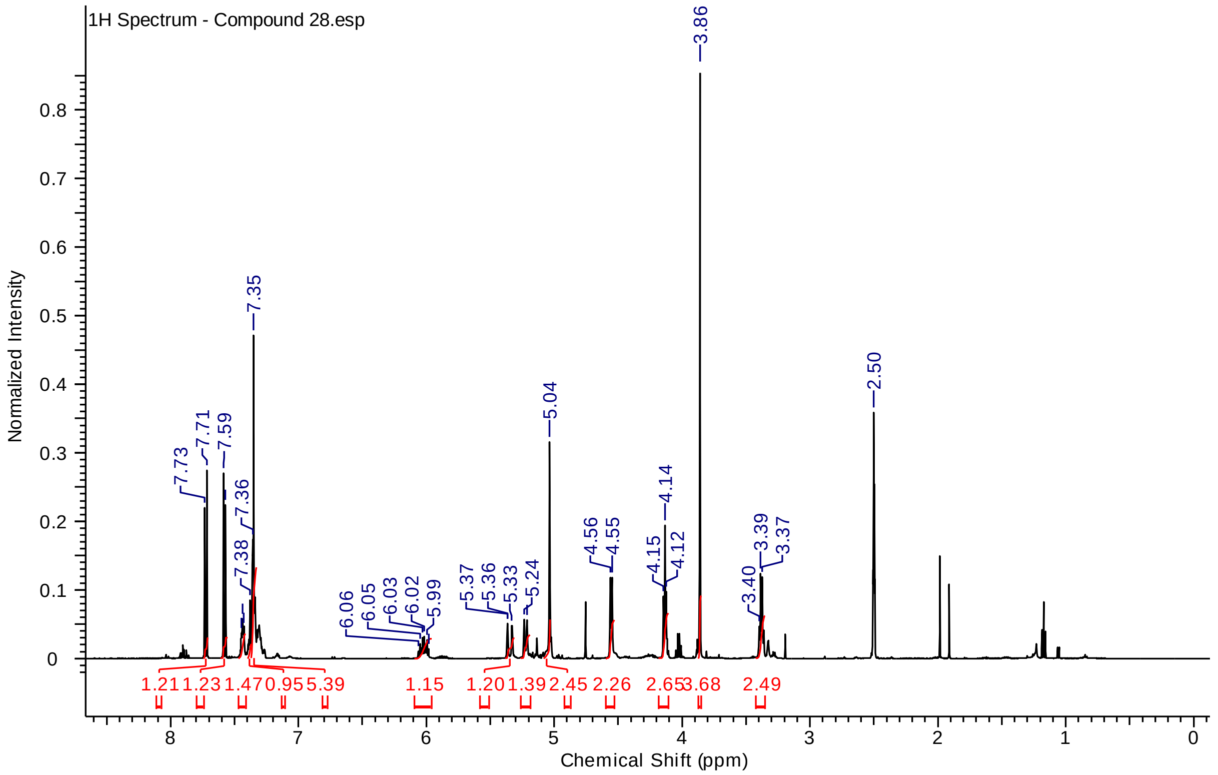

1H Spectrum - Compound 28.esp
Normalized Intensity
Chemical Shift (ppm)
7.73
7.71
7.59
7.36
7.38
7.35
6.06
6.05
6.03
6.02
5.99
5.37
5.36
5.33
5.24
5.04
4.56
4.55
4.15
4.14
4.12
3.86
3.40
3.39
3.37
2.50
1.21
1.23
1.47
0.95
5.39
1.15
1.20
1.39
2.45
2.26
2.65
3.68
2.49

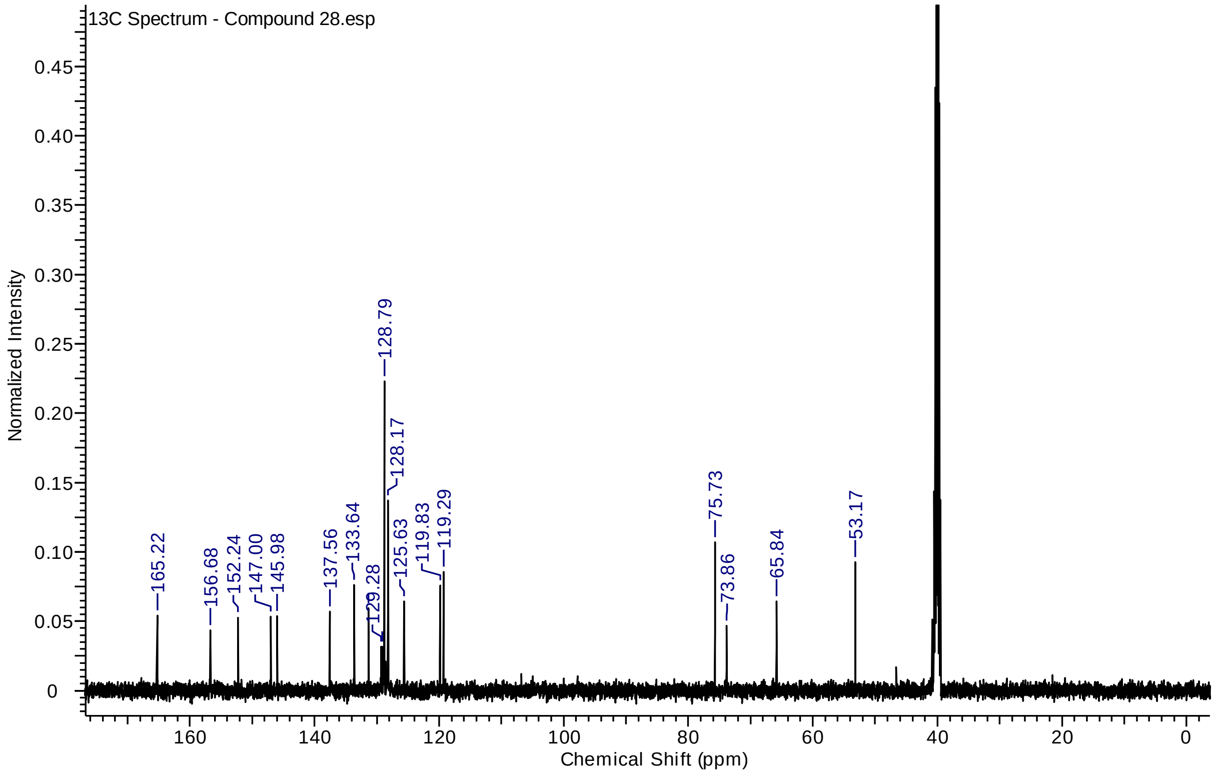

13C Spectrum - Compound 28.esp
Normalized Intensity
Chemical Shift (ppm)
165.22
156.68
152.24
147.00
145.98
137.56
133.64
129.28
128.79
128.17
125.63
119.83
119.29
75.73
73.86
65.84
53.17

Compound 29

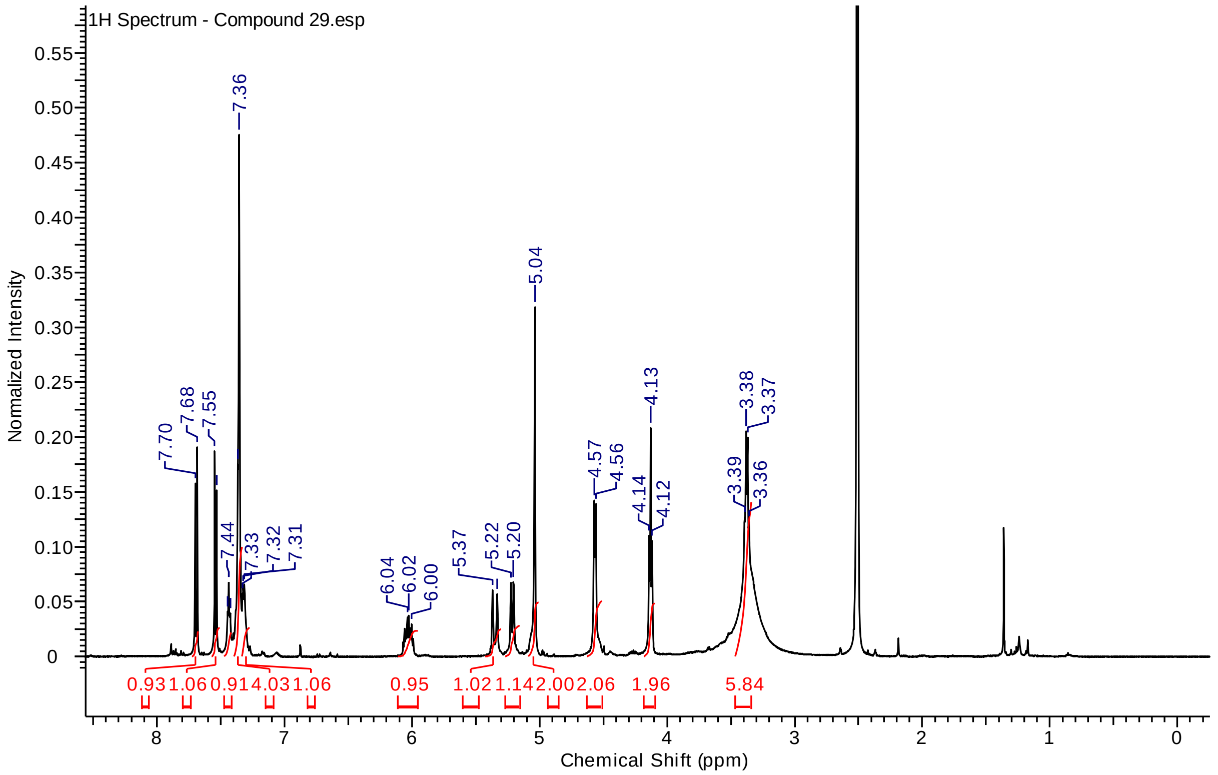
1H Spectrum - Compound 29.esp
Normalized Intensity
Chemical Shift (ppm)
7.70
7.68
7.55
7.36
7.44
7.33
7.32
7.31
6.04
6.02
6.00
5.37
5.22
5.20
5.04
4.57
4.56
4.14
4.12
4.13
3.39
3.38
3.37
3.36
0.93
1.06
0.91
4.03
1.06
0.95
1.02
1.14
2.00
2.06
1.96
5.84

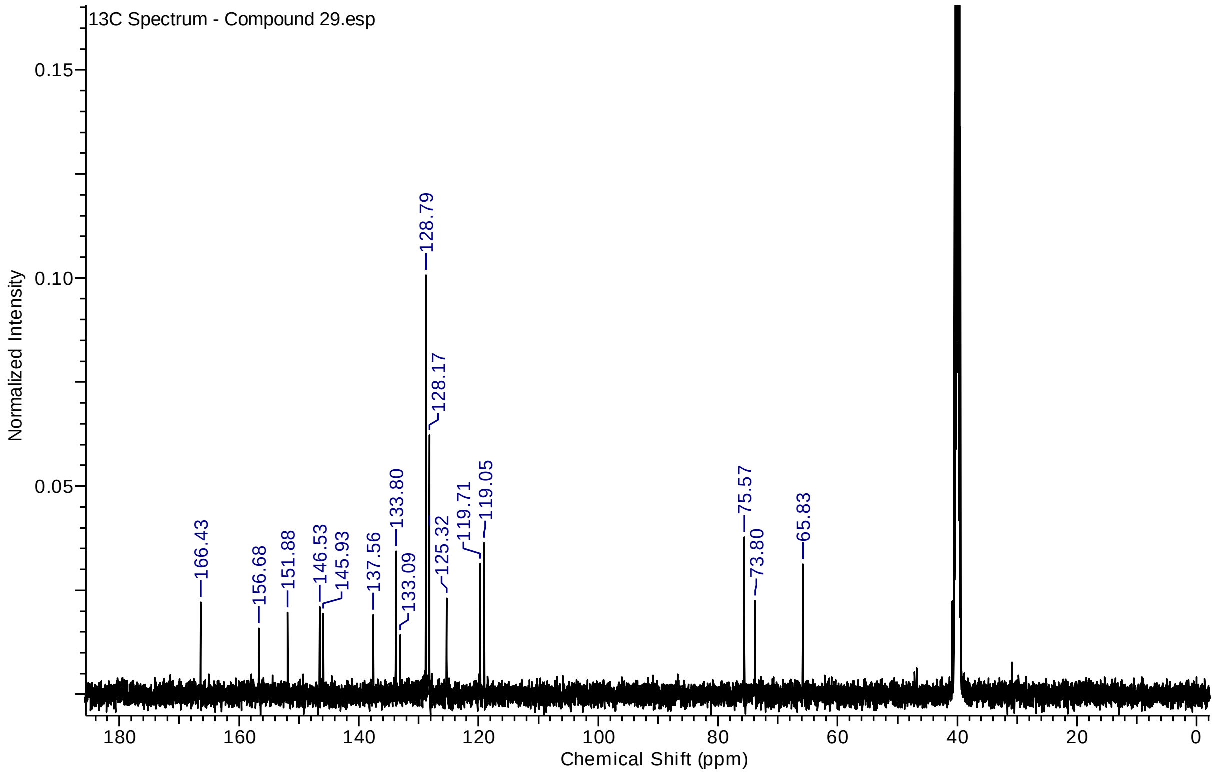
13C Spectrum - Compound 29.esp
Normalized Intensity
Chemical Shift (ppm)
166.43
156.68
151.88
146.53
145.93
137.56
133.80
133.09
128.79
128.17
125.32
119.71
119.05
75.57
73.80
65.83

Compound 30

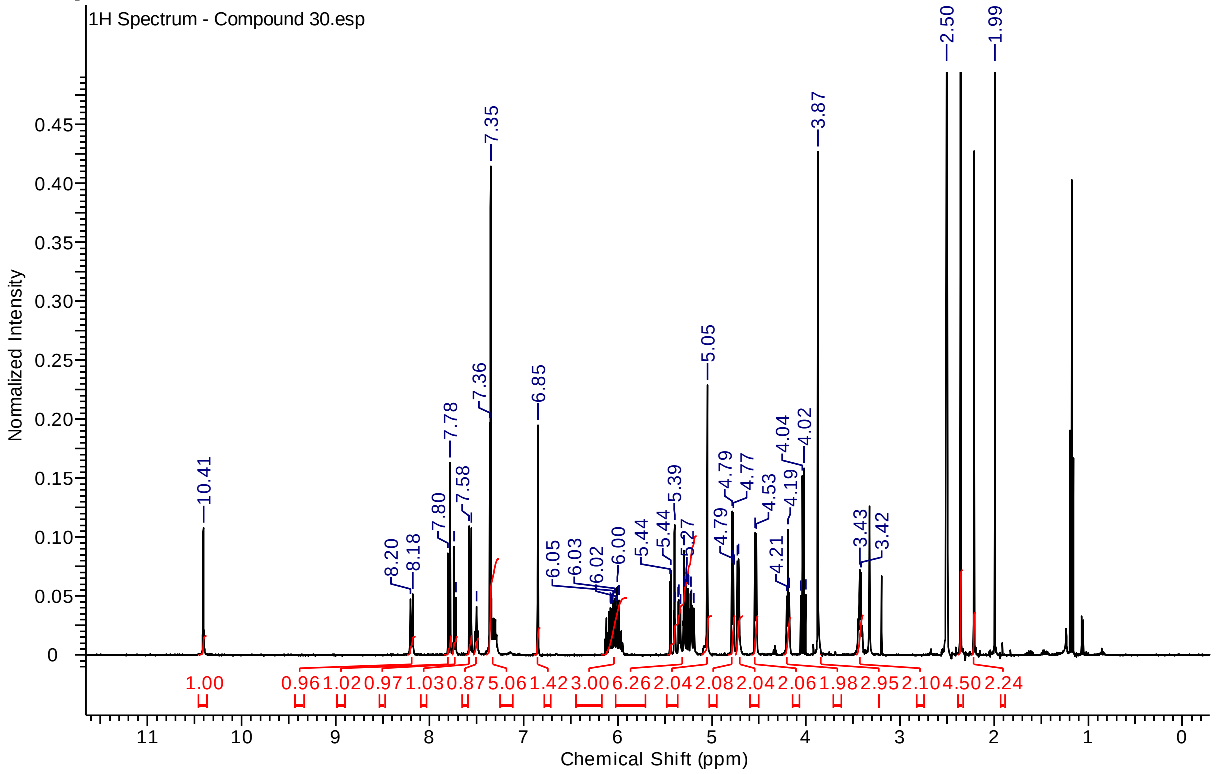
1H Spectrum - Compound 30.esp
Normalized Intensity
Chemical Shift (ppm)
10.41
8.20
8.18
7.80
7.78
7.58
7.36
7.35
6.85
6.05
6.03
6.02
6.00
5.44
5.44
5.39
5.27
5.05
4.79
4.79
4.77
4.53
4.21
4.19
4.04
4.02
3.87
3.43
3.42
2.50
1.99
1.00
0.96
1.02
0.97
1.03
0.87
5.06
1.42
3.00
6.26
2.04
2.08
2.04
2.06
1.98
2.95
2.10
4.50
2.24

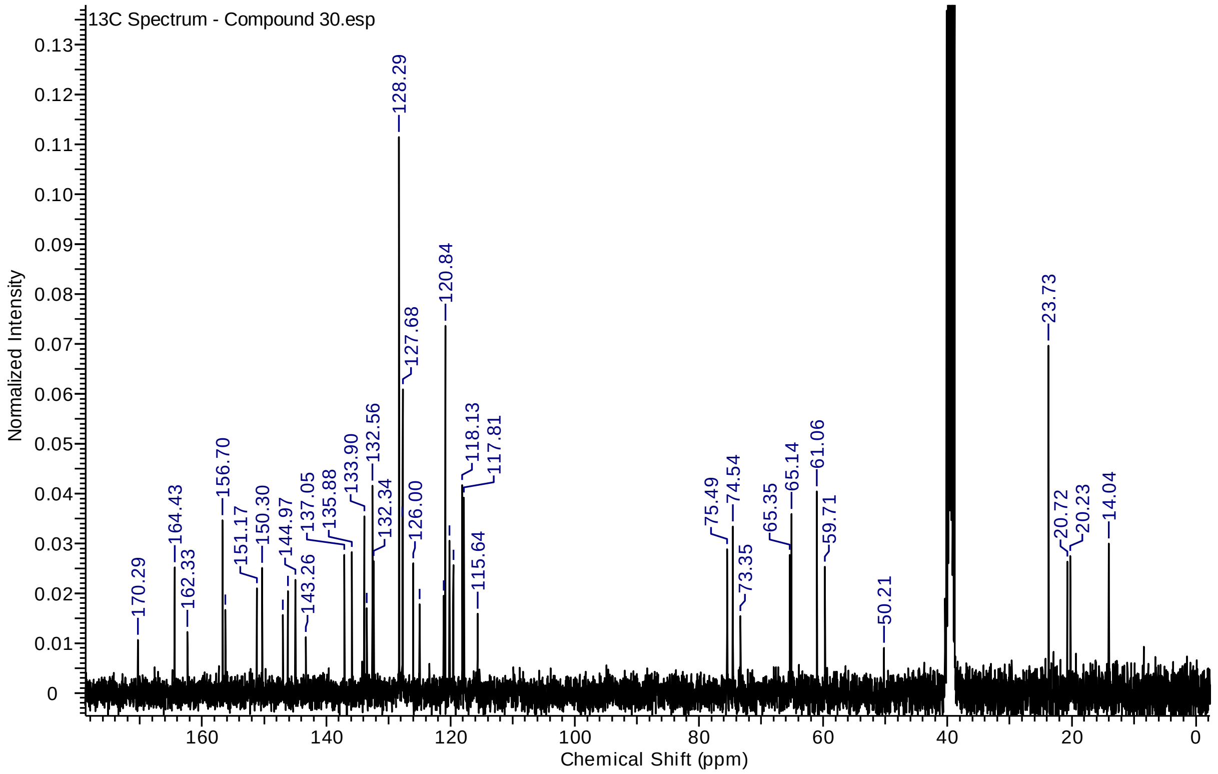
13C Spectrum - Compound 30.esp
Normalized Intensity
Chemical Shift (ppm)
170.29
164.43
162.33
156.70
151.17
150.30
144.97
143.26
137.05
135.88
133.90
132.56
132.34
128.29
127.68
126.00
120.84
118.13
117.81
115.64
75.49
74.54
73.35
65.35
65.14
61.06
59.71
50.21
23.73
20.72
20.23
14.04

Compound 31

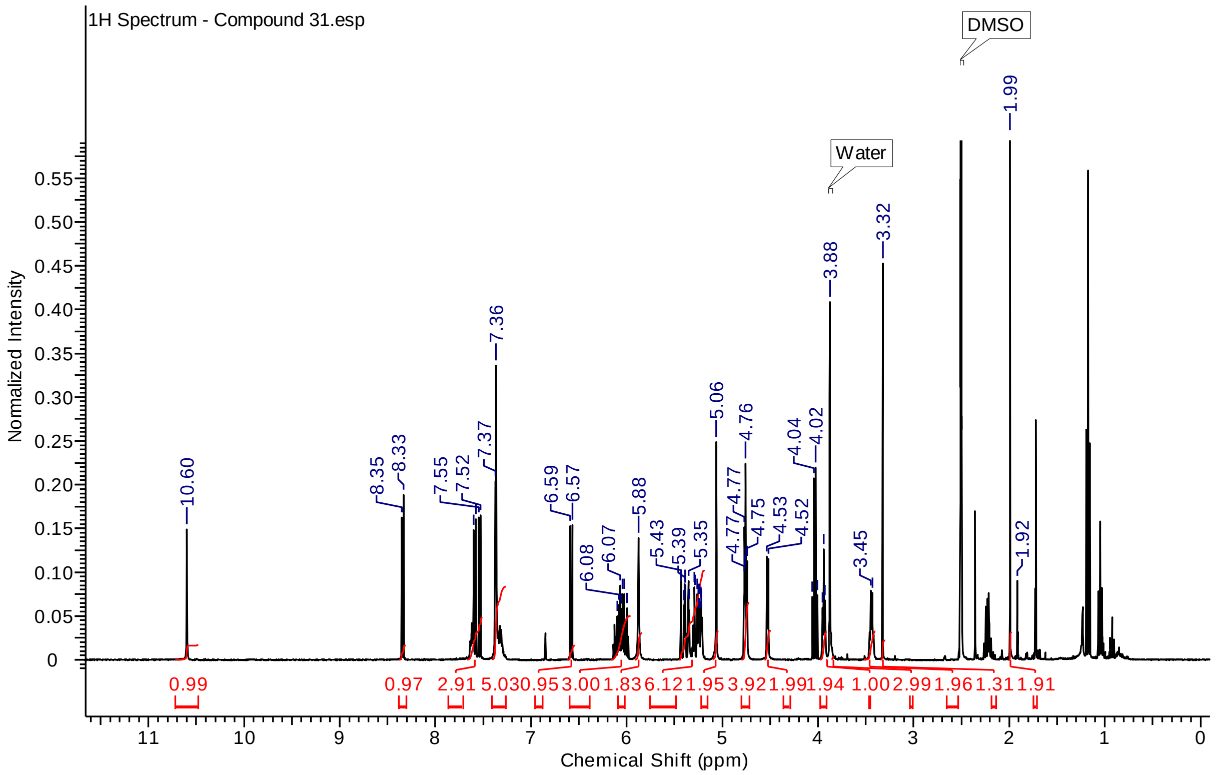
1H Spectrum - Compound 31.esp
DMSO
Water
Normalized Intensity
Chemical Shift (ppm)
10.60
8.35
8.33
7.55
7.52
7.37
7.36
6.59
6.57
6.08
6.07
5.88
5.43
5.39
5.35
5.06
4.77
4.77
4.76
4.75
4.53
4.52
4.04
4.02
3.88
3.45
3.32
1.99
1.92
0.99
0.97
2.91
5.03
0.95
3.00
1.83
6.12
1.95
3.92
1.99
1.94
1.00
2.99
1.96
1.31
1.91

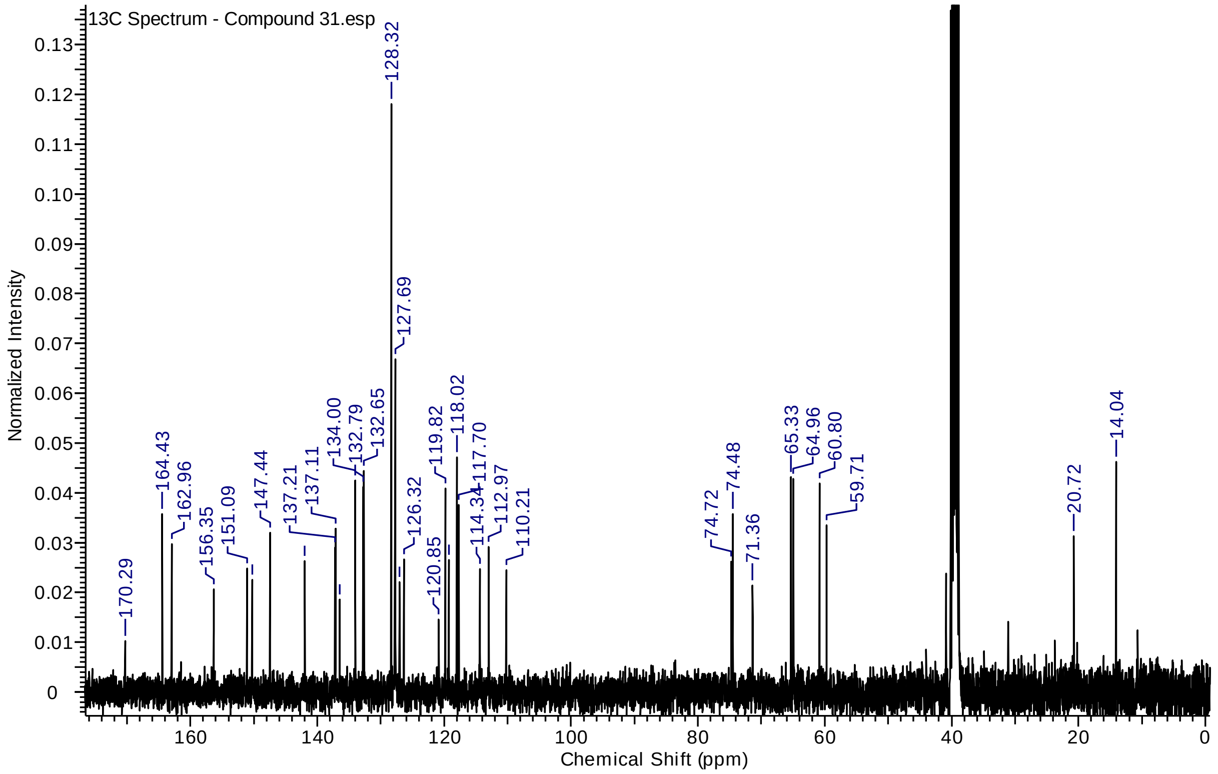
13C Spectrum - Compound 31.esp
Normalized Intensity
Chemical Shift (ppm)
170.29
164.43
162.96
156.35
151.09
147.44
137.21
137.11
134.00
132.79
132.65
128.32
127.69
126.32
120.85
119.82
118.02
117.70
114.34
112.97
110.21
74.72
74.48
71.36
65.33
64.96
60.80
59.71
20.72
14.04

Compound 32

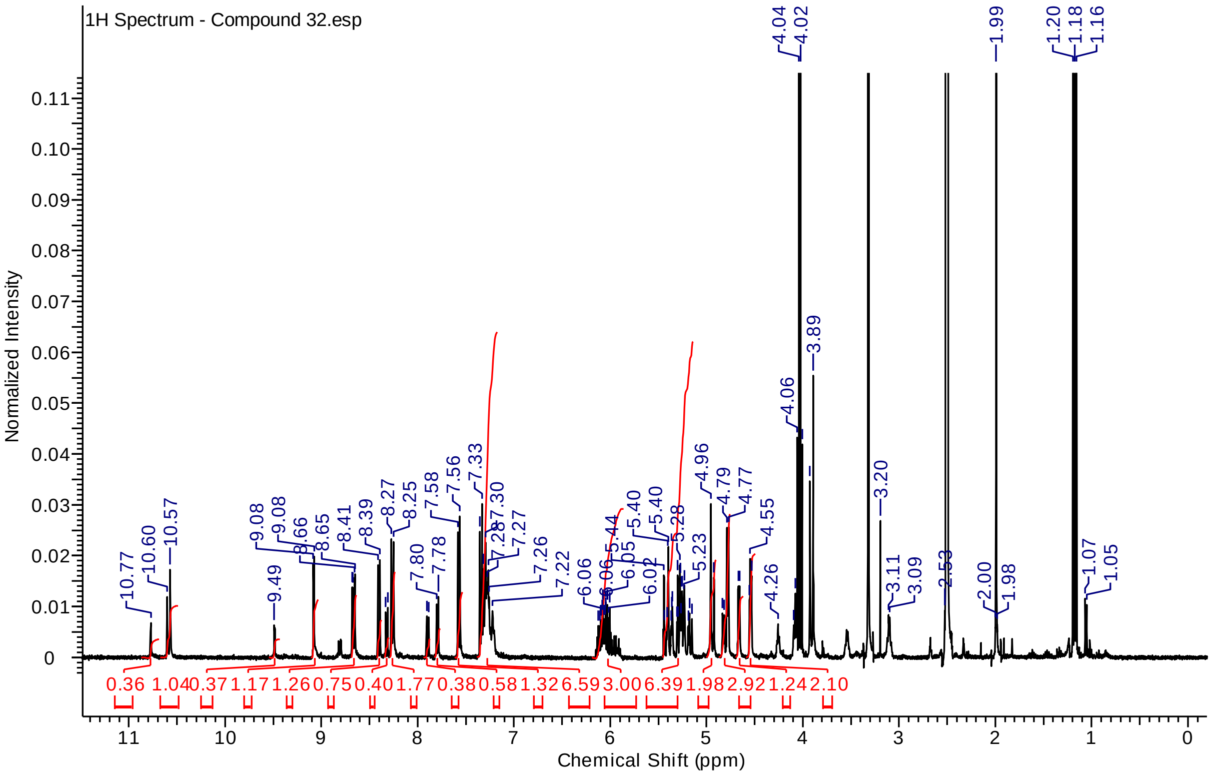
1H Spectrum - Compound 32.esp
Normalized Intensity
Chemical Shift (ppm)
10.77 10.60 10.57 9.49 9.08 9.08 8.66 8.65 8.41 8.39 8.27 8.25 7.80 7.78 7.58 7.56 7.33 7.30 7.28 7.27 7.26 7.22 6.06 6.06 6.05 6.02 5.44 5.40 5.40 5.28 5.23 4.96 4.79 4.77 4.55 4.26 4.06 4.04 4.02 3.89 3.20 3.11 3.09 2.53 2.00 1.99 1.98 1.20 1.18 1.16 1.07 1.05
0.36 1.04 0.37 1.17 1.26 0.75 0.40 1.77 0.38 0.58 1.32 6.59 3.00 6.39 1.98 2.92 1.24 2.10

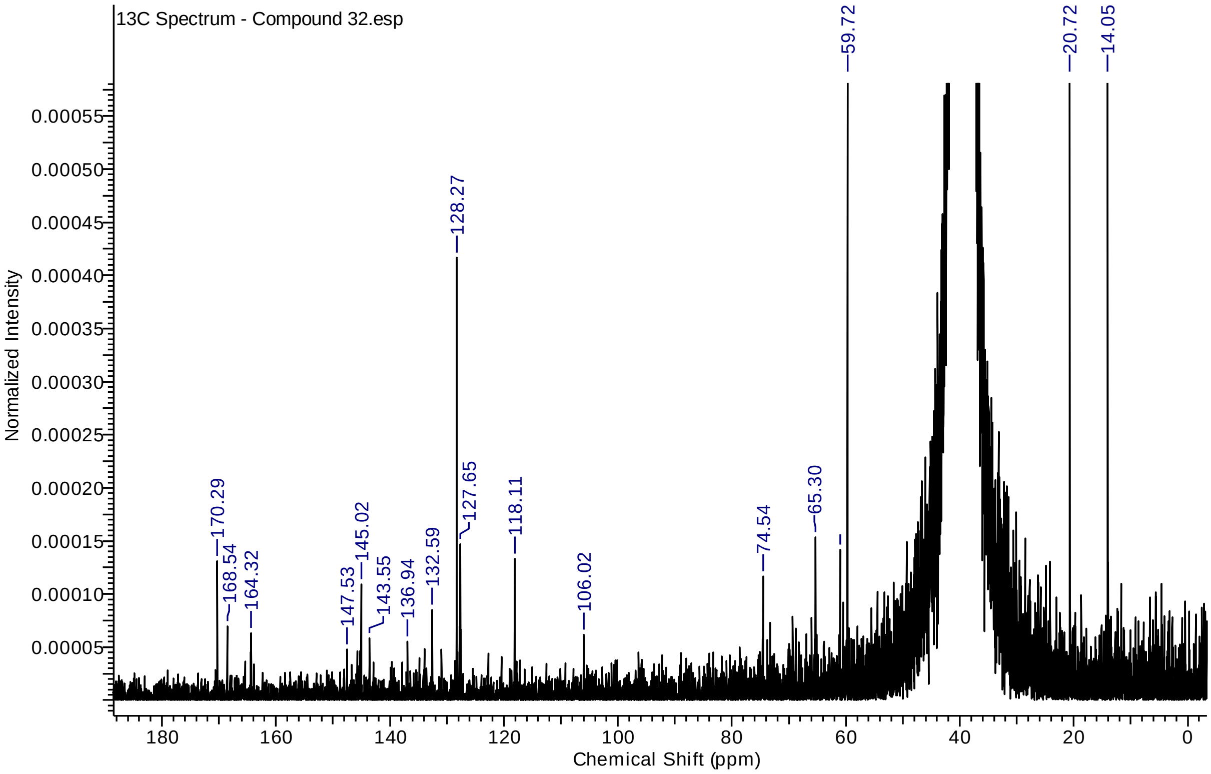
13C Spectrum - Compound 32.esp
Normalized Intensity
Chemical Shift (ppm)
170.29 168.54 164.32 147.53 145.02 143.55 136.94 132.59 128.27 127.65 118.11 106.02 74.54 65.30 59.72 20.72 14.05

Compound 33

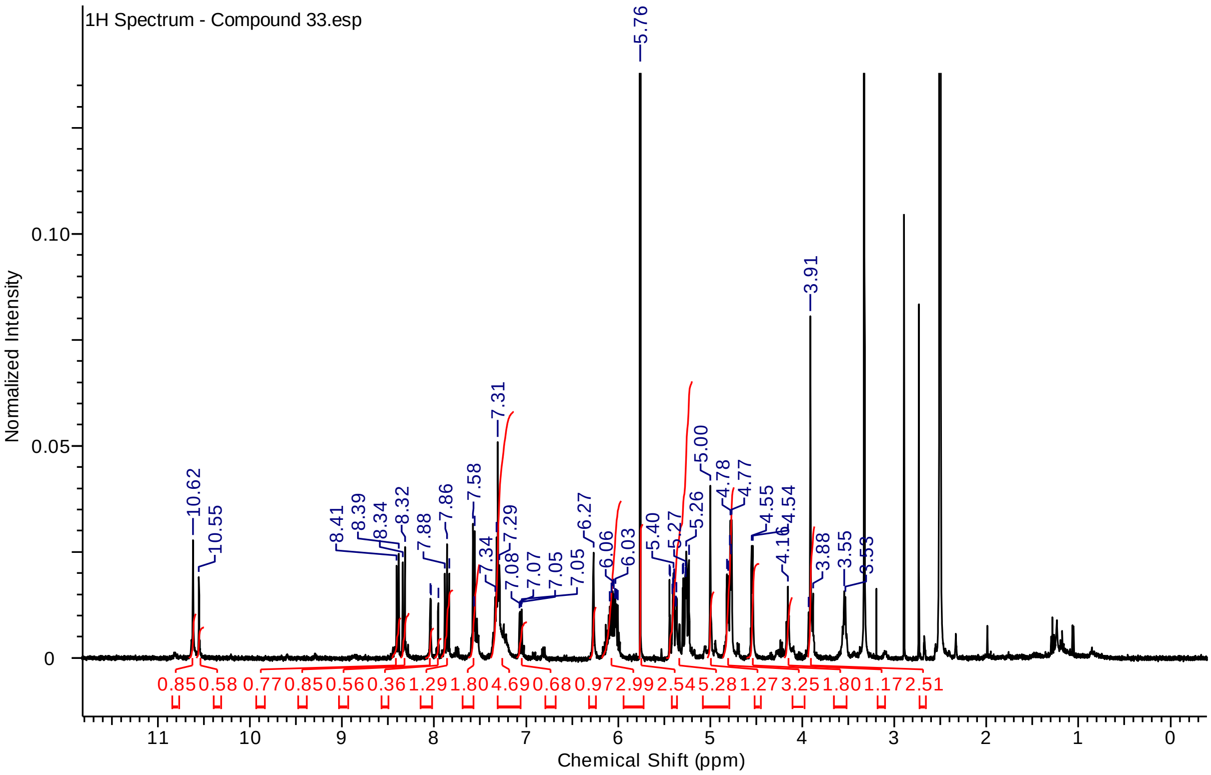

Compound 34

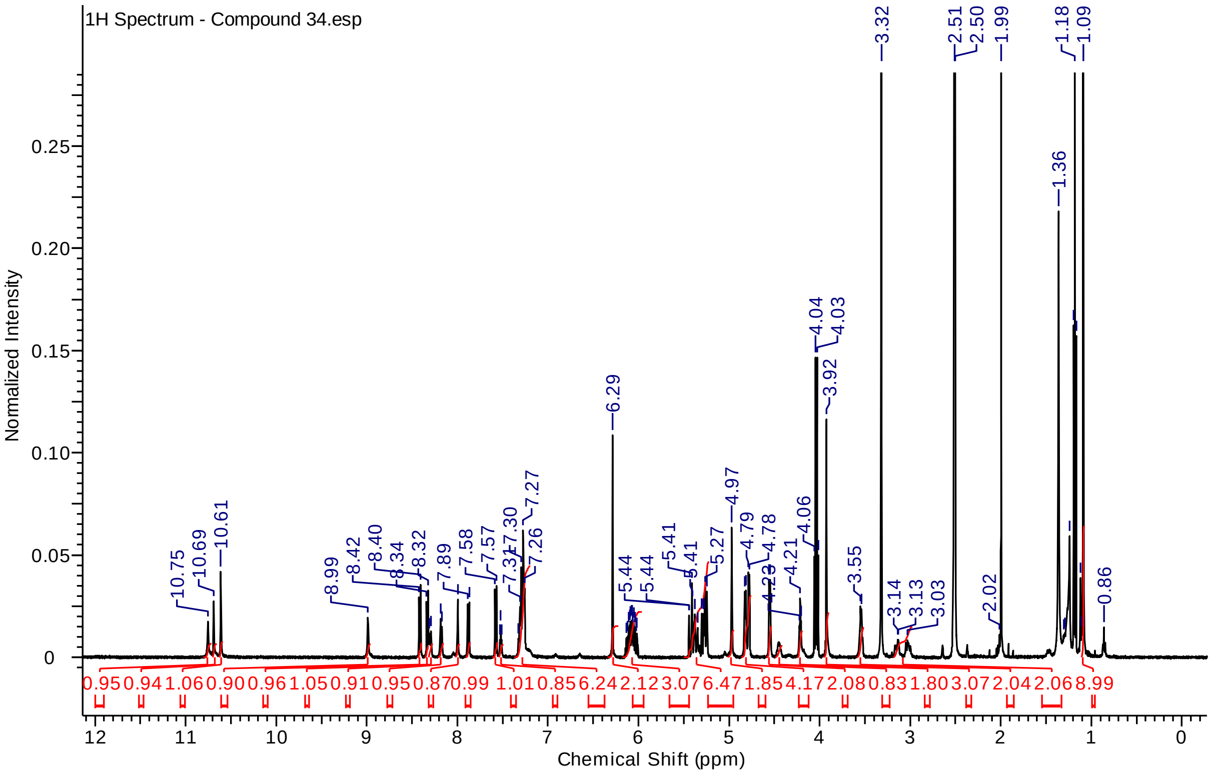

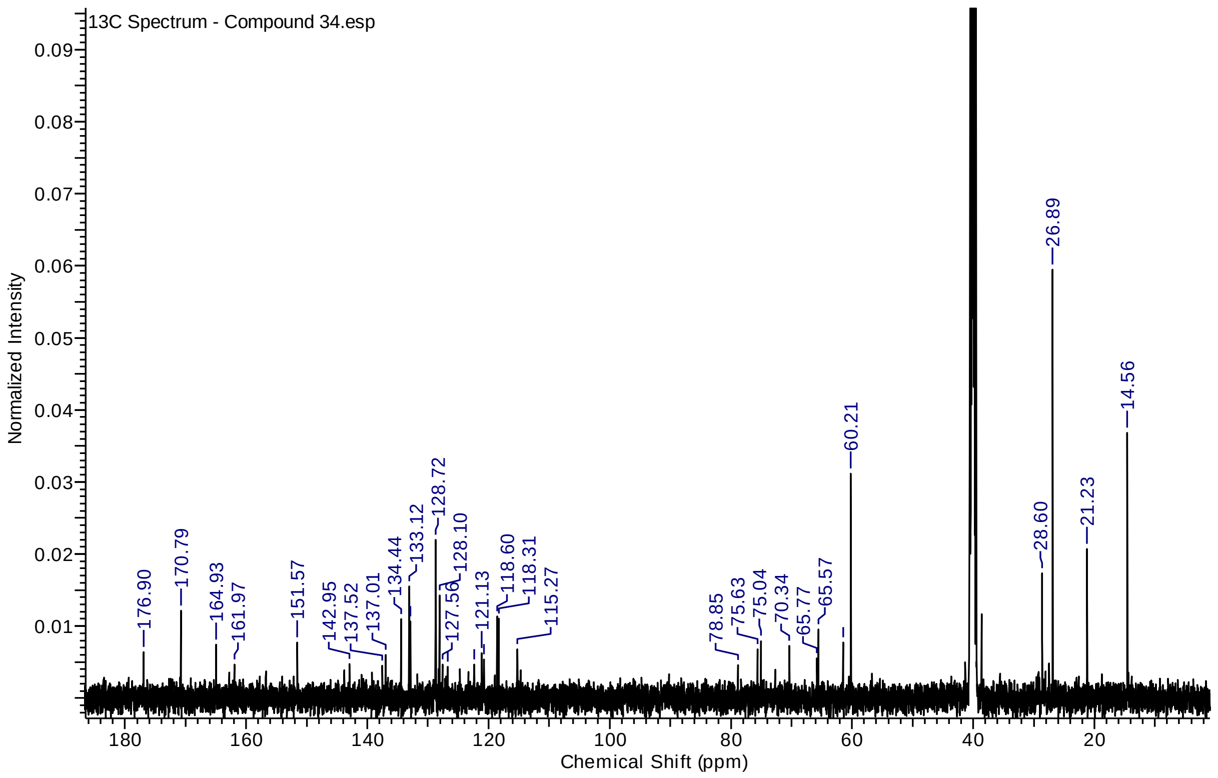
13C Spectrum - Compound 34.esp
Normalized Intensity
Chemical Shift (ppm)
176.90
170.79
164.93
161.97
151.57
142.95
137.52
137.01
134.44
133.12
128.72
128.10
127.56
121.13
118.60
118.31
115.27
78.85
75.63
75.04
70.34
65.77
65.57
60.21
28.60
26.89
21.23
14.56

Compound 35

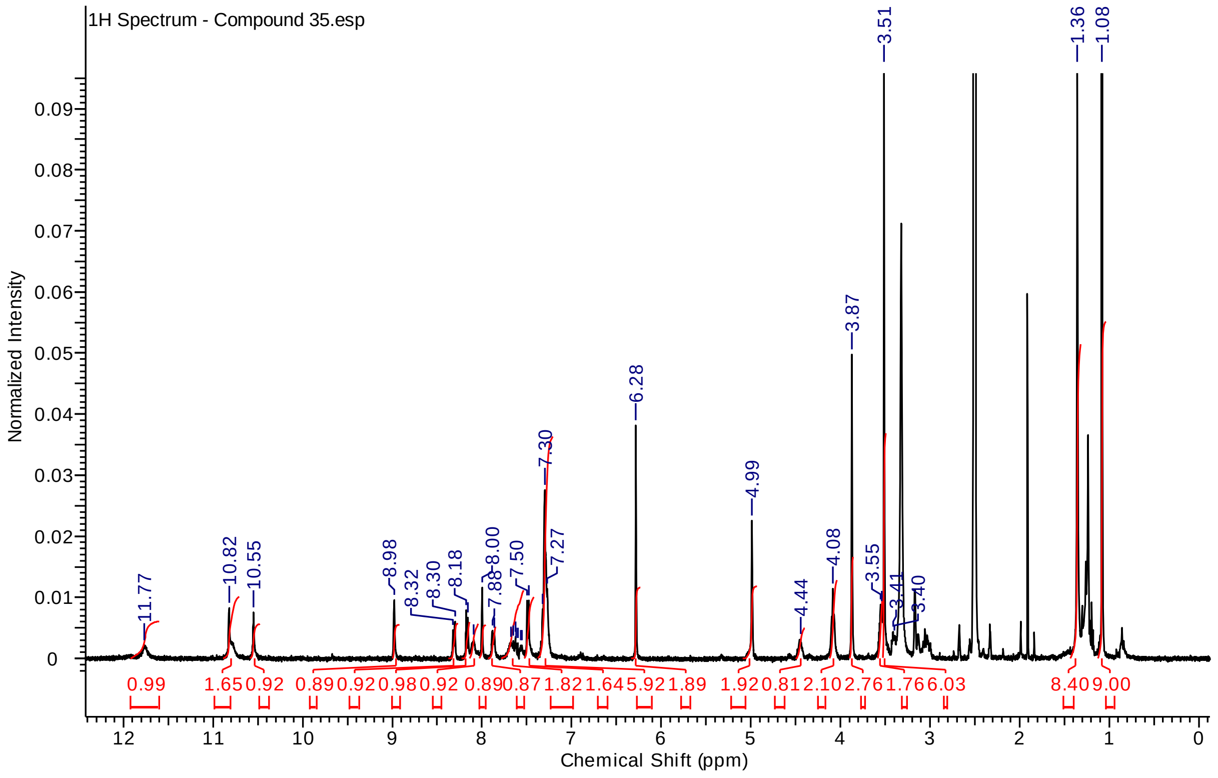
1H Spectrum - Compound 35.esp
Normalized Intensity
Chemical Shift (ppm)
11.77
10.82
10.55
8.98
8.32
8.30
8.18
8.00
7.88
7.50
7.30
7.27
6.28
4.99
4.44
4.08
3.87
3.55
3.51
3.41
3.40
1.36
1.08
0.99
1.65
0.92
0.89
0.92
0.98
0.92
0.89
0.87
1.82
1.64
5.92
1.89
1.92
0.81
2.10
2.76
1.76
6.03
8.40
9.00

Albi-2

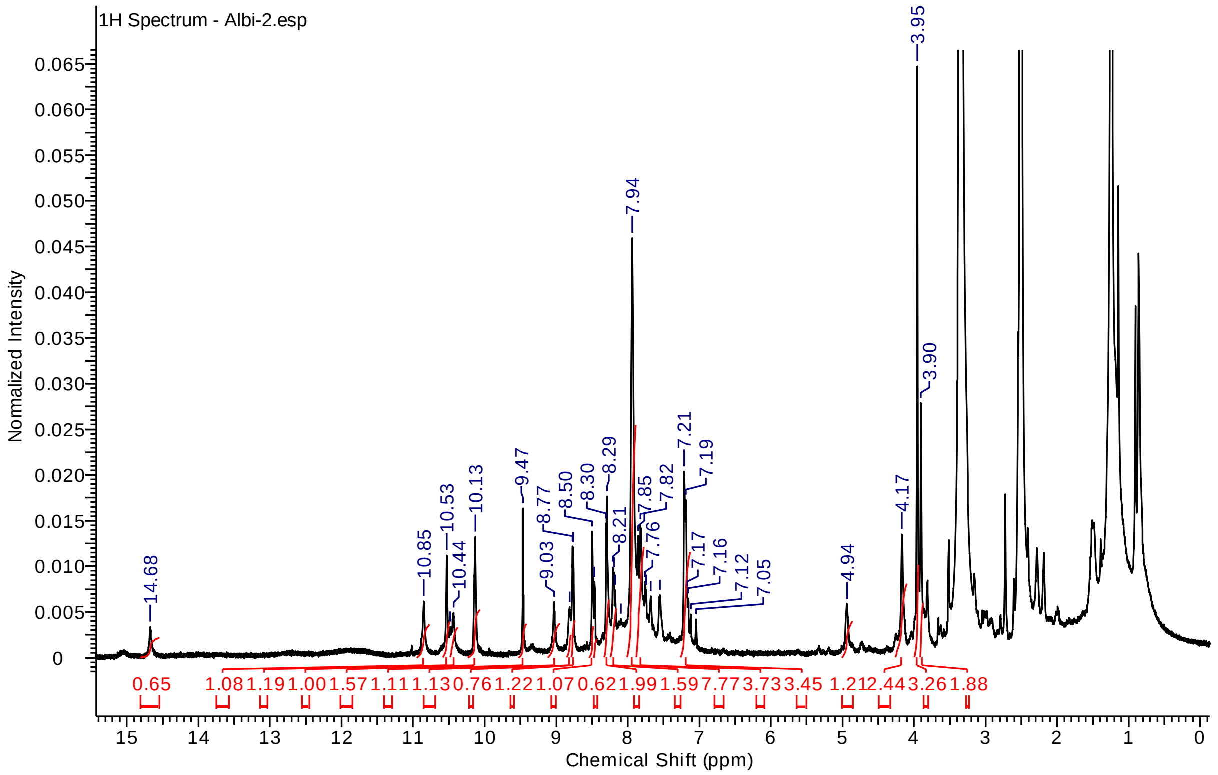

(^{1}H, ^{13}C)-HMBC - Albi-2

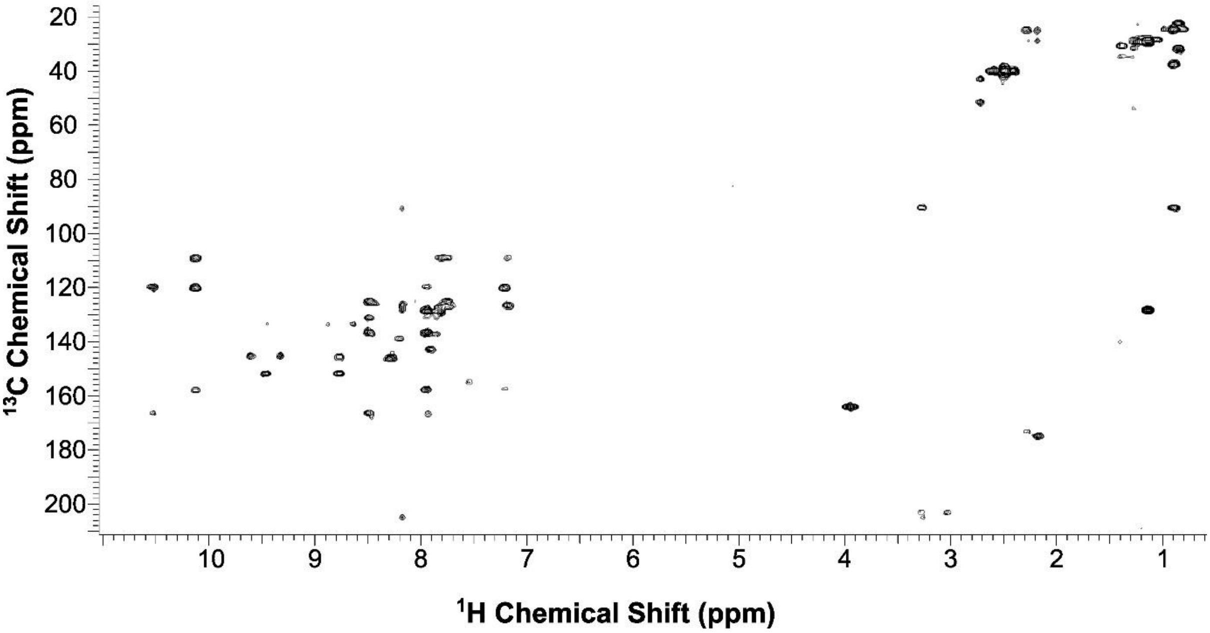

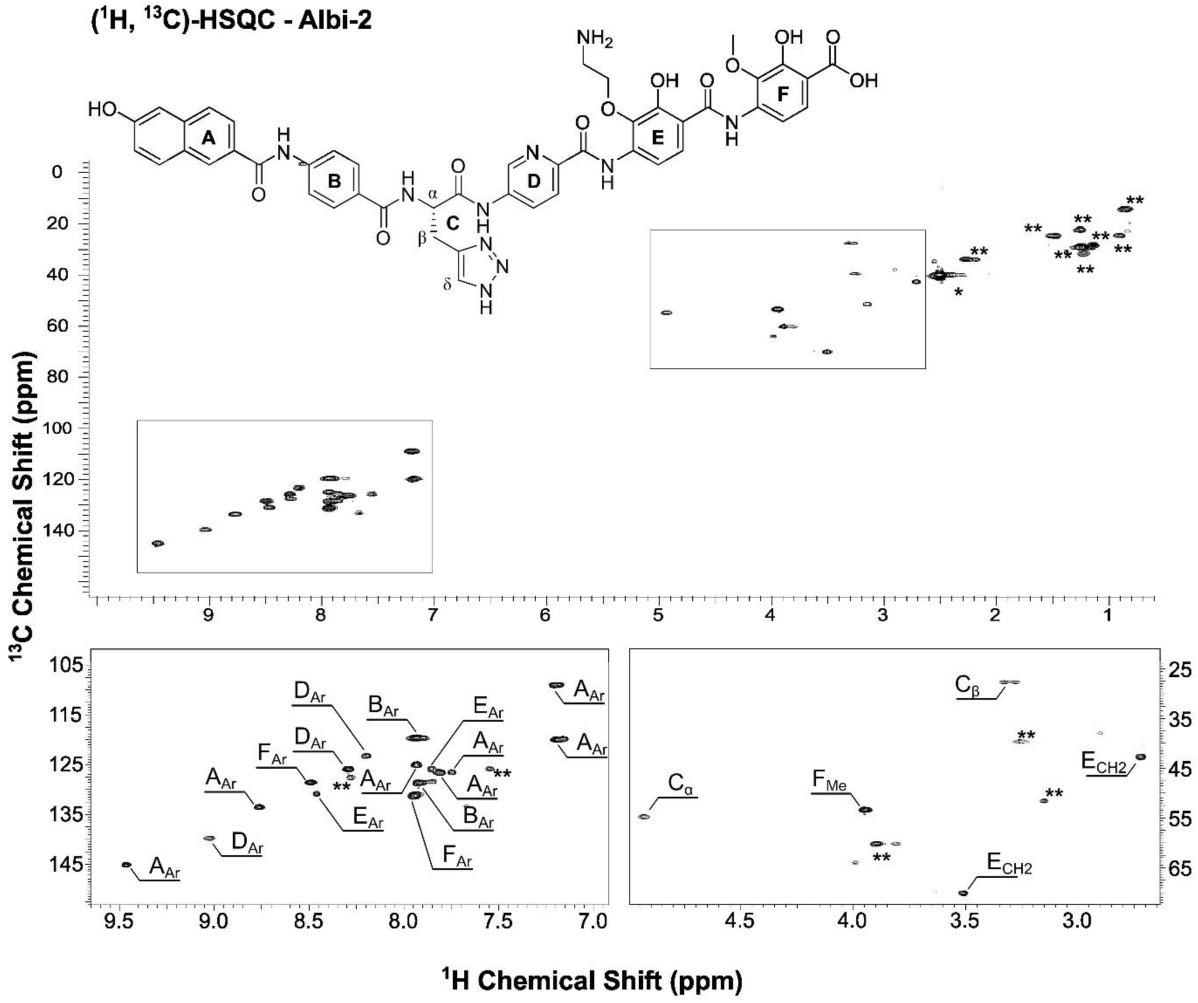

^{1}H-^{13}C-HSQC with magnification of relevant sections and annotations of signals (Ar: Aromatic, Me: Methyl, *: DMSO, ** impurity).

Compound 39

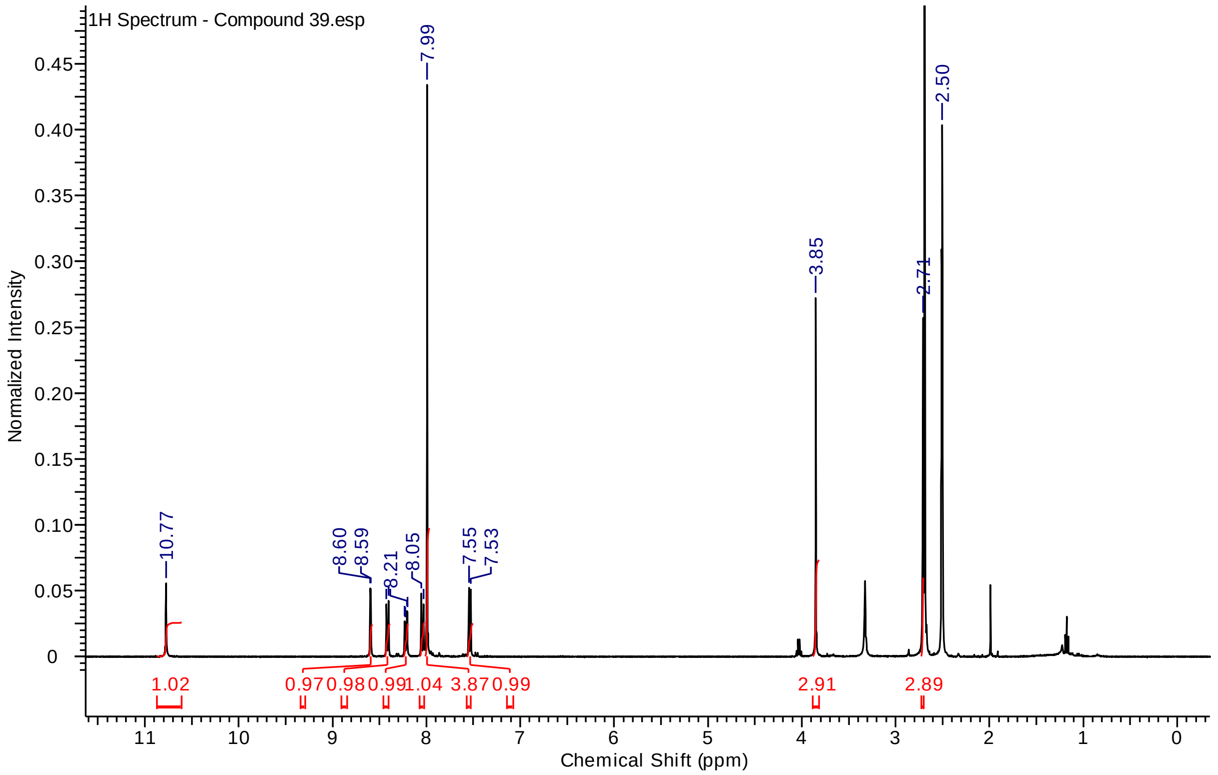
1H Spectrum - Compound 39.esp
Normalized Intensity
Chemical Shift (ppm)
10.77
8.60
8.59
8.21
8.05
7.99
7.55
7.53
3.85
2.71
2.50
1.02
0.97
0.98
0.99
1.04
3.87
0.99
2.91
2.89

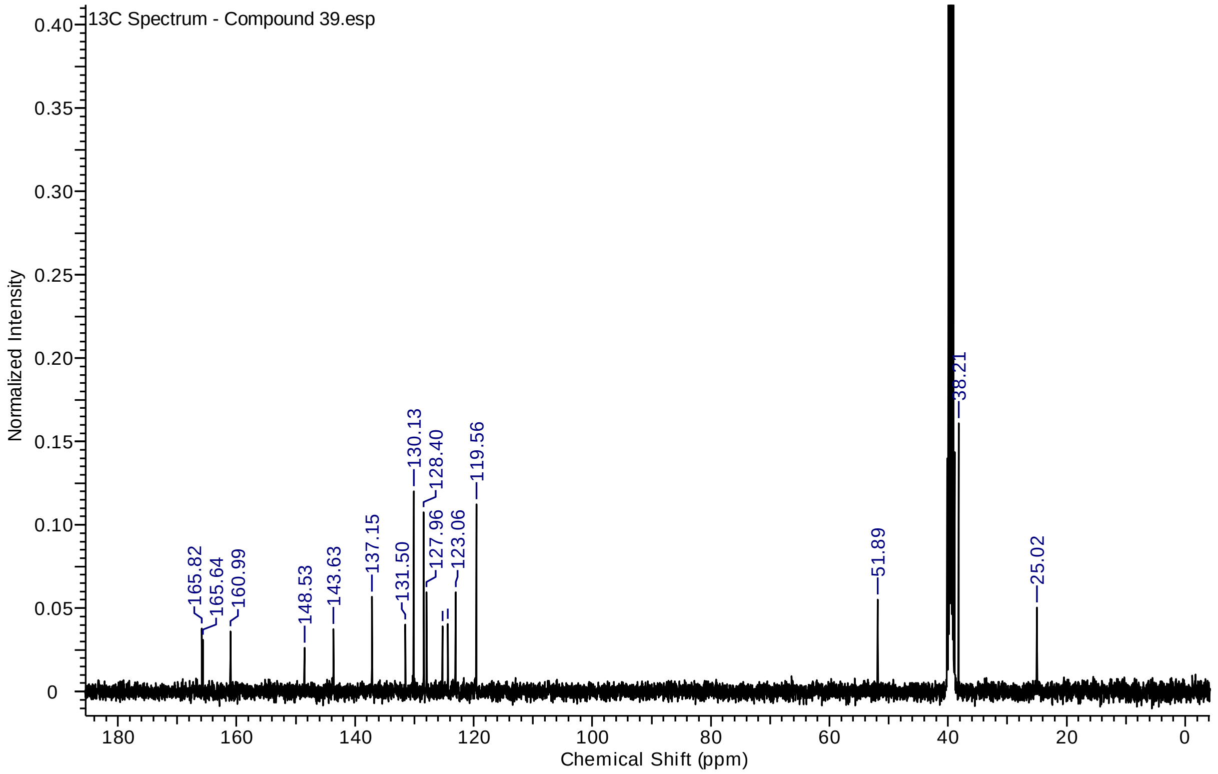
13C Spectrum - Compound 39.esp
Normalized Intensity
Chemical Shift (ppm)
165.82
165.64
160.99
148.53
143.63
137.15
131.50
130.13
128.40
127.96
123.06
119.56
51.89
38.21
25.02

Albi-3

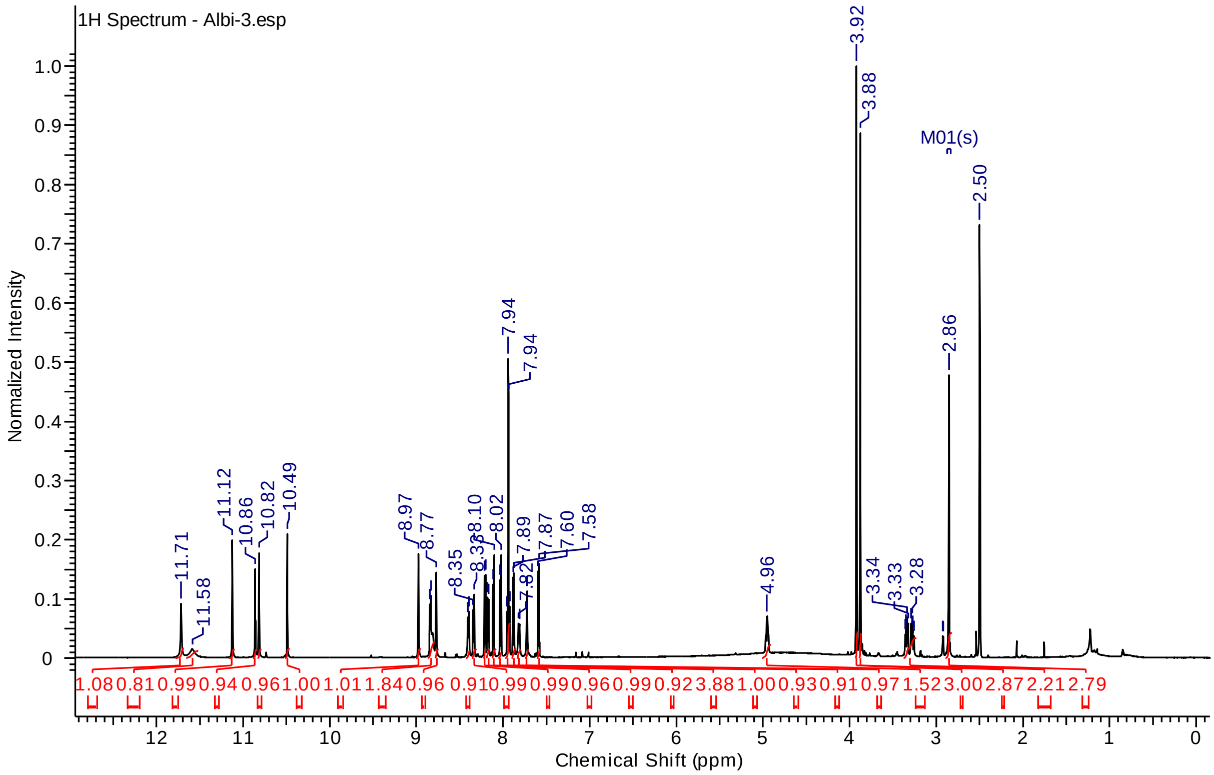

(^{1}H, ^{13}C)-HMBC - Albi-3

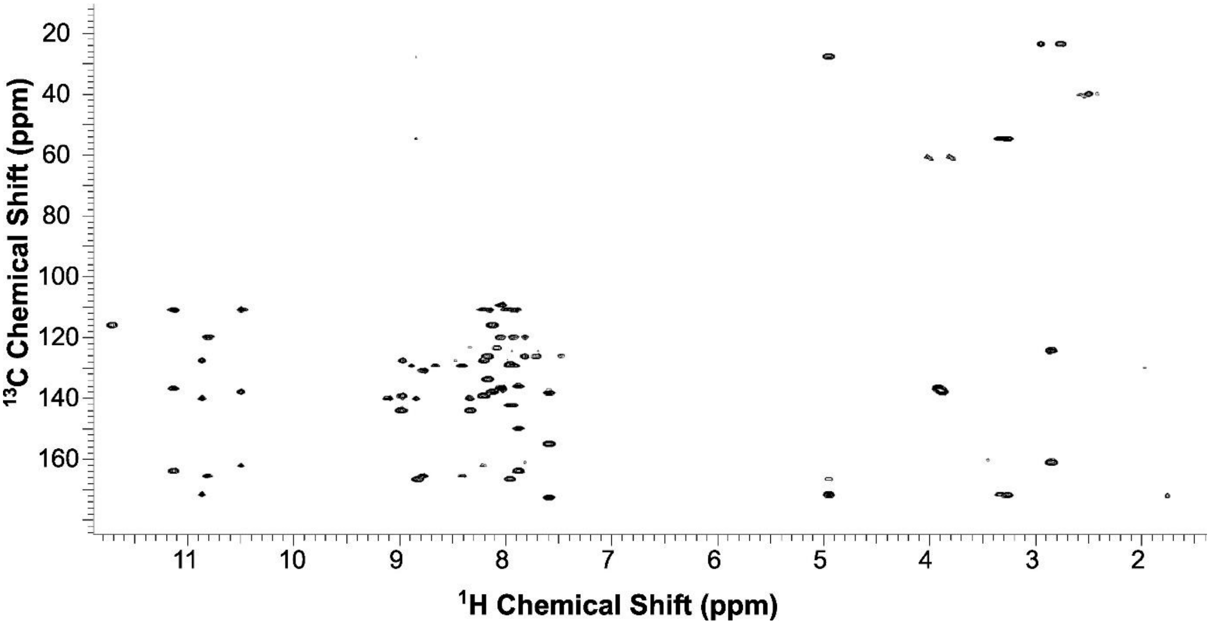

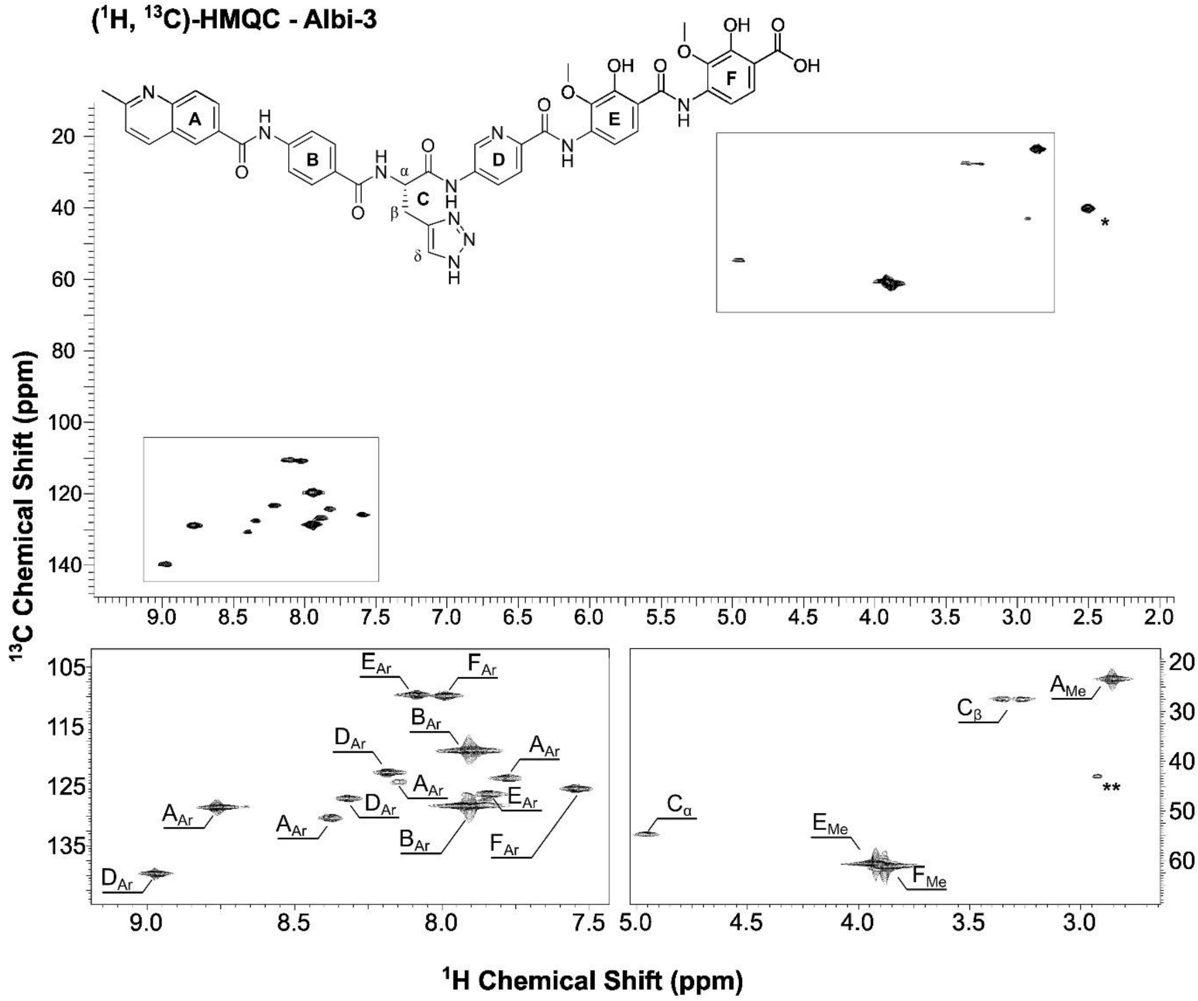

^{1}H-^{13}C-HMQC with magnification of relevant sections and annotations of signals (Ar: Aromatic, Me: Methyl, *: DMSO, ** impurity).

alkyne-Albi

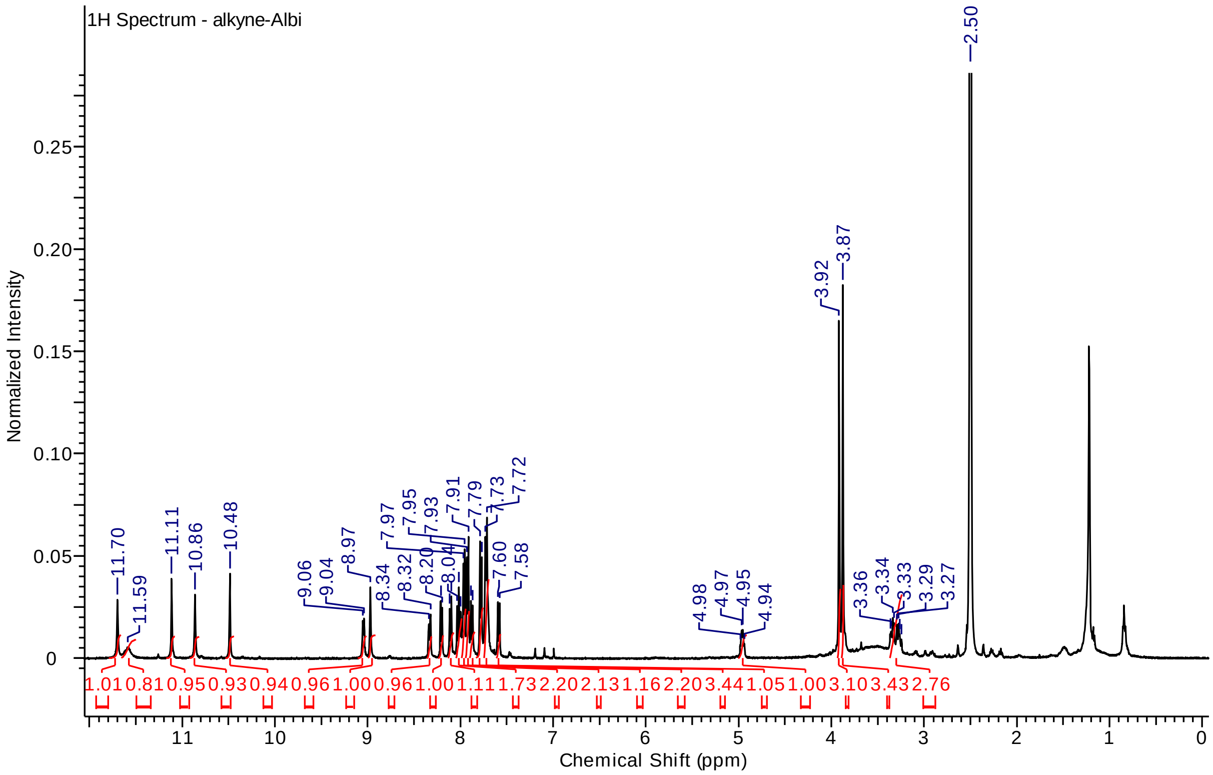

(^{1}H, ^{13}C)-HMBC - alkyne-Albi

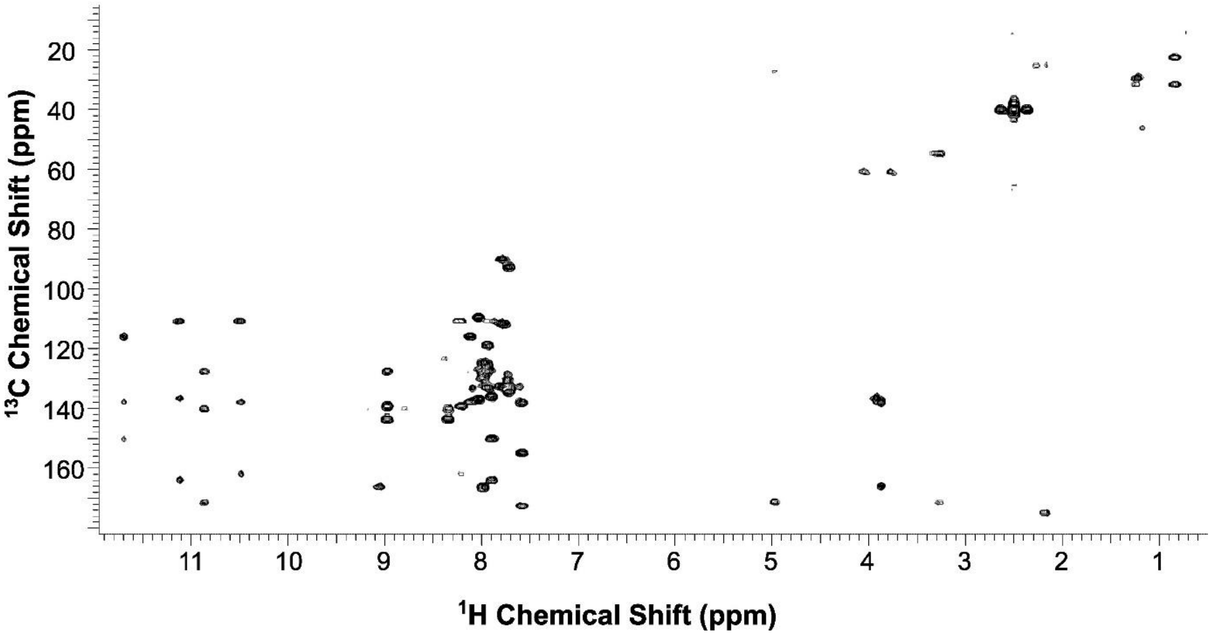

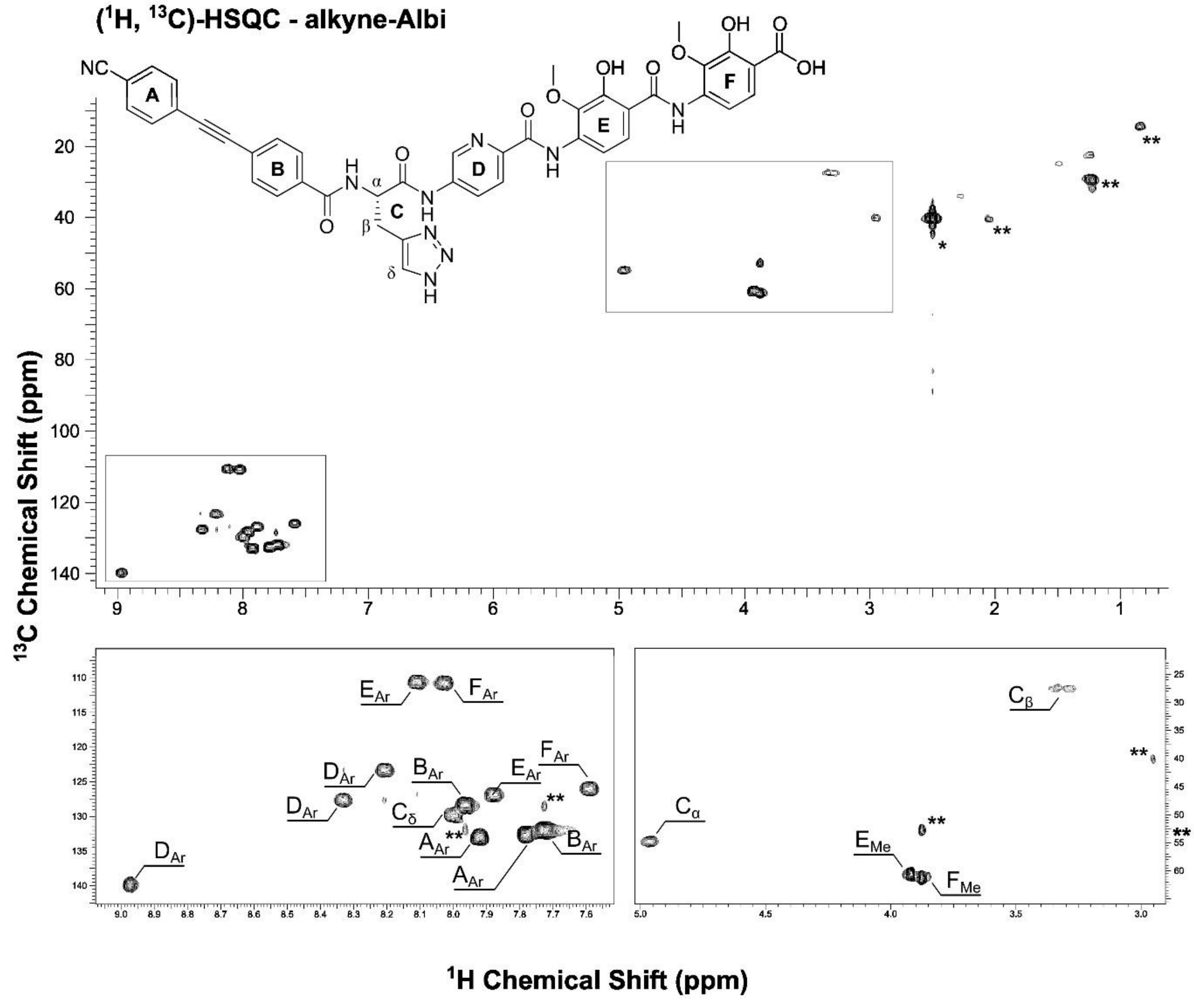

^{1}H-^{13}C-HSQC with magnification of relevant sections and annotations of signals (Ar: Aromatic, Me: Methyl, *: DMSO, ** impurity).

Compound 44

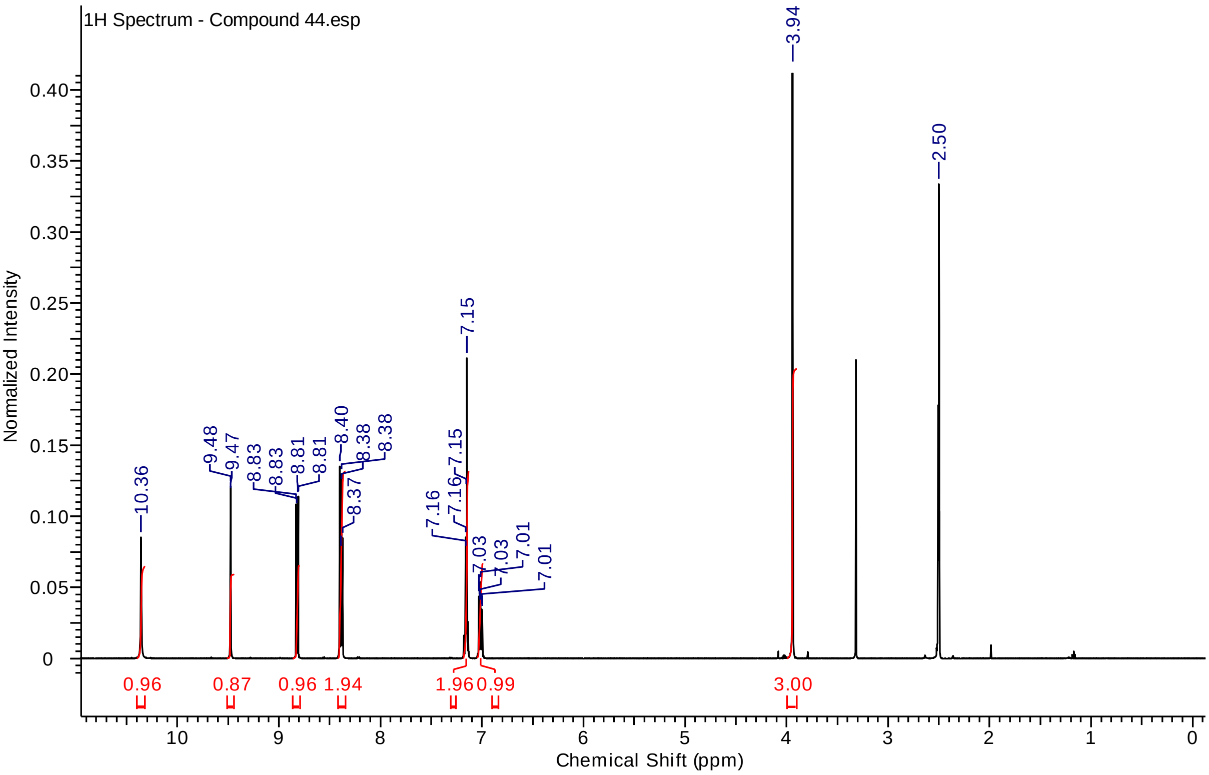

1H Spectrum - Compound 44.esp
Normalized Intensity
Chemical Shift (ppm)
10.36
9.48
9.47
8.83
8.83
8.81
8.81
8.40
8.38
8.38
8.37
7.16
7.16
7.15
7.15
7.03
7.03
7.01
7.01
3.94
2.50
0.96
0.87
0.96
1.94
1.96
0.99
3.00

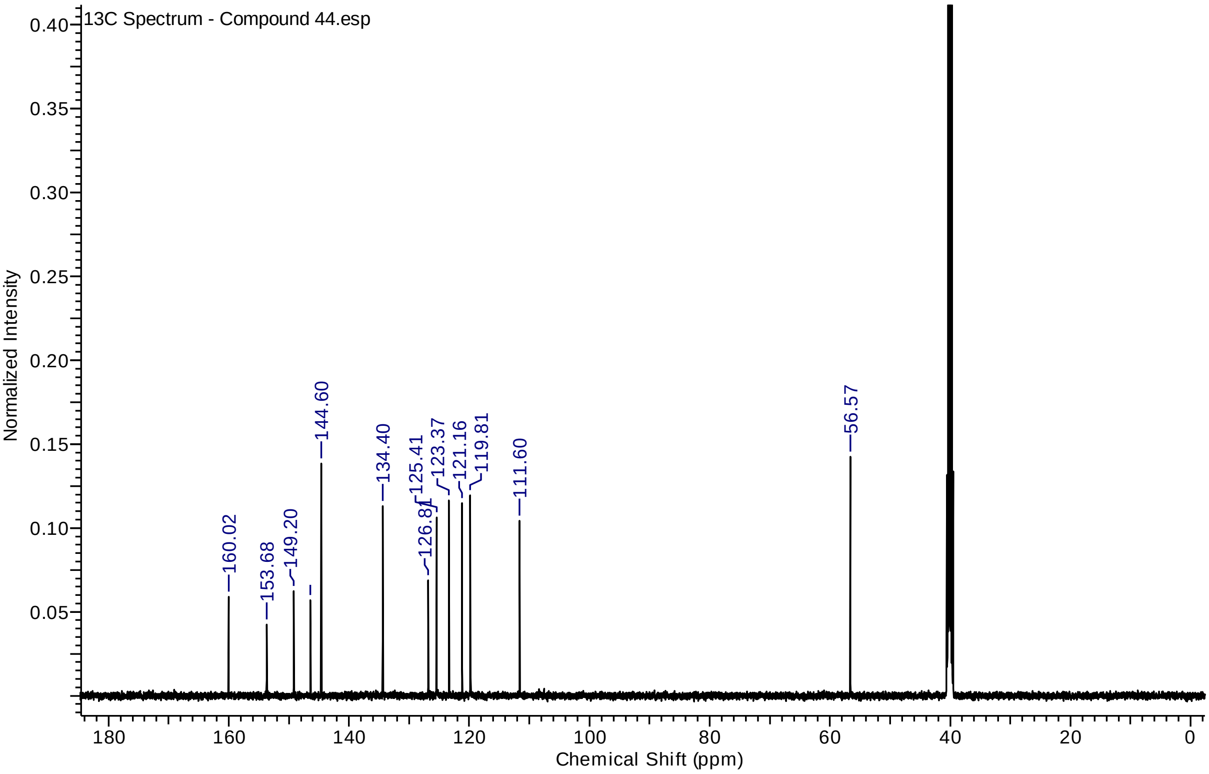

13C Spectrum - Compound 44.esp
Normalized Intensity
Chemical Shift (ppm)
160.02
153.68
149.20
144.60
134.40
126.81
125.41
123.37
121.16
119.81
111.60
56.57

Compound 45

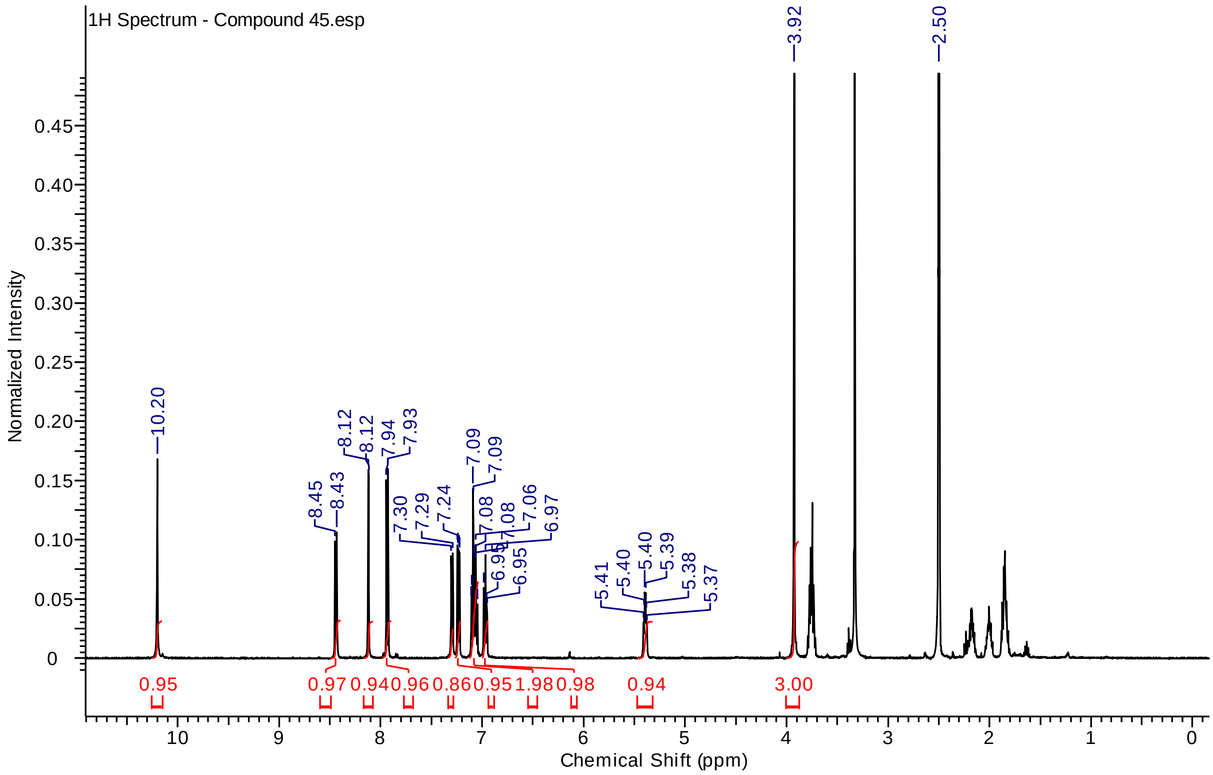
1H Spectrum - Compound 45.esp
Normalized Intensity
Chemical Shift (ppm)
10.20
8.45
8.43
8.12
8.12
7.94
7.93
7.30
7.29
7.24
7.09
7.09
7.08
7.08
7.06
6.97
6.95
6.95
5.41
5.40
5.40
5.39
5.38
5.37
3.92
2.50
0.95
0.97
0.94
0.96
0.86
0.95
1.98
0.98
0.94
3.00

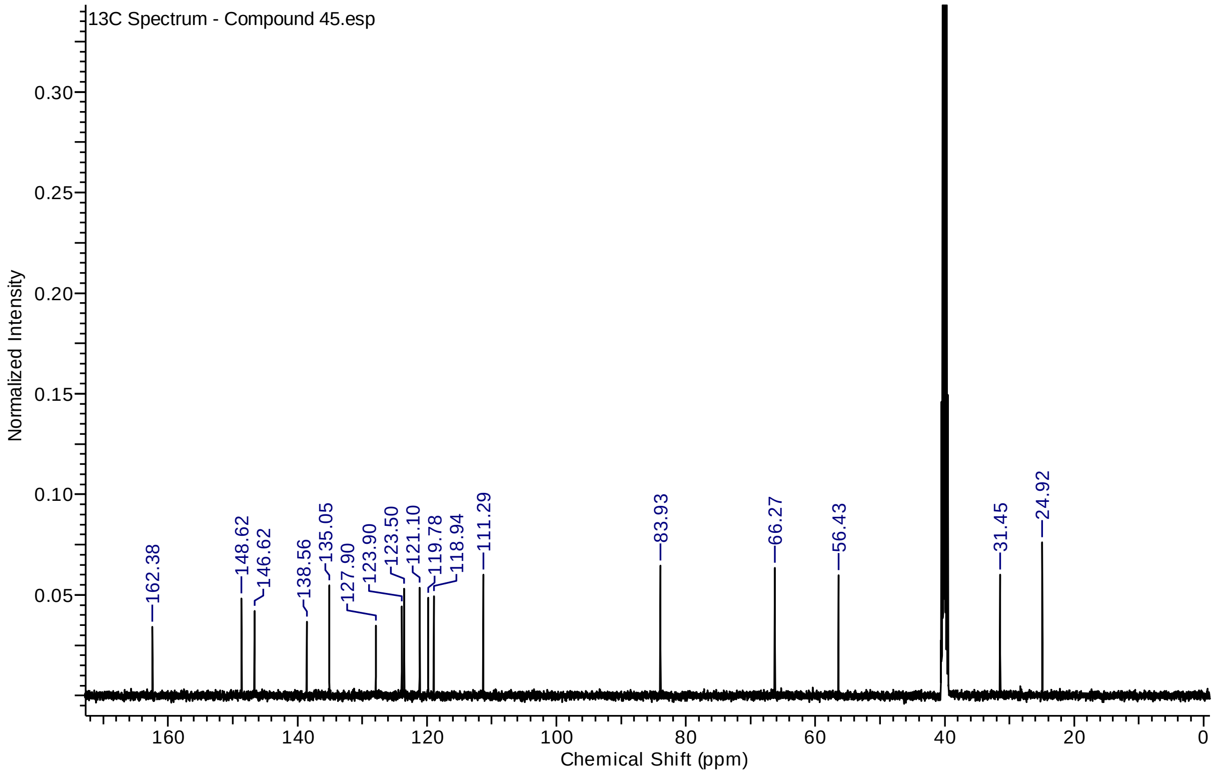
13C Spectrum - Compound 45.esp
Normalized Intensity
Chemical Shift (ppm)
162.38
148.62
146.62
138.56
135.05
127.90
123.90
123.50
121.10
119.78
118.94
111.29
83.93
66.27
56.43
31.45
24.92

Compound 46

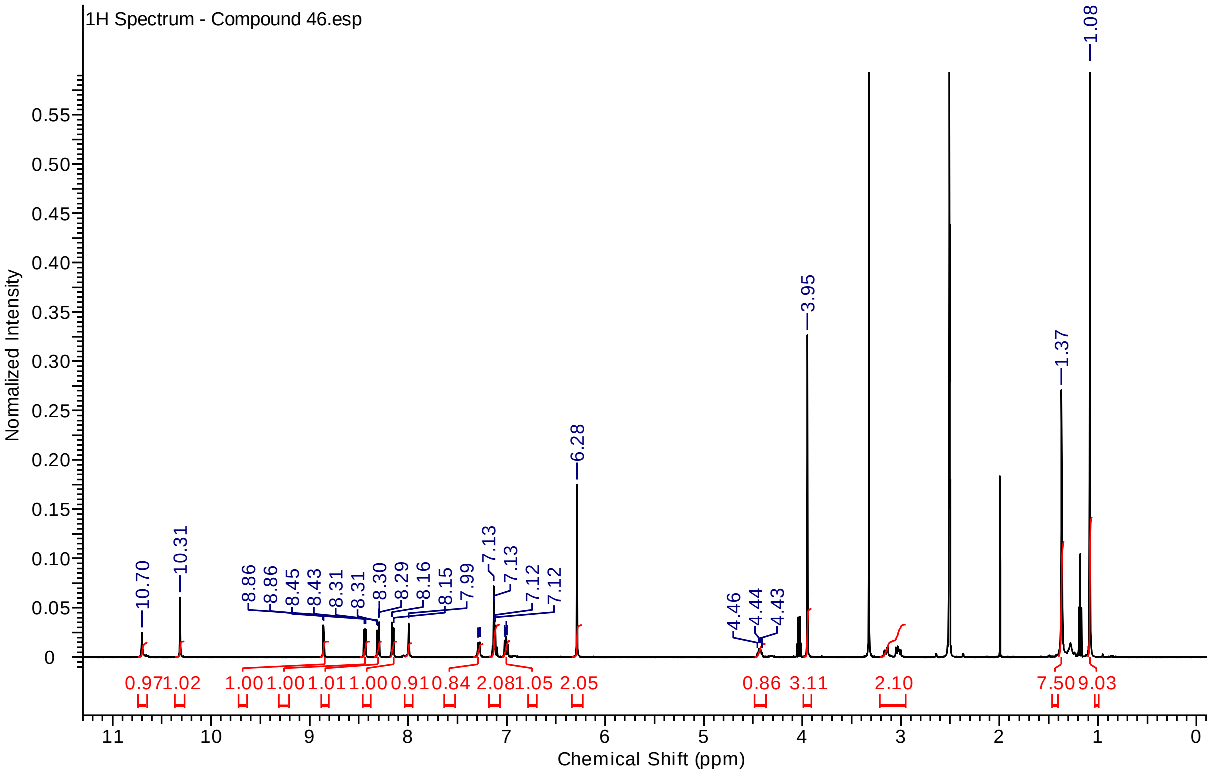
1H Spectrum - Compound 46.esp
Normalized Intensity
Chemical Shift (ppm)
10.70
10.31
8.86
8.86
8.45
8.43
8.31
8.31
8.30
8.29
8.16
8.15
7.99
7.13
7.13
7.12
7.12
6.28
4.46
4.44
4.43
3.95
1.37
1.08
0.97
1.02
1.00
1.00
1.01
1.00
0.91
0.84
2.08
1.05
2.05
0.86
3.11
2.10
7.50
9.03

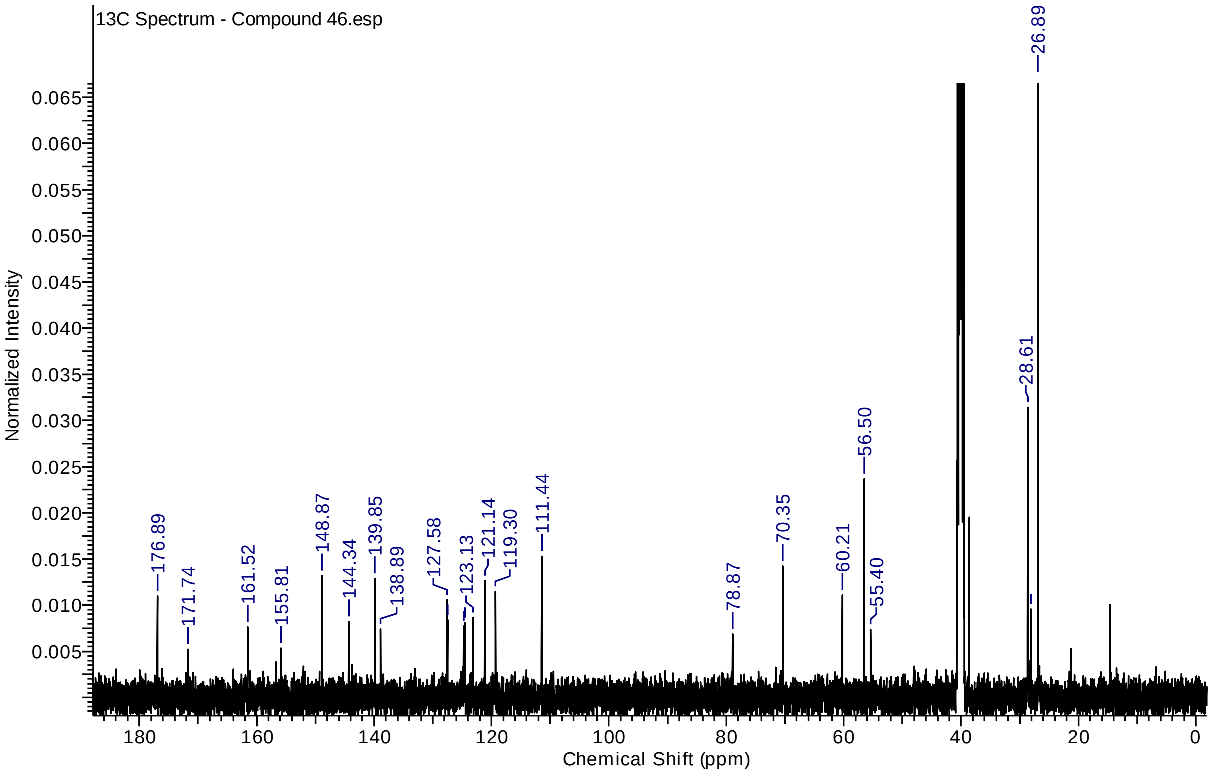
13C Spectrum - Compound 46.esp
Normalized Intensity
Chemical Shift (ppm)
176.89
171.74
161.52
155.81
148.87
144.34
139.85
138.89
127.58
123.13
121.14
119.30
111.44
78.87
70.35
60.21
56.50
55.40
28.61
26.89

Compound 47

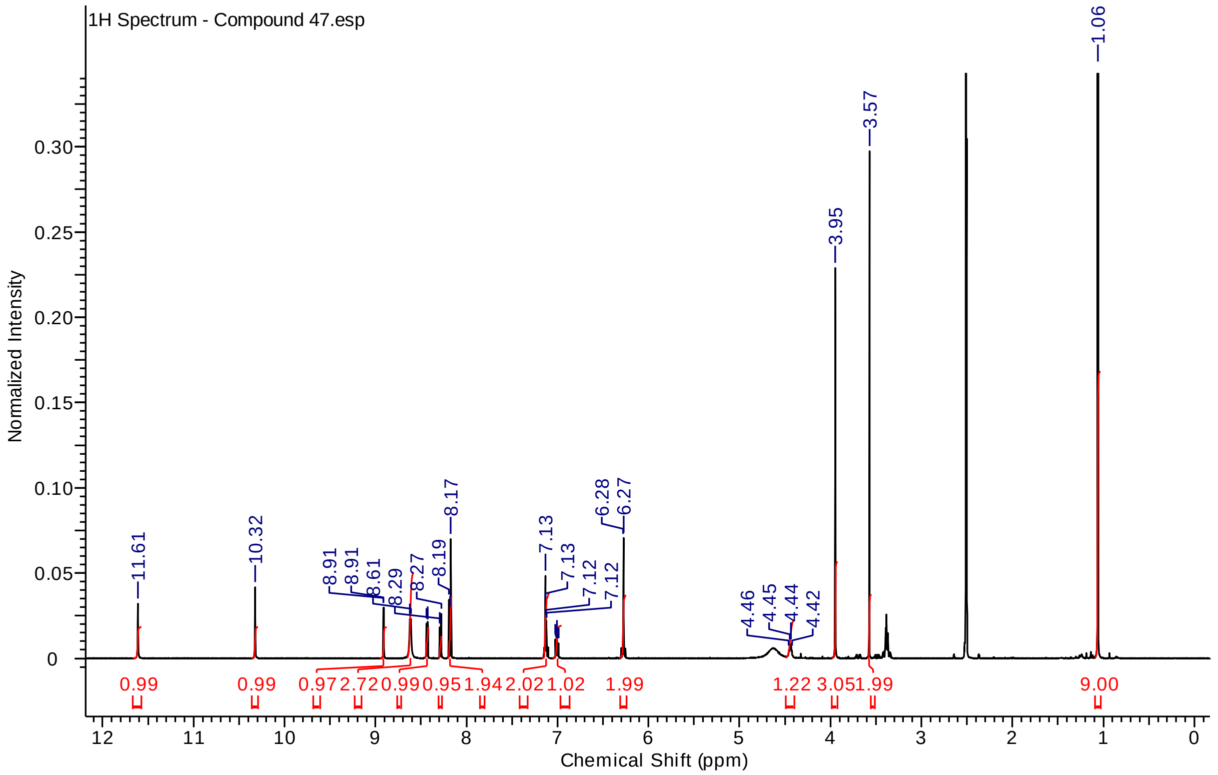
1H Spectrum - Compound 47.esp
Normalized Intensity
Chemical Shift (ppm)

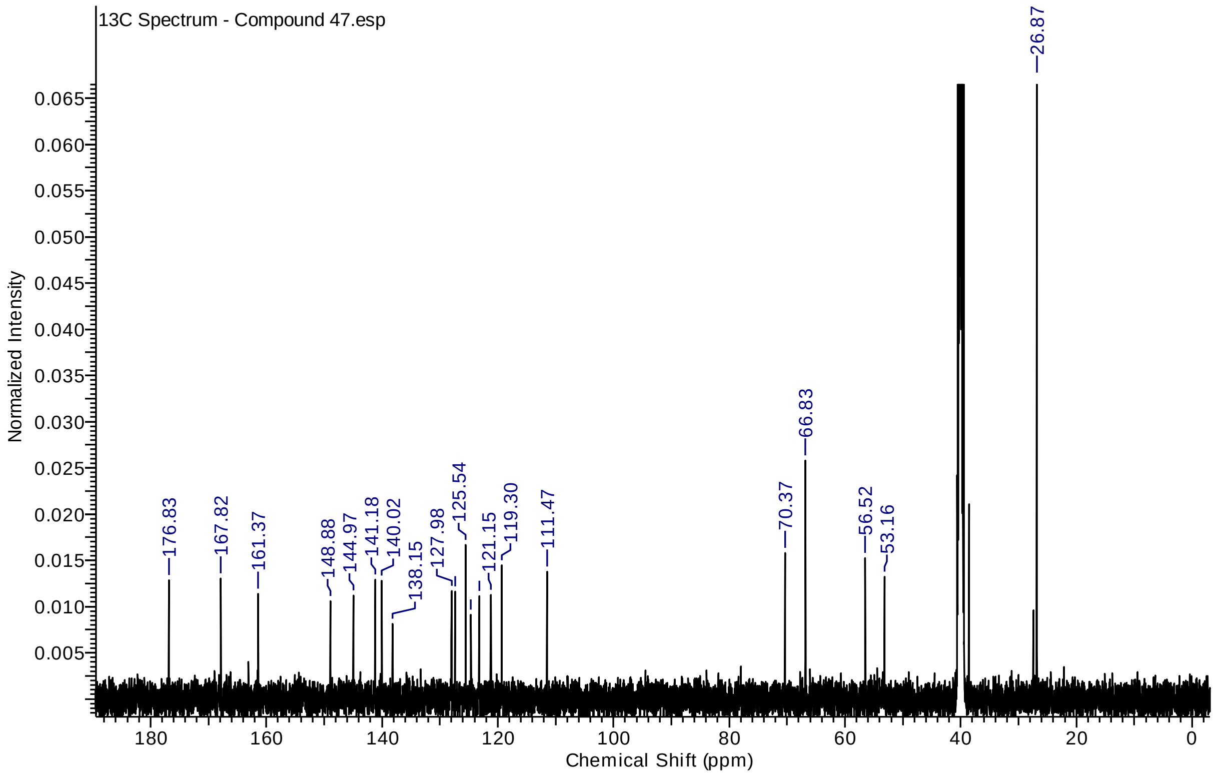
13C Spectrum - Compound 47.esp
Normalized Intensity
Chemical Shift (ppm)

AE-1

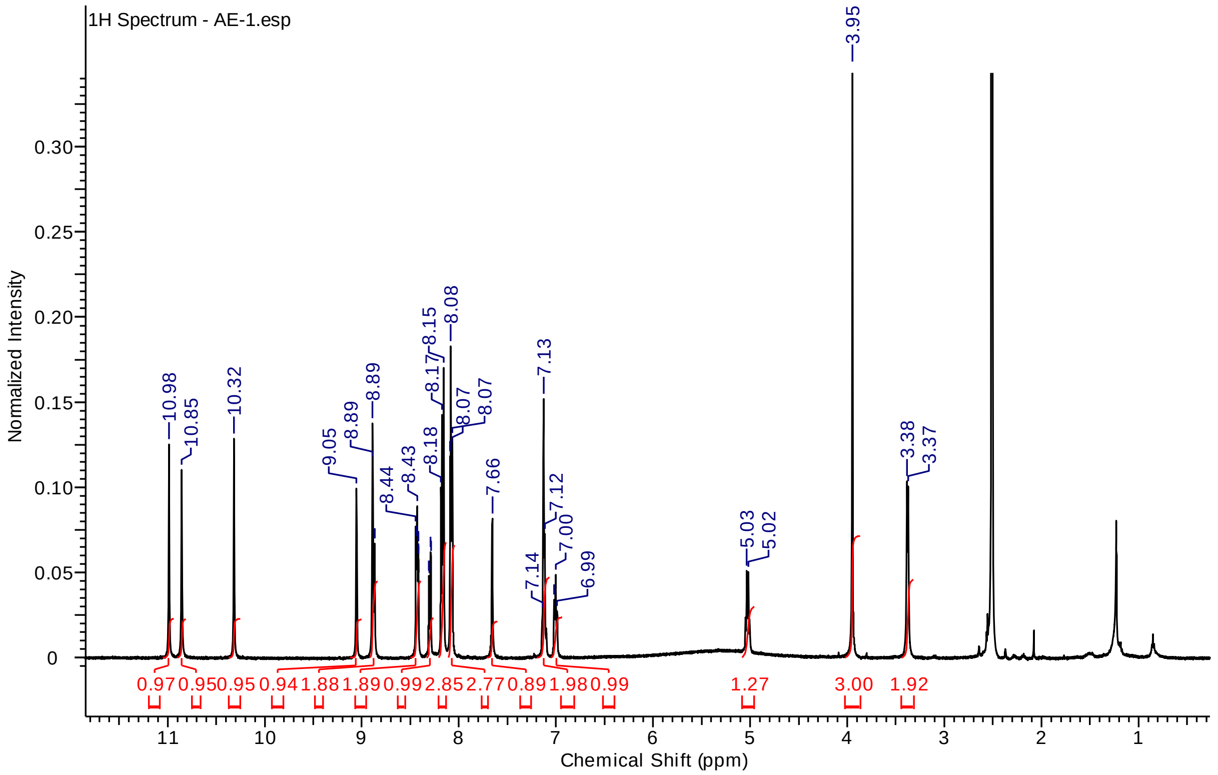
1H Spectrum - AE-1.esp
Normalized Intensity
Chemical Shift (ppm)
10.98
10.85
10.32
9.05
8.89
8.89
8.44
8.43
8.18
8.17
8.15
8.08
8.07
8.07
7.66
7.14
7.13
7.12
7.00
6.99
5.03
5.02
3.95
3.38
3.37
0.97
0.95
0.95
0.94
1.88
1.89
0.99
2.85
2.77
0.89
1.98
0.99
1.27
3.00
1.92

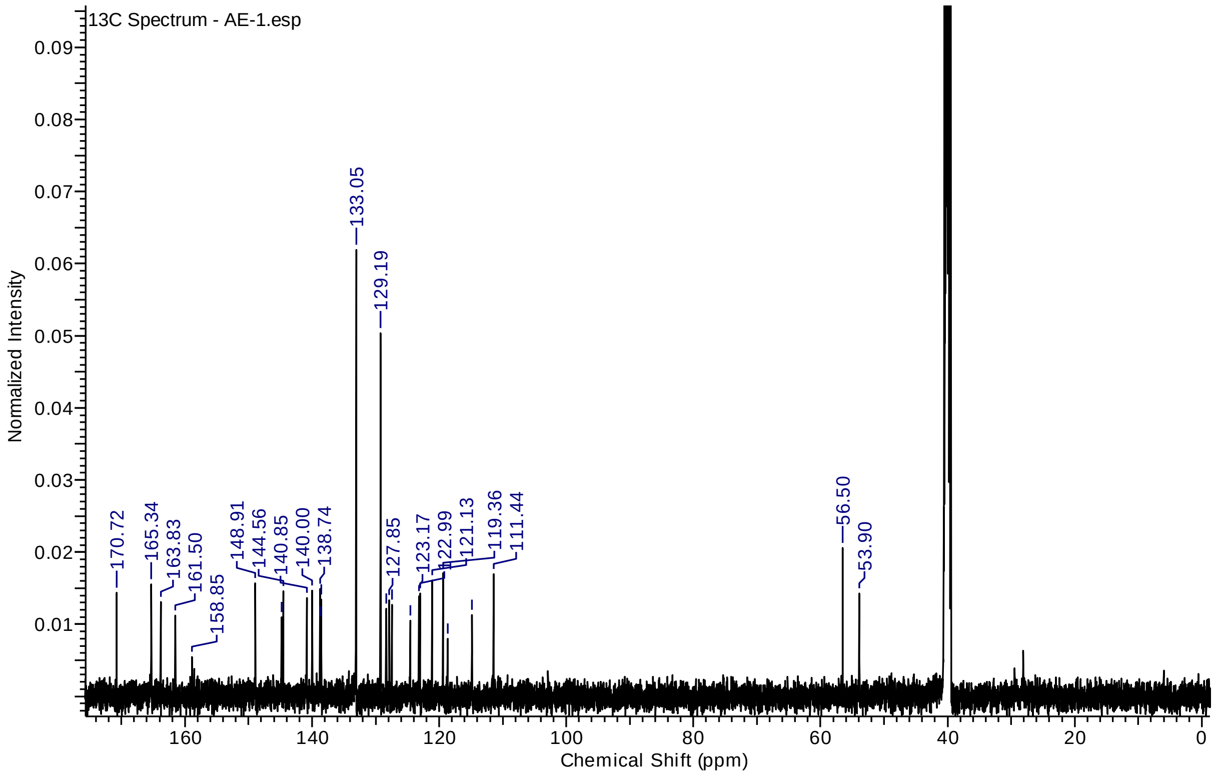
13C Spectrum - AE-1.esp
Normalized Intensity
Chemical Shift (ppm)
170.72
165.34
163.83
161.50
158.85
148.91
144.56
140.85
140.00
138.74
133.05
129.19
127.85
123.17
122.99
121.13
119.36
111.44
56.50
53.90

AE-2

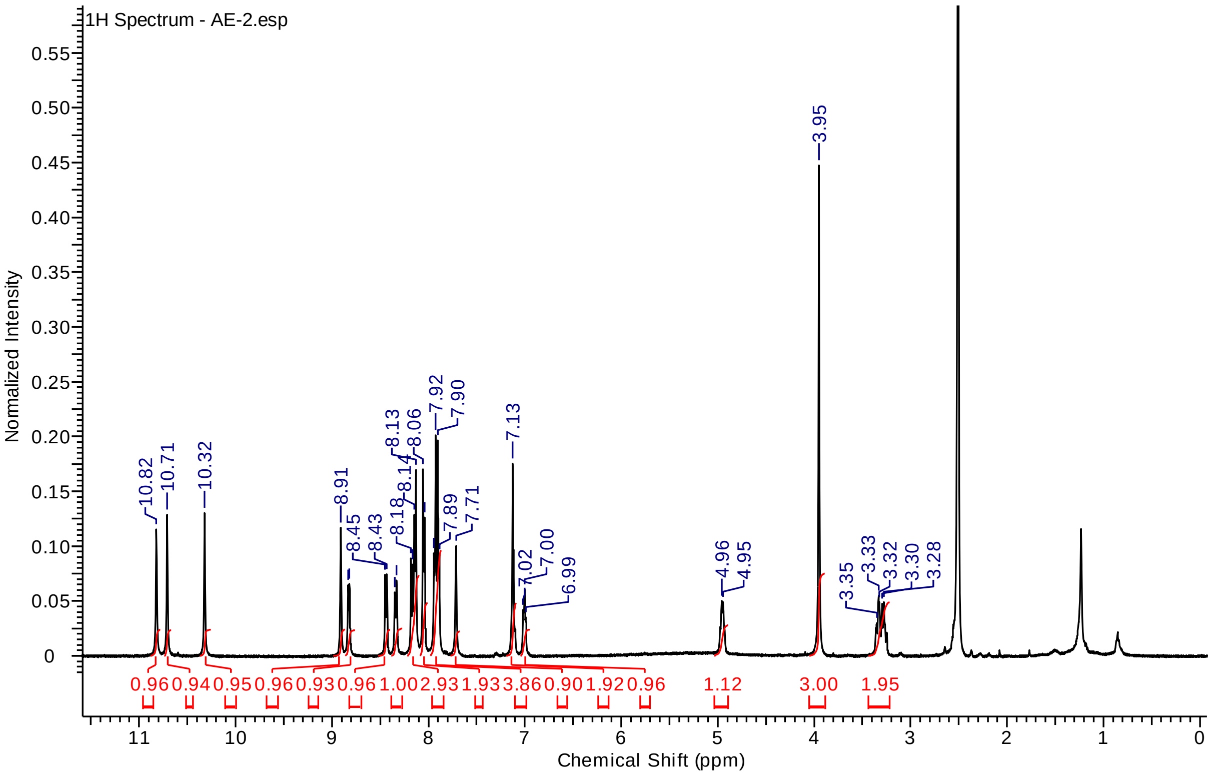
1H Spectrum - AE-2.esp
Normalized Intensity
Chemical Shift (ppm)
10.82
10.71
10.32
8.91
8.45
8.43
8.18
8.14
8.13
8.06
7.92
7.90
7.89
7.71
7.13
7.02
7.00
6.99
4.96
4.95
3.95
3.35
3.33
3.32
3.30
3.28
0.96
0.94
0.95
0.96
0.93
0.96
1.00
2.93
1.93
3.86
0.90
1.92
0.96
1.12
3.00
1.95

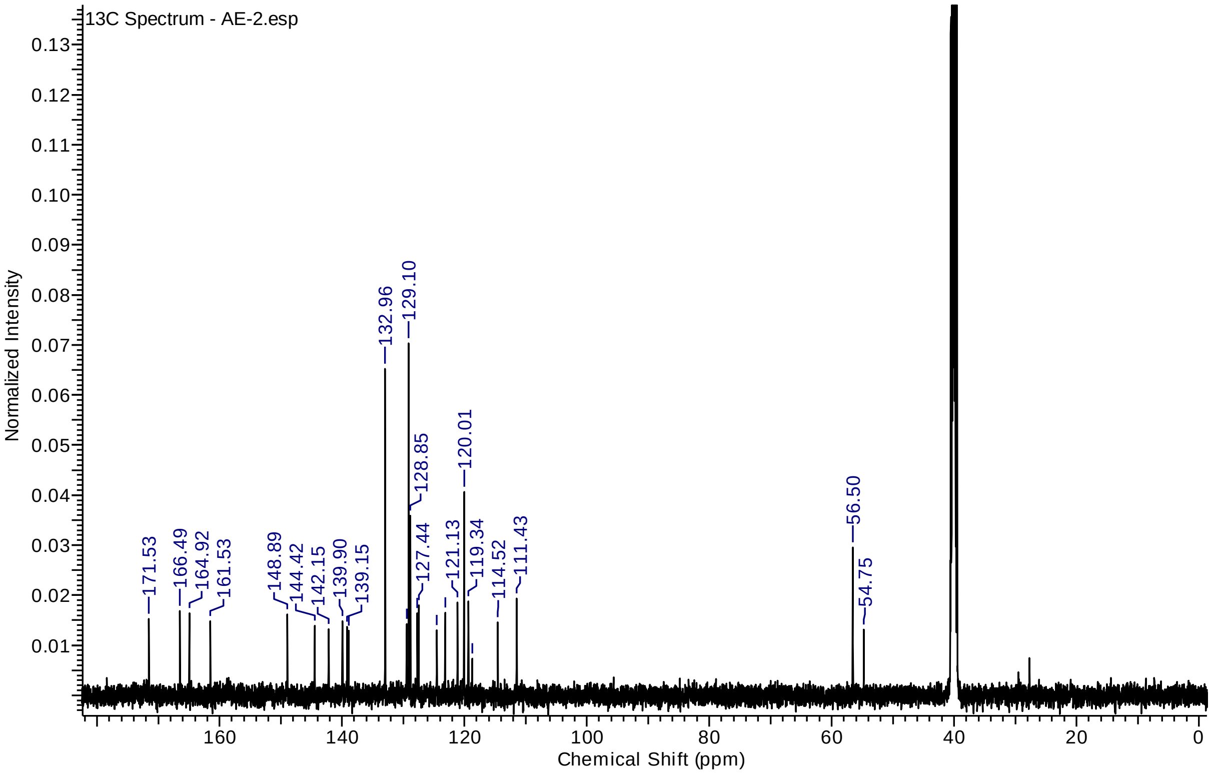
13C Spectrum - AE-2.esp
Normalized Intensity
Chemical Shift (ppm)
171.53
166.49
164.92
161.53
148.89
144.42
142.15
139.90
139.15
132.96
129.10
128.85
127.44
121.13
120.01
119.34
114.52
111.43
56.50
54.75

AE-3

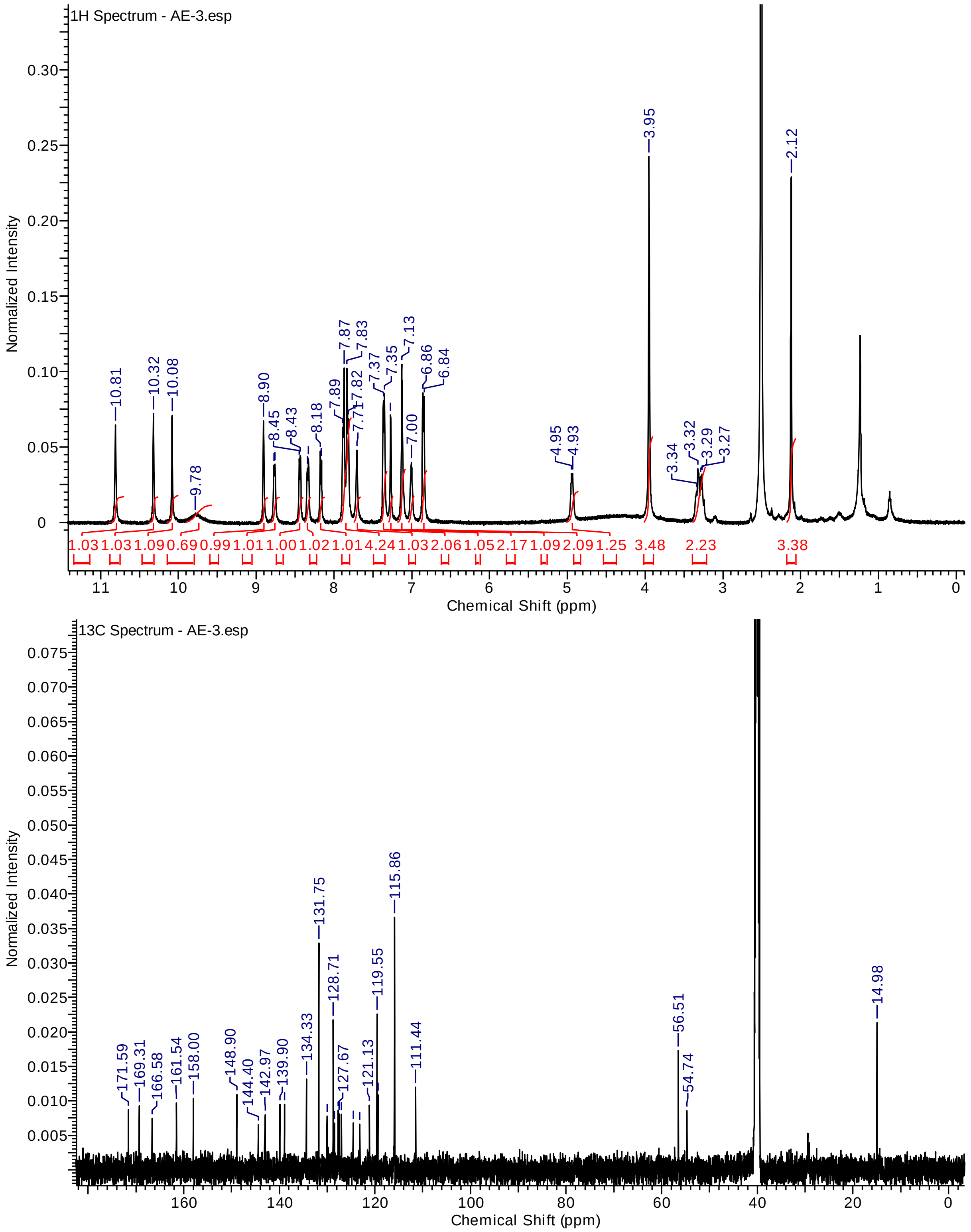
1H Spectrum - AE-3.esp
Normalized Intensity
Chemical Shift (ppm)
13C Spectrum - AE-3.esp
Normalized Intensity
Chemical Shift (ppm)

Compound 51

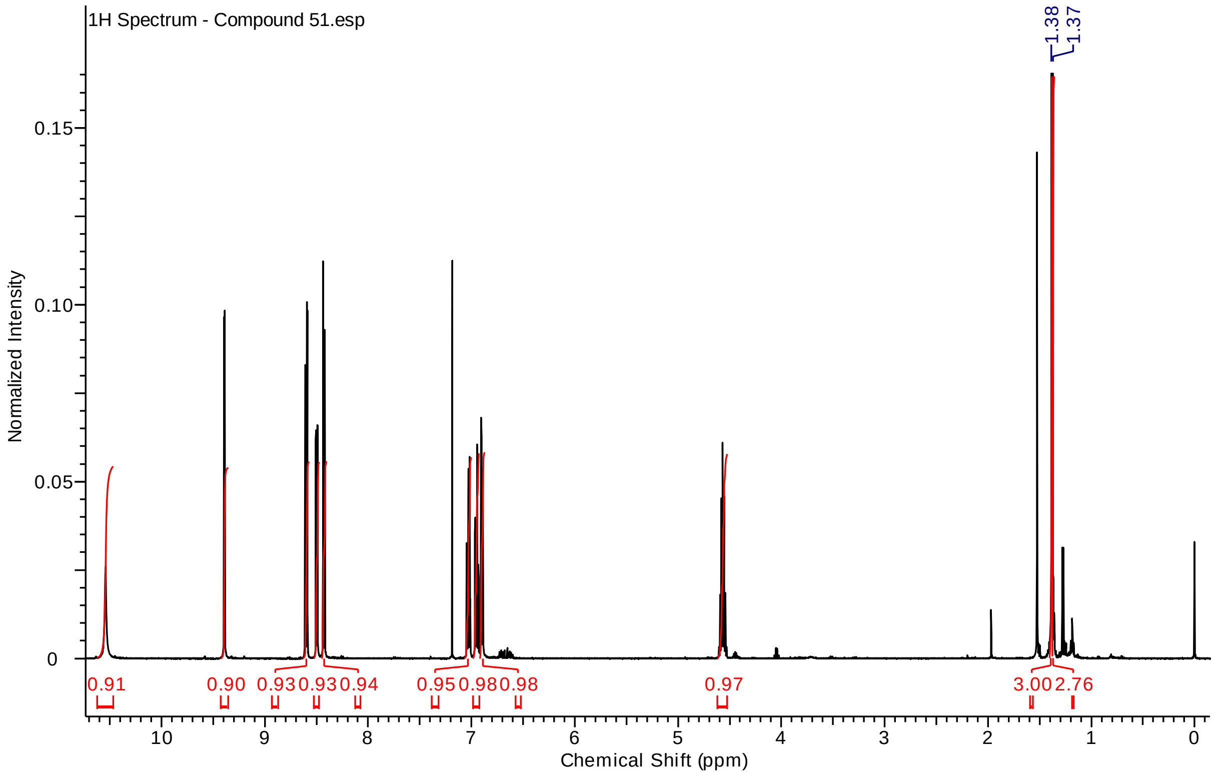
1H Spectrum - Compound 51.esp
1.38
1.37
Normalized Intensity
0.15
0.10
0.05
0
0.91
0.90
0.93
0.93
0.94
0.95
0.98
0.98
0.97
3.00
2.76
10
9
8
7
6
5
4
3
2
1
0
Chemical Shift (ppm)

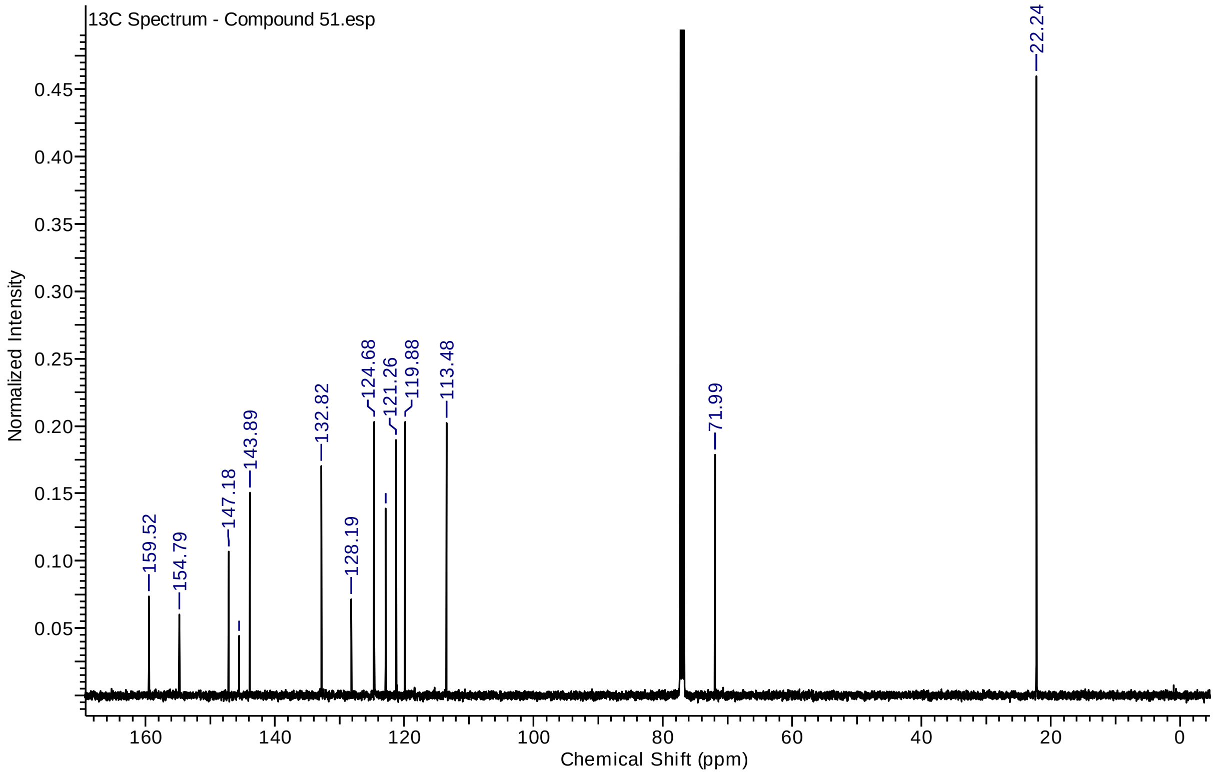
13C Spectrum - Compound 51.esp
22.24
159.52
154.79
147.18
143.89
132.82
128.19
124.68
121.26
119.88
113.48
71.99
Normalized Intensity
0.45
0.40
0.35
0.30
0.25
0.20
0.15
0.10
0.05
160
140
120
100
80
60
40
20
0
Chemical Shift (ppm)

Compound 53

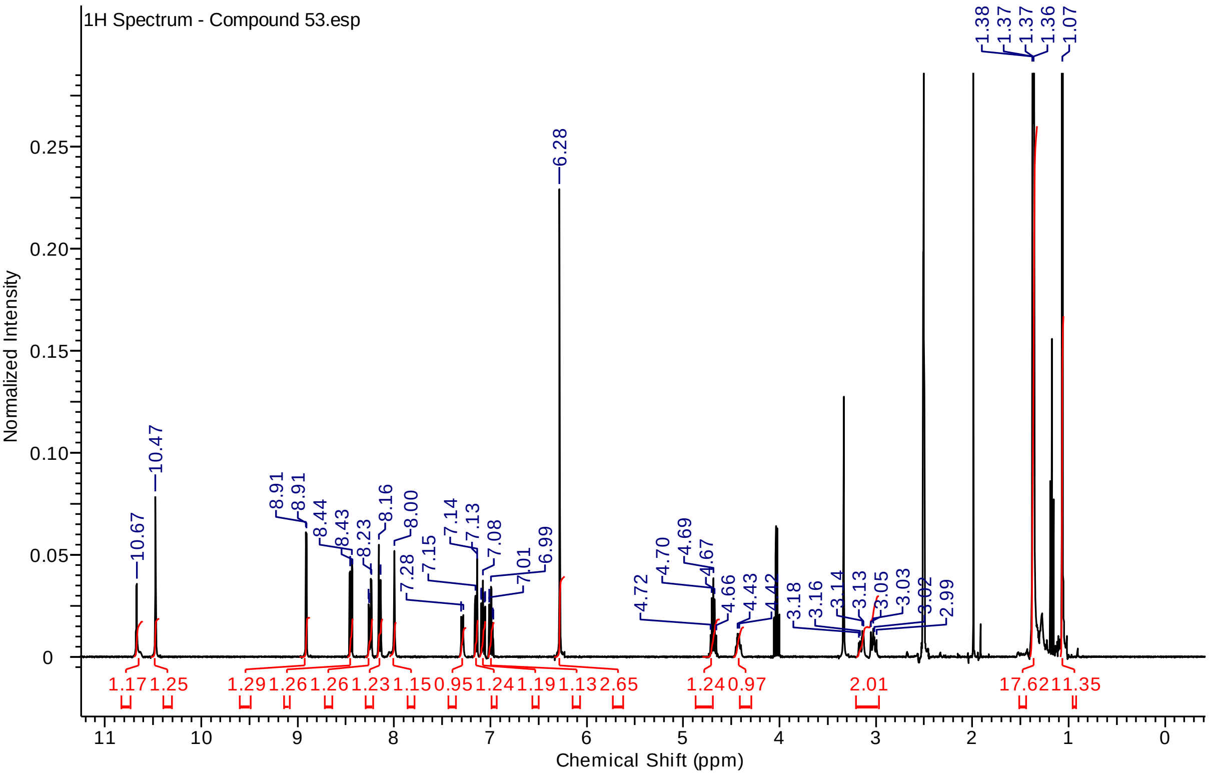
1H Spectrum - Compound 53.esp
Normalized Intensity
Chemical Shift (ppm)

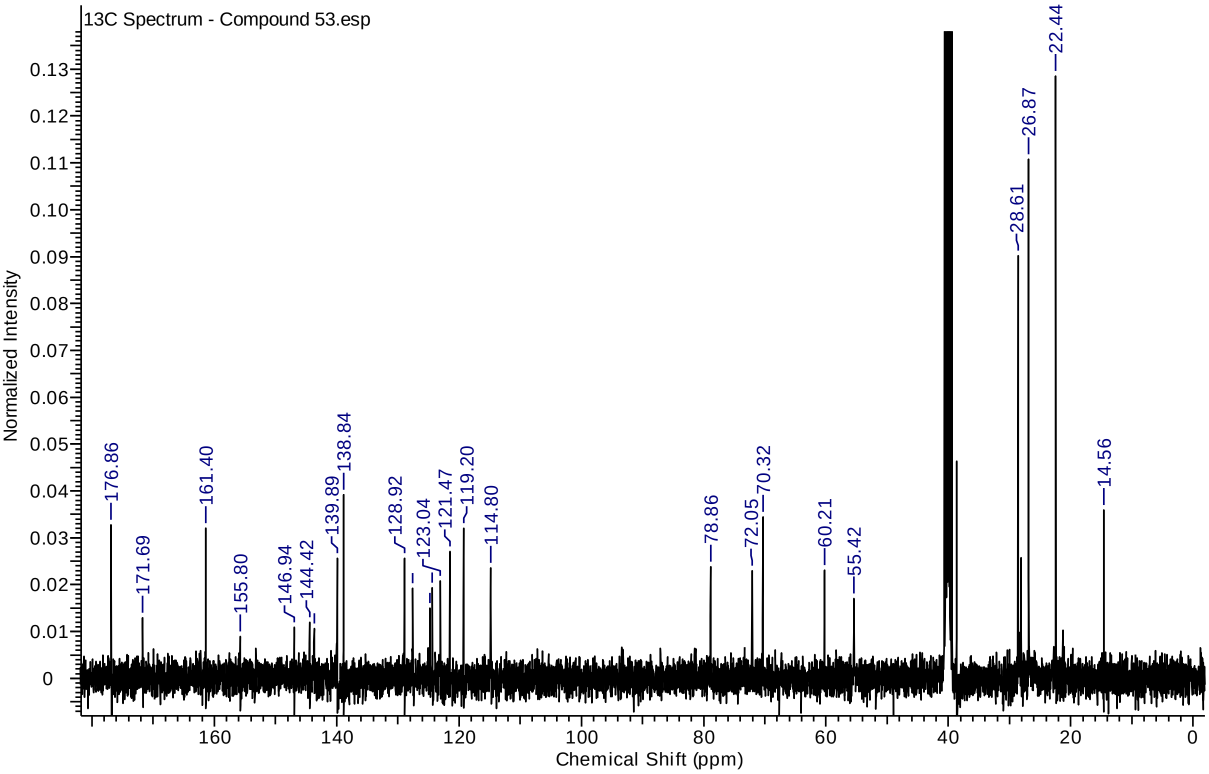
13C Spectrum - Compound 53.esp
Normalized Intensity
Chemical Shift (ppm)

AE-4

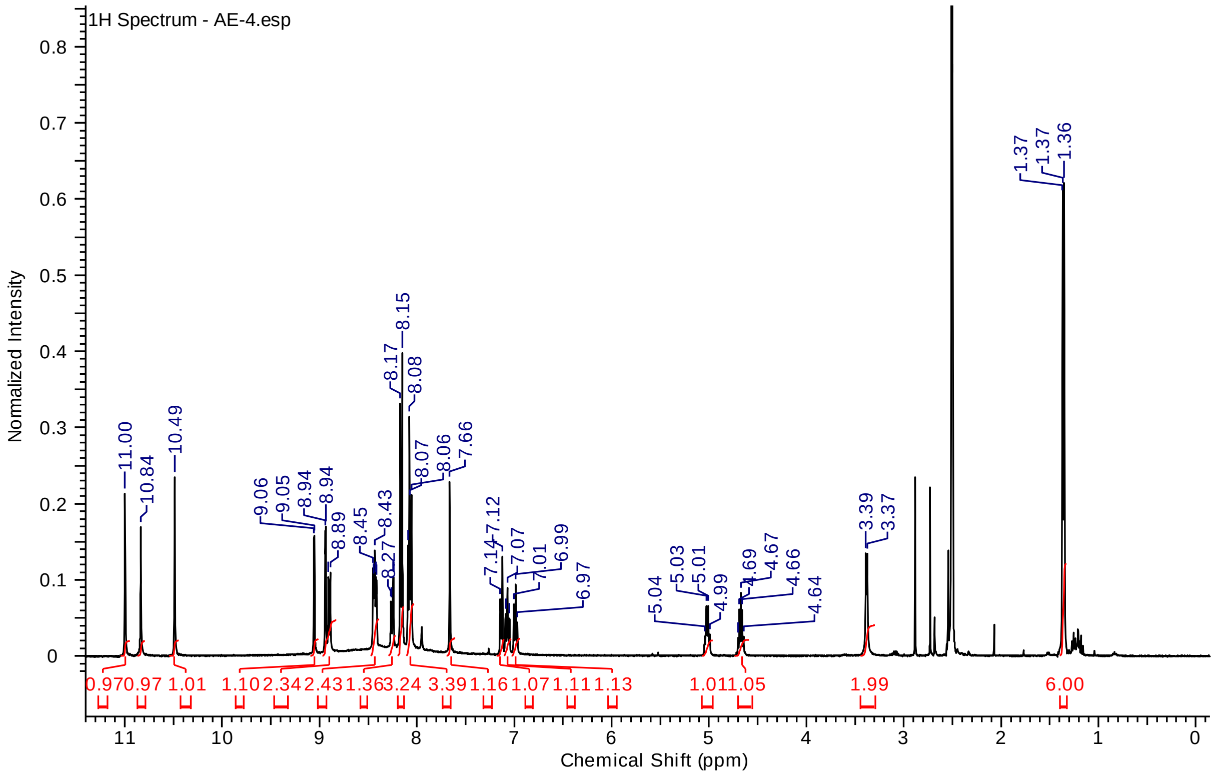
1H Spectrum - AE-4.esp
Normalized Intensity
Chemical Shift (ppm)

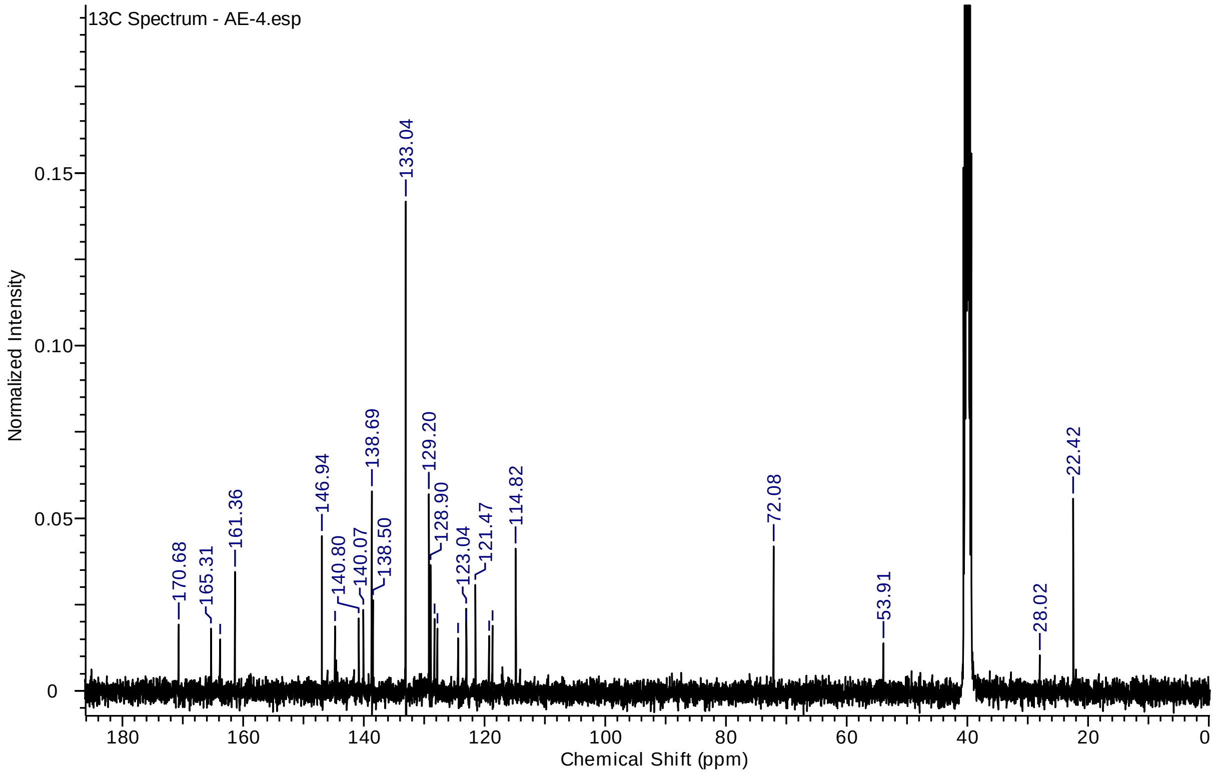
13C Spectrum - AE-4.esp
Normalized Intensity
Chemical Shift (ppm)

Compound 56

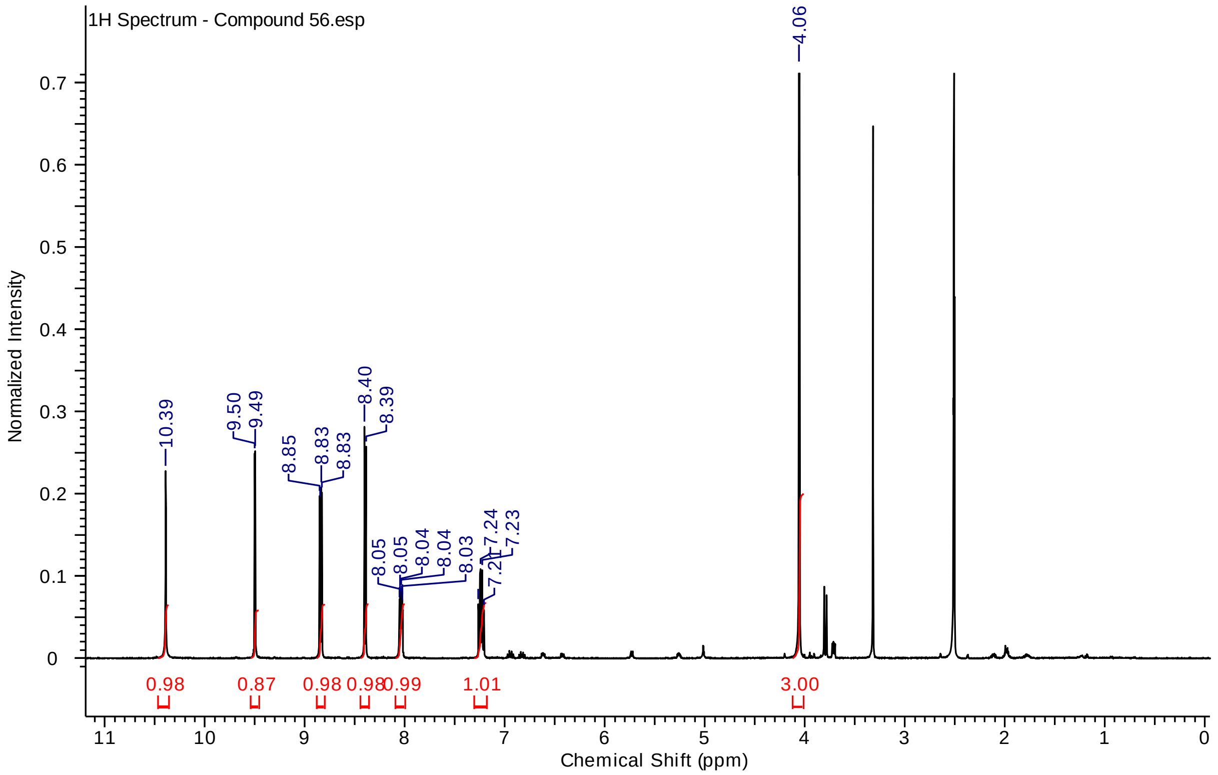
1H Spectrum - Compound 56.esp
Normalized Intensity
Chemical Shift (ppm)
10.39
9.50
9.49
8.85
8.83
8.83
8.40
8.39
8.05
8.05
8.04
8.04
8.03
7.24
7.23
7.21
4.06
0.98
0.87
0.98
0.98
0.99
1.01
3.00

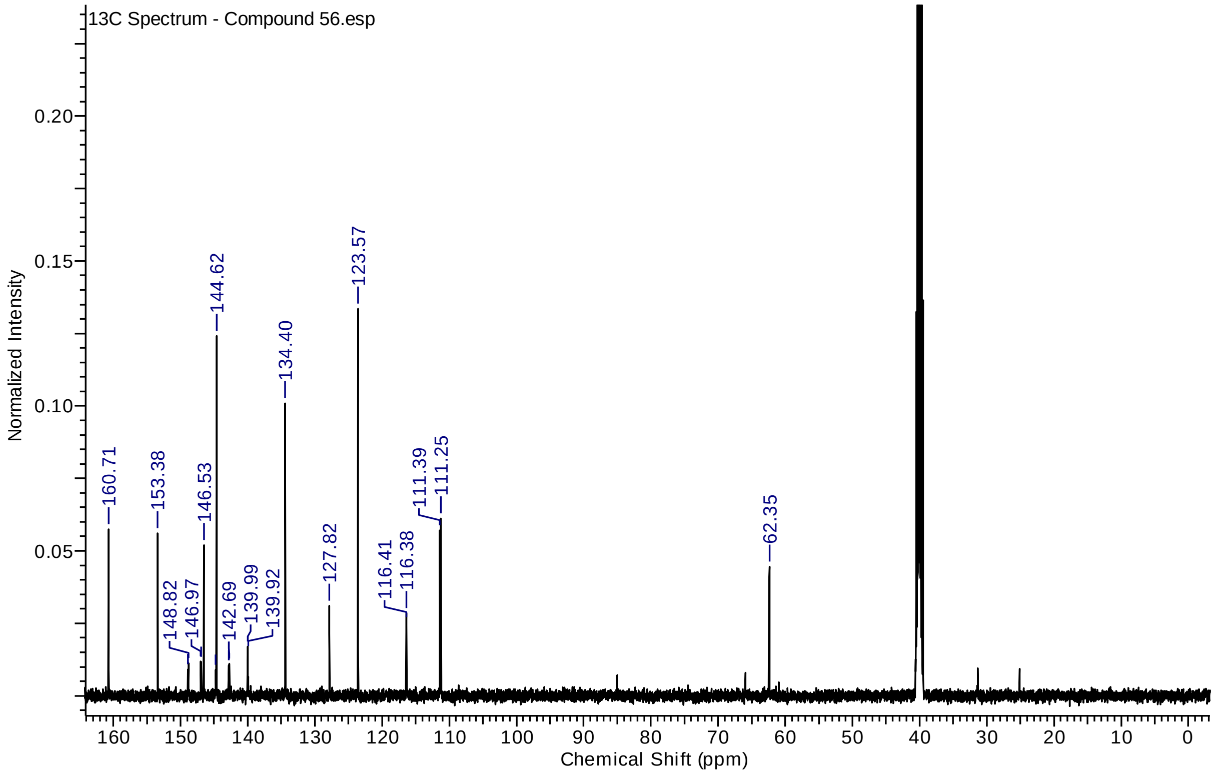
13C Spectrum - Compound 56.esp
Normalized Intensity
Chemical Shift (ppm)
160.71
153.38
148.82
146.97
146.53
144.62
142.69
139.99
139.92
134.40
127.82
123.57
116.41
116.38
111.39
111.25
62.35

Compound 57

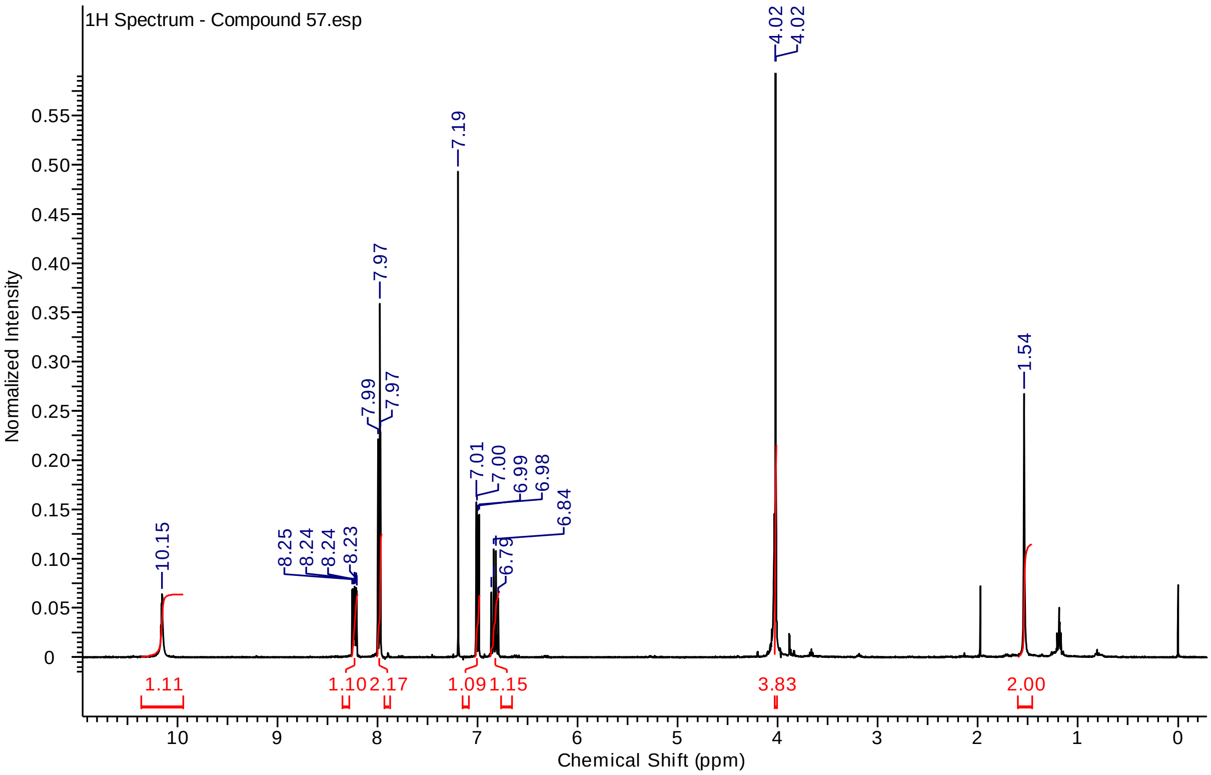
1H Spectrum - Compound 57.esp
Normalized Intensity
Chemical Shift (ppm)
10.15
8.25
8.24
8.24
8.23
7.99
7.97
7.97
7.19
7.01
7.00
6.99
6.98
6.84
6.79
4.02
4.02
1.54
1.11
1.10
2.17
1.09
1.15
3.83
2.00

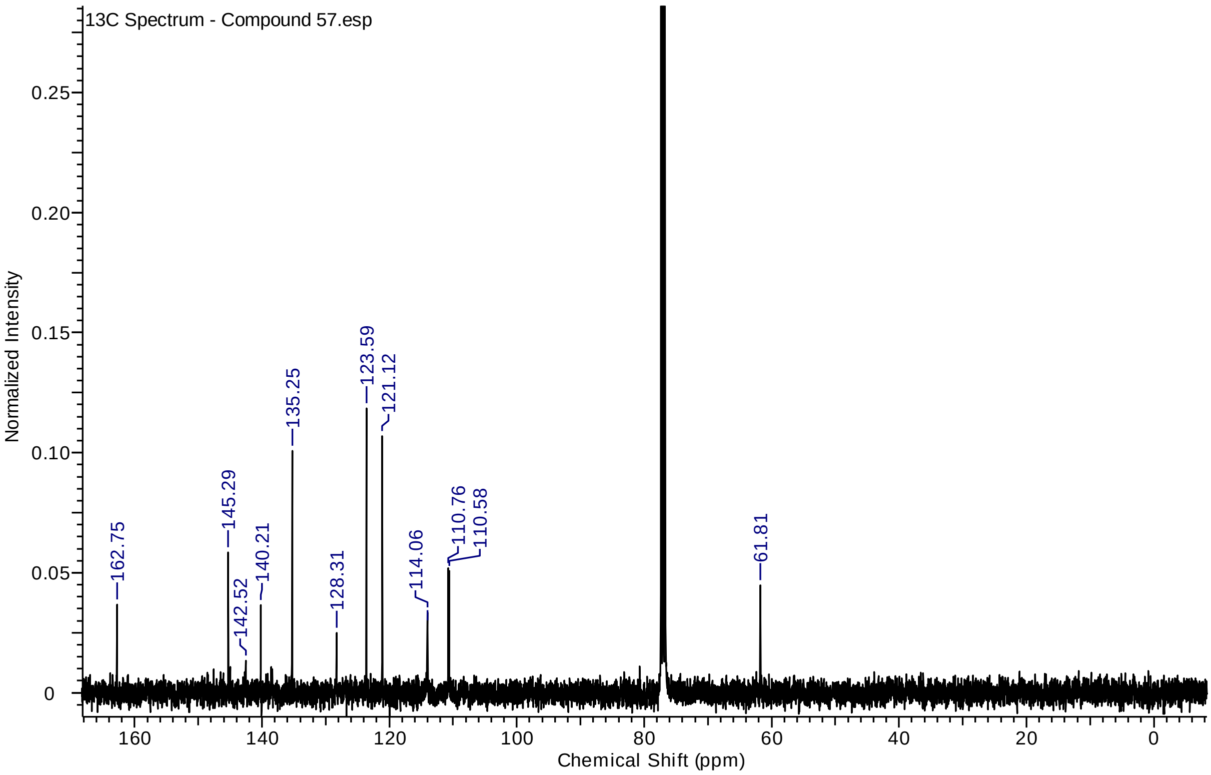
13C Spectrum - Compound 57.esp
Normalized Intensity
Chemical Shift (ppm)
162.75
145.29
142.52
140.21
135.25
128.31
123.59
121.12
114.06
110.76
110.58
61.81

Compound 58

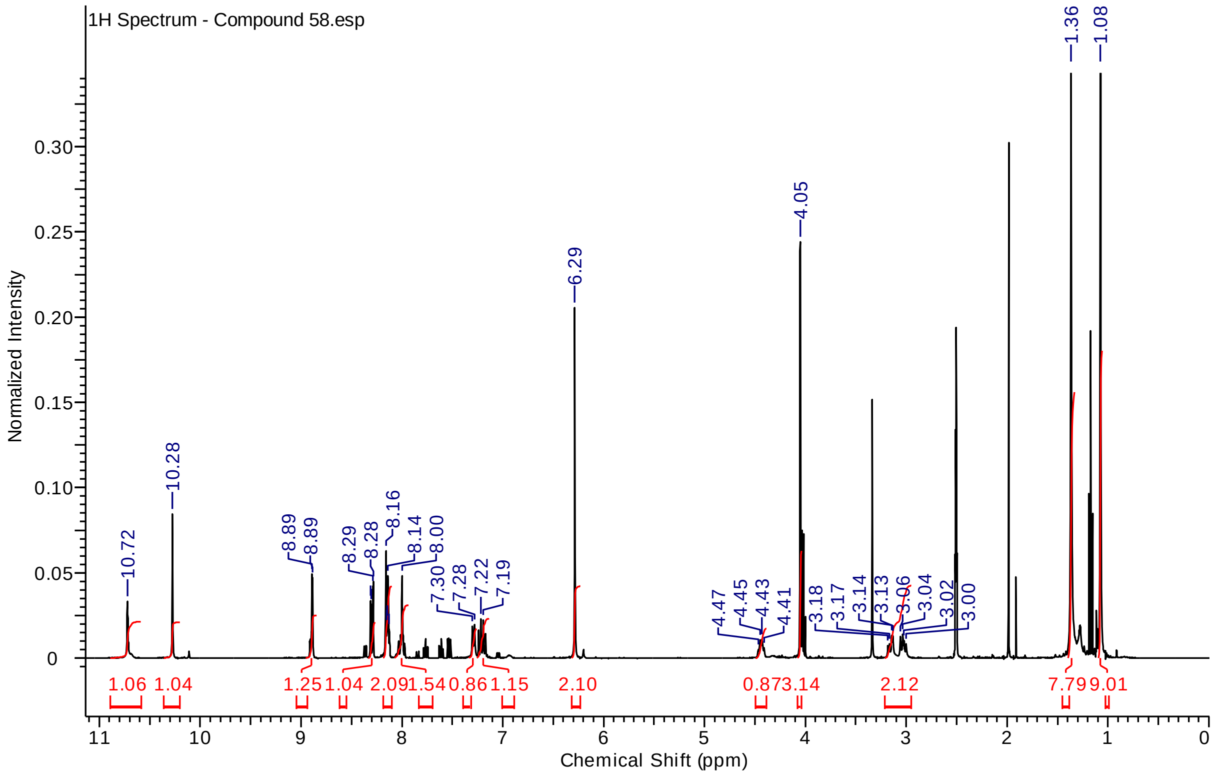

1H Spectrum - Compound 58.esp
Normalized Intensity
Chemical Shift (ppm)
10.72
10.28
8.89
8.89
8.29
8.28
8.16
8.14
8.00
7.30
7.28
7.22
7.19
6.29
4.47
4.45
4.43
4.41
4.05
3.18
3.17
3.14
3.13
3.06
3.04
3.02
3.00
1.36
1.08
1.06
1.04
1.25
1.04
2.09
1.54
0.86
1.15
2.10
0.87
3.14
2.12
7.79
9.01

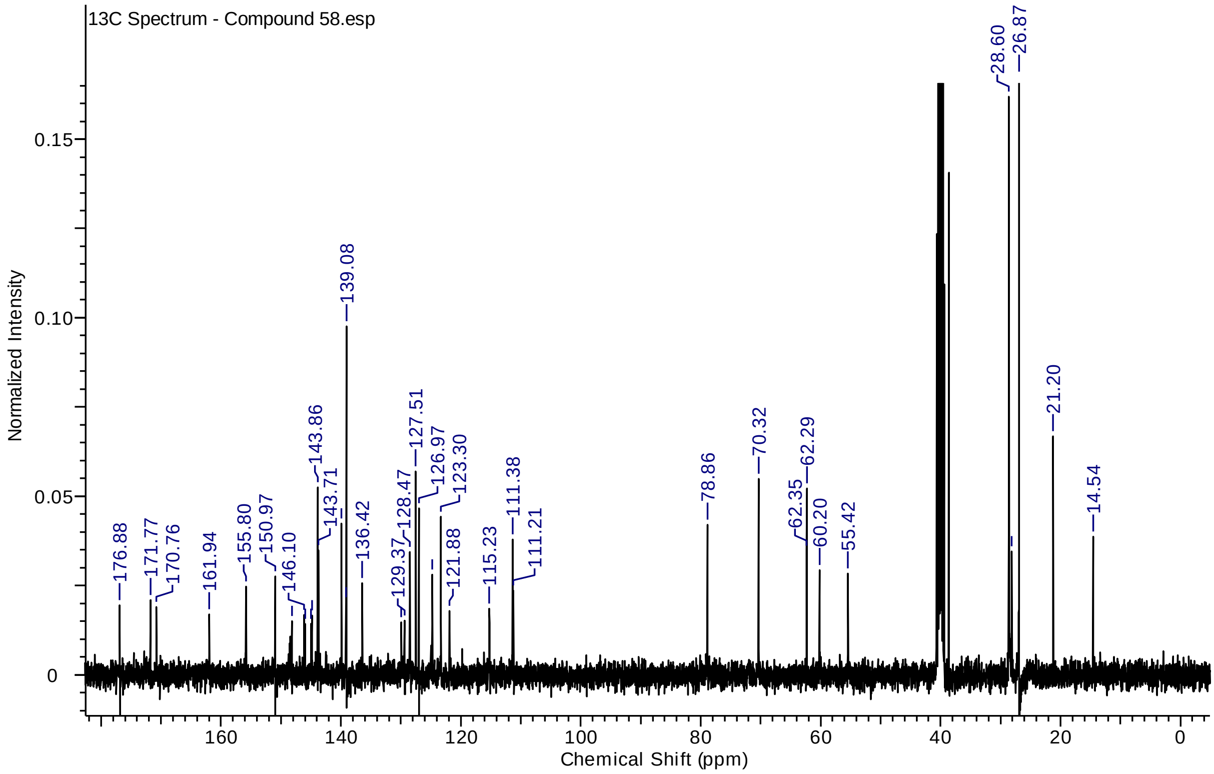

13C Spectrum - Compound 58.esp
Normalized Intensity
Chemical Shift (ppm)
176.88
171.77
170.76
161.94
155.80
150.97
146.10
143.86
143.71
139.08
136.42
129.37
128.47
127.51
126.97
123.30
121.88
115.23
111.38
111.21
78.86
70.32
62.35
62.29
60.20
55.42
28.60
26.87
21.20
14.54

AE-5

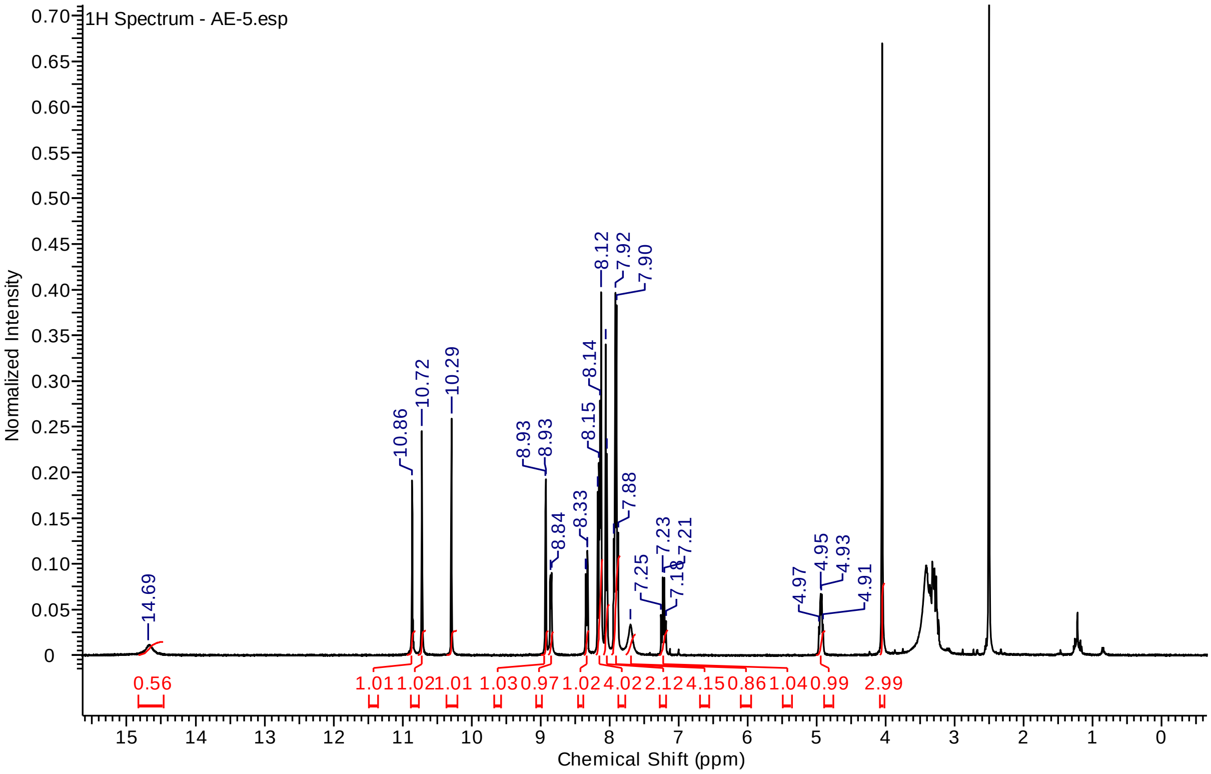
1H Spectrum - AE-5.esp
Normalized Intensity
Chemical Shift (ppm)
14.69
10.86
10.72
10.29
8.93
8.93
8.84
8.33
8.15
8.14
8.12
7.92
7.90
7.88
7.25
7.23
7.21
7.18
4.97
4.95
4.93
4.91
0.56
1.01
1.02
1.01
1.03
0.97
1.02
4.02
2.12
4.15
0.86
1.04
0.99
2.99

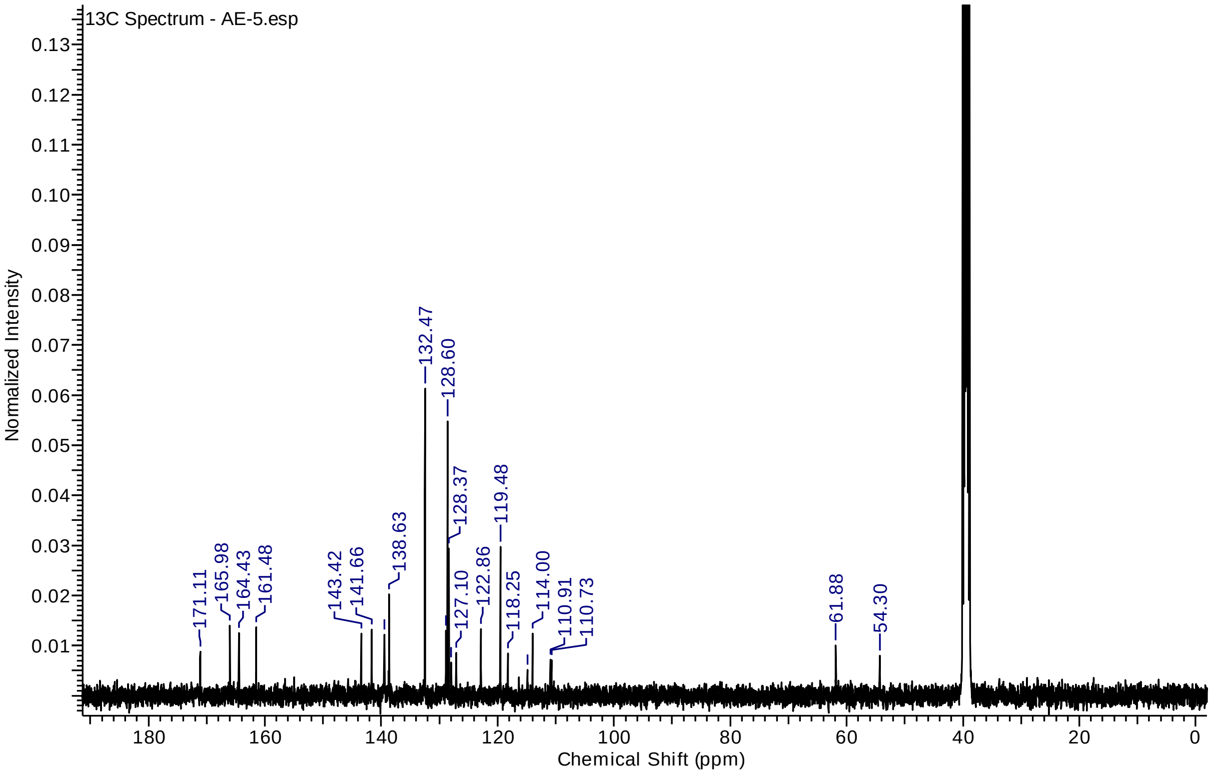
13C Spectrum - AE-5.esp
Normalized Intensity
Chemical Shift (ppm)
171.11
165.98
164.43
161.48
143.42
141.66
138.63
132.47
128.60
128.37
127.10
122.86
119.48
118.25
114.00
110.91
110.73
61.88
54.30

Supplementary References

1. Zborovsky, L. *et al.* Improvement of the antimicrobial potency, pharmacokinetic and pharmacodynamic properties of albicidin by incorporation of nitrogen atoms. *Chem. Sci.* **12**, 14606-14617, doi:10.1039/D1SC04019G (2021).

2. Behroz, I. *et al.* Extensive Structure-Activity Relationship Study of Albicidin's C-Terminal Dipeptidic p-Aminobenzoic Acid Moiety. *Eur. J. Chem.* **25**, 16538-16543, doi:10.1002/chem.201904752 (2019).

3. Moeller, M. *et al.* Scalable Syntheses of Methoxyaspartate and Preparation of the Antibiotic Cystobactamid 861-2 and Highly Potent Derivatives. *Org. Lett.* **21**, 8369-8372, doi:10.1021/acs.orglett.9b03143 (2019).

4. Behroz, I. *et al.* Acetylenic Replacement of Albicidin's Methacrylamide Residue Circumvents Detrimental E/Z Photoisomerization and Preserves Antibacterial Activity. *Eur. J. Chem.* **27**, 9077-9086, doi:10.1002/chem.202100523 (2021)
