# Supplementary material for: Molecular mechanism of topoisomerase poisoning by the peptide antibiotic albicidin
Source: Nat Catal. 2023 Jan 23;6(1):52–67. doi: 10.1038/s41929-022-00904-1 (PMC9886550; doi:10.1038/s41929-022-00904-1)
Supplement: Supplementary file 2 — Reporting Summary [file 41929_2022_904_MOESM2_ESM.pdf]

Corresponding author(s): Ghilarov, Dmitry; Heddle, Jonathan G.; Suessmuth, Roderich D.

Last updated by author(s): Oct 11, 2022

Reporting Summary

Nature Portfolio wishes to improve the reproducibility of the work that we publish. This form provides structure for consistency and transparency in reporting. For further information on Nature Portfolio policies, see our Editorial Policies and the Editorial Policy Checklist.

Statistics

For all statistical analyses, confirm that the following items are present in the figure legend, table legend, main text, or Methods section.

n/a	Confirmed	
☐	☒	The exact sample size (*n*) for each experimental group/condition, given as a discrete number and unit of measurement
☒	☐	A statement on whether measurements were taken from distinct samples or whether the same sample was measured repeatedly
☒	☐	The statistical test(s) used AND whether they are one- or two-sided<br>*Only common tests should be described solely by name; describe more complex techniques in the Methods section.*
☒	☐	A description of all covariates tested
☒	☐	A description of any assumptions or corrections, such as tests of normality and adjustment for multiple comparisons
☐	☒	A full description of the statistical parameters including central tendency (e.g. means) or other basic estimates (e.g. regression coefficient) AND variation (e.g. standard deviation) or associated estimates of uncertainty (e.g. confidence intervals)
☒	☐	For null hypothesis testing, the test statistic (e.g. *F*, *t*, *r*) with confidence intervals, effect sizes, degrees of freedom and *P* value noted<br>*Give P values as exact values whenever suitable.*
☒	☐	For Bayesian analysis, information on the choice of priors and Markov chain Monte Carlo settings
☒	☐	For hierarchical and complex designs, identification of the appropriate level for tests and full reporting of outcomes
☒	☐	Estimates of effect sizes (e.g. Cohen's *d*, Pearson's *r*), indicating how they were calculated

Our web collection on statistics for biologists contains articles on many of the points above.

Software and code

Policy information about availability of computer code

Data collection	EPU version 2.10.0.1941REL
Data analysis	cryoSPARC version 3.3.1; Relion version 3.1; Phenix v 1.19.2-4158; Coot for Windows version 0.9.6 EL; Origin v.2021b; ImageJ 1.53k; ChimeraX 1.3 including ISOLDE v.1.0b3 plugin.

For manuscripts utilizing custom algorithms or software that are central to the research but not yet described in published literature, software must be made available to editors and reviewers. We strongly encourage code deposition in a community repository (e.g. GitHub). See the Nature Portfolio guidelines for submitting code & software for further information.

Data

Policy information about availability of data

All manuscripts must include a data availability statement. This statement should provide the following information, where applicable:

- Accession codes, unique identifiers, or web links for publicly available datasets
- A description of any restrictions on data availability
- For clinical datasets or third party data, please ensure that the statement adheres to our policy

All data needed to evaluate the conclusions in the paper are present in the paper and/or the Supplementary Materials or from the authors upon reasonable request. The Gyr-Mu217-Albi, Gyr-Mu217-Albi1-TG, Gyr-Mu217-Albi1-AA and Gyr-Mu217-Albi2 coordinates have been submitted to the Protein Data Bank (https://www.rcsb.org/) with PDB IDs 7Z9C, 7Z9K, 7Z9M and 7Z9G, respectively. Final EM maps of have been submitted to Electron Microscopy Data Bank (https://

www.ebi.ac.uk/pdbe/emdb/) with IDs EMD-14570, EMD-14573, EMD-14574, and EMD-14572, respectively. Raw data were submitted to Electron Microscopy Public Image Archive (https://www.ebi.ac.uk/pdbe/emdb/empiar/) with IDs EMPIAR-11244 (albicidin), EMPIAR-11245 (Albi-1) and EMPIAR-11246 (Albi-2).

Human research participants

Policy information about studies involving human research participants and Sex and Gender in Research.

Reporting on sex and gender	N/A
Population characteristics	N/A
Recruitment	N/A
Ethics oversight	N/A

Note that full information on the approval of the study protocol must also be provided in the manuscript.

Field-specific reporting

Please select the one below that is the best fit for your research. If you are not sure, read the appropriate sections before making your selection.

☒ Life sciences ☐ Behavioural & social sciences ☐ Ecological, evolutionary & environmental sciences

For a reference copy of the document with all sections, see nature.com/documents/nr-reporting-summary-flat.pdf

Life sciences study design

All studies must disclose on these points even when the disclosure is negative.

Sample size	No statistical methods were used to pre-determine the sample size. Sample size (N ≥ 2) for biochemical assays or MIC assays was chosen according to the relevant literature. Number of collected cryo-EM movies was based on the microscope availability and expected resolution
Data exclusions	No data were excluded
Replication	MIC experiments and CC50 measurements have been done in triplicates. All other biochemical assays were confirmed by at least one replicate as described in the manuscript. All attempts at replication were successful
Randomization	All bacterial cultures were grown under identical conditions therefore randomization was not relevant for this study
Blinding	Blinding is not applicable for this study as it does not involve any subject assessment of the data that may influence validity of results

Reporting for specific materials, systems and methods

We require information from authors about some types of materials, experimental systems and methods used in many studies. Here, indicate whether each material, system or method listed is relevant to your study. If you are not sure if a list item applies to your research, read the appropriate section before selecting a response.

Materials & experimental systems

n/a	Involved in the study	
☒	☐	Antibodies
☒	☐	Eukaryotic cell lines
☒	☐	Palaeontology and archaeology
☒	☐	Animals and other organisms
☒	☐	Clinical data
☒	☐	Dual use research of concern

Methods

n/a	Involved in the study	
☒	☐	ChIP-seq
☒	☐	Flow cytometry
☒	☐	MRI-based neuroimaging
